# Patient-Derived Bone Marrow Spheroids Reveal Leukemia-Initiating Cells Supported by Mesenchymal Hypoxic Niches in Pediatric B-ALL

**DOI:** 10.3389/fimmu.2021.746492

**Published:** 2021-10-19

**Authors:** Juan Carlos Balandrán, José Dávila-Velderrain, Antonio Sandoval-Cabrera, Gabriela Zamora-Herrera, Vanessa Terán-Cerqueda, Lilia Adela García-Stivalet, José Alejandro Limón-Flores, Erick Armenta-Castro, Aurora Rodríguez-Martínez, Bertha Alicia Leon-Chavez, Verónica Vallejo-Ruiz, Duane C. Hassane, Sonia Mayra Pérez-Tapia, Vianney Ortiz-Navarrete, Monica L. Guzman, Rosana Pelayo

**Affiliations:** ^1^ Laboratorio de Oncoinmunología y Citómica, Centro de Investigación Biomédica de Oriente, Instituto Mexicano del Seguro Social Delegación Puebla, Puebla, Mexico; ^2^ Departamento de Biomedicina Molecular, Centro de Investigación y de Estudios Avanzados (CINVESTAV), Mexico City, Mexico; ^3^ Computer Science and Artificial Intelligence Lab, Massachusetts Institute of Technology (MIT), Cambridge, MA, United States; ^4^ The Broad Institute of MIT and Harvard, Cambridge, MA, United States; ^5^ Hospital para el Niño de Toluca, Instituto Materno Infantil del Estado de México (IMIEM), Toluca, Mexico; ^6^ Servicio de Hematología, Unidad Médica de Alta Especialidad, Hospital de Especialidades “Manuel Ávila Camacho”, Instituto Mexicano del Seguro Social, Puebla, Mexico; ^7^ Posgrado en Ciencias Químicas, Area de Bioquímica y Biología Molecular, Benemérita Universidad Autónoma de Puebla, Puebla, Mexico; ^8^ Division of Hematology and Medical Oncology, Department of Medicine, Weill Cornell Medicine, New York, NY, United States; ^9^ Unidad de Desarrollo e Investigación en Bioprocesos (UDIBI) and Unidad de Investigación, Desarrollo e Innovación Médica y Biotecnológica (UDIMEB), Escuela Nacional de Ciencias Biológicas, Instituto Politécnico Nacional, Mexico City, Mexico

**Keywords:** acute lymphoblastic leukemia, leukemia-initiating cell, bone marrow niche, mesenchymal stromal cells, tumor microenvironment, B-cell development

## Abstract

B-cell acute lymphoblastic leukemia (B-ALL) results from the expansion of malignant lymphoid precursors within the bone marrow (BM), where hematopoietic niches and microenvironmental signals provide leukemia-initiating cells (LICs) the conditions to survive, proliferate, initiate disease, and relapse. Normal and malignant lymphopoiesis are highly dependent on the BM microenvironment, particularly on CXCL12-abundant Reticular (CAR) cells, which provide a niche for maintenance of primitive cells. During B-ALL, leukemic cells hijack BM niches, creating a proinflammatory milieu incompetent to support normal hematopoiesis but favoring leukemic proliferation. Although the lack of a phenotypic stem cell hierarchy is apparent in B-ALL, LICs are a rare and quiescent population potentially responsible for chemoresistance and relapse. Here, we developed novel patient-derived leukemia spheroids (PDLS), an *ex vivo* avatar model, from mesenchymal stromal cells (MSCs) and primary B-ALL cells, to mimic specialized niche structures and cell-to-cell intercommunication promoting normal and malignant hematopoiesis in pediatric B-ALL. 3D MSC spheroids can recapitulate CAR niche-like hypoxic structures that produce high levels of CXCL10 and CXCL11. We found that PDLS were preferentially enriched with leukemia cells displaying functional properties of LICs, such as quiescence, low reactive oxygen species, drug resistance, high engraftment in immunodeficient mice, and long-term leukemogenesis. Moreover, the combination of PDLS and patient-derived xenografts confirmed a microenvironment-driven hierarchy in their leukemic potential. Importantly, transcriptional profiles of MSC derived from primary patient samples revealed two unique signatures (1), a *CXCL12^low^ inflammatory and leukemia expansion* (ILE)-like niche, that likely supports leukemic burden, and (2) a *CXCL11^hi^ immune-suppressive and leukemia-initiating cell* (SLIC)-like niche, where LICs are likely sustained. Interestingly, the CXCL11^+^ hypoxic zones were recapitulated within the PDLS that are capable of supporting LIC functions. Taken together, we have implemented a novel PDLS system that enriches and supports leukemia cells with stem cell features driven by CXCL11^+^ MSCs within hypoxic microenvironments capable of recapitulating key features, such as tumor reemergence after exposure to chemotherapy and tumor initiation. This system represents a unique opportunity for designing *ex vivo* personalized avatars for B-ALL patients to evaluate their own LIC pathobiology and drug sensitivity in the context of the tumor microenvironment.

## Introduction

Childhood cancer, a global health priority, remains a leading cause of death from disease in scholar age, with B-cell acute lymphoblastic leukemia (B-ALL) exhibiting substantial number of years of life lost and increasing rates of unfavorable outcome cases in low- to middle-income countries (1, 2).

B-ALL starts and progresses in the bone marrow (BM), where malignant precursor cells expand in the context of pro-inflammatory microenvironments and a highly complex and dynamic BM topology, endowed with the ability of selecting pre-malignant clones able to evolve into tumor (3–6). A number of genetic abnormalities associated with high-risk B-ALL suggest stem cell-like properties, such as colonization of hematopoietic niches, and highlight the cooperation between leukemia cells and BM microenvironment by intrinsic and extrinsic signals. Furthermore, the evaluation of the hematopoietic organization structure in B-ALL has challenged the traditional hierarchy of the differentiation, revealing that cell fate decisions are indeed supported by heterogeneous hematopoietic stem/progenitor cell (HSPC) niches in a more stochastic structure. A new model of hematopoietic forming units suggests that HSPC can respond to environmental cues driving intra- and inter-communication networks that may create adaptable niches (7, 8).

Accordingly, CXCL12-abundant reticular (CAR) niches, formed by specialized mesenchymal stromal cells (MSCs) (9) overlapping with nestin and leptin receptor (LepR) expression (10, 11) and producing high levels of CXCL12, SCF, and IL-7, are critical for B-cell lymphopoiesis (12). The essential roles of the CXCL12/CXCR4 axis in niche positioning and cell cycle status of leukemia stem cells have been highlighted by the specific deletion of CXCL12 from BM MSCs, suggesting the differential use of CXCL12-niches by CXCR4^+^ malignant cells (13). The professional cytokine-secreting CAR cells that create stage-specific micro-niche configurations crucial for maintenance, cell cycling, and differentiation fate decisions of lymphoid, and myeloid progenitors, have been recently defined by transcriptomics single-cell approaches (14, 15), and confirmed the critical interdependence of normal and malignant HSPC with their niches (16). Although the lack of a phenotypic stem cell hierarchy is apparent in B-ALL, leukemia-initiating cells (LICs) have been recognized as a rare subpopulation endowed with stemness properties and potentially responsible for chemoresistance and relapse (17, 18). Therefore, due to their clinical and therapeutical implications, it is critical to characterize the relationship between LICs and their microenvironment. Computational modeling approaches have recently inferred a unique inflammation-inducible CXCR7^+^ B-precursor cell population, displaying abnormal phenotypes and presumably able to colonize distinct emergent inflammatory niches producing CXCL11 (19). Moreover, three-dimensional (3D) hematopoietic structures have been instrumental to advance our knowledge on cell-to-cell intercommunication, nutrient diffusion, oxygen gradients, hypoxic zone formation, and HSPC expansion (20, 21).

Thus, to better investigate the LICs in their microenvironment, we sought to implement a co-culture method capable of mimicking the BM niche and sustain primary B-ALL cell growth and survival from B-ALL patients. The resulting patient-derived leukemic spheroids (PDLS) showed a remarkable ability to enrich leukemia cells with stem cell properties. RNA-seq data from pediatric B-ALL-derived MSCs provided evidence of two putative MSCs subpopulations with unique and distinguishable immunological expression profiles and potential clinical implications (1): a *pro-inflammatory and leukemia expansion* (ILE)-like niche, and (2) an *immune-suppressive and leukemia-initiating cell* (SLIC)-like niche. Strikingly, PDLS recapitulated the hypoxic CXCL11^+^ zones that support LICs, revealing the previously undescribed relevance of CXCL11^+^ mesenchymal niches for cell maintenance of long-term leukemia initiating and relapse population.

## Methods

### Patient Characteristics and Sample Collection

This research has been performed in accordance with the Declaration of Helsinki and was approved by the Ethics, Research and Biosafety Committee from IMIEM (CIEICE-007-01-13) and by the National Committee of Scientific Research at IMSS (R-2012-3602-29 and R-2015-785-120). All samples were collected after informed consent from parents. The study included 147 B-ALL pediatric patients, 8 months to 16 years old (8.15 ± 4.47), referred to the IMSS Specialties Hospital and the IMIEM Children’s Hospital. At clinical diagnosis, 85% of patients were classified as high risk and 41.5% as ProB/PreB-, 30.6% as ProB-, and 27.9% as PreB-ALL, with only 30% exhibiting prognostic translocations. Control BM was obtained from 12 healthy children undergoing minor orthopedic surgery. BM specimens were collected by aspiration before any treatment and according to international and institutional guidelines. (**Supplementary Table S1**).

### Isolation of Primitive Hematopoietic Cells

Mononuclear cells (MNCs) were separated by Ficoll-Paque Plus (GE Healthcare Bioscience, NJ, USA) gradient. No sample pooling was performed for any of the experimental strategies. By immunophenotyping with fluorochrome-conjugated antibodies (**Supplementary Table S2**), Pro-B cells were identified as CD45^low/-^CD34^+^CD10^+^CD19^+^ and Pre-B as CD45^low/-^CD34^-^CD10^+^CD19^+^, before sorting in a FACSAria II flow cytometer (BD Biosciences, USA) (**Supplementary Figures S1A, B**).

### Cell Lines

REH and RS4;11 B-ALL cell lines were purchased from ATCC (VA, USA) and cultured according to instructions. Nalm6 cell line was kindly provided by Dr. JL Maravillas (INNSZ, Mexico). Cell lines were tested for mycoplasma and authenticated using STR assays.

### Primary Mesenchymal Stromal Cells

MSCs were isolated by adhesion, as previously reported (22).

### Patient-Derived Leukemic Spheroids

A total of 25,000 MSCs were plated on 96-well round-bottom plates previously coated with 1% agarose to induce spheroid formation for 24 h, before co-culture with leukemic cells (22). For harvesting, PDLS were incubated with 0.05 mM PBS-EDTA for 5 min to detach cells from the surface, followed by 10 min enzymatic treatment (TrypLE Express, Gibco, CA, USA) and mechanical disruption. Cell suspension was recovered from the inside of PDLS (PDLS-in) and separated from outer cells and supernatant (PDLS-out), before staining with fluorochrome-conjugated antibodies and/or direct FACS analysis (**Supplementary Figures S1C, D**).

### Cell Tracking Strategies

MSCs or B-ALL cells were stained with fluorescent dyes Cell Trace Violet^®^, Cell Trace CFSE^®^, or Cell Trace Far Red^®^ (Invitrogen, Life Technologies, CA, USA), according to the manufacturer.

### Fluorescence Microscopy

PDLS were fixed with 4% PFA and 2 h treated with 0.01% Triton X-100 (Bio-Rad, MX). Upon 1 h blocking with 3% BSA, they were incubated overnight at 4°C with primary unlabeled antibodies in PBS 3% SFB, washed, and incubated for 1 h with conjugated secondary antibody before 10 min DAPI staining and Vectashield. BM biopsy staining was performed as described (22).

### Cytokine Detection

Supernatants were collected after 24 h of 3D culture and investigated for cytokines by multiplex assays (Milliplex Map, Millipore, Merck MX).

### Proliferation Assay

FACS-sorted B-ALL cells were stained with CellTrace CFSE^®^ (Invitrogen, Life Technologies, CA, USA), co-cultured with MSC spheroids, and further assayed for fluorescent dye dilution by flow cytometry.

### Pimonidazole Incorporation and Hypoxia Detection

Hypoxia was detected by the Hypoxyprobe-1 Plus Kit (Pimonidazole Hydrochloride, Chemicon International, Temecula, CA, USA). Pimonidazole incorporation was confirmed by flow cytometry and fluorescence microscopy. Image-IT green hypoxia (Invitrogen, Life Technologies, CA, USA) was used to track low oxygen levels.

### Side Population Assay

Harvested cells were adjusted to 10^6^ cells/ml and incubated with Hoechst 33342 to a final concentration of 5 μg/ml (Sigma-Aldrich, MX), 37°C for 2 h, prior to staining with anti-human CD45.

### Patient-Derived Xenografts

*In vivo* experiments were conducted according to the WCM Institutional Animal Care and Use Committee (IACUC) and the CINVESTAV Committee for Animal Care and Use (CICUAL) guidelines and regulations. NOD/SCID gamma chain (NSG) mice from the Jackson Laboratory (JAX, CA, USA) were i.v. injected with primary B-ALL cells from 48 h cultures. Animals were euthanized after 5 weeks or when exhibiting clinical signs of leukemic disease. Human CD45^+^ cell frequencies in peripheral blood and BM were investigated for engraftment monitoring.

### Limiting Dilution Assays

Serial dilutions of leukemic cells were injected into NSG mice. After 4 weeks, the engraftment was determined by flow cytometry and documented as positive when human CD45^+^ cells recorded within mouse BM cells were >1%. ELDA program was used to calculate the LIC content for each culture condition (23).

### RNA-Sequencing Library Preparation and Analysis

Whole RNA was extracted from 5 × 10^5^ MSC (RNeasy kit, QIAGEN, MX), and samples with RIN > 8 were used for experiments. Libraries were constructed by using the TruSeq Stranded mRNA Library Prep Kit (Illumina, CA, USA) before mRNA sequencing on a NextSeq 500 instrument at INMEGEN (Mexico). Paired-end reads were aligned to the human genome reference GRCh38/hg38 (build 38.2) with the R software package Rsubread (24) and read mapping statistics were reported (**Supplementary Table S3**). Mapped reads were summarized to gene level counts featured by counts function of Rsubread, considering the built-in NCBI RefSeq gene annotation for gene reference. Protein coding genes with detected counts in at least one sample library were retained and normalized using TMM normalization. Differential expression analysis was performed with the edgeR package (25). Statistical analyses and plots were performed using the programming language R (R Core Team, 2012). Gene ontology and functional enrichment analyses were performed by Metascape (26). The original contributions presented in the study are publicly available. RNA-seq data can be found in E-MTAB-10838 (https://www.ebi.ac.uk/arrayexpress/experiments/E-MTAB-10838/).

### Data Analysis and Statistics

FlowJo 10.0.8 (TreeStar Inc., Ashland, OR, USA) and Infinicyt 1.8 (Cytognos, Spain) software were used for cytometry data, while Prism 8 (GraphPad, CA, USA) software was used for statistical analysis. Differences within groups were established by non-parametric tests, considering significant probability values <0.05. Mann–Whitney *U* test with α of 5% to define significance was applied. Data were normally distributed and individual data points for independently repeated experiments and mean (SD) were graphed.

## Results

### Mesenchymal 3D Spheroids Are Capable of Reconstructing Unique B-ALL Niches

**Figure 1:**
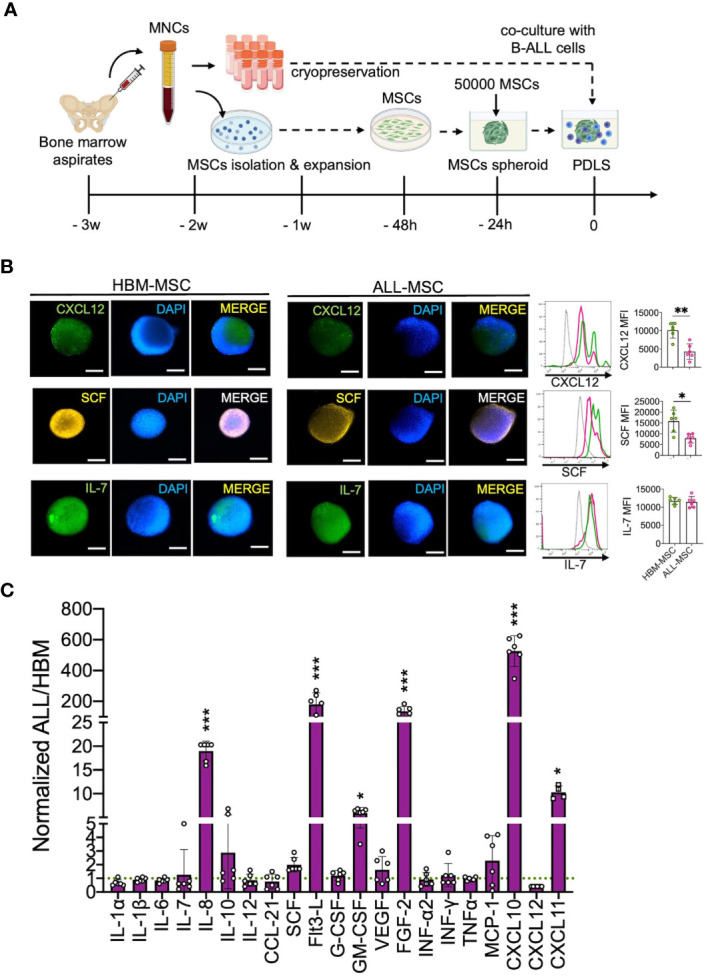
B-ALL BM MSC form 3D spheroids endowed with high CXCL10 and CXCL11 production. **(A)** Mononuclear cells (MNCs) were obtained from healthy BM (HBM) donors or B-ALL patients, and MSCs were isolated by their adherent properties and were cryopreserved to be used in further experiments. **(B)** A total of 50,000 MSCs from HBM or B-ALL were induced to form a stromal multicellular spheroid, and CAR-cell derived factors (CXCL12, SCF, and IL7) were evaluated by immunostaining and FACS (*n* = 6). **(C)** Growth factors, cytokines, and chemokine production from B-ALL-MSC spheroids (3D) were evaluated after collection of 24-h supernatants and normalized to HBM-MSC spheroids (*n* = 6). MSC, mesenchymal stromal cell; HBM, healthy bone marrow; B-ALL, B-cell acute lymphoblastic leukemia; CAR-cell, CXCL12-abundant reticular cell; FACS, Fluorescence-activated cell sorting. *P < 0.05; **P < 0.01; ***P < 0.001. Error bars represent SD.

As 3D cellular organization is essential to preserve physiological features of BM, we generated 3D structures to characterize the leukemic niche by using MSCs derived from either primary B-ALL at disease onset (ALL-MSC) or from healthy bone marrow donors (HBM-MSC) (**Figure 1A**). We found that MSCs were capable of forming a single multicellular spheroid within the first 24 h of non-adherent culture conditions with a direct cell number–size relationship (**Supplementary Figures S2A, B**). Despite the decrease in the MSC proliferation in 3D settings, the classical MSC markers were conserved (**Supplementary Figures S2C, D**). CXCL12-abundant reticular (CAR) immunophenotype was confirmed in CXCL12^hi^SCF^hi^IL-7^hi^ HBM-MSC spheroids (**Figure 1B**) as well as the expression of Nestin, PDGFRα, and LepR (**Supplementary Figure S2E**). In contrast, a substantially lower abundance of typical CXCL12^hi^SCF^hi^ CAR cells in ALL-MSC spheroids with weaker expression of CXCL12 and SCF (**Figure 1B**), but increased production of IL-8, Flt3-L, GM-CSF, FGF-2, CXCL10, and CXCL11, was observed in the supernatants evaluated at 24 h (**Figure 1C**). ALL-MSCs in a 3D organization have the ability to *in vitro* recapitulate unique CAR niche-like structures that produce high levels of CXCL10 and CXCL11.

### Primary B-ALL Cells Can Be Expanded in Mesenchymal 3D Spheroids

**Figure 2:**
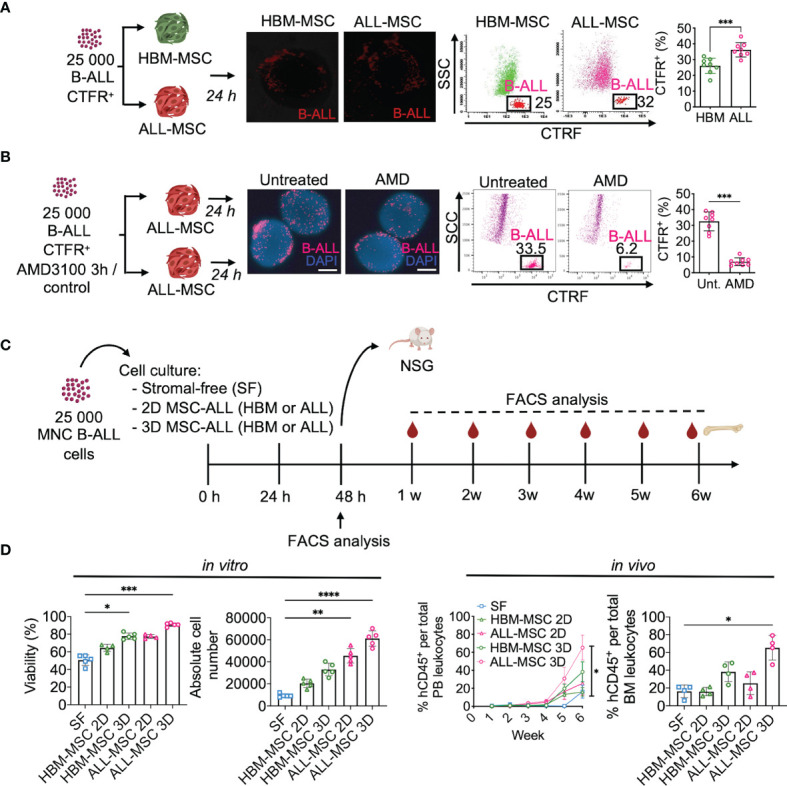
Mesenchymal stromal spheroids support primary leukemogenic B-ALL cells. **(A)** Primary sorted B-ALL CD10^+^CD19^+^ blasts were labeled with CTFR and co-cultured with HBM-MSC or ALL-MSC spheroids. After 24 h, spheroids were washed and analyzed by fluorescence microscopy and FACS. CTFR^+^ colonizer cell frequencies determined upon enzymatic digestion (*n* = 7). **(B)** CTFR-labeled primary B-ALL cells were treated with a CXCR4 inhibitor (AMD3100) 5 mM for 3 h and then co-cultured within PDLS. Upon 24 h, CTFR^+^ content was determined by FACS (*n* = 8). **(C)** Schematic representation of experimental design, 25,000 mononuclear cells from B-ALL patients were cultured in SF conditions, or co-cultured with HBM- or B-ALL-MSC monolayer (2D) or spheroids (3D). **(D)** Cell viability was analyzed by flow cytometry upon 48 h and absolute cell number was determined (*n* = 5) (left). Leukemic cells from independent experiments were harvested at 48 h of culture and transplanted into NSG mice. Human engraftment of hCD45^+^ was weekly monitored in PB by FACS and engraftment in BM was determined 6 weeks after transplantation (*n* = 4) (right). MSC, mesenchymal stromal cell; HBM, healthy bone marrow; B-ALL, B-cell acute lymphoblastic leukemia; CTFR, Cell Trace Far Red; FACS, Fluorescence-activated cell sorting; SF, stromal-free; PB, peripheral blood. *P < 0.05; **P < 0.01; ***P < 0.001, ***P < 0.0001. Error bars represent SD.

Since we confirmed the ability of the 3D ALL-MSCs spheroids of recapitulate CAR niche-like structures, we sought to evaluate the ability of primary B-ALL cells to migrate to the MSC spheroids by assessing their colonization capacity. First, we stablished the 3D HBM- or ALL-MSCs spheroids, and 24 h later, we seeded 25,000 primary B-ALL cells CD10^+^CD19^+^ (*n* = 8), labeled with Cell Trace Far Red. After 24 h of co-culture, spheroids were washed and prepared for the whole-mount fluorescence microscopy analysis or enzymatically disrupted to analyze their cellular content by multiparameter flow cytometry (MPFC). We found a clear advantage for ALL-MSC spheroids to facilitate the colonization of leukemic cells when compared with the HBM-MSC (**Figure 2A**). To evaluate niche saturation, serial spheroid sizes were tested, finding that in all cases, only near 1%–3% of leukemic cells were able to colonize inner niches (**Supplementary Figure S3A**). Since CXCR4 has been implicated in homing of leukemia cells to their niche (27, 28), we tested the effect of plerixafor (AMD3100) in the colonization of AMD3100-treated B-ALL cells to the 3D structures and found that it can only partially prevent B-ALL cell spheroid colonizing, with a 4.6-fold decrease (**Figure 2B**). The effect was similar when the niche positioning of leukemic cells into normal BM spheroids was investigated (**Supplementary Figure S3B**).

Next, we assessed the ability of the spheroids to maintain cells capable of initiating leukemia without enrichment. Thus, we co-cultured 25,000 MNCs from five different B-ALL patients with either stromal-free (SF), MSCs monolayers (2D), or spheroids (3D) for 48 h and then transplanted into NSG mice (**Figure 2C**). We observed that 3D architecture was best at facilitating survival and expansion of primary leukemia cells when compared to other culture conditions (**Figure 2D** and **Supplementary Figure S3C**). Importantly, 3D co-cultures in ALL-MSC expanded more robustly (**Figure 2D**) and exhibited higher leukemic engraftment at week 6 post-transplantation than other culture conditions (**Figure 2D** and **Supplementary Figure S3D**). In addition, cells cultured in the 3D system performed better than freshly thawed MNC and transplanted (**Supplementary Figure S3E**). Taken together, we demonstrated that ALL-MSC spheroids support homing, survival, growth, and efficient engraftment of primary B-ALL cells. This co-culture system is referred to as patient-derived leukemic spheroids (PDLS).

### Hypoxic Patient-Derived Leukemic Spheroids Support Leukemia Cells With Stem Cell Features

**Figure 3:**
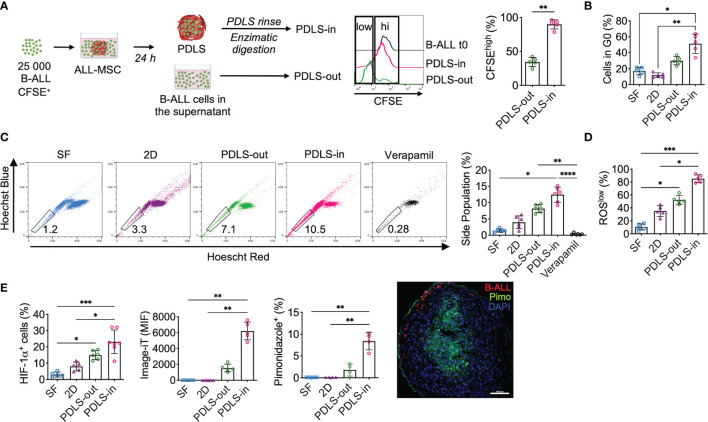
PDLS promote stem cell-like phenotype, quiescence, and hypoxia in a subset of primary B-ALL cells. **(A)** Primary sorted B-ALL CD10^+^CD19^+^ blasts were labeled with CFSE and co-cultured with ALL-MSC to form PDLS. At 24 h, frequency of CFSE^hi^ was determined by FACS in the supernatants after spheroid removal (PDLS-out) and in the PDLS colonizer cells (PDLS-in) after several washes and enzymatic digestion (*n* = 5). **(B)** Primary B-ALL blasts were cultured in stromal-free (SF) conditions and co-cultured with ALL-MSC in monolayer (2D) and PDLS settings for 48 h and cell cycle status was evaluated by Ki-67 staining and DNA content by FACS (*n* = 5). **(C)** Side population cell contents are shown (*n* = 5). **(D)** ROS production was measured by FACS and ROSlow frequency was recorded (*n* = 5). **(E)** Hypoxia was investigated by HIF-1α expression (left), image-iT fluorescent hypoxia probe (middle) and pimonidazole incorporation (right) by FACS. Fluorescence microscopy of pimonidazole incorporation of PDLS is shown (*n* = 7). MSC, mesenchymal stromal cell; B-ALL, B-cell acute lymphoblastic leukemia; PDLS, patient-derived leukemia spheroids; CFSE, carboxifluorescein; FACS, fluorescence-activated cell sorting. *P < 0.05; **P < 0.01; ***P < 0.001, ***P < 0.0001. Error bars represent SD.

Despite the fact that LICs in B-ALL have been controversial due to the lack of a specific immunophenotype (18), cells with stem cell features have been shown to be enriched in hypoxic zones within the BM (29). Here, we sought to characterize the cells capable of colonizing the PDLS. Because leukemia initiation in NSG mice is a feature of LICs, this and additional LIC properties were evaluated in different compartments of 3D structures using primary B-ALL samples. At 24 h, we harvested cells from the supernatant (PDLS-out) and, upon enzymatic digestion of the PDLS structure, collected the cells that migrated into the inner spheroid (PDLS-in). More than 90% of spheroid-colonizing B-ALL cells (PDLS-in) showed low proliferation activity when growing inside PDLS, while PDLS-out cells exhibited higher proliferation (**Figure 3A**). Consistently, a quiescent (G0) profile defined the PDLS-in cells (**Figure 3B**), which is a feature of LICs (18). Furthermore, we investigated stem cell features such as “side population” (**Figure 3C** and **Supplementary Figure S4A**) and low ROS production (30) (**Figure 3D**), confirming that PDLS-in cells also displayed such properties when compared with other culture scenarios. Moreover, an increase in HIF-1α expression was recorded (**Figure 3E** and **Supplementary Figure S4B**), consistent with increased hypoxia in the PDLS-in cells, assessed by the image-iT green hypoxia tracker and pimonidazole incorporation. These data confirmed a PDLS-in hypoxic setting for both MSCs and B-ALL (**Figure 3E** and **Supplementary Figure S4C**). Taken together, PDLS provide strong evidence that stem-like B-ALL can be enriched by their function and biological features within hypoxic niches, suggesting that they may be the foundation of leukemia-migrating and -proliferating cells.

### PDLS Foster Cells With the Capacity of Leukemia Initiation and Chemoresistance

**Figure 4:**
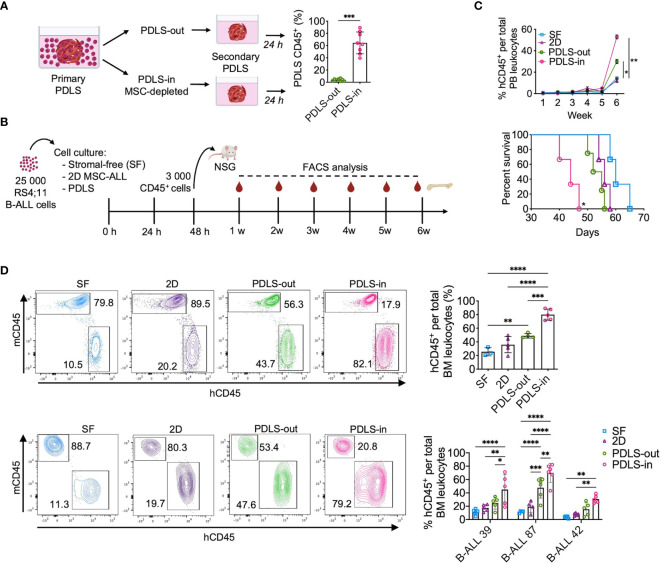
Leukemia-initiating cells (LICs) are enriched in PDLS internal niches. **(A)** Secondary spheroid colonization assay was performed with purified PDLS-in and PDLS-out leukemic cells from primary PDLS (*n* = 8). **(B)** Schematic representation of experimental design, Leukemic cells were cultured during 48 h on stromal-free (SF), MSC monolayers (2D), and PDLS and 3,000 CD45^+^ cells were transplanted into NSG mice. **(C)** Leukemia burden was monitored in peripheral blood by FACS (upper panel) and overall survival was plotted (lower panel) when RS4:11 cells were used (*n* = 5). **(D)** Engraftment was determined in BM after 6 weeks of xenotransplantation of RS4:11 cells (upper panel) or three different primary B-ALL cells (representative plots are shown in lower panel) (*n* = 5). B-ALL, B-cell acute lymphoblastic leukemia; PDLS, patient-derived leukemia spheroids; MSCs, mesenchymal stromal cells; FACS, Fluorescence-activated cell sorting. *P < 0.05; **P < 0.01; ***P < 0.001, ***P < 0.0001. Error bars represent SD.

As PDLS were colonized by leukemia cells with stem cell features, we sought to determine whether cells isolated from PDLS-in are characterized by the increased ability of homing. By serial spheroid seeding assay, we discovered that PDLS-in were capable of re-colonizing spheroids with higher efficiency than PDLS-out cells (**Figure 4A**), highlighting their homing and stem cell potentials. To further characterize the LICs capacity, 3,000 sorted CD45^+^ RS4;11 cells from 48 h PDLS-in and other culture scenarios were used to inject NSG mice. Leukemia burden was weekly monitored, and final engraftment was evaluated at 6 weeks (**Figure 4B**). Mice transplanted with purified PDLS-in cells showed the highest numbers of human CD45^+^ cells peripheral blood (PB) and exhibited the lowest overall survival (OS) of 44 days (**Figure 4C**). BM analysis confirmed the facilitated engraftment with PDLS-in RS4;11 cells. Such results were validated with three different primary human samples from ProB and PreB pediatric-ALL patients (**Figure 4D**). Limiting dilution assay revealed that LICs frequency was 10 times less in stroma-free settings when compared to PDLS-in conditions (**Supplementary Figures S5A, B**). Remarkably, a LICs enrichment was observed in PDLS-in (1/45.2), compared with PDLS-out (1/858) and SF conditions (1/704) (**Supplementary Figure S5C**). MPFC analysis of PDLS-in confirmed that LICs enrichment by PDLS was likely driven by functional attributes associated with leukemia stemness rather than by immunophenotype (**Supplementary Figure S5D**), supporting the notion of a functional LICs hierarchy driven by specialized microenvironmental cues. Thus, PDLS could be potentially used as a proxy to determine the presence of LICs in pediatric B-ALL patient samples.

**Figure 5:**
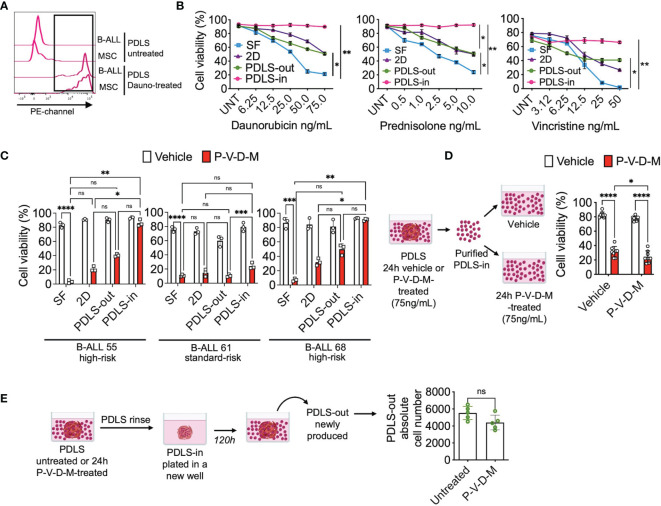
LICs are protected from chemotherapy within PDLS. **(A)** B-ALL PDLS were incubated with daunorubicin (75 ng/ml) for 1 h and enzymatically disrupted; red fluorescence was determined in CD45^+^ (B-ALL) and MSCs by FACS (*n* = 5). **(B)** Primary B-ALL blasts were cultured in SF, 2D and PDLS and CD45^+^ cell viability evaluated upon 24-h treatment with daunorubicin, prednisolone, and vincristine (*n* = 3), or **(C)** combined chemotherapy: daunorubicin [75 ng/ml], prednisolone [10 ng/ml], vincristine [50 ng/ml], and methotrexate [5 mM] (P-V-D-M). **(D)** Viable leukemic cells recovered from PDLS-in untreated or 24-h P-V-D-M-treated were re-exposed to the drugs for 24 h and their viability was measured by FACS (*n* = 5). **(E)** PDLS P-V-D-M-treated were replated; upon 120 h, PDLS-out leukemia re-emerging was recorded (*n* = 7). B-ALL, B-cell acute lymphoblastic leukemia; PDLS, patient-derived leukemia spheroids; MSCs, mesenchymal stromal cells; FACS, Fluorescence-activated cell sorting; NS, non-significant. *P < 0.05; **P < 0.01; ***P < 0.001, ***P < 0.0001. Error bars represent SD.

As LICs have also been described as chemo-resistant (13, 18, 27, 31), we proceeded to investigate the response of the PDLS-in cells to the most commonly used chemotherapy drugs for B-ALL treatment. To this end, the ability of drugs to diffuse inside the spheroid was investigated. When treating PDLS with the anthracycline daunorubicin, the cells were able to uptake daunorubicin within the first hour, evidenced by their red fluorescence (**Figure 5A**). By examining the viability at 24 h of treatment, we found that daunorubicin, prednisolone, and vincristine, even at high concentrations, were not effective in killing the PDLS-in cells (**Figure 5B**). Of note, combined chemotherapy commonly used in B-ALL, including daunorubicin, prednisolone, vincristine, and methotrexate (P-V-D-M), displayed similar results when investigated in high-risk (HR) and standard-risk (SR) patients (**Figure 5C**). Furthermore, when PDLS-in vehicle or P-V-D-M-treated cells were purified and exposed for an additional 24 h in stroma-free conditions, cells remained chemo-resistant (**Figure 5D**). Next, to determine the potential of PDLS-in cells to recapitulate disease after chemotherapy, PDLS were treated with combined chemotherapy for 24 h, washed to remove PDLS-out cells, and cultured again in fresh wells. Strikingly, newly formed PDLS-out cells were harvested upon 120 h and no differences were observed when compared to untreated PDLS (**Figure 5E**), suggesting that PDLS can capture clinical features, such as tumor reemergence after cell survival within internal niches during chemotherapy.

### Gene Expression Signatures for BM MSCs Reveals Pro-Inflammatory and Suppressor Niches in B-ALL Patients

**Figure 6:**
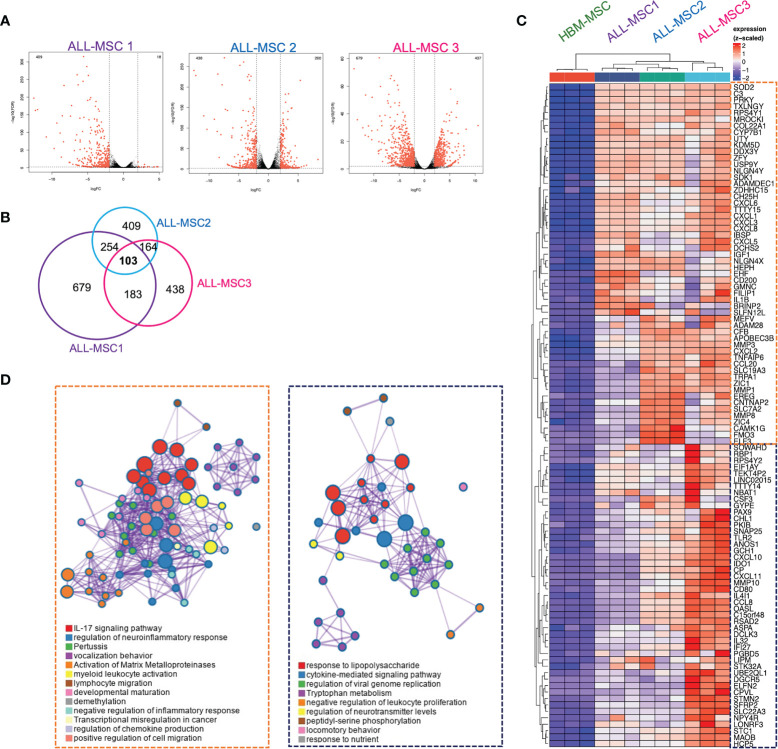
Differentially expressed genes in MSCs derived from B-ALL pediatric patients: RNA-seq approach. **(A)** Volcano plots of statistically significant differentially expressed genes from three B-ALL patients compared to normal counterpart from a healthy donor is shown (*n* = 3). **(B)** Venn diagram on intersection analysis of overexpressed genes found in **(A)**. **(C)** Heatmap of genes consistently overexpressed after analysis of intersections (fold change > 2 and FDR < 0.05). **(D)** Gene ontology and functional enrichment analysis is shown for two clusters. Dataset E-MTAB-10838.

In order to investigate the identity of MSCs isolated from primary pediatric-ALL patients, we performed RNA sequencing analysis of three different ALL-MSCs specimens and HBM-MSC. Substantial and heterogeneous dysregulation of gene expression was found when compared with HBM-MSC (**Figure 6A** and **Supplementary Table S3**). Specifically, 103 genes were consistently overexpressed among ALL-MSC (fold change > 2 and FDR < 0.05) (**Figures 6B, C** and **Supplementary Figures S6A, B**) and, of high interest, two major gene ontology (GO) signatures were identified. A pro-inflammatory signature was characterized by a large set of chemokines involved in neutrophil recruitment, IL-17 signaling, metalloproteinase functional activation, and leukocyte migration including *CXCL1, CXCL2, CXCL3, CXCL5, CXCL6, CXCL8, CCL20*, and pro-inflammatory molecules like *IL1B, IGF1, MMP1, MMP3*, and *MMP8* (**Figure 6D**). An additional signature, predominantly displayed by ALL-MSC3, showed a TLR signaling, cytokine-mediated signaling, and a negative regulation of leukocyte proliferation signatures. Moreover, high expression of chemokines CXCL10 and CXCL11 and a substantial expression of suppressor molecules like indoleamine 2,3-dioxygenase (*IDO1*) and galectin 9 (*LGALS9*) (**Figure 6D**) were apparent. Importantly, ALL-MSC did not exhibit transcriptional differences in the typical MSCs markers CD73, CD90, and CD105 (**Supplementary Figure S6C**), but a very low transcriptional expression of CAR-niche associated genes *CXCL12* and *SCF* were found in CXCL10^+^CXCL11^+^ ALL-MSC3 (**Supplementary Figure S6D**). When downregulated genes were analyzed, we did not find apparent intersections among samples. However, GO analysis at the individual level showed that some extracellular matrix-associated proteins and cell division-associated networks were dramatically altered in similar extent for all three B-ALL MSCs (**Supplementary Figure S6A**). Taken together, the MSC gene expression profiling suggests two potential niches, according to their functional elements within the B-ALL BM microenvironment. A *pro-inflammatory and leukemia expansion* (ILE) niche, where leukemic clones may proliferate and increase tumor burden in the context of an activating pro-inflammatory milieu, and an *immune-suppressive and leukemia-initiating cell* (SLIC) niche, endowed with immunoregulatory and suppressive properties and high transcription of *CXCL10* and *CXCL11*.

### A Hypoxic CXCL11^hi^ Mesenchymal Niche Can Be Recapitulated in the PDLS

**Figure 7:**
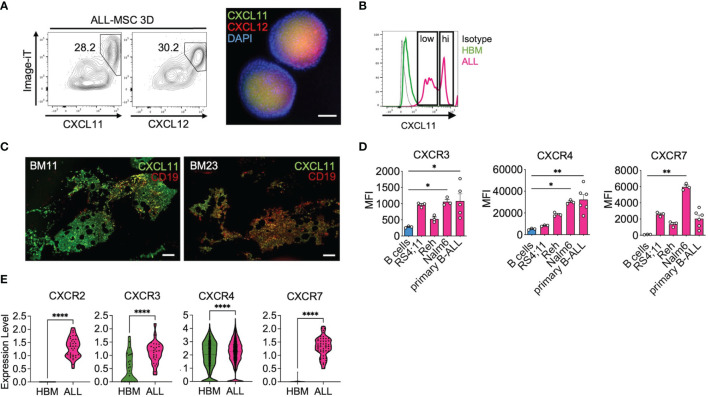
CXCL11 characterize the MSC niche in B-ALL. **(A)** CXCL11 and CXCL12 was hypoxia-tracked by using the image-iT probe and analyzed by FACS and representative CXCL11 and CXCL12 immunofluorescence staining in ALL-MSC spheroids (*n* = 3). **(B)** CXCL11 was determined by FACS in HBM-MSC and ALL-MSC spheroids (nHBM-MSC = 3, nALL-MSC = 9). **(C)** CXCL11 and CD19 immunostaining in BM biopsies. **(D)** CXCR3, CXCR4, and CXCR7 expression analyzed by FACS in B-ALL cell lines (*n* = 3) and primary B-ALL cells (*n* = 6). **(E)** Expression of CXCR2, CXCR3, CXCR4, and CXCR7 in B-ALL and Healthy BM CD19+CD79+ populations obtained from database GSE132509 analyses. MSC, mesenchymal stromal cell; HBM, healthy bone marrow; B-ALL, B-cell acute lymphoblastic leukemia; CAR, CXCL12-derived abundant reticular; FACS, Fluorescence-activated cell sorting. *P < 0.05; **P < 0.01; ****P < 0.0001. Error bars represent SD.

**Figure 8:**
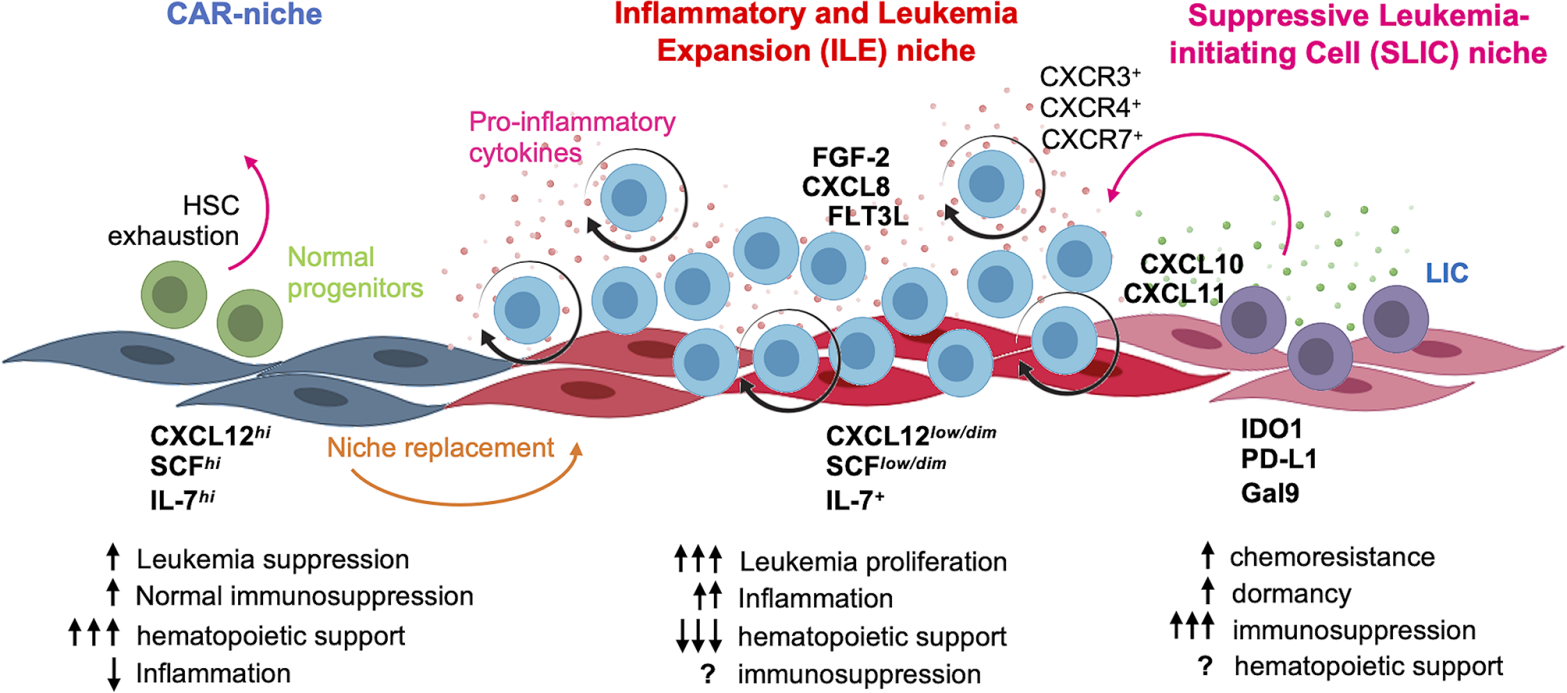
Concluding model. At leukemia debut, B-ALL blasts hijack and remodel CAR niche by inducing CXCL12 and SCF downregulation, concomitant to proinflammatory cytokine production and followed by the emergence of a LIC-supporting niche endowed with suppressor properties. The functional identity of normal CAR niche and the suggested *inflammatory and leukemia expansion* (ILE) and *suppressive leukemia-initiating cell* (SLIC) hematopoietic niches are highlighted. Figure was created using BioRender templates.

In order to assess our transcriptional observations in our PDLS system, we used immunostaining approaches to characterize CXCL11 expression in a leukemia microenvironment. Strikingly, we found that CXCL11^hi^ MSC spheroids were enriched in hypoxic CXCL12^hi^ zones with partial overlapping (**Figure 7A**). Distinct CXCL11^low^ and CXCL11^hi^ cell populations were also evident in ALL-MSC spheroids, while HBM-MSC spheroids did not show CXCL11 expression (**Figure 7B**). Moreover, the occurrence of CXCL11^low/hi^ MSCs in B-ALL BM biopsies was confirmed (**Figure 7C**), where CXCL11 co-stained with CD19. Additionally, B-ALL cells, but not normal CD34^+^ precursor cells, expressed CXCR3 and CXCR7, the receptors for CXCL10, CXCL11, and CXCL12, suggesting their advantage for selective niche colonization (**Figures 7D, E** and **Supplementary Figure S7A**). CXCL10^hi^CXCL11^hi^ zones may represent exclusive leukemia-positioning niches where B-ALL cells may also contribute to CXCL11 expression (**Supplementary Figure S7B**) presumably relevant for positioning of CXCR3^+^CXCR7^+^ LICs and suitable for immune scape (**Figure 8** and **Supplementary Figure S7C**). Taken together, we demonstrate that PDLS are capable of capturing a SLIC niche endowed with a specialized gene expression signature and the selection of malignant cells with stem cell functions.

## Discussion

93.5% of poor prognosis cases of pediatric leukemias are registered in low- to middle-income countries, where 90% of the world’s children live, with relapses occurring at very early stages of treatment and increasing numbers of high-risk fates (1, 2, 32). Such epidemiology highlights the importance of a comprehensive understanding of the origins and coevolution of the disease in the context of micro/macroenvironmental cues. The phenotypical and functional identity of LICs and leukemia-relapsing cells (LRCs) and the niches where they evolve are critical for the construction of a more integrated view of the nature of leukemia subtypes and their potential control (33, 34).

Here, we have addressed key aspects of the microenvironment-related leukemia etiopathogenesis through implementation of PDLS. This *in vitro* avatar model is a powerful tool to recapitulate malignant niche biology in human–human settings that better mimic natural ecosystems (**Figure 8**). We demonstrated that LIC activity is promoted within PDLS niches and facilitated by hypoxic microenvironments. The CXCL12/CXCR4 axis has been considered the most important player in the chemotaxis and retention of hematopoietic cells into their BM niches (9, 35, 36), and in normal settings, MSC spheroids have shown to increase the CXCL12 expression (20, 22) as a result of hypoxia (37) and to promote high cellular connectivity mediated by connexins (38). However, upon leukemia onset, CXCL12 expression is downregulated in MSCs (13, 22, 39, 40). Several studies have suggested that normal HSPCs live in anatomic regions with lower O_2_ levels (29) and it is becoming clear the critical role of the BM hypoxic niches in the low oxidative stress status of quiescent HSPCs that avoid their continuous differentiation and exhaustion. Indeed, pseudohypoxia increases the HSPC engraftment, suggesting that HSPC exhaustion can occur in non-hypoxic conditions (41). Further metabolic studies at single-cell resolution in PDLS may reveal how pseudohypoxia and the hostile hypoxic-inflammatory niches cooperate to preserve LIC functions at the expense of normal hematopoiesis.

One of the crucial challenges when working on primary B-ALL cells is the lack of suitable *in vitro* conditions to maintain or expand them *ex vivo*, to accelerate therapy screening and even target microenvironmental cues (16). Advances in the understanding the microenvironment regulation in B-ALL have been occurring by using mouse models and human co-culture systems to replicate BM niches that support LICs (13, 16). So far, our data strongly suggest that LICs can be enriched in PDLS, by their niche requirements rather than immunophenotypic features, according to the stochastic model proposed for B-ALL (17, 18). LIC gene expression profiles are alike to those from measurable residual disease (MRD) and LRCs, where low metabolic activity and increased cell adhesion are common features (17, 42). Interestingly, when LICs are released from their protective niches, chemoresistance can be reversed as the stem cell characteristics are modified (17). Unfortunately, in B-ALL, the ability of certain niches to induce and support malignant stemness remains unknown. An active competition for the niche may displace normal HSPC, where pro-inflammatory signals provided by leukemic cells or their microenvironment are crucial (14, 22, 27, 28, 39, 43–45). We recently reported the relevance of cortactin-mediated cell migration of B-ALL relapse cells for extramedullary infiltration and intra-niche positioning with high tropism for hypoxic PDLS zones (46). So far, our PDLS model has been only investigated with MSCs and B-ALL cells, but additional niche-associated cells, including those from CNS or gonads, can be further studied at individual or collective levels for their contribution on LICs maintenance.

Two MSC niches with unique and distinguishable expression profiles and potential clinical implications are apparent, and suggest the sequential replacement of normal niches with the *inflammatory and leukemia expansion* (ILE) niche, followed by the emergence of a CXCL10^hi^CXCL11^hi^ *suppressive and leukemia-initiating cell* (SLIC) niche, endowed with suppressive capabilities that might be involved in maintenance of long-term initiating or relapse clones. The classical CD73 pan-MSCs marker was found to be increased in some ALL-MSC (data not shown), which may relate to the suppressor role of adenosine (ADO) metabolism in chemoresistance and Treg and suppressor cell development, suggesting a niche-promoted “education”.

Although there is increasing evidence of the Nestin^+^CXCL12^+^ as one essential BM niche (11), dysregulation of CXCL12 and SCF related to pro-inflammatory microenvironment is a feature of ALL (22, 28, 39, 40); the CXCL10/CXCL11/CXCR3 axis has been implicated in chemotherapy resistance and CNS infiltration in B-ALL (47). CXCL10 and CXCL11 share CXCR3 receptor, while CXCL11 is recognized by CXCR7 with more affinity than CXCL12. Theoretical models have suggested an unexpected role of CXCR7 in leukemogenesis (48) and our finding of a CXCL11^hi^ hypoxic niche highlights this. The newly identified CXCL11^hi^ hypoxic niche may play an important role attracting CXCR3^hi^CXCR7^hi^ leukemic cells even within a CXCL12^low^ scenario. These observations suggest that the remaining CXCL11^hi^ sanctuaries and poor recovery of CXCL12^hi^ niches after treatment are likely to be supportive of relapse or/and poor HSPC engraftment during BM transplantation. In fact, the immunosuppressive landscape associated with such CXCL11^hi^ hypoxic niche supports the notion of a potential transient stage that may function as an attractive therapeutic target as it only occurs in leukemia settings (**Figure 8**). In a very elegant work, Witkowski et al. recently discovered an increased frequency of non-classical monocytes CX3CR1^+^ at diagnosis and relapse (49). Moreover, their elimination improves B-ALL treatment response and survival. Interestingly, we discovered that CX3CL1 is highly produced in the SLIC niche (data not shown). Thus, there is a possibility that non-classical monocytes CX3CR1^+^ are also located in the SLIC niche to cooperate with immunosuppressive/chemoprotective signatures.

Finally, it is well-known that MSCs can protect leukemic cells in the presence of chemotherapeutic agents (13, 16, 31) by several protective mechanisms (50) and now we have shown that LICs enriched by PDLS can be moderately sensitized when they are released form their niche.

Together, our data established, for the first time, an *in vitro* functional 3D hematopoietic-mesenchymal avatar to study human hematopoietic malignancies, which restore important BM mesenchymal niche features with positive impact on primary LICs in pediatric B-ALL. There are great expectations to use this model in precision medicine to predict chemo-resistant leukemic phenotypes, to explore novel therapeutic targets for elimination of LICs in their own niche without affecting normal HSPC or to test abnormal niche elimination strategies that favor niche fitness recovery. PDLS may contribute the comprehensive understanding of mechanisms behind human BM microenvironment alterations, avoiding the use of laboratory animals. Moreover, we have provided strong experimental evidence that supports the idea that LICs are critically dependent on mesenchymal niche interactions and evidenced the existence of a regulatory CXCL11^hi^ MSC niche with a potential role in leukemia initiation. Our new findings contribute directly to understand the pathobiology of childhood leukemias and may be the foundation of niche scoring for Next-Gen patient stratification and design of novel tools for their intervention and prevention.

## Data Availability Statement

The original contributions presented in the study are publicly available. These data can be found here: https://www.ebi.ac.uk/arrayexpress/experiments/E-MTAB-10838.

## Ethics Statement

The studies involving human participants were reviewed and approved by Comité Nacional de Investigación Científica. Written informed consent to participate in this study was provided by the participants’ legal guardian/next of kin. The animal study was reviewed and approved by CICUAL CINVESTAV.

## Author Contributions

JCB, MG, and RP conceived and designed the work, interpreted results, and wrote the manuscript. JCB performed most experiments. JD-V performed RNA-seq analysis and drafted the work. AS-C, VT-C, LG-S, and JL-F provided patient samples, clinical discussion, and approved the final version. GZ-H, EA-C, AR-M, BL-C, and VV-R performed experiments. DH, SP-T, and VO-N provided reagents, critical discussion, and drafted the manuscript. All authors contributed to the article and approved the submitted version.

## Funding

This work was supported by grants from the National Council of Science and Technology (CONACYT) (FOSISSS 2015-1-261848 to RP, PRONAII 302941 to RP, and PRONAII 302941 to SP-T), the Mexican Institute for Social Security (IMSS) (FIS/IMSS/PROT/G13/1229 and FIS/IMSS/PROT/G14/1289 to RP), from “Red de Desarrollo de Fármacos y Métodos de Diagnóstico” and “Red para la Investigación en Células Troncales” from CONACYT (to RP and JCB), and R01CA234478, Irma T. Hirschl/Monique Weill-Caulier Trust, Unravel Cancer Foundation and 1R21CA24545401A1 to MG.

## Conflict of Interest

The authors declare that the research was conducted in the absence of any commercial or financial relationships that could be construed as a potential conflict of interest.

## Publisher’s Note

All claims expressed in this article are solely those of the authors and do not necessarily represent those of their affiliated organizations, or those of the publisher, the editors and the reviewers. Any product that may be evaluated in this article, or claim that may be made by its manufacturer, is not guaranteed or endorsed by the publisher.
