# Supplementary material for: Patient-Derived Bone Marrow Spheroids Reveal Leukemia-Initiating Cells Supported by Mesenchymal Hypoxic Niches in Pediatric B-ALL
Source: Front Immunol. 2021 Oct 19;12:746492. doi: 10.3389/fimmu.2021.746492 (PMC8561951; doi:10.3389/fimmu.2021.746492)
Supplement: Supplementary file 1 [file DataSheet_1.docx]

Supplementary Material

# Supplementary Tables

Supplementary Table 1. B-ALL patients characteristics.

| **#** | **Age (y)** | **Sex** | **Immunophenotype** | **WBC (x103/mm3)** | **BM Blast (%)** | **Chromosomal aberrations** | **Risk** | **Assays** |
| --- | --- | --- | --- | --- | --- | --- | --- | --- |
| 1 | 3 | F | PreB | 293.4 | 94.6 | - | High | MSC isolation, immunophenotype, subernatants, PDOs, |
| 2 | 2 | F | PreB | 7.1 | 67 | - | High | MSC isolation, drug treatments |
| 3 | 9 | M | PreB | - | - | - | High | MSC isolation, immunophenotype, subernatants, cel cycle |
| 4 | 4 | M | PreB | 281 | 89 | t(9;22) | High | MSC isolation, immunophenotype, subernatants, side populaton |
| 5 | 9 | M | PreB | 123 | 73.5 | - | High | MSC isolation; PDOs |
| 6 | 9 | F | PreB | 188 | 48.3 | - | High | MSC isolation, PDOs, cell culture, side population |
| 7 | 8 | F | PreB | 23 | 67 | - | High | MSC isolation, PDOs, drug treatments |
| 8 | 11 | M | PreB | 143 | 56 | - | High | MSC isolation, immunophenotype, PDOs |
| 9 | 10 | M | PreB | 121 | 78 | - | High | MSC isolation, immunophenotype, subernatants |
| 10 | 9 | F | PreB | 109 | 67 | - | High | MSC isolation, hypoxia assays |
| 11 | 10 | M | PreB | 71.7 | 90.4 | - | High | MSC isolation, PDOs |
| 12 | 15 | M | PreB | - | - | - | High | MSC isolation, PDOs, cell culture |
| 13 | 13 | F | PreB | - | - | - | Standard | MSC isolation |
| 14 | 7 | F | PreB | 45.6 | 98.6 | - | High | MSC isolation, PDOs |
| 15 | 8 | M | PreB | 112 | 78.6 | - | Standard | MSC isolation, PDOs |
| 16 | 7 | M | PreB | 108.6 | 94.6 | - | Standard | MSC isolation, PDOs, cell culture |
| 17 | 14 | M | PreB | 110.6 | 56.4 | - | High | MSC isolation, immunophenotype, PDOs |
| 18 | 14 | M | PreB | 2.2 | 0.07 | - | High | MSC isolation |
| 19 | 12 | M | PreB | 187 | 92.33 | - | High | MSC isolation, immunophenotype, PDOs |
| 20 | 8 | M | PreB | 5.1 | 70 | - | High | MSC isolation |
| 21 | 3 | F | PreB | 293.4 | 94.6 | - | High | MSC isolation, immunophenotype, subernatants |
| 22 | 2 | F | PreB | 7.1 | 67 | - | High | MSC isolation, immunophenotype, PDOs, cell cycle |
| 23 | 9 | M | PreB | - | - | - | High | MSC isolation, immunophenotype, PDOs, cell cycle, side population |
| 24 | 4 | M | PreB | 281 | 89 | t (9;22) | High | MSC isolation, immunophenotype, PDOs, drug treatments |
| 25 | 9 | M | PreB | 123 | 73.5 | - | HIgh | MSC isolation, immunophenotype, PDOs |
| 26 | 9 | F | PreB | 188 | 48.3 | - | High | MSC isolation, PDOs, cell culture, PDXs |
| 27 | 8 | F | PreB | 23 | 67 | - | High | MSC isolation, PDOs, cell culture, drug treatments |
| 28 | 11 | M | PreB | 143 | 56 | - | High | MSC isolation, PDOs, cell culture |
| 29 | 10 | M | PreB | 121 | 78 | - | High | MSC isolation, PDOs, cell culture, side population |
| 30 | 9 | F | PreB | 109 | 67 | - | High | MSC isolation, PDOs, cell culture |
| 31 | 10 | M | PreB | 71.7 | 90.4 | - | High | PDxs |
| 32 | 15 | M | PreB | - | - | - | HIgh | MSC isolation, PDOs, cell culture, cell cycle |
| 33 | 13 | F | PreB | - | - | - | Standard | Drug treatments |
| 34 | 7 | M | PreB | 45.6 | 98.6 | - | HIgh | MSC isolation, PDOs, cell culture |
| 35 | 8 | M | PreB | 112 | 78.6 | - | HIgh | MSC isolation, PDOs, cell culture |
| 36 | 7 | M | PreB | 108.6 | 94.6 | - | HIgh | MSC isolation, PDOs, cell culture |
| 37 | 14 | F | PreB | 110.6 | 56.4 | - | HIgh | MSC isolation, PDOs, cell culture |
| 38 | 7 | F | PreB | 110.8 | 84 | - | HIgh | MSC isolation, PDOs, cell culture |
| 39 | 8 | F | PreB | - | - | - | HIgh | MSC isolation, PDXs, |
| 40 | 8 | F | PreB (relapse) | - | - | - | High | MSC isolation |
| 41 | 7 | F | PreB (relpase) | 110.8 | 84 | - | High | Drug treatments |
| 42 | 14 | F | ProB | 108.3 | 67 | - | High | MSC isolation, PDXs, |
| 43 | 5 | F | ProB | 53.5 | 87.8 | t(12;21) | High | MSC isolation, immunophenotype, subernatants, PDOs, |
| 44 | 10 | F | ProB | 164.5 | 85 | t(9;22) | High | MSC isolation |
| 45 | 12 | F | ProB | 4.9 | 72 | t(1;19) | High | MSC isolation, PDOs |
| 46 | 4 | F | ProB | 58.6 | 83.1 | t(12;21) | High | MSC isolation |
| 47 | 6 | F | ProB | 34 | 12 | - | Standard | MSC isolation, RNA-seq |
| 48 | 14 | F | ProB | 45.6 | 65 | - | High | MSC isolation |
| 49 | 9 | F | ProB | 121.4 | 78 | - | High | MSC isolation |
| 50 | 9 | F | ProB | 112 | 56 | - | High | MSC isolation, RNA-seq |
| 51 | 5 | F | ProB | 60.4 | 49.5 | - | Standard | MSC isolation, PDOs, cell cycle |
| 52 | 16 | M | ProB | 45.7 | 28.5 | - | High | MSC isolation |
| 53 | 5 | F | ProB | 34.5 | 86.4 | - | Standard | MSC isolation |
| 54 | 15 | F | ProB | - | - | - | High | MSC isolation, RNA-seq |
| 55 | 10 | M | ProB | 12.5 | 30.4 | t (1;19) | HIgh | MSC isolation, PDOs, drug treatments |
| 56 | 3 | F | ProB | 104.9 | 82.53 | - | HIgh | MSC isolation |
| 57 | 15 | F | ProB | 58.3 | 47 | - | Standard | MSC isolation, immunophenotype, subernatants |
| 58 | 14 | F | ProB | 108.3 | 67 | t (15;17) | HIgh | MSC isolation |
| 59 | 5 | F | ProB | 53.5 | 87.8 | t (12;21) | HIgh | MSC isolation, immunophenotype, subernatants |
| 60 | 10 | F | ProB | 164.5 | 85 | t (9;22) | HIgh | MSC isolation, immunophenotype, PDOs |
| 61 | 7 | M | ProB | 107.9 | 72 | - | Standard | MSC isolation, immunophenotype, PDOs, drug treatments |
| 62 | 12 | F | ProB | 4.9 | 70 | t (1;19) | HIgh | MSC isolation, immunophenotype, PDOs, cell cycle |
| 63 | 8 | F | ProB | 5.1 | 92.3 | - | HIgh | MSC isolation, hypoxia assays |
| 64 | 3 | F | ProB | 118.9 | 83.1 | - | HIgh | MSC isolation |
| 65 | 4 | F | ProB | 58.6 | 90.8 | t (12;21) | HIgh | MSC isolation, PDOs |
| 66 | 4 | F | ProB | 47.9 | 90 | - | High | MSC isolation, PDOs |
| 67 | 7 | M | ProB | 285.5 | 43.2 | - | Standard | MSC isolation |
| 68 | 11 | F | ProB | - | - | - | HIgh | drug treatments |
| 69 | 6 | F | ProB | 34 | 26 | - | Standard | MSC isolation |
| 70 | 14 | F | ProB | 45.6 | 65 | - | HIgh | MSC isolation, immunophenotype, subernatants |
| 71 | 14 | M | ProB | 78.5 | 80.02 | - | HIgh | cell culture, hypoxia assays |
| 72 | 9 | F | ProB | 121.4 | 78 | - | HIgh | MSC isolation |
| 73 | 9 | F | ProB | 112 | 56 | - | HIgh | PDXs |
| 74 | 5 | F | ProB | 60.4 | 49.5 | - | HIgh | cell culture, hypoxia assays |
| 75 | 9 | F | ProB | 83.51 | 83.5 | - | HIgh | MSC isolation |
| 76 | 8 | F | ProB | - | - | - | HIgh | cell culture |
| 77 | 9 | F | ProB | - | - | - | Standard | MSC isolation, immunophenotype, PDOs |
| 78 | 16 | M | ProB | 45.7 | 28.5 | - | HIgh | , PDOs |
| 79 | 5 | F | ProB | 34.5 | 86.4 | - | HIgh | MSC isolation |
| 80 | 15 | F | ProB | - | - | - | HIgh | MSC isolation, immunophenotype, subernatants |
| 81 | 7 | M | ProB | 29 | 83.74 | - | HIgh | MSC isolation, immunophenotype, PDOs |
| 82 | 8 | M | ProB | 89.4 | 70 | - | HIgh | MSC isolation, immunophenotype, PDOs |
| 83 | 7 | M | ProB | - | - | - | HIgh | MSC isolation |
| 84 | 13 | F | ProB | 60.3 | 81 | - | HIgh | MSC isolation, drug treatments |
| 85 | 8 | M | ProB (reapse) | 89.4 | 70 | - | High | MSC isolation, immunophenotype, PDOs |
| 86 | 7 | M | ProB (relapse) | - | - | - | High | MSC isolation, immunophenotype, PDOs |
| 87 | 8 | F | ProB | 48.6 | 80 | - | High | PDXs |
| 88 | 7 | F | ProB/PreB | 117 | 71.23 | t(15;17) | High | MSC isolation, immunophenotype, subernatants |
| 89 | 9 | M | ProB/PreB | 100.7 | 81 | t(1;19) | High | MSC isolation, immunophenotype, PDOs |
| 90 | 3 | M | ProB/PreB | 119.2 | 83 | - | High | MSC isolation, immunophenotype, PDOs |
| 91 | 2 | M | ProB/PreB | 160.5 | 86.35 | t(1;19) | High | MSC isolation, immunophenotype, PDOs |
| 92 | 4 | F | ProB/PreB | 37.5 | 87 | t(12;21) | High | MSC isolation, immunophenotype, PDOs |
| 93 | 12 | F | ProB/PreB | 25.6 | 62.4 | t(1;19) | High | MSC isolation, immunophenotype, PDOs |
| 94 | 9 | M | ProB/PreB | 55 | 86.6 | t(12;21) | High | MSC isolation, immunophenotype, PDOs |
| 95 | 16 | M | ProB/PreB | 9.5 | 74.9 | t(16;21) | High | MSC isolation, immunophenotype, PDOs |
| 96 | 4 | M | ProB/PreB | 64.6 | 89.7 | t(12;21) | High | MSC isolation, immunophenotype, PDOs |
| 97 | 3 | F | ProB/PreB | 123 | 66 | - | High | MSC isolation, immunophenotype, PDOs |
| 98 | 7 | M | ProB/PreB | 89 | 65.4 | - | Standard | MSC isolation |
| 99 | 5 | M | ProB/PreB | 45.3 | 65.4 | - | Standard | MSC isolation, immunophenotype, subernatants |
| 100 | 6 | F | ProB/PreB | 12.4 | 37.8 | - | Standard | MSC isolation, immunophenotype, PDOs |
| 101 | 6 | M | ProB/PreB | 27.6 | 68.4 | - | Standard | drug treatments |
| 102 | 15 | F | ProB/PreB | 26.9 | 78.6 | - | High | MSC isolation, immunophenotype, PDOs |
| 103 | 5 | M | ProB/PreB | 29.4 | 76.1 | - | Standard | drug treatments |
| 104 | 4 | M | ProB/PreB | 145.2 | 67.8 | - | High | MSC isolation, hypoxia assays |
| 105 | 6 | F | ProB/PreB | 43.2 | 25 | - | Standard | MSC isolation, immunophenotype, PDOs |
| 106 | 2 | F | ProB/PreB | 134.5 | 89.4 | - | High | MSC isolation, cell cycle |
| 107 | 14 | M | ProB/PreB | 23.5 | 76.4 | - | High | MSC isolation, immunophenotype, PDOs |
| 108 | 8 | F | ProB/PreB | 48.6 | 71.2 | - | HIgh | MSC isolation, immunophenotype, PDOs |
| 109 | 7 | F | ProB/PreB | 117 | 88.5 | t (15;17) | HIgh | MSC isolation, immunophenotype, PDOs |
| 110 | 14 | M | ProB/PreB | 62.3 | 81 | - | HIgh | MSC isolation, immunophenotype, PDOs |
| 111 | 9 | M | ProB/PreB | 100.7 | 85 | t (1;19) | HIgh | MSC isolation, immunophenotype, PDOs |
| 112 | 11 | M | ProB/PreB | 270 | 83 | t (12;21) | HIgh | MSC isolation, immunophenotype, PDOs |
| 113 | 3 | M | ProB/PreB | 119.2 | 92.2 | - | HIgh | MSC isolation, immunophenotype, PDOs |
| 114 | 7 | F | ProB/PreB | 33.9 | 86.4 | - | HIgh | MSC isolation, immunophenotype, PDOs |
| 115 | 2 | M | ProB/PreB | 160.5 | 87 | - | HIgh | MSC isolation, immunophenotype, PDOs, drug treatments |
| 116 | 4 | F | ProB/PreB | 37.5 | 76 | t (1;19) | HIgh | MSC isolation, immunophenotype, PDOs |
| 117 | 3 | F | ProB/PreB | 176.3 | 90.6 | - | HIgh | MSC isolation, immunophenotype, PDOs |
| 118 | 2 | F | ProB/PreB | 45.6 | 93.8 | t (12;21) | HIgh | MSC isolation, immunophenotype, PDOs |
| 119 | 8m | M | ProB/PreB | 125.3 | 62.4 | t(16;21) | HIgh | MSC isolation, immunophenotype, PDOs |
| 120 | 3 | F | ProB/PreB | 2150 | 93.9 | t (12;21) | HIgh | MSC isolation, immunophenotype, PDOs |
| 121 | 9 | F | ProB/PreB | 55 | 74 | - | HIgh | MSC isolation, immunophenotype, PDOs |
| 122 | 14 | M | ProB/PreB | 9.5 | 89.7 | - | HIgh | MSC isolation, immunophenotype, PDOs |
| 123 | 31 | M | ProB/PreB | 64.6 | 66 | - | HIgh | MSC isolation, immunophenotype, PDOs |
| 124 | 3 | M | ProB/PreB | 123 | 70 | - | HIgh | MSC isolation, immunophenotype, PDOs |
| 125 | 7 | F | ProB/PreB | 16.5 | 95 | - | HIgh | MSC isolation, immunophenotype, PDOs |
| 126 | 5 | F | ProB/PreB | 162.5 | 65 | - | HIgh | MSC isolation, immunophenotype, PDOs |
| 127 | 6 | M | ProB/PreB | - | - | - | HIgh | MSC isolation, immunophenotype |
| 128 | 6 | M | ProB/PreB | 45.3 | 65.4 | - | HIgh | MSC isolation, immunophenotype |
| 129 | 15 | F | ProB/PreB | 12.4 | 37.8 | - | Standard | MSC isolation, immunophenotype, PDOs |
| 130 | 5 | M | ProB/PreB | 27.6 | 68.4 | - | Standard | MSC isolation, immunophenotype, |
| 131 | 4 | M | ProB/PreB | 26.9 | 78.6 | - | HIgh | drug treatments |
| 132 | 6 | F | ProB/PreB | 145.2 | 76.1 | - | HIgh | MSC isolation, immunophenotype, PDOs |
| 133 | 2 | F | ProB/PreB | 43.2 | 67.8 | - | HIgh | MSC isolation, immunophenotype, PDOs |
| 134 | 5 | M | ProB/PreB | 134.5 | 32 | - | Standard | MSC isolation, immunophenotype, PDOs |
| 135 | 6 | M | ProB/PreB | 40.6 | 89.1 | - | HIgh | MSC isolation, immunophenotype, PDOs |
| 136 | 17 | F | ProB/PreB | 15.6 | 71.3 | - | HIgh | MSC isolation, immunophenotype, PDOs |
| 137 | 2 | M | ProB/PreB | 23.5 | 88.05 | t (12;21) | HIgh | MSC isolation, immunophenotype, PDOs |
| 138 | 2 | M | ProB/PreB | 45.2 | 76.4 | - | HIgh | MSC isolation, immunophenotype, PDOs,drug treatments, |
| 139 | 1 | F | ProB/PreB | 59.9 | 27.6 | - | Standard | MSC isolation, immunophenotype, PDOs |
| 140 | 3 | F | ProB/PreB | 42.1 | 91.4 | - | HIgh | PDOs, drug treatments, |
| 141 | 13 | F | ProB/PreB | 135.6 | 82.3 | - | HIgh | MSC isolation, immunophenotype, PDOs |
| 142 | 12 | F | ProB/PreB | 791 | 67.3 | - | HIgh | MSC isolation, immunophenotype, PDOs |
| 143 | 5 | M | ProB/PreB | 116 | 86 | - | HIgh | MSC isolation, PDOs, cell culture, cell cycle, side population, hypoxia assays, drug treatments, |
| 144 | 15 | F | ProB/PreB | 316.2 | 88 | - | HIgh | MSC isolation, immunophenotype, |
| 145 | 7 | M | ProB/PreB (relapse) | 45.2 | 22.8 | - | High | MSC isolation, immunophenotype, PDOs |
| 146 | 2 | F | ProB/PreB (relapse) | 59.9 | 91.47 | t(12;21) | High | PDOs, cell culture, cell cycle, side population, hypoxia assays, drug treatments, |
| 147 | 1 | M | ProB/PreB (relapse) | 135.6 | 67.3 | - | High | MSC isolation, PDOs, cell culture, cell cycle, side population, |
| HBM1 | 8 | F | Healthy bone marrow donnor | - | - | - | - | MSC isolation, 2D supernatants, immunophenotype, PDOs |
| HBM2 | 6 | M | Healthy bone marrow donnor | - | - | - | - | MSC isolation, 2D supernatants, immunophenotype, PDOs |
| HBM3 | 9 | F | Healthy bone marrow donnor | - | - | - | - | MSC isolation, 2D supernatants, immunophenotype, PDOs |
| HBM4 | 4 | F | Healthy bone marrow donnor | - | - | - | - | MSC isolation, 2D supernatants, immunophenotype, PDOs |
| HBM5 | 5 | M | Healthy bone marrow donnor | - | - | - | - | MSC isolation, 2D supernatants, immunophenotype, PDOs |
| HBM6 | 7 | M | Healthy bone marrow donnor | - | - | - | - | MSC isolation, 2D supernatants, immunophenotype, PDOs |
| HBM7 | 3 | F | Healthy bone marrow donnor | - | - | - | - | MSC isolation, 2D supernatants, immunophenotype, PDOs |
| HBM8 | 5 | M | Healthy bone marrow donnor | - | - | - | - | MSC isolation, 2D supernatants, immunophenotype, PDOs |
| HBM9 | 4 | F | Healthy bone marrow donnor | - | - | - | - | MSC isolation, 2D supernatants, immunophenotype, PDOs |
| HBM10 | 6 | M | Healthy bone marrow donnor | - | - | - | - | MSC isolation, 2D supernatants, immunophenotype, PDOs |
| HBM11 | 2 | M | Healthy bone marrow donnor | - | - | - | - | MSC isolation, 2D supernatants, immunophenotype, PDOs |
| HBM12 | 1 | M | Healthy bone marrow donnor | - | - | - | - | MSC isolation, RNA-seq |

Supplementary Table 2. Human and mouse antibodies.

| **Antibody** | **Origen** | **Clone/Cat** | **Company** |
| --- | --- | --- | --- |
| PDGFR𝛼-PE | Mouse monoclonal | 16A1 | BioLegend |
| CD34-APC | Mouse monoclonal | 581 | BioLegend |
| CD19-FITC | Mouse monoclonal | HI30 | BioLegend |
| CD90-APC | Mouse monoclonal | 5E10 | BioLegend |
| CD105-FITC | Mouse monoclonal | 43A3 | BioLegend |
| CD73-PE | Mouse monoclonal | AD2 | BioLegend |
| CD45-APC/CY7 | Mouse monoclonal | 2D1 | BioLegend |
| CD34-Pacific Blue | Mouse monoclonal | 581 | BioLegend |
| CD10-PE | Mouse monoclonal | H10A | BioLegend |
| CD3-PerCP | Mouse monoclonal | HIT3a | BioLegend |
| CXCR3-PE | Mouse monoclonal | G025H7 | BioLegend |
| CXCR4-PE/Cy7 | Mouse monoclonal | 12G5 | BioLegend |
| CXCR7-APC | Mouse monoclonal | 10D1-J16 | BioLegend |
| Mouse CD45-PE/Cy7 | Rat monoclonal | I3/2.3 | BioLegend |
| Ki-67 PerCP/Cy5.5 | Rat monoclonal | Ki-67 | BioLegend |
| CXCL12 | Rabbit polyclonal | ab9797 | Abcam |
| SCF | Rabbit monoclonal | ab52603 | Abcam |
| HIF-1α | Rabit monoclonal | ab51608 | Abcam |
| CXCL11 | Rabbit polyclonal | ab9955 | Abcam |
| Nestin | Mouse monoclonal | ab6320 | Abcam |
| LepR | Rabbit polyclonal | ab104403 | Abcam |
| IL-7 | Rabbit polyclonal | ab175380 | Abcam |
| Mouse IgG H&L) Alexa Fluor® 405 | Donkey polyclonal | ab175658 | Abcam |
| Mouse IgG H&L Alexa Fluor® 488 | Goat polyclonal | ab150113 | Abcam |
| Rabbit IgG H&L Alexa Fluor® 405 | Goat polyclonal | ab175652 | Abcam |
| Rabbit IgG H&L Alexa Fluor® 488 | Goat polyclonal | ab150077 | Abcam |
| Rabbit IgG H&L) Alexa Fluor® 647 | Goat polyclonal | ab150079 | Abcam |

Supplementary Table S3. RNAseq data.

**ALL-MSC1**

|  | **logFC** | **logCPM** | **LR** | **PValue** | **FDR** | **ALL1** | **NBM** |
| --- | --- | --- | --- | --- | --- | --- | --- |
| KDM5D | -12.534356 | 3.64904418 | 916.148611 | 3.03E-201 | 6.36E-198 | 4.6093531 | 0 |
| ADGRG7 | -12.320359 | 3.44208701 | 754.064242 | 5.24E-166 | 6.12E-163 | 4.39439158 | 0 |
| NLGN4Y | -12.010737 | 3.13904016 | 765.050662 | 2.14E-168 | 2.65E-165 | 4.11192552 | 0 |
| RPS4Y1 | -11.556932 | 5.47806956 | 1662.70331 | 0 | 0 | 6.42171273 | 0.03420061 |
| UTY | -10.801187 | 1.97694451 | 407.486983 | 1.29E-90 | 5.42E-88 | 2.99336447 | 0 |
| TTTY15 | -9.6976156 | 0.92488184 | 255.506221 | 1.64E-57 | 4.58E-55 | 2.09195491 | 0 |
| CXCL10 | -9.3075098 | 3.26985562 | 588.819063 | 4.53E-130 | 3.28E-127 | 4.20758545 | 0.0348435 |
| ZFY | -8.8594733 | 2.04878054 | 457.652354 | 1.56E-101 | 7.27E-99 | 3.07676474 | 0.01676674 |
| HOXD11 | -8.6908193 | -0.0078663 | 142.665292 | 6.96E-33 | 1.00E-30 | 1.38486911 | 0 |
| CXCL6 | -8.5542006 | 3.03693359 | 684.832934 | 5.94E-151 | 6.24E-148 | 4.00738253 | 0.05044505 |
| TXLNGY | -8.3968917 | 1.6131938 | 343.282051 | 1.23E-76 | 4.53E-74 | 2.66652174 | 0.01676674 |
| CXCL8 | -8.3146372 | 5.98754947 | 1080.97537 | 4.51E-237 | 1.18E-233 | 6.89336528 | 0.45664098 |
| CSF3 | -7.5832532 | -1.0037925 | 73.816698 | 8.57E-18 | 5.77E-16 | 0.8143737 | 0 |
| TTTY14 | -7.4885965 | -1.0838229 | 69.908931 | 6.21E-17 | 3.88E-15 | 0.77341146 | 0 |
| USP9Y | -7.408661 | 3.21362297 | 679.787953 | 7.43E-150 | 7.43E-147 | 4.17172307 | 0.1322899 |
| DDX3Y | -7.2664122 | 3.93640914 | 818.108087 | 6.24E-180 | 9.36E-177 | 4.87375594 | 0.2395204 |
| CD38 | -7.0732278 | -1.4507668 | 46.5849073 | 8.77E-12 | 2.80E-10 | 0.61170032 | 0 |
| CXCL11 | -6.8610238 | 1.4307477 | 228.06821 | 1.57E-51 | 3.63E-49 | 2.46424823 | 0.05166739 |
| EIF1AY | -6.7102949 | 1.92460628 | 364.54741 | 2.88E-81 | 1.10E-78 | 2.94172434 | 0.08291571 |
| HLA-DQB1 | -6.6437039 | -1.7674019 | 42.3283119 | 7.72E-11 | 2.05E-09 | 0.47348727 | 0 |
| ZIC1 | -6.5437951 | 2.20396159 | 434.910547 | 1.39E-96 | 6.07E-94 | 3.20977316 | 0.11561399 |
| BARX1 | -6.4864307 | -1.9009929 | 35.2630528 | 2.88E-09 | 5.58E-08 | 0.43648992 | 0 |
| RSAD2 | -6.4771789 | 5.45161207 | 1222.57475 | 7.57E-268 | 3.97E-264 | 6.3796411 | 0.92736046 |
| HOXD10 | -6.4393545 | -0.2091298 | 120.565717 | 4.76E-28 | 5.61E-26 | 1.25162918 | 0.01743386 |
| CD163 | -6.4011743 | -1.9618401 | 32.8364586 | 1.00E-08 | 1.71E-07 | 0.41077013 | 0 |
| CXCL1 | -6.3364827 | 4.90466956 | 1090.0799 | 4.73E-239 | 1.42E-235 | 5.83425514 | 0.74946765 |
| MPEG1 | -6.2869381 | -2.029632 | 31.5936906 | 1.90E-08 | 3.02E-07 | 0.37450071 | 0 |
| TSPAN8 | -6.2765136 | -0.337839 | 92.2955614 | 7.47E-22 | 6.79E-20 | 1.13783117 | 0.01743386 |
| TNF | -6.1998246 | -2.0938764 | 29.2667009 | 6.31E-08 | 8.94E-07 | 0.3547852 | 0 |
| ELF3 | -6.1160343 | -0.4984467 | 101.568652 | 6.90E-24 | 7.07E-22 | 1.07283006 | 0.01676674 |
| HLA-DRB1 | -6.0937017 | 3.2662295 | 652.781912 | 5.55E-144 | 5.06E-141 | 4.21733288 | 0.32528087 |
| FMO3 | -6.0683145 | 1.03490564 | 242.904649 | 9.15E-55 | 2.37E-52 | 2.16501128 | 0.06717951 |
| IGF1 | -6.0583796 | 1.9236317 | 342.469329 | 1.85E-76 | 6.70E-74 | 2.93313797 | 0.13334505 |
| PRKY | -6.0541428 | 1.91905225 | 366.532187 | 1.06E-81 | 4.14E-79 | 2.93577574 | 0.12918928 |
| CHL1 | -5.8733899 | -0.7082986 | 82.9556905 | 8.39E-20 | 6.67E-18 | 0.94458867 | 0.01676674 |
| EHF | -5.7626088 | -2.389521 | 21.6557489 | 3.26E-06 | 3.04E-05 | 0.26572174 | 0 |
| TDRD1 | -5.7392257 | -2.3909288 | 19.5226621 | 9.94E-06 | 8.14E-05 | 0.25722107 | 0 |
| NBAT1 | -5.7285167 | -2.4271726 | 22.3428843 | 2.28E-06 | 2.21E-05 | 0.26847929 | 0 |
| ARAP2 | -5.7259393 | -2.4276532 | 21.5818673 | 3.39E-06 | 3.13E-05 | 0.26751244 | 0 |
| SLC7A2 | -5.7232831 | 2.61997387 | 478.43633 | 4.68E-106 | 2.46E-103 | 3.58685178 | 0.26268989 |
| SIGLEC1 | -5.7172439 | -2.428995 | 22.0008416 | 2.73E-06 | 2.59E-05 | 0.2621787 | 0 |
| FMO2 | -5.7129231 | -0.1202814 | 118.878576 | 1.11E-27 | 1.28E-25 | 1.29571792 | 0.03421096 |
| NOD2 | -5.6848373 | -0.8841858 | 70.5683242 | 4.45E-17 | 2.81E-15 | 0.86784329 | 0.01676674 |
| LINC02225 | -5.6596544 | -2.4705213 | 21.7509504 | 3.10E-06 | 2.91E-05 | 0.2568921 | 0 |
| MMP3 | -5.653812 | 5.46596325 | 1136.4602 | 3.93E-249 | 1.38E-245 | 6.3807145 | 1.39757679 |
| MIR6891 | -5.5916407 | -2.5143762 | 20.7619891 | 5.20E-06 | 4.60E-05 | 0.24577417 | 0 |
| MEFV | -5.5849956 | -0.9831629 | 57.570956 | 3.26E-14 | 1.50E-12 | 0.81861176 | 0.01676674 |
| MX1 | -5.5756641 | 8.36847491 | 1461.93659 | 7.30624277070035e-320 | 7.67009366068123e-316 | 9.27616456 | 3.80574554 |
| MIR4712 | -5.5721607 | -2.5169552 | 19.596745 | 9.56E-06 | 7.86E-05 | 0.23596101 | 0 |
| MT1F | -5.5329526 | 3.96206572 | 664.539701 | 1.54E-146 | 1.47E-143 | 4.88505758 | 0.66976108 |
| LILRB4 | -5.4604682 | -2.6042895 | 18.4358173 | 1.76E-05 | 0.00013397 | 0.23095679 | 0 |
| APOBEC3B | -5.4530289 | 0.11269808 | 136.778371 | 1.35E-31 | 1.81E-29 | 1.46214362 | 0.05051291 |
| SLFN12L | -5.4494479 | -2.6057205 | 18.6931381 | 1.54E-05 | 0.000119 | 0.22628276 | 0 |
| CCL8 | -5.4491095 | 1.17539003 | 256.443212 | 1.02E-57 | 2.90E-55 | 2.28023882 | 0.11652116 |
| CCL20 | -5.3659847 | 0.03317162 | 122.157833 | 2.13E-28 | 2.57E-26 | 1.40271163 | 0.05037818 |
| SNORA26 | -5.2891078 | -2.7038892 | 16.6734526 | 4.44E-05 | 0.00030288 | 0.20583042 | 0 |
| CXCL13 | -5.2864008 | -2.7042296 | 16.7200456 | 4.33E-05 | 0.00029686 | 0.20446492 | 0 |
| RPS4Y2 | -5.2845223 | -2.7045699 | 16.525509 | 4.80E-05 | 0.00032573 | 0.20241847 | 0 |
| SLC12A5 | -5.1846761 | -2.758001 | 15.5804582 | 7.91E-05 | 0.00050721 | 0.18647834 | 0 |
| SLFN14 | -5.1813715 | -2.7579237 | 13.7954067 | 0.00020383 | 0.00115824 | 0.18112782 | 0 |
| MRC1 | -5.1492617 | -2.7609567 | 14.1003664 | 0.00017331 | 0.00100575 | 0.17359106 | 0 |
| IFIT2 | -5.1269846 | 7.05186083 | 1330.8761 | 2.21E-291 | 1.54E-287 | 7.95101441 | 3.0134581 |
| CP | -5.1259927 | 1.05499521 | 215.925517 | 7.00E-49 | 1.53E-46 | 2.16345906 | 0.13392651 |
| LIPM | -5.1073114 | -2.8101865 | 13.9576377 | 0.00018698 | 0.00107349 | 0.18077621 | 0 |
| GYPE | -5.1012171 | -1.3725362 | 45.9137979 | 1.24E-11 | 3.86E-10 | 0.63022555 | 0.01676674 |
| C1QC | -5.1002919 | -2.8104503 | 14.6105763 | 0.00013217 | 0.00079812 | 0.1803861 | 0 |
| HPGD | -5.082724 | -2.8126565 | 14.3188517 | 0.00015431 | 0.00091137 | 0.17182217 | 0 |
| CPVL | -5.0766522 | -2.8134172 | 14.2884535 | 0.00015682 | 0.00092439 | 0.16960378 | 0 |
| BRINP2 | -5.0431299 | -2.8639935 | 12.5187255 | 0.00040289 | 0.00209801 | 0.17506294 | 0 |
| DCLK3 | -5.0102423 | -0.7501397 | 70.9906055 | 3.59E-17 | 2.28E-15 | 0.92657646 | 0.03420061 |
| TENM1 | -5.0015109 | -2.8664733 | 13.1890566 | 0.00028159 | 0.00153924 | 0.16744245 | 0 |
| CMPK2 | -5.0001025 | 4.84343976 | 885.956273 | 1.11E-194 | 1.94E-191 | 5.74644805 | 1.40406551 |
| EREG | -4.9884708 | 0.04953905 | 120.227668 | 5.64E-28 | 6.62E-26 | 1.40095698 | 0.06614897 |
| AQP9 | -4.9838544 | -2.8684222 | 13.6325859 | 0.00022229 | 0.00125128 | 0.16356496 | 0 |
| ALOX15B | -4.9744552 | -2.8695276 | 13.4164262 | 0.00024943 | 0.00138509 | 0.15918673 | 0 |
| SPANXA2-OT1 | -4.9527789 | -2.870974 | 12.6185334 | 0.00038194 | 0.00200782 | 0.1517606 | 0 |
| MX2 | -4.9093191 | 6.75952157 | 1212.63218 | 1.10E-265 | 4.60E-262 | 7.65309737 | 2.93936724 |
| SULT1C4 | -4.8753125 | -0.0566231 | 105.608919 | 8.98E-25 | 9.52E-23 | 1.33244739 | 0.06623653 |
| C1QA | -4.8704236 | -2.9271015 | 12.5365993 | 0.00039906 | 0.00208061 | 0.14970914 | 0 |
| PDZK1IP1 | -4.8416534 | -2.9287114 | 10.889616 | 0.00096705 | 0.00446049 | 0.14281078 | 0 |
| NKD1 | -4.834681 | -2.9831437 | 10.297506 | 0.0013321 | 0.0058721 | 0.15216461 | 0 |
| CXCL5 | -4.8280324 | 0.60975183 | 159.25237 | 1.65E-36 | 2.60E-34 | 1.80020715 | 0.11661472 |
| TAS2R4 | -4.822032 | -2.9301611 | 11.0287075 | 0.00089712 | 0.00418203 | 0.137182 | 0 |
| XAGE3 | -4.822032 | -2.9301611 | 11.0287075 | 0.00089712 | 0.00418203 | 0.137182 | 0 |
| OASL | -4.8212228 | 3.86743086 | 632.205151 | 1.66E-139 | 1.34E-136 | 4.77373852 | 0.94755616 |
| VAT1L | -4.7705304 | -2.9857972 | 11.5841618 | 0.00066516 | 0.00323055 | 0.14657553 | 0 |
| HTR1D | -4.763465 | -2.9864857 | 11.6112539 | 0.00065554 | 0.00319269 | 0.14361198 | 0 |
| IDO1 | -4.7590782 | 2.06761897 | 351.341655 | 2.16E-78 | 8.11E-76 | 3.05457066 | 0.33956898 |
| HLA-DQA1 | -4.7509246 | -2.9880223 | 11.6710671 | 0.0006348 | 0.00310608 | 0.13929208 | 0 |
| MGAT4C | -4.7492883 | -2.9879421 | 11.1413767 | 0.00084423 | 0.00396278 | 0.13639033 | 0 |
| KLHDC7B | -4.745569 | 1.69878014 | 260.14122 | 1.60E-58 | 4.60E-56 | 2.71995374 | 0.26770288 |
| PDE6A | -4.7220285 | -2.9898209 | 10.5872858 | 0.00113868 | 0.00513152 | 0.12903482 | 0 |
| MMP1 | -4.7016238 | 7.21905066 | 896.848084 | 4.75E-197 | 9.07E-194 | 8.09464378 | 3.52700266 |
| SAMSN1 | -4.6932825 | -2.9913544 | 10.0845663 | 0.00149516 | 0.00646601 | 0.12345301 | 0 |
| BTC | -4.6917318 | -0.2172232 | 102.271504 | 4.84E-24 | 4.98E-22 | 1.22681293 | 0.06846871 |
| CAMK1G | -4.6771878 | -0.5668172 | 76.0914532 | 2.71E-18 | 1.90E-16 | 1.00665468 | 0.05164482 |
| C15orf48 | -4.6652271 | 1.9151829 | 328.147949 | 2.43E-73 | 8.37E-71 | 2.91394261 | 0.32679333 |
| CNTN2 | -4.6537623 | -3.0492775 | 9.78283723 | 0.00176148 | 0.00745046 | 0.13109678 | 0 |
| TSPEAR-AS1 | -4.6473929 | -3.0490134 | 10.4827174 | 0.00120496 | 0.00538859 | 0.13585305 | 0 |
| RXRG | -4.6423616 | -3.0502108 | 9.64888792 | 0.00189466 | 0.00791226 | 0.13024688 | 0 |
| HSD3BP4 | -4.6349946 | -3.0503937 | 9.79965835 | 0.00174544 | 0.00739155 | 0.12694688 | 0 |
| LRFN5 | -4.6340175 | 0.42641495 | 137.735785 | 8.32E-32 | 1.13E-29 | 1.67276989 | 0.11432479 |
| CD84 | -4.6293279 | -3.0504748 | 10.6780798 | 0.00108413 | 0.00492051 | 0.13123875 | 0 |
| A3GALT2 | -4.6261913 | -3.0509007 | 10.6073952 | 0.00112636 | 0.00508692 | 0.13020552 | 0 |
| GDF10 | -4.6234676 | -3.0511645 | 10.1584935 | 0.00143638 | 0.00625744 | 0.12590172 | 0 |
| HERC5 | -4.6139202 | 2.91885881 | 453.942998 | 1.00E-100 | 4.56E-98 | 3.84489049 | 0.62770091 |
| DGCR5 | -4.5976583 | -3.0530493 | 9.84724016 | 0.00170087 | 0.00722026 | 0.11898463 | 0 |
| RNASE1 | -4.5874259 | -3.0535554 | 9.4906231 | 0.00206525 | 0.00851402 | 0.11894114 | 0 |
| MMP8 | -4.5863105 | 3.86727504 | 532.842633 | 6.80E-118 | 4.33E-115 | 4.75966214 | 1.05715528 |
| SNAP25 | -4.5856216 | 0.39236161 | 132.725937 | 1.04E-30 | 1.35E-28 | 1.63185132 | 0.11567325 |
| SLC19A3 | -4.5803203 | -0.325322 | 87.2060034 | 9.78E-21 | 8.28E-19 | 1.1704738 | 0.06846871 |
| C3 | -4.5728069 | 6.36901764 | 921.975806 | 1.64E-202 | 3.82E-199 | 7.24629871 | 2.87939506 |
| MIR656 | -4.5722434 | -3.0545879 | 9.53238661 | 0.00201877 | 0.00834867 | 0.11380605 | 0 |
| MMP10 | -4.5296482 | -0.0798032 | 93.3227679 | 4.44E-22 | 4.09E-20 | 1.29438974 | 0.08291571 |
| PIK3AP1 | -4.5131491 | -0.3735269 | 80.9525362 | 2.31E-19 | 1.80E-17 | 1.12319694 | 0.06787892 |
| COL22A1 | -4.5019225 | -0.1165783 | 104.662428 | 1.45E-24 | 1.52E-22 | 1.2978777 | 0.08523546 |
| GBP5 | -4.4926189 | 1.47268756 | 247.519002 | 9.02E-56 | 2.37E-53 | 2.50758713 | 0.27039017 |
| ERVW-1 | -4.4882212 | -3.11702 | 9.45807137 | 0.00210222 | 0.00863255 | 0.11823332 | 0 |
| HORMAD1 | -4.4861991 | -3.1171017 | 9.60264995 | 0.00194297 | 0.00808934 | 0.11971691 | 0 |
| FAM151A | -4.4822076 | -3.1174475 | 9.67504235 | 0.00186788 | 0.00781547 | 0.11853875 | 0 |
| NPY4R | -4.4822076 | -3.1174475 | 9.67504235 | 0.00186788 | 0.00781547 | 0.11853875 | 0 |
| ELFN2 | -4.4822076 | -3.1174475 | 9.67504235 | 0.00186788 | 0.00781547 | 0.11853875 | 0 |
| TRPA1 | -4.4746462 | 1.38249608 | 199.199737 | 3.12E-45 | 6.56E-43 | 2.41072109 | 0.25347873 |
| SCARNA27 | -4.468467 | -3.1189113 | 9.46517407 | 0.0020941 | 0.00860592 | 0.11195427 | 0 |
| C9orf131 | -4.4651608 | -3.1193383 | 9.4957735 | 0.00205946 | 0.00849349 | 0.11159139 | 0 |
| MT1G | -4.4632986 | 4.00005079 | 495.759551 | 7.95E-110 | 4.77E-107 | 4.88797103 | 1.18315553 |
| OR3A1 | -4.4605707 | -3.1196835 | 9.40596038 | 0.00216281 | 0.00885366 | 0.10951899 | 0 |
| TTTY10 | -4.4484675 | -3.1204554 | 9.17470518 | 0.00245383 | 0.00985097 | 0.10652467 | 0 |
| SIM1 | -4.4217456 | -1.2407137 | 48.1870287 | 3.87E-12 | 1.32E-10 | 0.6763893 | 0.03296855 |
| PLEKHS1 | -4.3436327 | 0.75387242 | 169.509503 | 9.47E-39 | 1.63E-36 | 1.91484485 | 0.17639603 |
| CXCL9 | -4.2835532 | -1.324479 | 35.5252287 | 2.52E-09 | 4.95E-08 | 0.60646413 | 0.03421096 |
| SIRPB2 | -4.1825874 | -2.0598778 | 23.703307 | 1.12E-06 | 1.18E-05 | 0.35772877 | 0.01743386 |
| GBP6 | -4.1800714 | -2.0603389 | 22.6923039 | 1.90E-06 | 1.88E-05 | 0.3547852 | 0.01676674 |
| MCF2L | -4.0959049 | -2.0969401 | 18.2574492 | 1.93E-05 | 0.0001447 | 0.32756097 | 0.01676674 |
| HSD11B1 | -4.0647728 | 4.34663722 | 642.238148 | 1.09E-141 | 9.53E-139 | 5.21852163 | 1.66032861 |
| IFIT1B | -4.0526617 | -1.5256355 | 34.8257634 | 3.61E-09 | 6.83E-08 | 0.5418011 | 0.03421096 |
| MAOB | -4.0227756 | 0.46968146 | 134.443825 | 4.37E-31 | 5.77E-29 | 1.68080001 | 0.17894236 |
| PLEKHA6 | -4.0096576 | -0.5446305 | 70.1576261 | 5.47E-17 | 3.44E-15 | 1.02549807 | 0.08464566 |
| ADAM28 | -3.9917052 | -0.8199675 | 58.3657751 | 2.18E-14 | 1.02E-12 | 0.86645445 | 0.0672118 |
| OAS3 | -3.9677515 | 6.03768195 | 482.214262 | 7.05E-107 | 3.79E-104 | 6.88491351 | 3.06307742 |
| CYTL1 | -3.9267909 | 3.45067882 | 472.001027 | 1.18E-104 | 5.74E-102 | 4.33324689 | 1.17059749 |
| IFIT1 | -3.9215396 | 7.71495654 | 809.112906 | 5.63E-178 | 7.89E-175 | 8.55565864 | 4.69778708 |
| CACNA1H | -3.8981212 | -2.2656668 | 18.359148 | 1.83E-05 | 0.00013831 | 0.30714308 | 0.01743386 |
| IL1RN | -3.8978939 | -2.2656471 | 18.3560834 | 1.83E-05 | 0.00013849 | 0.30714308 | 0.01740964 |
| CFB | -3.8420555 | 6.87145742 | 872.002305 | 1.20E-191 | 1.93E-188 | 7.7153308 | 3.96467725 |
| ANOS1 | -3.8418688 | 3.56195606 | 435.281837 | 1.15E-96 | 5.14E-94 | 4.43279074 | 1.27487596 |
| CHRNA1 | -3.8136302 | -1.7329983 | 24.5672336 | 7.18E-07 | 7.87E-06 | 0.47678747 | 0.03421096 |
| ZIC4 | -3.8014716 | 1.46985273 | 198.201518 | 5.16E-45 | 1.07E-42 | 2.48125033 | 0.39683247 |
| GRIP2 | -3.7585321 | 0.64206293 | 123.370746 | 1.16E-28 | 1.40E-26 | 1.8026595 | 0.23958923 |
| CSF2 | -3.7549776 | -0.9971448 | 37.4759419 | 9.25E-10 | 1.98E-08 | 0.74505682 | 0.06792254 |
| SPATA22 | -3.7438946 | -1.33985 | 34.5244314 | 4.21E-09 | 7.83E-08 | 0.60457174 | 0.04864225 |
| MS4A7 | -3.7403053 | -2.3134356 | 12.052309 | 0.00051728 | 0.00259457 | 0.25399597 | 0.01676674 |
| GBP1P1 | -3.7246444 | 0.78313116 | 142.14034 | 9.06E-33 | 1.29E-30 | 1.90255282 | 0.27043158 |
| MMP13 | -3.710351 | 6.10861537 | 636.581421 | 1.85E-140 | 1.55E-137 | 6.93989045 | 3.36857562 |
| TNFAIP6 | -3.7038635 | 5.48922852 | 615.722331 | 6.37E-136 | 4.95E-133 | 6.32276284 | 2.82741823 |
| IL4I1 | -3.703276 | 2.0514909 | 231.298114 | 3.11E-52 | 7.58E-50 | 2.98625548 | 0.61517672 |
| NLGN4X | -3.7018211 | 2.89652388 | 314.213281 | 2.64E-70 | 8.79E-68 | 3.78420922 | 0.96979329 |
| NRIR | -3.6925428 | -2.3871383 | 15.0688114 | 0.00010366 | 0.00064416 | 0.25917459 | 0.01676674 |
| LGALS9 | -3.6825017 | 3.00800979 | 338.82335 | 1.15E-75 | 4.10E-73 | 3.88936275 | 1.03895158 |
| CNTNAP2 | -3.6753574 | 4.29781712 | 543.634237 | 3.05E-120 | 2.00E-117 | 5.14720334 | 1.88100562 |
| CHI3L2 | -3.6683262 | 4.48929834 | 484.940401 | 1.80E-107 | 9.93E-105 | 5.3246017 | 2.03252991 |
| CXCL3 | -3.6425553 | 2.57651271 | 322.247141 | 4.69E-72 | 1.59E-69 | 3.47765864 | 0.85296015 |
| HLA-DRB5 | -3.625629 | 1.25917853 | 174.192203 | 8.99E-40 | 1.61E-37 | 2.29675112 | 0.39438672 |
| NKAIN3 | -3.5987188 | -1.8744454 | 23.5946707 | 1.19E-06 | 1.24E-05 | 0.41553332 | 0.03296855 |
| LBP | -3.5866858 | 4.13607478 | 461.389314 | 2.40E-102 | 1.14E-99 | 4.97893584 | 1.82122587 |
| MLC1 | -3.57646 | 2.13962727 | 269.251139 | 1.65E-60 | 4.89E-58 | 3.07043101 | 0.69295318 |
| ADAMDEC1 | -3.5741568 | -0.32051 | 64.8994346 | 7.88E-16 | 4.39E-14 | 1.11698015 | 0.13010453 |
| CCR1 | -3.5729818 | 1.16163704 | 177.352813 | 1.83E-40 | 3.41E-38 | 2.21406333 | 0.38162483 |
| SMIM2-AS1 | -3.5631567 | -2.4702973 | 13.4179106 | 0.00024923 | 0.00138437 | 0.24209441 | 0.01743386 |
| FER1L6 | -3.5213006 | 1.57301183 | 196.49693 | 1.21E-44 | 2.50E-42 | 2.56198287 | 0.4994447 |
| SLC7A11-AS1 | -3.5200673 | -2.5104619 | 13.2212478 | 0.00027679 | 0.00151738 | 0.24367481 | 0.01740964 |
| IFIT3 | -3.5188414 | 7.63460699 | 771.973787 | 6.69E-170 | 8.78E-167 | 8.45264464 | 4.98168755 |
| GPR84 | -3.5143812 | -0.3918252 | 61.8043359 | 3.79E-15 | 1.94E-13 | 1.10004835 | 0.12898069 |
| LINC00535 | -3.4956317 | -2.5142141 | 13.5773758 | 0.00022893 | 0.00128587 | 0.23196007 | 0.01676674 |
| CYBB | -3.4935382 | -2.5141523 | 13.3342339 | 0.0002606 | 0.00144111 | 0.23245604 | 0.01743386 |
| TBX1 | -3.4922976 | -0.0016564 | 77.2116985 | 1.54E-18 | 1.11E-16 | 1.33513095 | 0.17753707 |
| IL32 | -3.4685178 | 1.44448677 | 193.165075 | 6.48E-44 | 1.30E-41 | 2.44754762 | 0.48059631 |
| PCDH17 | -3.4600868 | -2.5554687 | 11.1893391 | 0.00082269 | 0.00387637 | 0.23100818 | 0.01743386 |
| TNFSF13B | -3.455983 | 2.8400328 | 296.946495 | 1.52E-66 | 4.85E-64 | 3.70604527 | 1.07116354 |
| IFI44L | -3.4527163 | 7.1881273 | 561.339448 | 4.30E-124 | 3.01E-121 | 7.99724097 | 4.59938982 |
| TLR2 | -3.4487222 | -0.9829355 | 34.1675212 | 5.06E-09 | 9.25E-08 | 0.7467879 | 0.08146228 |
| PREX2 | -3.4444406 | -2.5568845 | 11.5111452 | 0.0006918 | 0.00333833 | 0.22582169 | 0.01676674 |
| ENPP3 | -3.4338684 | -2.5571414 | 12.4746117 | 0.00041252 | 0.00214388 | 0.22771911 | 0.01740964 |
| RNU6-26P | -3.4319303 | -1.9679346 | 17.7679093 | 2.50E-05 | 0.00018143 | 0.36346494 | 0.03421096 |
| NKAIN3-IT1 | -3.4297875 | -2.5575543 | 12.8618458 | 0.00033535 | 0.00179069 | 0.22794345 | 0.01740964 |
| LINC00612 | -3.4279623 | -2.5579125 | 12.8014155 | 0.00034636 | 0.00184338 | 0.22628276 | 0.01743386 |
| ENPEP | -3.4154311 | 1.94236169 | 229.86593 | 6.38E-52 | 1.54E-49 | 2.87461535 | 0.67213802 |
| PEX5L | -3.4095427 | -0.3150887 | 67.4558256 | 2.15E-16 | 1.29E-14 | 1.12641054 | 0.14661023 |
| CCKAR | -3.4049191 | -2.5600151 | 10.3886806 | 0.0012679 | 0.00562928 | 0.21198528 | 0.01743386 |
| IFI27 | -3.3894545 | 4.98169144 | 554.297155 | 1.46E-122 | 9.91E-120 | 5.79929278 | 2.64135418 |
| HERC2P10 | -3.388885 | -2.6017281 | 10.2898987 | 0.0013376 | 0.00589114 | 0.22089021 | 0.01743386 |
| SOD2 | -3.3886627 | 7.98559638 | 744.71379 | 5.66E-164 | 6.25E-161 | 8.7933797 | 5.43943093 |
| RTP4 | -3.3848717 | 2.7259844 | 272.711992 | 2.91E-61 | 8.73E-59 | 3.59123644 | 1.03697113 |
| IL34 | -3.383783 | 1.24019574 | 169.320491 | 1.04E-38 | 1.76E-36 | 2.26996102 | 0.44463304 |
| RNF175 | -3.3597931 | -2.6035758 | 11.4045515 | 0.00073264 | 0.00350481 | 0.21745439 | 0.01743386 |
| ALOX12P2 | -3.3537172 | -2.604541 | 11.7056204 | 0.00062312 | 0.00305676 | 0.21657092 | 0.01676674 |
| RORB | -3.351249 | 0.52709899 | 116.517449 | 3.66E-27 | 4.18E-25 | 1.70437566 | 0.28148142 |
| PKIB | -3.348902 | -0.349352 | 44.9134903 | 2.06E-11 | 6.16E-10 | 1.07517621 | 0.1410562 |
| PAX9 | -3.3482609 | 0.0968889 | 88.4291847 | 5.27E-21 | 4.55E-19 | 1.39509082 | 0.20949478 |
| AGT | -3.3469139 | 2.88483275 | 310.726942 | 1.52E-69 | 4.98E-67 | 3.7496159 | 1.14187657 |
| GCH1 | -3.3397905 | 2.19928145 | 228.09753 | 1.55E-51 | 3.62E-49 | 3.10937171 | 0.79397221 |
| LONRF3 | -3.338286 | -2.6067243 | 11.7754909 | 0.00060016 | 0.00295483 | 0.20957047 | 0.01676674 |
| ISG15 | -3.3304209 | 7.27127774 | 494.9504 | 1.19E-109 | 6.96E-107 | 8.07171786 | 4.7802541 |
| CCL3 | -3.3295859 | -2.0582738 | 17.9874041 | 2.22E-05 | 0.00016448 | 0.34766816 | 0.03420061 |
| CD80 | -3.3122675 | -0.9013745 | 46.8121821 | 7.81E-12 | 2.53E-10 | 0.80962412 | 0.09866468 |
| CX3CL1 | -3.3088251 | 0.62921835 | 112.388752 | 2.94E-26 | 3.26E-24 | 1.77398996 | 0.29801337 |
| SNORD116-24 | -3.2789068 | -2.6516653 | 10.7012755 | 0.00107062 | 0.00487004 | 0.20862923 | 0.01743386 |
| LANCL1-AS1 | -3.2596186 | -2.6538365 | 11.1180291 | 0.00085493 | 0.0040076 | 0.20245228 | 0.01740964 |
| CNTN1 | -3.2594349 | 0.02000093 | 78.6817669 | 7.30E-19 | 5.38E-17 | 1.34238174 | 0.21048697 |
| ALB | -3.2498473 | -2.6552704 | 10.8465406 | 0.00098981 | 0.00455645 | 0.19632279 | 0.01740964 |
| IFIH1 | -3.2130547 | 5.12162583 | 474.278838 | 3.76E-105 | 1.92E-102 | 5.91734668 | 2.8979975 |
| LINC01139 | -3.2110404 | 2.10940273 | 229.531997 | 7.54E-52 | 1.80E-49 | 3.00998345 | 0.81866261 |
| CD200 | -3.190156 | 0.77110502 | 118.90214 | 1.10E-27 | 1.28E-25 | 1.87953023 | 0.35641961 |
| MROCKI | -3.1798572 | 1.3870914 | 170.637881 | 5.37E-39 | 9.32E-37 | 2.3731133 | 0.54612455 |
| SCN11A | -3.1791477 | -2.7028111 | 10.0470564 | 0.00152592 | 0.00657867 | 0.1942002 | 0.01740964 |
| ANGPTL7 | -3.1790713 | -2.1595055 | 16.3581159 | 5.24E-05 | 0.0003525 | 0.31874573 | 0.03417638 |
| SCHLAP1 | -3.1763805 | -0.828465 | 46.3493964 | 9.89E-12 | 3.13E-10 | 0.84644514 | 0.1127466 |
| CDHR5 | -3.1671571 | -2.7048235 | 10.2168179 | 0.00139166 | 0.00608227 | 0.18863818 | 0.01676674 |
| BIRC3 | -3.1609666 | 1.95425198 | 177.925759 | 1.38E-40 | 2.58E-38 | 2.84983974 | 0.76763533 |
| LINC01436 | -3.1439514 | 0.02524905 | 74.820637 | 5.15E-18 | 3.53E-16 | 1.32378942 | 0.22334094 |
| MIR1281 | -3.1397232 | -2.1918581 | 14.8197209 | 0.00011829 | 0.00072389 | 0.31407368 | 0.03421096 |
| TEKT4P2 | -3.1377988 | 2.04575415 | 219.129854 | 1.40E-49 | 3.13E-47 | 2.94854279 | 0.81703692 |
| BST2 | -3.1345442 | 5.36705204 | 389.636473 | 9.93E-87 | 4.01E-84 | 6.15939931 | 3.15450007 |
| BPI | -3.1224667 | -0.8700681 | 43.5088733 | 4.22E-11 | 1.19E-09 | 0.8198279 | 0.11652116 |
| SCN1A | -3.1167643 | -1.8179364 | 20.6593269 | 5.49E-06 | 4.82E-05 | 0.42414387 | 0.05051291 |
| HOXB9 | -3.1156923 | 0.25019256 | 76.6329259 | 2.06E-18 | 1.47E-16 | 1.47624367 | 0.26517346 |
| IGFBP1 | -3.1140627 | -0.1078104 | 65.034484 | 7.36E-16 | 4.14E-14 | 1.25479257 | 0.21092114 |
| RASGEF1B | -3.1123386 | 1.10353405 | 116.119381 | 4.48E-27 | 5.08E-25 | 2.11289452 | 0.46932546 |
| CXCL2 | -3.1092948 | 3.83946905 | 402.2613 | 1.77E-89 | 7.30E-87 | 4.65041347 | 1.92642276 |
| KMO | -3.0861222 | -0.8987287 | 42.9373907 | 5.65E-11 | 1.55E-09 | 0.80488459 | 0.11652116 |
| OAS2 | -3.0813993 | 7.04317368 | 602.297983 | 5.30E-133 | 3.97E-130 | 7.82300595 | 4.7917874 |
| STK32A | -3.0520327 | -2.2628973 | 12.277194 | 0.00045853 | 0.00234069 | 0.29926046 | 0.03425775 |
| CYP7B1 | -3.0366918 | 0.9875753 | 134.442897 | 4.37E-31 | 5.77E-29 | 2.03635203 | 0.46020761 |
| WTAPP1 | -3.0233223 | -2.265705 | 14.3728056 | 0.00014995 | 0.00089013 | 0.2944413 | 0.03425775 |
| ICOSLG | -3.0229728 | -2.2656656 | 14.3669631 | 0.00015042 | 0.00089214 | 0.2944413 | 0.03421096 |
| HERC6 | -3.011764 | 5.70404326 | 515.904422 | 3.29E-114 | 2.03E-111 | 6.4792063 | 3.58448071 |
| HOXD1 | -3.0062653 | -2.2690449 | 13.900536 | 0.00019274 | 0.00110358 | 0.28253594 | 0.03420061 |
| DCHS2 | -3.003959 | -1.3531551 | 30.0362221 | 4.24E-08 | 6.27E-07 | 0.59623421 | 0.08472387 |
| LINC02211 | -2.9933319 | -1.9037249 | 19.0472025 | 1.28E-05 | 0.00010142 | 0.39153811 | 0.0509777 |
| FGF10 | -2.9769656 | -1.3580064 | 24.2002002 | 8.68E-07 | 9.35E-06 | 0.57674935 | 0.08135624 |
| MAB21L1 | -2.9656829 | -1.9308564 | 18.1579478 | 2.03E-05 | 0.00015197 | 0.39033802 | 0.04864225 |
| DSC2 | -2.9591233 | -0.1358861 | 66.087908 | 4.31E-16 | 2.47E-14 | 1.23136775 | 0.22247677 |
| AKAP3 | -2.9536146 | -0.2339041 | 64.063095 | 1.20E-15 | 6.64E-14 | 1.16581009 | 0.21048697 |
| DNAH3 | -2.9470114 | -0.4674903 | 40.9478202 | 1.56E-10 | 3.92E-09 | 1.03252816 | 0.1776796 |
| FGF13 | -2.9445236 | -1.9336543 | 17.0315604 | 3.68E-05 | 0.00025678 | 0.38036528 | 0.05044505 |
| LINC02432 | -2.9245469 | -0.8673047 | 41.2019929 | 1.37E-10 | 3.47E-09 | 0.81958137 | 0.13341604 |
| CHRM5 | -2.9160353 | -2.342305 | 11.4653256 | 0.00070907 | 0.00340911 | 0.27324979 | 0.03296855 |
| ANGPTL1 | -2.9158444 | 2.57836053 | 195.118395 | 2.43E-44 | 4.95E-42 | 3.40195576 | 1.1903146 |
| IL26 | -2.9005022 | 0.35186027 | 79.7785059 | 4.19E-19 | 3.14E-17 | 1.53534014 | 0.32761872 |
| OAS1 | -2.8973714 | 5.41770931 | 473.337442 | 6.02E-105 | 3.01E-102 | 6.18292622 | 3.41372345 |
| PDE10A | -2.8960899 | 1.40713221 | 133.637963 | 6.56E-31 | 8.60E-29 | 2.35755217 | 0.63811202 |
| GTF2IP20 | -2.8836621 | -1.6872133 | 22.1467214 | 2.53E-06 | 2.41E-05 | 0.46424276 | 0.06717951 |
| CCDC144A | -2.8829159 | -1.9657281 | 14.1469899 | 0.00016907 | 0.00098549 | 0.36066237 | 0.05051291 |
| IL18RAP | -2.8813634 | -2.3791194 | 9.90359151 | 0.00164957 | 0.00704092 | 0.26978458 | 0.03421096 |
| TNFRSF18 | -2.8763832 | -2.3467109 | 11.686326 | 0.00062961 | 0.00308485 | 0.25705536 | 0.03417638 |
| GPR37L1 | -2.8635553 | -2.3818536 | 9.32912139 | 0.00225541 | 0.00918078 | 0.25728596 | 0.03296855 |
| SCN3A | -2.8518573 | -0.0486601 | 68.7681473 | 1.11E-16 | 6.76E-15 | 1.27684927 | 0.25434717 |
| STC1 | -2.8448429 | 3.62659362 | 293.221319 | 9.88E-66 | 3.10E-63 | 4.41532581 | 1.92955246 |
| HEPH | -2.8284955 | 5.61193785 | 492.374886 | 4.34E-109 | 2.46E-106 | 6.3697958 | 3.6487588 |
| PLCH1 | -2.824972 | -1.2894989 | 27.2105178 | 1.82E-07 | 2.29E-06 | 0.6243757 | 0.10149061 |
| SYCP3 | -2.822734 | -2.0236752 | 16.4166741 | 5.08E-05 | 0.00034276 | 0.35770181 | 0.0509777 |
| UBE2QL1 | -2.8217111 | -1.1005831 | 27.5820137 | 1.51E-07 | 1.93E-06 | 0.68479651 | 0.11661472 |
| EPHA7 | -2.8205456 | -0.8106932 | 38.5330405 | 5.38E-10 | 1.22E-08 | 0.84458183 | 0.14445802 |
| PPP4R1-AS1 | -2.8055484 | -1.7585447 | 17.7702306 | 2.49E-05 | 0.00018127 | 0.44631291 | 0.06781205 |
| ACTN2 | -2.8008999 | -2.0515542 | 14.1631657 | 0.00016762 | 0.0009795 | 0.35675888 | 0.05164482 |
| LINC01018 | -2.7734115 | -1.579592 | 13.8445319 | 0.00019857 | 0.00113172 | 0.5066432 | 0.07853564 |
| SAMD9 | -2.7713311 | 5.68800009 | 192.709177 | 8.15E-44 | 1.60E-41 | 6.40877512 | 3.72780851 |
| ALK | -2.7392629 | -1.7908029 | 18.9938288 | 1.31E-05 | 0.00010393 | 0.42581634 | 0.06607611 |
| PGR | -2.7310518 | -1.3529349 | 26.6141052 | 2.48E-07 | 3.02E-06 | 0.58631627 | 0.09915515 |
| SALL1 | -2.7245944 | -0.0127444 | 48.7330815 | 2.93E-12 | 1.03E-10 | 1.29390973 | 0.27803598 |
| GLDN | -2.7211417 | -1.5666408 | 17.6188029 | 2.70E-05 | 0.00019421 | 0.49689614 | 0.08123082 |
| DSC3 | -2.7099081 | -2.4655521 | 9.83394603 | 0.0017132 | 0.00726448 | 0.24367481 | 0.03421096 |
| PCDHA6 | -2.7059314 | -2.4658894 | 9.97626911 | 0.00158571 | 0.00680292 | 0.24272443 | 0.03421096 |
| HOXB8 | -2.6978793 | 0.69765143 | 82.6364548 | 9.86E-20 | 7.78E-18 | 1.78128231 | 0.45558966 |
| XAF1 | -2.6967327 | 5.44971228 | 387.046861 | 3.64E-86 | 1.44E-83 | 6.18604557 | 3.60320452 |
| SELL | -2.6856835 | -2.4695249 | 9.95191416 | 0.00160682 | 0.00688217 | 0.23245604 | 0.03296855 |
| LINC01814 | -2.6813566 | -2.4696329 | 9.89569302 | 0.00165666 | 0.00706546 | 0.22922394 | 0.03417638 |
| PLA1A | -2.6766734 | 0.39406612 | 82.5753107 | 1.02E-19 | 8.00E-18 | 1.56248987 | 0.38015208 |
| VANGL2 | -2.6762671 | -2.470452 | 9.89012888 | 0.00166168 | 0.00708541 | 0.22830255 | 0.03417638 |
| DDX58 | -2.6723681 | 5.83984808 | 414.111618 | 4.67E-92 | 2.00E-89 | 6.57187057 | 3.9875168 |
| USP30-AS1 | -2.6661029 | -1.6098227 | 19.7165113 | 8.98E-06 | 7.44E-05 | 0.48925196 | 0.08463594 |
| NKX6-1 | -2.642684 | 1.05839653 | 101.543203 | 6.99E-24 | 7.13E-22 | 2.0650635 | 0.58785441 |
| HELZ2 | -2.6420944 | 6.15972064 | 250.744587 | 1.79E-56 | 4.81E-54 | 6.875202 | 4.29532426 |
| TBX18 | -2.6343566 | 3.30412213 | 260.303285 | 1.47E-58 | 4.30E-56 | 4.07960794 | 1.83118846 |
| PGBD5 | -2.6277827 | -2.1557163 | 12.8195153 | 0.00034302 | 0.00182702 | 0.31665244 | 0.05040241 |
| ELOVL7 | -2.6212728 | -1.8499332 | 13.0285211 | 0.00030678 | 0.00165724 | 0.38399212 | 0.0672118 |
| HOXA-AS3 | -2.6074792 | -1.1129643 | 26.6084499 | 2.49E-07 | 3.02E-06 | 0.68381137 | 0.13184047 |
| HTATSF1P2 | -2.6064485 | 2.89633219 | 217.612701 | 3.00E-49 | 6.63E-47 | 3.68493437 | 1.55757019 |
| SFRP2 | -2.6038635 | 2.45764799 | 181.406441 | 2.39E-41 | 4.52E-39 | 3.27669564 | 1.2693566 |
| NR2F2-AS1 | -2.6003027 | -1.1268935 | 23.2459675 | 1.43E-06 | 1.46E-05 | 0.67749959 | 0.13392651 |
| SERTM2 | -2.5998345 | -2.5141917 | 9.17859966 | 0.00244861 | 0.00983756 | 0.21758683 | 0.03425775 |
| PLP1 | -2.5979626 | -1.6573747 | 19.5511322 | 9.79E-06 | 8.04E-05 | 0.47565497 | 0.08523546 |
| PWAR5 | -2.5970812 | -0.7247462 | 34.3278608 | 4.66E-09 | 8.57E-08 | 0.86079788 | 0.17469186 |
| SMC2-AS1 | -2.5845326 | -1.9009261 | 15.3157998 | 9.10E-05 | 0.00057502 | 0.38799436 | 0.06605188 |
| LINC00865 | -2.5711634 | -0.4476453 | 40.7476421 | 1.73E-10 | 4.31E-09 | 1.01266955 | 0.21707901 |
| PCDHGB2 | -2.5523263 | 2.54694278 | 119.172793 | 9.60E-28 | 1.12E-25 | 3.35305788 | 1.31382193 |
| KIAA1755 | -2.5490909 | 2.7223723 | 164.448932 | 1.21E-37 | 1.93E-35 | 3.51270884 | 1.46217529 |
| PALM2-AKAP2 | -2.5484641 | -1.310882 | 22.4756302 | 2.13E-06 | 2.08E-05 | 0.59623501 | 0.11666188 |
| SDK1 | -2.53504 | 0.8350252 | 82.8980411 | 8.64E-20 | 6.85E-18 | 1.87327036 | 0.53594962 |
| IL1B | -2.5344718 | -1.9315894 | 14.7254813 | 0.00012435 | 0.00075592 | 0.37416002 | 0.06605188 |
| PCDHB2 | -2.5255573 | 1.11537701 | 71.3452145 | 3.00E-17 | 1.92E-15 | 2.08464649 | 0.63961908 |
| GBP4 | -2.5250859 | 2.65370184 | 166.707387 | 3.88E-38 | 6.41E-36 | 3.44465404 | 1.42872438 |
| PI3 | -2.5241665 | -2.2264639 | 10.3842172 | 0.00127097 | 0.00563814 | 0.29458227 | 0.05040241 |
| COL28A1 | -2.5232096 | 0.10472652 | 59.7677562 | 1.07E-14 | 5.20E-13 | 1.34408929 | 0.33752099 |
| CCL13 | -2.5186747 | -1.0352709 | 29.6102951 | 5.28E-08 | 7.63E-07 | 0.71101404 | 0.14746735 |
| LINC01583 | -2.51456 | -1.9567339 | 13.5712875 | 0.00022967 | 0.00128936 | 0.37763848 | 0.06843413 |
| ZBED6 | -2.513279 | 4.01863915 | 68.8523419 | 1.06E-16 | 6.53E-15 | 4.75636408 | 2.31418522 |
| LRRC4C | -2.5122463 | 2.1121387 | 140.405852 | 2.17E-32 | 3.02E-30 | 2.93752865 | 1.11597268 |
| GRIK4 | -2.5098994 | -0.6942873 | 35.4816547 | 2.57E-09 | 5.05E-08 | 0.88026109 | 0.19377116 |
| GMNC | -2.5028214 | -0.6962416 | 38.2404488 | 6.25E-10 | 1.39E-08 | 0.87943594 | 0.19620668 |
| LINC00944 | -2.4879242 | -0.2665873 | 39.7296459 | 2.92E-10 | 6.93E-09 | 1.11880123 | 0.25468849 |
| SLC22A3 | -2.4689016 | 3.22761752 | 224.096863 | 1.16E-50 | 2.64E-48 | 3.9847311 | 1.87655354 |
| IGF2 | -2.4649223 | 0.8137336 | 76.5727169 | 2.12E-18 | 1.50E-16 | 1.85223389 | 0.54924024 |
| MT1H | -2.4592825 | -0.0860072 | 48.5297165 | 3.25E-12 | 1.13E-10 | 1.23149145 | 0.31205225 |
| CLDN1 | -2.4592557 | 2.72808664 | 175.978672 | 3.66E-40 | 6.68E-38 | 3.49992669 | 1.53180731 |
| PDZD2 | -2.4562321 | -0.0798113 | 55.4601063 | 9.54E-14 | 4.10E-12 | 1.22794224 | 0.3107499 |
| CCL5 | -2.432623 | 1.78912446 | 130.975871 | 2.51E-30 | 3.17E-28 | 2.65537621 | 0.97361115 |
| ASPA | -2.4119975 | -1.9970103 | 11.5628614 | 0.00067282 | 0.00325722 | 0.33634411 | 0.06607611 |
| HYDIN2 | -2.4038658 | -1.444485 | 14.5037241 | 0.00013988 | 0.0008389 | 0.54529231 | 0.11717346 |
| FCGR1A | -2.3971314 | -1.2721422 | 21.4978807 | 3.54E-06 | 3.26E-05 | 0.60858806 | 0.13174373 |
| CCDC178 | -2.3866779 | -2.3025499 | 9.57857049 | 0.00196862 | 0.00816698 | 0.27226067 | 0.05044505 |
| MTUS1 | -2.3678125 | 1.56167128 | 108.575674 | 2.01E-25 | 2.18E-23 | 2.44389182 | 0.88614944 |
| ADGRE2 | -2.3558867 | 0.07370132 | 47.7093838 | 4.94E-12 | 1.66E-10 | 1.31668957 | 0.36572585 |
| C7 | -2.3558747 | -0.3711998 | 36.7644243 | 1.33E-09 | 2.77E-08 | 1.04665304 | 0.25984373 |
| RBP1 | -2.3495834 | -0.7030042 | 27.3060732 | 1.74E-07 | 2.20E-06 | 0.84555813 | 0.20321315 |
| TRIL | -2.338126 | 0.40582488 | 55.1306164 | 1.13E-13 | 4.79E-12 | 1.53046145 | 0.45919371 |
| MT1M | -2.3327879 | 2.88401509 | 192.913486 | 7.35E-44 | 1.46E-41 | 3.63693288 | 1.71248677 |
| CYTIP | -2.3298644 | -1.0495466 | 25.2618069 | 5.01E-07 | 5.66E-06 | 0.69336518 | 0.16431549 |
| RBM12B-AS1 | -2.3295588 | -1.8411169 | 14.2305201 | 0.00016173 | 0.00094876 | 0.40495572 | 0.08470281 |
| C3orf20 | -2.3272344 | -1.1958107 | 16.1328269 | 5.91E-05 | 0.00039211 | 0.62765696 | 0.14740401 |
| SLPI | -2.3163749 | 2.12640247 | 121.738097 | 2.63E-28 | 3.16E-26 | 2.92769525 | 1.2115611 |
| CH25H | -2.3123992 | 3.65314279 | 221.27934 | 4.76E-50 | 1.07E-47 | 4.36739966 | 2.31556382 |
| LPAR4 | -2.3103518 | -1.0657797 | 25.0658891 | 5.54E-07 | 6.23E-06 | 0.68776626 | 0.16325951 |
| PDK4 | -2.2928704 | -0.414507 | 37.3906215 | 9.67E-10 | 2.06E-08 | 1.01278299 | 0.26779771 |
| CTSS | -2.292114 | 4.24789456 | 249.799488 | 2.87E-56 | 7.63E-54 | 4.94429159 | 2.8270583 |
| FCMR | -2.2911369 | -1.4963941 | 19.1382287 | 1.22E-05 | 9.72E-05 | 0.51610894 | 0.1177247 |
| PKNOX2 | -2.2890843 | 0.27616924 | 59.1149424 | 1.49E-14 | 7.13E-13 | 1.45185144 | 0.4339354 |
| GIMAP2 | -2.2856736 | 0.60340085 | 68.5388135 | 1.24E-16 | 7.55E-15 | 1.6738097 | 0.52875078 |
| CDKL5 | -2.2843059 | 1.11473049 | 92.3092014 | 7.41E-22 | 6.77E-20 | 2.06806415 | 0.72136899 |
| PCDHB16 | -2.2709478 | 1.31206896 | 97.398964 | 5.67E-23 | 5.48E-21 | 2.2308299 | 0.8193222 |
| STMN2 | -2.2698537 | -0.3456669 | 30.6305643 | 3.12E-08 | 4.76E-07 | 1.0164556 | 0.28470914 |
| SCUBE1 | -2.2608193 | -0.6993564 | 26.9244592 | 2.12E-07 | 2.61E-06 | 0.8709415 | 0.22356214 |
| DDIT4L | -2.2504515 | 3.98394666 | 151.918534 | 6.60E-35 | 1.00E-32 | 4.66859652 | 2.6081105 |
| EFHC2 | -2.2438647 | 1.1351369 | 80.5023138 | 2.90E-19 | 2.22E-17 | 2.0735099 | 0.73531881 |
| FGF11 | -2.2357804 | 0.28019149 | 52.5118127 | 4.28E-13 | 1.69E-11 | 1.43929926 | 0.43186913 |
| ADGRL3 | -2.2353298 | -0.8894599 | 22.9221452 | 1.69E-06 | 1.70E-05 | 0.74552109 | 0.19138187 |
| CCN6 | -2.2333147 | 2.0536131 | 131.518545 | 1.91E-30 | 2.46E-28 | 2.85613365 | 1.22174442 |
| CETP | -2.224004 | -1.5410068 | 15.2973098 | 9.18E-05 | 0.00057911 | 0.49077224 | 0.11668158 |
| FREM1 | -2.2226791 | -1.3923241 | 18.5554091 | 1.65E-05 | 0.00012665 | 0.54730388 | 0.12898069 |
| SLCO2B1 | -2.2212515 | -1.9252716 | 10.0879732 | 0.0014924 | 0.00645672 | 0.37498203 | 0.08472387 |
| HGF | -2.2153776 | 5.52391641 | 253.798353 | 3.86E-57 | 1.07E-54 | 6.19143163 | 4.04180182 |
| ANKRD45 | -2.2115782 | -1.3953181 | 15.3922281 | 8.73E-05 | 0.00055406 | 0.53776214 | 0.1240887 |
| FRAS1 | -2.2050279 | 2.31045769 | 111.963089 | 3.64E-26 | 4.02E-24 | 3.08021625 | 1.39300418 |
| NFE2L3 | -2.2037048 | 2.27550014 | 129.290171 | 5.86E-30 | 7.32E-28 | 3.04761963 | 1.36696215 |
| IFI6 | -2.1961794 | 8.64180533 | 281.208399 | 4.10E-63 | 1.25E-60 | 9.29015366 | 7.10864286 |
| ZDHHC15 | -2.1934309 | 0.09964866 | 43.2710758 | 4.77E-11 | 1.33E-09 | 1.32386818 | 0.38930864 |
| OLR1 | -2.189462 | -0.4969552 | 34.5609435 | 4.13E-09 | 7.70E-08 | 0.96224346 | 0.26683758 |
| OR52N4 | -2.185847 | -1.7372579 | 14.5117241 | 0.00013929 | 0.00083606 | 0.42220483 | 0.10089109 |
| RARB | -2.1830803 | -1.288719 | 16.3188815 | 5.35E-05 | 0.00035907 | 0.59340487 | 0.14460056 |
| HOXB-AS3 | -2.1821654 | 0.35795027 | 52.9833957 | 3.36E-13 | 1.35E-11 | 1.49771769 | 0.47530352 |
| HMCN1 | -2.1809006 | 6.75945428 | 165.49874 | 7.12E-38 | 1.15E-35 | 7.41145659 | 5.22932131 |
| ABCC9 | -2.1744494 | 2.5238338 | 131.347054 | 2.08E-30 | 2.66E-28 | 3.27777613 | 1.54044492 |
| TMEM233 | -2.1706472 | -1.4309763 | 16.6054701 | 4.60E-05 | 0.00031289 | 0.53297051 | 0.13392651 |
| ODF3B | -2.1690555 | 2.96752057 | 94.9999045 | 1.90E-22 | 1.78E-20 | 3.68738513 | 1.8184374 |
| HCP5 | -2.1501908 | 1.21678713 | 91.2662011 | 1.26E-21 | 1.12E-19 | 2.13428511 | 0.81649301 |
| KIAA0754 | -2.1466552 | 4.7591652 | 60.6758398 | 6.73E-15 | 3.34E-13 | 5.41341441 | 3.25138213 |
| EXOC3L4 | -2.144984 | -0.8665226 | 21.6701058 | 3.24E-06 | 3.02E-05 | 0.76731928 | 0.20513827 |
| FILIP1 | -2.1419004 | -1.0775207 | 22.9194497 | 1.69E-06 | 1.70E-05 | 0.68158576 | 0.17943517 |
| IBSP | -2.1367948 | 0.71951185 | 62.7313767 | 2.37E-15 | 1.26E-13 | 1.73260312 | 0.61724379 |
| GARNL3 | -2.1288465 | -0.8812685 | 25.7310275 | 3.92E-07 | 4.56E-06 | 0.76535911 | 0.20634438 |
| SLC34A3 | -2.1277185 | -0.2107053 | 25.9368332 | 3.53E-07 | 4.15E-06 | 1.09806356 | 0.33161567 |
| IFITM1 | -2.1262392 | 7.79453663 | 229.385163 | 8.12E-52 | 1.92E-49 | 8.43738038 | 6.31198388 |
| FRZB | -2.1255212 | 2.53112215 | 131.132159 | 2.32E-30 | 2.95E-28 | 3.2739589 | 1.57199418 |
| PCDHB3 | -2.1244826 | -0.795402 | 24.882299 | 6.09E-07 | 6.79E-06 | 0.80749317 | 0.22319334 |
| PLAAT2 | -2.1226725 | -1.9639792 | 10.5057731 | 0.00119002 | 0.00533253 | 0.34317783 | 0.08348146 |
| IL6 | -2.1204284 | 6.81568286 | 331.171056 | 5.34E-74 | 1.87E-71 | 7.46020798 | 5.37263194 |
| TCAF2 | -2.1183491 | 0.90438289 | 63.3763768 | 1.71E-15 | 9.22E-14 | 1.87585881 | 0.67763096 |
| FAT3 | -2.1161313 | 1.37134811 | 61.804393 | 3.79E-15 | 1.94E-13 | 2.25921786 | 0.86131321 |
| LINC02015 | -2.1090328 | -0.414058 | 33.7441296 | 6.29E-09 | 1.12E-07 | 0.99747752 | 0.29778695 |
| TNFAIP2 | -2.0963159 | 6.89695233 | 251.211704 | 1.41E-56 | 3.85E-54 | 7.52678907 | 5.47020334 |
| PNLIPRP3 | -2.0952679 | 0.31607413 | 32.7199415 | 1.06E-08 | 1.80E-07 | 1.41558672 | 0.4758616 |
| ZSCAN23 | -2.086469 | 1.45916874 | 75.7890228 | 3.16E-18 | 2.19E-16 | 2.32886719 | 0.93668114 |
| HYDIN | -2.0861652 | -0.7493763 | 17.4166915 | 3.00E-05 | 0.00021343 | 0.81037903 | 0.24045766 |
| SAMHD1 | -2.0820148 | 5.21587037 | 189.027759 | 5.18E-43 | 1.01E-40 | 5.8641858 | 3.84311909 |
| ICAM1 | -2.0782043 | 4.96419895 | 232.173686 | 2.00E-52 | 4.94E-50 | 5.61075576 | 3.63294795 |
| AKR1B15 | -2.0746338 | -0.1397712 | 37.6619771 | 8.41E-10 | 1.82E-08 | 1.16131027 | 0.36304593 |
| SLC14A2 | -2.0742189 | -1.9950884 | 9.52172501 | 0.00203054 | 0.00838575 | 0.33107823 | 0.08125505 |
| PARP14 | -2.072226 | 6.71871934 | 297.18863 | 1.35E-66 | 4.36E-64 | 7.35347734 | 5.31446815 |
| GPRASP2 | -2.071434 | -0.8354826 | 22.1526962 | 2.52E-06 | 2.41E-05 | 0.78163614 | 0.22615503 |
| SOWAHD | -2.059511 | -1.8181084 | 11.9297191 | 0.00055246 | 0.00274996 | 0.38794434 | 0.10084747 |
| PLPPR4 | -2.0582868 | 1.52223054 | 91.4827827 | 1.13E-21 | 1.01E-19 | 2.36948471 | 0.99645528 |
| HOXA6 | -2.0544322 | 0.97933448 | 52.5121111 | 4.28E-13 | 1.69E-11 | 1.92132694 | 0.7280663 |
| PALMD | -2.0496239 | 2.1420044 | 98.3650063 | 3.48E-23 | 3.43E-21 | 2.91386047 | 1.34593017 |
| ETV7 | -2.0443948 | 1.31660892 | 88.0038379 | 6.53E-21 | 5.55E-19 | 2.20241151 | 0.90442187 |
| MIA | -2.043131 | -2.0239914 | 9.31044534 | 0.00227851 | 0.00925331 | 0.33173293 | 0.07853564 |
| PILRA | -2.0429839 | -0.8526553 | 25.5447467 | 4.32E-07 | 4.97E-06 | 0.77209707 | 0.22468235 |
| SSC5D | -2.0344724 | 5.84807322 | 182.715008 | 1.24E-41 | 2.36E-39 | 6.46171966 | 4.49974226 |
| RNF224 | -2.0314245 | -1.2859217 | 14.7257018 | 0.00012434 | 0.00075592 | 0.59082421 | 0.15834645 |
| NPY2R | -2.0312023 | -0.409805 | 31.9900618 | 1.55E-08 | 2.51E-07 | 1.0003614 | 0.3109179 |
| HERC2P3 | -2.0301008 | 1.95161832 | 76.5735737 | 2.12E-18 | 1.50E-16 | 2.73647473 | 1.2368609 |
| TXLNB | -2.0298164 | 0.11788294 | 36.0137446 | 1.96E-09 | 3.94E-08 | 1.31398338 | 0.4219357 |
| BHLHE41 | -2.0239128 | 4.29624226 | 211.93043 | 5.21E-48 | 1.12E-45 | 4.94712056 | 3.06469251 |
| RNF144A | -2.0231431 | 2.65511818 | 136.061846 | 1.93E-31 | 2.59E-29 | 3.37465154 | 1.72757597 |
| EDNRB | -2.022372 | 0.78715297 | 38.2954498 | 6.08E-10 | 1.35E-08 | 1.74247689 | 0.66127963 |
| PLSCR1 | -2.019574 | 5.54324189 | 240.689765 | 2.78E-54 | 7.12E-52 | 6.17630975 | 4.21552938 |
| CCL2 | -2.0132934 | 6.63193489 | 290.202184 | 4.49E-65 | 1.39E-62 | 7.25735872 | 5.27540465 |
| C1RL-AS1 | -2.0102813 | 2.44022141 | 95.9649727 | 1.17E-22 | 1.12E-20 | 3.17419966 | 1.5642702 |
| TYMP | -2.0081644 | 5.26675998 | 171.485547 | 3.51E-39 | 6.13E-37 | 5.89623113 | 3.94816128 |
| GBP2 | -2.0028911 | 5.48243611 | 235.559558 | 3.66E-53 | 9.25E-51 | 6.11307643 | 4.1698187 |
| ABI3 | 2.04744869 | 1.05875632 | 78.7593254 | 7.02E-19 | 5.19E-17 | 0.83077785 | 2.08737405 |
| SMCO2 | 2.05873722 | -1.2204609 | 18.0621415 | 2.14E-05 | 0.00015891 | 0.19818508 | 0.67923806 |
| VGF | 2.06689909 | -2.0712092 | 10.5411533 | 0.00116746 | 0.00524543 | 0.09684925 | 0.36331354 |
| ASMTL-AS1 | 2.17194295 | -1.738896 | 15.4352815 | 8.54E-05 | 0.0005429 | 0.12488602 | 0.48095117 |
| BCL2L14 | 2.53754877 | -2.5938556 | 9.71655567 | 0.00182615 | 0.00767299 | 0.0406234 | 0.23960558 |
| KRT13 | 2.8403608 | -2.7483851 | 10.4309832 | 0.00123919 | 0.00552516 | 0.02219 | 0.20949478 |
| TREM2 | 2.94495799 | -2.6955461 | 11.3372561 | 0.00075968 | 0.00362088 | 0.02219 | 0.22334094 |
| LRRD1 | 2.95091985 | -2.6950734 | 11.2658277 | 0.00078947 | 0.00374085 | 0.02219 | 0.22473404 |
| DBET | 2.95203432 | 0.01020242 | 45.463668 | 1.55E-11 | 4.76E-10 | 0.28819778 | 1.37757111 |
| LINC00618 | 3.36063969 | -2.9174424 | 11.0007418 | 0.00091075 | 0.00423613 | 0.01379283 | 0.17894236 |
| MIR339 | 3.36783568 | -2.9183708 | 10.8456641 | 0.00099027 | 0.0045576 | 0.01122298 | 0.177516 |
| MEGF11 | 3.58718451 | -2.8007413 | 12.3423621 | 0.00044279 | 0.00227587 | 0.01560759 | 0.20634438 |
| AP1M2 | 4.2724572 | -3.3345869 | 9.32599688 | 0.00225925 | 0.00919111 | 0 | 0.10030963 |
| POU4F3 | 4.2743621 | -3.3344475 | 9.3268179 | 0.00225824 | 0.00918877 | 0 | 0.10089109 |
| SPINK2 | 4.27462925 | -3.3344271 | 9.40257174 | 0.00216681 | 0.00886658 | 0 | 0.10143726 |
| GFRA3 | 4.66569826 | -3.1818429 | 11.9118727 | 0.00055777 | 0.00277315 | 0 | 0.13018046 |
| MIR6837 | 4.67432957 | -3.1812663 | 12.1240567 | 0.00049776 | 0.00251404 | 0 | 0.13344833 |
| HYPK | 4.81979384 | -3.1112753 | 11.9045875 | 0.00055996 | 0.00278204 | 0 | 0.14093681 |

**MSC-ALL2**

|  | **logFC** | **logCPM** | **LR** | **PValue** | **FDR** | **ALL2** | **NBM** |
| --- | --- | --- | --- | --- | --- | --- | --- |
| RPS4Y1 | -12.546134 | 3.61006798 | 2242.25704 | 0 | 0 | 6.47224443 | 0.03420061 |
| CXCL6 | -12.240376 | 3.31518467 | 1542.36263 | 0 | 0 | 5.85231911 | 0.05044505 |
| CXCL8 | -11.646562 | 5.57755486 | 2562.49924 | 0 | 0 | 7.52741459 | 0.45664098 |
| CXCL1 | -10.894435 | 2.03284775 | 2028.89843 | 0 | 0 | 6.87921617 | 0.74946765 |
| C3 | -10.502776 | 4.96461853 | 1648.39235 | 0 | 0 | 7.44851783 | 2.87939506 |
| DDX3Y | -10.347368 | 1.51708181 | 1258.39298 | 1.24E-275 | 4.36E-272 | 4.99275857 | 0.2395204 |
| SERPINB2 | -9.1069744 | 2.32539459 | 1211.32241 | 2.11E-265 | 6.33E-262 | 6.46639677 | 2.55924356 |
| MMP3 | -8.9488998 | 6.63472769 | 1173.4505 | 3.59E-257 | 9.43E-254 | 5.52145522 | 1.39757679 |
| NLGN4Y | -8.6329061 | 1.87366453 | 1115.05619 | 1.76E-244 | 4.12E-241 | 4.50403278 | 0 |
| GSTM1 | -8.5236441 | 3.41316973 | 1074.65531 | 1.07E-235 | 2.24E-232 | 4.30837216 | 0.06781205 |
| SOD2 | -8.2966986 | -0.3851855 | 1068.61649 | 2.19E-234 | 4.18E-231 | 8.6930065 | 5.43943093 |
| KDM5D | -8.0127347 | 1.29347812 | 1057.40627 | 5.98E-232 | 1.05E-228 | 4.21591813 | 0 |
| STC2 | -7.8024534 | 3.6333401 | 1049.62123 | 2.94E-230 | 4.75E-227 | 10.9731157 | 8.07222457 |
| GREM1 | -7.4222038 | 5.99261654 | 1043.52084 | 6.23E-229 | 9.35E-226 | 9.26905311 | 5.79532935 |
| HSPB7 | -7.4079861 | 4.10750099 | 1003.58135 | 2.99E-220 | 4.19E-217 | 7.81082819 | 4.70630499 |
| PPL | -7.2426634 | 2.48165671 | 1001.80095 | 7.29E-220 | 9.57E-217 | 1.21481433 | 5.85521762 |
| USP9Y | -6.9148742 | 2.78610299 | 952.551655 | 3.70E-209 | 4.57E-206 | 4.51964751 | 0.1322899 |
| OLFML2A | -6.9108574 | -1.5822666 | 851.286986 | 3.82E-187 | 4.45E-184 | 3.40164006 | 6.68824748 |
| COMP | -6.7907818 | -1.6740935 | 846.510496 | 4.17E-186 | 4.61E-183 | 6.09107056 | 8.94899576 |
| LINC00968 | -6.7362709 | -1.7210714 | 795.144737 | 6.13E-175 | 6.44E-172 | 5.90510083 | 3.00505178 |
| FOSB | -6.5738459 | -1.8477771 | 756.557528 | 1.51E-166 | 1.50E-163 | 3.95994242 | 6.80490852 |
| TGM2 | -6.2351543 | -2.1069574 | 701.8102 | 1.21E-154 | 1.15E-151 | 7.18226679 | 4.70921371 |
| CD70 | -6.2346195 | -2.1072443 | 692.27683 | 1.43E-152 | 1.30E-149 | 0.97798596 | 4.02365144 |
| SFTA1P | -6.1708307 | 1.47340596 | 688.538645 | 9.29E-152 | 8.13E-149 | 1.20272273 | 4.34119809 |
| PENK | -6.1658439 | 1.90549147 | 682.385376 | 2.02E-150 | 1.70E-147 | 9.81552839 | 7.46843331 |
| EIF1AY | -6.1062177 | -2.2053468 | 676.514947 | 3.83E-149 | 3.09E-146 | 3.39646715 | 0.08291571 |
| CXCL3 | -6.099684 | -2.2056046 | 663.872407 | 2.15E-146 | 1.67E-143 | 4.03948511 | 0.85296015 |
| IGF1 | -6.0940561 | -2.2068941 | 663.592527 | 2.47E-146 | 1.85E-143 | 3.68544086 | 0.13334505 |
| ALPK2 | -6.0044449 | 1.9300114 | 655.045701 | 1.79E-144 | 1.29E-141 | 7.88867486 | 5.50784248 |
| CDH6 | -5.8825615 | -2.3504326 | 652.482004 | 6.45E-144 | 4.51E-141 | 4.66541348 | 1.76997152 |
| KCNK15 | -5.8714697 | -2.3510748 | 652.256262 | 7.22E-144 | 4.89E-141 | 1.60784752 | 4.43335803 |
| NR4A1 | -5.855498 | -2.3517613 | 635.235838 | 3.63E-140 | 2.38E-137 | 4.54890982 | 6.99583543 |
| CRISPLD2 | -5.7278466 | -2.4657827 | 634.209507 | 6.07E-140 | 3.86E-137 | 6.30752258 | 3.80408343 |
| HEPH | -5.7221926 | -2.4669054 | 605.567466 | 1.03E-133 | 6.36E-131 | 6.06671033 | 3.6487588 |
| UTY | -5.7026217 | -0.0773485 | 603.187438 | 3.39E-133 | 2.04E-130 | 2.98530962 | 0 |
| ZFY | -5.6675997 | -2.507128 | 564.699775 | 7.98E-125 | 4.66E-122 | 3.24899645 | 0.01676674 |
| ITGA7 | -5.6559198 | -2.5091612 | 564.477026 | 8.93E-125 | 5.07E-122 | 3.65220437 | 6.06736971 |
| NGEF | -5.592758 | -2.5512161 | 561.523632 | 3.92E-124 | 2.17E-121 | 1.53132303 | 4.28245329 |
| ALDH1A3 | -5.5275353 | -2.5950915 | 559.204227 | 1.25E-123 | 6.74E-121 | 6.30157888 | 3.95121103 |
| FOXQ1 | -5.5242789 | -2.5953846 | 545.507116 | 1.20E-120 | 6.27E-118 | 4.33342216 | 1.2525117 |
| DUSP5 | -5.453494 | -2.6404947 | 543.519532 | 3.23E-120 | 1.66E-117 | 3.43196791 | 5.75703075 |
| GALNT5 | -5.4491706 | -2.6415167 | 527.596943 | 9.41E-117 | 4.71E-114 | 8.16201076 | 5.45898924 |
| BIRC5 | -5.4285124 | -1.055806 | 518.323744 | 9.80E-115 | 4.79E-112 | 4.15067675 | 6.53663081 |
| MEOX2 | -5.3709785 | -2.6885642 | 516.211607 | 2.82E-114 | 1.35E-111 | 2.85582566 | 5.21918105 |
| CH25H | -5.3709785 | -2.6885642 | 509.185229 | 9.54E-113 | 4.45E-110 | 4.96927868 | 2.31556382 |
| STMN1 | -5.3687541 | -2.6886402 | 496.80113 | 4.72E-110 | 2.15E-107 | 5.54935565 | 7.66522333 |
| SFRP2 | -5.3667986 | -2.688967 | 495.346539 | 9.78E-110 | 4.37E-107 | 4.01722716 | 1.2693566 |
| LSP1 | -5.2947999 | -2.7365929 | 494.168818 | 1.76E-109 | 7.72E-107 | 3.4050552 | 5.9214316 |
| ENC1 | -5.294274 | -2.7366691 | 485.762061 | 1.19E-107 | 5.10E-105 | 8.2284456 | 6.07395854 |
| EGR2 | -5.1989118 | -2.7878432 | 474.819686 | 2.86E-105 | 1.20E-102 | 1.85015158 | 4.45274904 |
| SERPINA9 | -5.116834 | -2.8394265 | 471.794137 | 1.30E-104 | 5.37E-102 | 3.19778227 | 0.44239516 |
| PRKY | -5.1162047 | -2.8393169 | 466.447186 | 1.90E-103 | 7.67E-101 | 2.87140513 | 0.12918928 |
| ACTC1 | -5.1162047 | -2.8393169 | 458.553094 | 9.93E-102 | 3.93E-99 | 3.98898201 | 1.56235473 |
| KRT14 | -5.1047399 | 0.49436764 | 456.330979 | 3.02E-101 | 1.18E-98 | 2.8598859 | 5.74820899 |
| MKI67 | -5.0256954 | -2.8928008 | 454.742877 | 6.70E-101 | 2.56E-98 | 4.71219036 | 6.7746971 |
| F2R | -5.0256807 | -2.8926909 | 453.997199 | 9.73E-101 | 3.65E-98 | 5.0042127 | 7.53637878 |
| TTTY15 | -5.0239773 | -2.8931306 | 449.863503 | 7.72E-100 | 2.85E-97 | 2.51691466 | 0 |
| CDC20 | -5.0229681 | -2.8932068 | 449.404501 | 9.72E-100 | 3.52E-97 | 3.56072265 | 5.99673833 |
| TFPI2 | -5.0217396 | -2.8933167 | 443.344383 | 2.03E-98 | 7.21E-96 | 6.85861876 | 4.75957749 |
| PAPPA | -5.0189515 | -2.8937226 | 435.640249 | 9.62E-97 | 3.31E-94 | 8.87512318 | 5.51085572 |
| PLK1 | -5.0096516 | -2.8942382 | 435.268846 | 1.16E-96 | 3.93E-94 | 3.32229155 | 5.68276825 |
| PGF | -4.946776 | -2.9477555 | 430.940021 | 1.01E-95 | 3.38E-93 | 5.16599079 | 2.33518949 |
| TNFAIP6 | -4.9252778 | -2.9489765 | 429.865532 | 1.74E-95 | 5.70E-93 | 5.02612714 | 2.82741823 |
| DMKN | -4.9204192 | -2.9493071 | 428.3725 | 3.67E-95 | 1.19E-92 | 3.55841202 | 6.06286295 |
| TXLNGY | -4.9185591 | -2.9496036 | 426.030278 | 1.19E-94 | 3.72E-92 | 2.83335165 | 0.01676674 |
| CYP1B1 | -4.9114729 | -2.9499001 | 422.816711 | 5.95E-94 | 1.84E-91 | 10.1270294 | 7.54909344 |
| MRVI1 | -4.903331 | -2.9506369 | 407.540928 | 1.26E-90 | 3.77E-88 | 4.27757476 | 1.39585429 |
| CDKN3 | -4.8601963 | -0.8218551 | 404.828226 | 4.90E-90 | 1.45E-87 | 2.55240782 | 4.58545554 |
| AHRR | -4.8414213 | -3.0064927 | 403.278903 | 1.06E-89 | 3.10E-87 | 6.265064 | 4.30327873 |
| PODN | -4.8225405 | -3.0069005 | 395.951265 | 4.19E-88 | 1.21E-85 | 3.89339374 | 6.07019423 |
| IQGAP3 | -4.8155646 | -1.5603815 | 395.659107 | 4.85E-88 | 1.38E-85 | 3.08422695 | 5.35035346 |
| HJURP | -4.8152953 | -1.5602189 | 391.976093 | 3.07E-87 | 8.61E-85 | 2.7135176 | 4.69669685 |
| MYBL2 | -4.8095049 | -3.0078263 | 390.516241 | 6.39E-87 | 1.77E-84 | 2.37214325 | 4.6824435 |
| CCKAR | -4.8045673 | 4.67502048 | 390.1433 | 7.70E-87 | 2.10E-84 | 2.32335626 | 0.01743386 |
| GTSE1 | -4.8041988 | -0.4052921 | 387.146902 | 3.46E-86 | 9.31E-84 | 3.02750799 | 5.18252211 |
| KIFC1 | -4.8037144 | -3.0086411 | 386.621726 | 4.50E-86 | 1.19E-83 | 3.09925174 | 5.25173845 |
| LDLRAD4 | -4.7951307 | 6.60129465 | 386.610835 | 4.53E-86 | 1.19E-83 | 3.40743361 | 0.86235984 |
| UBE2C | -4.7930377 | -3.008938 | 384.676625 | 1.19E-85 | 3.09E-83 | 2.94910449 | 5.24709371 |
| CD200 | -4.7759446 | -0.8972934 | 383.840896 | 1.81E-85 | 4.65E-83 | 2.84792005 | 0.35641961 |
| KRT81 | -4.7691987 | -1.5835938 | 383.448416 | 2.21E-85 | 5.59E-83 | 4.15032303 | 1.38235508 |
| PKMYT1 | -4.7367533 | -1.6248964 | 380.499411 | 9.69E-85 | 2.42E-82 | 1.94770652 | 4.13573861 |
| TROAP | -4.6872925 | -3.0691488 | 379.854467 | 1.34E-84 | 3.31E-82 | 2.15972728 | 4.24778462 |
| ASF1B | -4.6750588 | -3.0698887 | 379.099502 | 1.95E-84 | 4.77E-82 | 2.30649289 | 4.44498675 |
| C1QTNF1 | -4.6736276 | -3.0701861 | 377.883317 | 3.60E-84 | 8.68E-82 | 5.02777574 | 3.02375167 |
| CXCL5 | -4.6334383 | 1.66136739 | 375.673221 | 1.09E-83 | 2.60E-81 | 2.84415176 | 0.11661472 |
| ATF3 | -4.5565262 | -3.132792 | 370.533631 | 1.43E-82 | 3.30E-80 | 2.12067484 | 4.32569064 |
| NLGN4X | -4.5466095 | -3.1337207 | 367.770983 | 5.72E-82 | 1.31E-79 | 3.52947679 | 0.96979329 |
| MN1 | -4.5456491 | -3.1338315 | 364.832501 | 2.50E-81 | 5.64E-79 | 6.71571302 | 4.56754057 |
| CFB | -4.54124 | -3.1339423 | 359.523659 | 3.58E-80 | 7.99E-78 | 5.88374904 | 3.96467725 |
| TACC3 | -4.5072497 | 2.3121618 | 358.765584 | 5.23E-80 | 1.16E-77 | 4.0118917 | 6.0793805 |
| TDRD12 | -4.4679379 | 0.14656509 | 355.940043 | 2.16E-79 | 4.72E-77 | 2.47040721 | 0.08358283 |
| CCNB2 | -4.4278003 | -3.1987089 | 349.568089 | 5.26E-78 | 1.13E-75 | 3.26868754 | 5.23512186 |
| SPC24 | -4.4267237 | -3.1985977 | 346.998023 | 1.91E-77 | 4.01E-75 | 1.992772 | 4.3884147 |
| MGAM | -4.4240185 | 1.93381943 | 346.834049 | 2.07E-77 | 4.31E-75 | 2.8022764 | 0.46661208 |
| TIPARP | -4.4167242 | -3.1993063 | 345.881727 | 3.34E-77 | 6.88E-75 | 6.07286592 | 4.04578964 |
| KIF2C | -4.4167242 | -3.1993063 | 345.103281 | 4.94E-77 | 1.01E-74 | 3.33959731 | 5.25719689 |
| SLC16A6 | -4.4131508 | -3.1994175 | 341.953528 | 2.40E-76 | 4.79E-74 | 3.60134689 | 1.43548326 |
| HOTAIR | -4.4112612 | -3.1996398 | 339.480364 | 8.28E-76 | 1.62E-73 | 3.57125869 | 1.00424947 |
| CDCA3 | -4.4103017 | -3.1997509 | 338.37099 | 1.44E-75 | 2.81E-73 | 2.61524127 | 4.72658072 |
| IRAK3 | -4.4089772 | -3.1999384 | 337.202645 | 2.59E-75 | 5.00E-73 | 3.96355037 | 1.96338222 |
| NTNG1 | -4.4082334 | -3.2000495 | 332.612853 | 2.59E-74 | 4.82E-72 | 1.62590936 | 3.63615366 |
| NGFR | -4.4082334 | -3.2000495 | 331.584855 | 4.34E-74 | 7.93E-72 | 2.4536832 | 4.52559127 |
| F3 | -4.4060613 | -3.2001606 | 329.410494 | 1.29E-73 | 2.32E-71 | 2.60927647 | 0.26542828 |
| XYLT1 | -4.4031412 | -3.2004591 | 328.014554 | 2.60E-73 | 4.63E-71 | 8.53323172 | 6.08985288 |
| PRDM1 | -4.3476139 | -1.9266146 | 326.579566 | 5.34E-73 | 9.43E-71 | 3.56474969 | 1.3046568 |
| FAM43A | -4.3408435 | 0.23164621 | 324.854956 | 1.27E-72 | 2.20E-70 | 5.6083773 | 3.65839925 |
| ADAM12 | -4.3318798 | -1.9531301 | 324.59124 | 1.45E-72 | 2.49E-70 | 9.52596959 | 7.32154277 |
| PDGFRL | -4.28588 | -1.3036941 | 323.350845 | 2.70E-72 | 4.61E-70 | 2.32450912 | 4.30683087 |
| SAPCD2 | -4.2745508 | 3.20063339 | 321.028034 | 8.65E-72 | 1.46E-69 | 2.03211769 | 3.94864424 |
| EPHA5 | -4.2674762 | -1.9844704 | 319.941262 | 1.49E-71 | 2.50E-69 | 4.65060178 | 2.2692568 |
| ANGPT1 | -4.2283069 | -0.2943074 | 317.426778 | 5.27E-71 | 8.57E-69 | 5.7792186 | 3.42627718 |
| IFI27 | -4.1807937 | 5.64519125 | 315.582003 | 1.33E-70 | 2.15E-68 | 4.56319933 | 2.64135418 |
| MMP1 | -4.1298152 | -2.0768592 | 312.9264 | 5.03E-70 | 8.00E-68 | 5.43752398 | 3.52700266 |
| MGP | -4.0608967 | -1.0422336 | 312.372032 | 6.65E-70 | 1.05E-67 | 4.08364879 | 6.07486792 |
| KRT7 | -4.0046612 | -2.1718174 | 311.207047 | 1.19E-69 | 1.87E-67 | 7.22486884 | 4.98406739 |
| LMNB1 | -3.9913376 | -2.2345576 | 308.435047 | 4.79E-69 | 7.34E-67 | 3.0294425 | 5.00295472 |
| PTTG1 | -3.9592263 | 1.90301315 | 307.774189 | 6.67E-69 | 1.01E-66 | 3.64480185 | 5.60276896 |
| AURKB | -3.9580389 | -2.2048675 | 306.452186 | 1.29E-68 | 1.96E-66 | 2.19424389 | 4.35831169 |
| CDCA5 | -3.9374138 | -0.5581513 | 300.031586 | 3.24E-67 | 4.70E-65 | 2.86327496 | 4.74764392 |
| GAP43 | -3.9230432 | -2.2386337 | 298.793218 | 6.04E-67 | 8.68E-65 | 4.12019167 | 2.31811549 |
| ERV3-1 | -3.8771235 | -0.3734445 | 295.644885 | 2.93E-66 | 4.18E-64 | 3.8193013 | 1.81176574 |
| SLC22A3 | -3.8569199 | -1.6473676 | 295.069932 | 3.91E-66 | 5.54E-64 | 3.93172031 | 1.87655354 |
| FOXF2 | -3.8390582 | 1.34816431 | 294.467775 | 5.29E-66 | 7.45E-64 | 4.28969585 | 2.44994736 |
| CDCA8 | -3.8084185 | 3.52738237 | 293.840929 | 7.24E-66 | 1.01E-63 | 2.56322133 | 4.54571487 |
| TEKT4P2 | -3.8025372 | -2.3109506 | 293.015206 | 1.10E-65 | 1.52E-63 | 2.96890477 | 0.81703692 |
| DNER | -3.7954984 | -1.6945826 | 291.639683 | 2.18E-65 | 3.02E-63 | 3.29846128 | 1.27321026 |
| CYP7B1 | -3.7873856 | 1.7054939 | 290.84308 | 3.26E-65 | 4.47E-63 | 2.61487512 | 0.46020761 |
| CXCL2 | -3.7847076 | -1.7160973 | 289.537892 | 6.27E-65 | 8.55E-63 | 3.82642936 | 1.92642276 |
| STARD4-AS1 | -3.7542755 | -0.1188419 | 289.329977 | 6.96E-65 | 9.43E-63 | 4.17396902 | 2.01189416 |
| ANOS1 | -3.7539466 | -2.3472388 | 287.039326 | 2.20E-64 | 2.94E-62 | 3.37718438 | 1.27487596 |
| STC1 | -3.7461621 | -2.3485756 | 286.773039 | 2.51E-64 | 3.34E-62 | 4.02617056 | 1.92955246 |
| MTSS1 | -3.7395905 | -0.9809679 | 284.064288 | 9.77E-64 | 1.29E-61 | 2.04754899 | 4.00191211 |
| SYNPO2 | -3.7371121 | -2.3497987 | 279.581135 | 9.27E-63 | 1.21E-60 | 5.38982861 | 3.27054672 |
| CPM | -3.7310364 | -1.7429779 | 276.740628 | 3.85E-62 | 4.99E-60 | 3.95489328 | 1.99386254 |
| EGR3 | -3.7309888 | -2.3494719 | 275.007079 | 9.20E-62 | 1.18E-59 | 2.76272153 | 4.82301886 |
| CRYBB2P1 | -3.7008071 | -1.7677514 | 274.009984 | 1.52E-61 | 1.94E-59 | 3.32376862 | 5.3206763 |
| FAM83D | -3.6647824 | -1.7931831 | 273.453452 | 2.01E-61 | 2.54E-59 | 2.22620446 | 4.05918409 |
| KIF18B | -3.6377269 | -1.0572834 | 269.49324 | 1.46E-60 | 1.83E-58 | 2.27854433 | 4.12699177 |
| GALNT16 | -3.6274135 | -1.0713471 | 267.687272 | 3.62E-60 | 4.47E-58 | 1.65624143 | 3.6398231 |
| TNFAIP8L1 | -3.6086323 | -0.8190783 | 267.310946 | 4.38E-60 | 5.37E-58 | 1.30169956 | 3.18992533 |
| ZNF385D | -3.6041837 | -1.4159431 | 264.28524 | 2.00E-59 | 2.41E-57 | 2.75187656 | 4.73033938 |
| TRH | -3.5960978 | -0.8406052 | 263.635611 | 2.77E-59 | 3.32E-57 | 0.16644442 | 1.9719831 |
| TMEM158 | -3.5766505 | -2.465253 | 262.497235 | 4.90E-59 | 5.81E-57 | 3.84170203 | 6.24464143 |
| DDX11 | -3.572811 | 0.14126984 | 260.795037 | 1.15E-58 | 1.36E-56 | 2.43085847 | 4.27020371 |
| CNTNAP2 | -3.5601678 | -2.4671432 | 259.197974 | 2.57E-58 | 2.99E-56 | 3.73177523 | 1.88100562 |
| WDR62 | -3.5488507 | 2.58289443 | 255.2841 | 1.83E-57 | 2.12E-55 | 2.39593197 | 4.23686229 |
| ABCC9 | -3.5396595 | -0.8833176 | 254.126265 | 3.27E-57 | 3.78E-55 | 3.5906923 | 1.54044492 |
| PIMREG | -3.5296591 | 2.04411039 | 253.266973 | 5.04E-57 | 5.78E-55 | 2.76974795 | 4.70653663 |
| DLX2 | -3.5271101 | -0.671701 | 253.206079 | 5.19E-57 | 5.93E-55 | 3.17610714 | 1.26811227 |
| KCTD12 | -3.5234837 | -0.4867503 | 252.335291 | 8.04E-57 | 9.13E-55 | 9.03729602 | 6.69706643 |
| SKA1 | -3.5224577 | 8.48938501 | 251.029352 | 1.55E-56 | 1.74E-54 | 1.80911431 | 3.75584415 |
| LINC01423 | -3.5216091 | -0.1601398 | 249.658972 | 3.08E-56 | 3.44E-54 | 1.62560784 | 3.88471109 |
| EPGN | -3.5058267 | 0.79579057 | 245.066356 | 3.09E-55 | 3.33E-53 | 4.07371755 | 1.93955369 |
| IL32 | -3.499606 | -2.507907 | 244.62677 | 3.85E-55 | 4.11E-53 | 2.39515776 | 0.48059631 |
| LYPD1 | -3.4951559 | 3.48787328 | 241.511238 | 1.84E-54 | 1.92E-52 | 2.30689253 | 0.35421239 |
| F2RL2 | -3.484964 | 1.24857667 | 239.173465 | 5.96E-54 | 6.16E-52 | 3.18322925 | 5.20540339 |
| APOE | -3.4833091 | -2.5086677 | 239.146823 | 6.04E-54 | 6.21E-52 | 1.7434888 | 4.21664683 |
| FIBCD1 | -3.4805281 | -0.5201164 | 238.425092 | 8.67E-54 | 8.84E-52 | 3.67689889 | 1.76868348 |
| ROS1 | -3.4538154 | 0.03057319 | 234.898413 | 5.09E-53 | 5.14E-51 | 2.47700981 | 0.62440636 |
| CCDC85A | -3.4481147 | 2.76805544 | 234.202389 | 7.23E-53 | 7.26E-51 | 2.90006132 | 0.62654573 |
| MXD3 | -3.4479217 | 2.71706676 | 231.022101 | 3.57E-52 | 3.53E-50 | 2.17230689 | 4.00648422 |
| TMEM178B | -3.4350159 | 3.22360836 | 230.332031 | 5.05E-52 | 4.97E-50 | 3.01884887 | 1.0672931 |
| CENPA | -3.42899 | 1.46993963 | 225.879903 | 4.72E-51 | 4.59E-49 | 1.65963464 | 3.45895464 |
| SECTM1 | -3.4110541 | -0.4114783 | 225.034665 | 7.22E-51 | 6.95E-49 | 4.18012486 | 2.40156381 |
| ESPNL | -3.3908039 | 8.10615974 | 224.932886 | 7.59E-51 | 7.28E-49 | 3.66166051 | 1.6212731 |
| MROCKI | -3.3727174 | 1.29138864 | 223.480888 | 1.57E-50 | 1.49E-48 | 2.39239716 | 0.54612455 |
| NDNF | -3.3707726 | 0.07163006 | 223.297114 | 1.73E-50 | 1.63E-48 | 4.60025864 | 2.68082583 |
| PARM1 | -3.3659222 | -1.6019886 | 219.688858 | 1.06E-49 | 9.74E-48 | 2.20941841 | 0.40333904 |
| STMN3 | -3.3657078 | -2.5933904 | 215.717378 | 7.77E-49 | 7.00E-47 | 2.08805966 | 3.88745577 |
| IBSP | -3.3630966 | 3.89053563 | 214.652469 | 1.33E-48 | 1.19E-46 | 2.42608777 | 0.61724379 |
| SLC7A14 | -3.355141 | 3.36541896 | 212.065383 | 4.87E-48 | 4.31E-46 | 3.28735135 | 1.66987935 |
| MBP | -3.3532403 | -2.5953234 | 210.105823 | 1.30E-47 | 1.13E-45 | 0.9101188 | 2.59333785 |
| KIT | -3.3303601 | 0.32928114 | 205.749087 | 1.16E-46 | 9.84E-45 | 2.39661756 | 0.56872693 |
| EBF2 | -3.3175398 | -1.3048597 | 205.312984 | 1.45E-46 | 1.22E-44 | 1.8410403 | 3.57328091 |
| HRH2 | -3.3123163 | 7.931675 | 205.218646 | 1.52E-46 | 1.27E-44 | 0.32131783 | 2.11304714 |
| NRCAM | -3.2871417 | -2.6393825 | 200.756511 | 1.43E-45 | 1.17E-43 | 2.34646217 | 0.63733808 |
| EDNRB | -3.273221 | -2.0752845 | 199.160821 | 3.18E-45 | 2.58E-43 | 2.44719215 | 0.66127963 |
| GATD3A | -3.2646571 | -2.6414957 | 199.152525 | 3.20E-45 | 2.58E-43 | 2.71722611 | 1.08812212 |
| ABCA1 | -3.2628587 | -0.8938678 | 195.80059 | 1.72E-44 | 1.36E-42 | 6.68922619 | 4.28909381 |
| CDT1 | -3.2535223 | -1.6730056 | 193.79175 | 4.73E-44 | 3.65E-42 | 1.97461705 | 4.03726133 |
| NAMPT | -3.2515542 | -2.103465 | 193.573169 | 5.28E-44 | 4.06E-42 | 6.25973362 | 4.22891995 |
| KY | -3.2242901 | -2.1068313 | 189.824997 | 3.47E-43 | 2.61E-41 | 3.07755894 | 1.4886931 |
| INHBA | -3.223028 | 1.4810238 | 188.709417 | 6.08E-43 | 4.56E-41 | 8.0145073 | 5.46366534 |
| ADGRG2 | -3.2052141 | -2.6866401 | 187.980486 | 8.77E-43 | 6.51E-41 | 1.26589458 | 2.9874872 |
| C1QTNF3 | -3.1924776 | -2.6875556 | 186.60952 | 1.75E-42 | 1.29E-40 | 1.07933967 | 2.70693791 |
| EFHD1 | -3.1865573 | 2.14059005 | 185.774814 | 2.66E-42 | 1.95E-40 | 1.16923588 | 2.82223999 |
| CHRDL2 | -3.181895 | 7.061297 | 185.668942 | 2.80E-42 | 2.05E-40 | 0.31096843 | 1.8959025 |
| VSNL1 | -3.1650097 | 1.50145232 | 184.922095 | 4.08E-42 | 2.97E-40 | 0.47885482 | 2.26071304 |
| GINS2 | -3.1575691 | -1.7443272 | 181.716632 | 2.04E-41 | 1.44E-39 | 1.761111 | 3.76393061 |
| P2RY2 | -3.1398033 | -2.1699946 | 181.310609 | 2.51E-41 | 1.76E-39 | 1.08333434 | 2.79029531 |
| CENPM | -3.1237093 | -2.1724076 | 177.538565 | 1.67E-40 | 1.14E-38 | 1.71329346 | 3.67657107 |
| E2F2 | -3.1131732 | 4.42071487 | 173.545821 | 1.24E-39 | 8.26E-38 | 0.58293363 | 2.10421404 |
| CHRM3 | -3.1124034 | -1.7917162 | 172.39973 | 2.21E-39 | 1.45E-37 | 0.69038229 | 2.98553772 |
| SAXO2 | -3.0970085 | 5.16208467 | 168.97791 | 1.24E-38 | 7.94E-37 | 1.85083075 | 0.29692093 |
| GRB14 | -3.0845634 | 1.5915835 | 168.412678 | 1.64E-38 | 1.05E-36 | 2.11476972 | 0.58288628 |
| MPPED2 | -3.0845064 | -1.037749 | 167.451013 | 2.67E-38 | 1.66E-36 | 2.66090086 | 0.99264202 |
| CCL5 | -3.0843914 | 0.28182279 | 167.085771 | 3.20E-38 | 1.98E-36 | 2.61464575 | 0.97361115 |
| HMCN1 | -3.0581375 | 1.53776594 | 166.867978 | 3.57E-38 | 2.21E-36 | 7.47336863 | 5.22932131 |
| DDIT4L | -3.0417066 | -0.1065118 | 165.141488 | 8.52E-38 | 5.20E-36 | 4.44596751 | 2.6081105 |
| COL22A1 | -3.0415228 | -1.0687333 | 163.189138 | 2.27E-37 | 1.36E-35 | 1.65234223 | 0.08523546 |
| RSPO2 | -2.9789806 | -0.2568621 | 161.66388 | 4.90E-37 | 2.92E-35 | 1.0608914 | 2.70064556 |
| HAS3 | -2.9643409 | 4.2396063 | 160.42033 | 9.16E-37 | 5.40E-35 | 2.73767996 | 1.21054378 |
| KRT80 | -2.9604203 | -0.50779 | 156.317258 | 7.22E-36 | 4.10E-34 | 2.35737464 | 0.84399213 |
| SPTBN5 | -2.9571339 | 3.23631578 | 151.530152 | 8.03E-35 | 4.37E-33 | 0.98084299 | 2.40598502 |
| ATP13A3 | -2.9448232 | -1.9001206 | 151.15279 | 9.70E-35 | 5.22E-33 | 8.26805112 | 6.10913067 |
| PPARGC1A | -2.9380847 | 0.37466626 | 150.082943 | 1.66E-34 | 8.84E-33 | 2.64286761 | 1.19280611 |
| OSR2 | -2.9354393 | 10.2499414 | 145.591359 | 1.59E-33 | 8.13E-32 | 0.35378594 | 1.87417281 |
| NCKAP5 | -2.9350447 | -2.3094466 | 144.21155 | 3.19E-33 | 1.62E-31 | 2.69613768 | 1.13330227 |
| ZIC1 | -2.9309154 | -2.8381571 | 142.860903 | 6.30E-33 | 3.15E-31 | 1.45798337 | 0.11561399 |
| ANKRD20A5P | -2.9253968 | 1.57224702 | 140.017932 | 2.64E-32 | 1.28E-30 | 1.40716626 | 0.10028707 |
| RSAD2 | -2.9197202 | -0.9806454 | 139.718031 | 3.07E-32 | 1.48E-30 | 2.73891212 | 0.92736046 |
| SHOX2 | -2.9112816 | -1.6441648 | 139.598575 | 3.26E-32 | 1.56E-30 | 0.87448641 | 2.71864207 |
| GJB2 | -2.8997161 | 2.80379196 | 138.639594 | 5.28E-32 | 2.49E-30 | 0.15078425 | 1.4498354 |
| TAC3 | -2.8936004 | -2.3461665 | 138.190522 | 6.62E-32 | 3.11E-30 | 1.27433678 | 0.03425775 |
| NEURL1B | -2.8924993 | 1.45575011 | 137.811811 | 8.01E-32 | 3.75E-30 | 1.57327887 | 3.18808411 |
| OASL | -2.8726887 | -2.3468388 | 137.535498 | 9.21E-32 | 4.30E-30 | 2.46475313 | 0.94755616 |
| KCTD4 | -2.8654608 | -2.3481865 | 137.025371 | 1.19E-31 | 5.52E-30 | 0.97791347 | 2.40039297 |
| TTTY14 | -2.8591325 | -2.3487671 | 131.713814 | 1.73E-30 | 7.77E-29 | 1.099583 | 0 |
| CDKL5 | -2.8543254 | -2.3484455 | 131.132614 | 2.32E-30 | 1.04E-28 | 2.29550054 | 0.72136899 |
| E2F8 | -2.8529132 | -1.9820579 | 130.952725 | 2.54E-30 | 1.13E-28 | 0.69706769 | 2.05042015 |
| BMS1P2 | -2.8519006 | 5.38417286 | 130.795453 | 2.74E-30 | 1.22E-28 | 2.5194338 | 0.90022182 |
| MMP8 | -2.8431545 | -0.8809434 | 129.888947 | 4.33E-30 | 1.90E-28 | 2.53481946 | 1.05715528 |
| PNLDC1 | -2.8277375 | -0.5024461 | 126.759073 | 2.10E-29 | 8.83E-28 | 0.05523664 | 1.16112676 |
| GRK3 | -2.8161687 | 0.05969417 | 124.035837 | 8.27E-29 | 3.40E-27 | 2.73003462 | 1.26152684 |
| ERG | -2.8091014 | 4.30765123 | 122.931066 | 1.44E-28 | 5.90E-27 | 0.26368466 | 1.92241987 |
| ASPN | -2.7895097 | -0.9231191 | 121.90952 | 2.42E-28 | 9.77E-27 | 1.22315197 | 3.16342119 |
| FRMPD3 | -2.7851352 | -0.5287287 | 118.220029 | 1.55E-27 | 6.03E-26 | 2.27676682 | 0.73478618 |
| COL25A1 | -2.7645489 | 1.19054561 | 116.570289 | 3.57E-27 | 1.36E-25 | 2.22759501 | 0.42305141 |
| PDE3A | -2.7590418 | -1.7429803 | 114.036256 | 1.28E-26 | 4.74E-25 | 4.54727428 | 2.63670633 |
| HTR2A | -2.7575903 | -2.0429539 | 112.917882 | 2.25E-26 | 8.23E-25 | 1.76747932 | 0.44482022 |
| POMK | -2.7552993 | 0.76252574 | 112.575923 | 2.67E-26 | 9.72E-25 | 2.07869172 | 0.7569343 |
| MMP28 | -2.7520128 | -1.1139508 | 111.837463 | 3.88E-26 | 1.40E-24 | 0.92972184 | 2.25455374 |
| HCP5 | -2.7501478 | 7.46127637 | 111.381402 | 4.88E-26 | 1.76E-24 | 2.20057821 | 0.81649301 |
| PCLO | -2.7473224 | -2.4248158 | 111.12306 | 5.56E-26 | 1.99E-24 | 2.11249204 | 0.73704741 |
| REL | -2.7440413 | -1.7643616 | 110.2806 | 8.51E-26 | 3.02E-24 | 2.80077577 | 1.21736834 |
| DHRS9 | -2.7378453 | -1.3012497 | 108.314654 | 2.29E-25 | 8.04E-24 | 1.87085393 | 0.55236841 |
| CLEC12A | -2.7327539 | -2.4265192 | 108.216756 | 2.41E-25 | 8.40E-24 | 0 | 1.11905401 |
| GCH1 | -2.731353 | 2.85713897 | 106.755151 | 5.04E-25 | 1.73E-23 | 2.24298092 | 0.79397221 |
| ISLR2 | -2.7296813 | 2.620172 | 106.742784 | 5.07E-25 | 1.74E-23 | 0.46498541 | 1.68511581 |
| STOX2 | -2.7277381 | -0.1788952 | 106.641943 | 5.33E-25 | 1.82E-23 | 1.50322787 | 0.31068229 |
| ANO3 | -2.7130205 | -1.7689822 | 106.451762 | 5.87E-25 | 1.99E-23 | 1.49326493 | 0.23422408 |
| SLC7A2 | -2.7108573 | -2.0737872 | 105.709476 | 8.54E-25 | 2.86E-23 | 1.45622944 | 0.26268989 |
| LRRC4 | -2.7067695 | -1.5361682 | 102.930169 | 3.47E-24 | 1.12E-22 | 2.08191946 | 0.69673303 |
| SLC24A3 | -2.6983172 | 2.23763942 | 101.806783 | 6.12E-24 | 1.97E-22 | 1.89678627 | 0.62661484 |
| SAP30L-AS1 | -2.6793351 | -1.7936349 | 101.165402 | 8.46E-24 | 2.71E-22 | 1.95159806 | 0.73472636 |
| IDI2-AS1 | -2.6782026 | -0.7396828 | 100.524133 | 1.17E-23 | 3.72E-22 | 0.63838276 | 1.8976055 |
| KANK4 | -2.6750487 | -0.3853938 | 98.4173323 | 3.39E-23 | 1.04E-21 | 1.32582081 | 0.18903393 |
| STAB1 | -2.6738116 | -1.3556829 | 98.3513188 | 3.50E-23 | 1.08E-21 | 0.26515502 | 1.45952785 |
| APOBEC3B | -2.6717469 | 3.94792676 | 97.716292 | 4.83E-23 | 1.47E-21 | 1.0603506 | 0.05051291 |
| GSTM5 | -2.6714514 | -1.3557507 | 97.4129042 | 5.63E-23 | 1.71E-21 | 1.11942738 | 0.07853564 |
| FAM43B | -2.6689651 | 0.21772785 | 96.1954623 | 1.04E-22 | 3.10E-21 | 0.29948596 | 1.65935495 |
| CST6 | -2.6608905 | 1.95057851 | 95.5817788 | 1.42E-22 | 4.20E-21 | 0.7916328 | 2.13993266 |
| BEND3P3 | -2.6603855 | -2.4671609 | 95.2600399 | 1.67E-22 | 4.87E-21 | 2.97904131 | 1.10455219 |
| TINAGL1 | -2.6541406 | 2.54505246 | 95.0710031 | 1.84E-22 | 5.35E-21 | 2.04929246 | 0.75644497 |
| L1CAM | -2.6529504 | -1.5782802 | 94.2004285 | 2.85E-22 | 8.18E-21 | 1.21638792 | 0.12411292 |
| MAOB | -2.6498621 | -0.2444359 | 93.159163 | 4.83E-22 | 1.37E-20 | 1.30129491 | 0.17894236 |
| BEX1 | -2.6483863 | 1.70648061 | 92.2764473 | 7.54E-22 | 2.12E-20 | 1.06878206 | 0.09915515 |
| ANO1 | -2.6460387 | -1.2086142 | 91.8054491 | 9.56E-22 | 2.65E-20 | 0.27452927 | 1.30364679 |
| GAL | -2.6360183 | -0.4378667 | 91.7749548 | 9.71E-22 | 2.69E-20 | 1.64947222 | 0.45050334 |
| SDK1 | -2.63087 | -0.0140799 | 90.614989 | 1.75E-21 | 4.77E-20 | 1.78257072 | 0.53594962 |
| PSORS1C1 | -2.6238176 | -1.6003907 | 88.4209352 | 5.29E-21 | 1.40E-19 | 0.62587927 | 1.74302453 |
| IL4I1 | -2.6219426 | 5.62191824 | 87.4242909 | 8.76E-21 | 2.28E-19 | 1.7230248 | 0.61517672 |
| FOXS1 | -2.6171693 | -2.5058981 | 87.3257848 | 9.20E-21 | 2.39E-19 | 0.10090424 | 1.0351494 |
| GUCY1A2 | -2.6156185 | -0.2587876 | 87.2497417 | 9.56E-21 | 2.48E-19 | 2.51274549 | 0.89832867 |
| LERFS | -2.612851 | -2.5062038 | 87.0045915 | 1.08E-20 | 2.79E-19 | 0.05623709 | 0.9535879 |
| SHISA2 | -2.6100357 | 9.45069958 | 86.8471735 | 1.17E-20 | 3.02E-19 | 2.33674513 | 0.90184497 |
| GPT | -2.6074655 | -2.5078532 | 84.0371257 | 4.86E-20 | 1.18E-18 | 1.55486158 | 0.48406004 |
| KIAA0754 | -2.6021618 | 1.42643105 | 81.5138993 | 1.74E-19 | 4.05E-18 | 6.1000521 | 3.25138213 |
| WNT2 | -2.5790029 | -1.2465432 | 80.4095503 | 3.04E-19 | 6.98E-18 | 0.45737048 | 1.57889655 |
| CDCA7 | -2.5750982 | -1.2607873 | 79.317455 | 5.29E-19 | 1.20E-17 | 0.52511817 | 1.62973999 |
| PWAR5 | -2.5646372 | 1.42313574 | 79.0567841 | 6.04E-19 | 1.36E-17 | 1.36362095 | 0.17469186 |
| PTCH2 | -2.5574282 | 7.33986141 | 78.9951202 | 6.23E-19 | 1.40E-17 | 0.53480137 | 1.55743096 |
| ANKRD36C | -2.5508156 | -1.8962209 | 78.5245014 | 7.90E-19 | 1.76E-17 | 2.00361373 | 0.70155297 |
| NPFF | -2.5484288 | 6.50804844 | 77.4790639 | 1.34E-18 | 2.93E-17 | 0.45279599 | 1.64673884 |
| APCDD1 | -2.5463718 | 5.39026702 | 77.3039815 | 1.47E-18 | 3.20E-17 | 1.59261146 | 0.54985303 |
| ADAMDEC1 | -2.5450874 | -0.8417612 | 75.9175946 | 2.96E-18 | 6.31E-17 | 1.0281276 | 0.13010453 |
| KRT86 | -2.5450027 | 2.87025599 | 75.7776383 | 3.17E-18 | 6.75E-17 | 1.53575351 | 0.46835155 |
| ADH1B | -2.5440093 | -1.897274 | 75.6739871 | 3.35E-18 | 7.07E-17 | 0.33903696 | 1.51983884 |
| CCL8 | -2.5343147 | -2.5505956 | 74.6701502 | 5.56E-18 | 1.16E-16 | 0.9724396 | 0.11652116 |
| DCLK3 | -2.5324377 | 2.43091144 | 74.665851 | 5.58E-18 | 1.16E-16 | 0.83598495 | 0.03420061 |
| RARB | -2.5302726 | -0.5094406 | 73.3804537 | 1.07E-17 | 2.17E-16 | 1.17017848 | 0.14460056 |
| SMOC2 | -2.5266931 | -2.5511731 | 73.3303432 | 1.10E-17 | 2.22E-16 | 0.11536094 | 0.922421 |
| TBC1D3L | -2.5231476 | 0.60917427 | 73.2679176 | 1.13E-17 | 2.29E-16 | 0.55358168 | 1.71039632 |
| LINC00944 | -2.5196464 | 0.91885834 | 71.5838624 | 2.66E-17 | 5.20E-16 | 1.288536 | 0.25468849 |
| CCDC144B | -2.5141036 | -0.9942344 | 70.9388604 | 3.68E-17 | 7.12E-16 | 2.53863331 | 0.77889861 |
| NPTX2 | -2.5101878 | 3.48190768 | 70.0576499 | 5.76E-17 | 1.10E-15 | 0.44313203 | 1.45984279 |
| TNIP3 | -2.5093167 | 2.21598795 | 69.6376377 | 7.13E-17 | 1.35E-15 | 1.61460278 | 0.49852366 |
| C15orf48 | -2.4989961 | -1.6917981 | 69.6107449 | 7.22E-17 | 1.37E-15 | 1.36549129 | 0.32679333 |
| HYDIN | -2.4935275 | -0.6558791 | 68.5543185 | 1.23E-16 | 2.29E-15 | 1.13367083 | 0.24045766 |
| LINC01929 | -2.4921978 | -1.6942014 | 68.5395556 | 1.24E-16 | 2.30E-15 | 0.34827441 | 1.35428014 |
| CXCL10 | -2.4865085 | 3.37891086 | 68.4563818 | 1.30E-16 | 2.40E-15 | 0.80648668 | 0.0348435 |
| LINC00842 | -2.4809275 | 5.11261705 | 68.3155853 | 1.39E-16 | 2.56E-15 | 0.8971321 | 0.10028707 |
| GMNC | -2.4790363 | 2.95054474 | 66.4071073 | 3.67E-16 | 6.55E-15 | 1.09489583 | 0.19620668 |
| PLAC9P1 | -2.4770771 | -0.1507337 | 66.3143878 | 3.84E-16 | 6.86E-15 | 1.09480818 | 0.2385864 |
| ZDHHC15 | -2.4723194 | 0.23292728 | 66.2948913 | 3.88E-16 | 6.92E-15 | 1.42398745 | 0.38930864 |
| KCNQ3 | -2.4707216 | -1.3348167 | 65.1893238 | 6.80E-16 | 1.18E-14 | 1.19696545 | 0.20277638 |
| FBXO43 | -2.4700231 | 7.87145566 | 63.9430523 | 1.28E-15 | 2.17E-14 | 0.40653331 | 1.33289841 |
| ZIC4 | -2.4659453 | 1.70650457 | 63.8689287 | 1.33E-15 | 2.24E-14 | 1.40963725 | 0.39683247 |
| GSTT2B | -2.4593656 | -2.5944663 | 62.7209697 | 2.38E-15 | 3.94E-14 | 1.31983979 | 0.37905264 |
| CHL1 | -2.4589827 | -2.2385022 | 62.6672958 | 2.45E-15 | 4.04E-14 | 0.73272738 | 0.01676674 |
| NR6A1 | -2.4567765 | 3.33325011 | 61.1017854 | 5.42E-15 | 8.63E-14 | 0.37534576 | 1.25585829 |
| ZBED6 | -2.4567556 | 5.63918322 | 61.0142842 | 5.67E-15 | 9.01E-14 | 5.02525092 | 2.31418522 |
| LINC00473 | -2.4542224 | 1.72261802 | 60.4867936 | 7.41E-15 | 1.17E-13 | 0.96732584 | 0.08348575 |
| C3orf80 | -2.4487009 | 6.02889701 | 59.6581879 | 1.13E-14 | 1.75E-13 | 1.36345978 | 0.36532048 |
| LINC02015 | -2.4430772 | -1.3527248 | 59.5219193 | 1.21E-14 | 1.87E-13 | 1.12809501 | 0.29778695 |
| RAB39B | -2.4401279 | 3.24103558 | 58.9111899 | 1.65E-14 | 2.54E-13 | 1.63635692 | 0.59442344 |
| SNCA | -2.438149 | 0.81482996 | 58.2597415 | 2.30E-14 | 3.46E-13 | 0.43206866 | 1.38616209 |
| KCNMB1 | -2.4355707 | 7.23208745 | 57.5211383 | 3.34E-14 | 5.00E-13 | 1.29309208 | 0.39002282 |
| PART1 | -2.427983 | 1.87273563 | 57.0196531 | 4.31E-14 | 6.37E-13 | 0.38205324 | 1.3761484 |
| KCNJ6 | -2.4221847 | -0.8249882 | 56.6207102 | 5.29E-14 | 7.76E-13 | 0.1634903 | 0.98984143 |
| LINC01018 | -2.4145013 | -2.2730928 | 56.6151283 | 5.30E-14 | 7.78E-13 | 0.79129314 | 0.07853564 |
| IL1B | -2.4133658 | 3.12511129 | 56.3049615 | 6.21E-14 | 9.05E-13 | 0.74841826 | 0.06605188 |
| ECEL1 | -2.4109937 | 1.82151087 | 56.037552 | 7.11E-14 | 1.03E-12 | 0.24450354 | 1.03002459 |
| PHKG1 | -2.4089857 | -2.2733458 | 55.0356008 | 1.18E-13 | 1.67E-12 | 0.50056718 | 1.57582067 |
| PTTG3P | -2.3993483 | 4.36313405 | 54.8748384 | 1.28E-13 | 1.80E-12 | 0.74708438 | 1.93419283 |
| TFAP2A | -2.3985778 | -2.2746043 | 53.9688059 | 2.04E-13 | 2.80E-12 | 0.95814523 | 0.16004192 |
| NOTUM | -2.3959539 | -0.0729317 | 53.9505234 | 2.06E-13 | 2.83E-12 | 1.14191149 | 0.27199115 |
| EHF | -2.3879157 | 1.52302533 | 53.4482414 | 2.65E-13 | 3.62E-12 | 0.51919107 | 0 |
| SCN1A | -2.3877842 | 1.1936044 | 53.2695442 | 2.91E-13 | 3.93E-12 | 0.71652409 | 0.05051291 |
| MBOAT1 | -2.3853854 | -1.3928261 | 53.1905417 | 3.03E-13 | 4.07E-12 | 0.61700001 | 1.7200824 |
| STRA6 | -2.3834369 | 0.30812018 | 52.8402106 | 3.62E-13 | 4.84E-12 | 1.21099694 | 0.28106916 |
| SNAP25 | -2.3825567 | 9.16786897 | 52.1807455 | 5.06E-13 | 6.63E-12 | 0.98711373 | 0.11567325 |
| DIO2 | -2.3796983 | -1.76762 | 52.0809067 | 5.33E-13 | 6.95E-12 | 1.45306446 | 0.48679315 |
| LPAR3 | -2.3794655 | -2.0112205 | 51.9413267 | 5.72E-13 | 7.43E-12 | 0.04845427 | 0.79055768 |
| SAA1 | -2.3791282 | -1.5765764 | 51.8785232 | 5.90E-13 | 7.64E-12 | 1.08509274 | 0.29692093 |
| ADAP2 | -2.3751645 | -0.1590878 | 51.8292202 | 6.05E-13 | 7.82E-12 | 0.25349919 | 1.02845258 |
| SLC6A15 | -2.3719525 | -2.0123112 | 51.7225389 | 6.39E-13 | 8.22E-12 | 0.31552458 | 1.11121412 |
| ZC3H12D | -2.3670808 | 0.6118082 | 51.6881369 | 6.51E-13 | 8.36E-12 | 0.93668176 | 0.18102774 |
| DUXAP10 | -2.3663929 | 8.39823241 | 50.9240491 | 9.60E-13 | 1.21E-11 | 1.3645051 | 0.45365946 |
| MMP10 | -2.3604539 | 3.28170821 | 50.7695274 | 1.04E-12 | 1.30E-11 | 0.81631111 | 0.08291571 |
| PAX9 | -2.359606 | -1.4128186 | 50.7562725 | 1.05E-12 | 1.31E-11 | 0.97551954 | 0.20949478 |
| FOXD2-AS1 | -2.3575815 | -1.4125469 | 50.4783777 | 1.20E-12 | 1.50E-11 | 0.46647609 | 1.40092449 |
| PCBP3 | -2.3546756 | 0.28302171 | 49.0237278 | 2.53E-12 | 3.04E-11 | 0.13548319 | 0.94864373 |
| RCSD1 | -2.3513058 | -1.2621167 | 48.637893 | 3.08E-12 | 3.66E-11 | 0.69689931 | 0.06781205 |
| FILIP1 | -2.3509705 | 0.97202631 | 47.8472787 | 4.61E-12 | 5.37E-11 | 0.96361141 | 0.17943517 |
| CASC15 | -2.3504686 | -1.7924351 | 47.400449 | 5.79E-12 | 6.64E-11 | 1.31659088 | 0.39604423 |
| PLCH1 | -2.345921 | 1.40612983 | 47.2725564 | 6.18E-12 | 7.06E-11 | 0.78048244 | 0.10149061 |
| ADH1C | -2.3406039 | -2.0409489 | 46.2247546 | 1.05E-11 | 1.18E-10 | 0.1790784 | 0.8659831 |
| RGPD1 | -2.332522 | 1.94853199 | 45.5214524 | 1.51E-11 | 1.65E-10 | 0.47407363 | 1.54393022 |
| CYP1A1 | -2.3199875 | -1.8163652 | 45.2715324 | 1.72E-11 | 1.86E-10 | 0.61027563 | 0.0348435 |
| HOXA-AS3 | -2.316198 | 0.15345526 | 45.0762467 | 1.90E-11 | 2.05E-10 | 0.77072177 | 0.13184047 |
| LINC00922 | -2.3156315 | 1.2433399 | 44.9273155 | 2.04E-11 | 2.20E-10 | 0.35271004 | 1.1506736 |
| NBAT1 | -2.3085623 | 0.47514362 | 44.863685 | 2.11E-11 | 2.27E-10 | 0.4639367 | 0 |
| MIR24-2 | -2.308431 | 1.67273171 | 44.7148103 | 2.28E-11 | 2.44E-10 | 0.12937404 | 0.73784197 |
| ST8SIA6 | -2.3032987 | -1.8410215 | 44.5746615 | 2.45E-11 | 2.61E-10 | 1.23832773 | 0.40313264 |
| BABAM2-AS1 | -2.3028642 | -0.2173217 | 44.432885 | 2.63E-11 | 2.79E-10 | 0.30314432 | 1.13704351 |
| EGR4 | -2.3018131 | -1.4686653 | 44.4292432 | 2.64E-11 | 2.79E-10 | 0.69757979 | 0.0672118 |
| LPL | -2.3004151 | 0.51158318 | 44.0649986 | 3.18E-11 | 3.32E-10 | 0.51635435 | 0.01743386 |
| PRL | -2.2983421 | 3.28255583 | 43.9143781 | 3.43E-11 | 3.57E-10 | 0.86537519 | 0.19414963 |
| KCNA1 | -2.29742 | 2.99296115 | 43.5237127 | 4.19E-11 | 4.32E-10 | 0.28974144 | 1.27605284 |
| TMEM176A | -2.2897985 | 6.59097767 | 42.7275674 | 6.29E-11 | 6.34E-10 | 0 | 0.39251605 |
| NKD1 | -2.2845372 | -2.072688 | 42.6421431 | 6.57E-11 | 6.60E-10 | 0.47748724 | 0 |
| CRYBB2 | -2.2825578 | -1.1786915 | 42.4535007 | 7.24E-11 | 7.23E-10 | 0.2128545 | 1.11050168 |
| SGCG | -2.2822052 | -2.3480334 | 42.3946708 | 7.46E-11 | 7.44E-10 | 0.71888927 | 0.11767135 |
| TRPA1 | -2.2733706 | -0.7273788 | 42.3380939 | 7.68E-11 | 7.63E-10 | 0.94870403 | 0.25347873 |
| DEF6 | -2.264722 | 3.15577102 | 42.2334668 | 8.10E-11 | 8.00E-10 | 0.17992431 | 0.82657141 |
| MYOCD | -2.263666 | 1.3578469 | 42.0092536 | 9.08E-11 | 8.91E-10 | 0.68842083 | 0.10095796 |
| GALNT14 | -2.2628526 | -0.4712397 | 41.7363342 | 1.04E-10 | 1.02E-09 | 0.15647699 | 0.84079825 |
| DCHS2 | -2.2605625 | 6.8446535 | 41.610857 | 1.11E-10 | 1.08E-09 | 0.8056322 | 0.08472387 |
| AATK | -2.2558559 | 4.75346697 | 41.5810314 | 1.13E-10 | 1.09E-09 | 0.83129508 | 0.15832389 |
| MEFV | -2.253126 | -0.3286169 | 41.5072886 | 1.17E-10 | 1.13E-09 | 0.49874609 | 0.01676674 |
| CADM3 | -2.2489903 | 1.11150802 | 40.5317084 | 1.93E-10 | 1.81E-09 | 0.74377683 | 0.13228841 |
| TRHDE | -2.2428161 | 1.20466812 | 40.0092706 | 2.53E-10 | 2.33E-09 | 0.51397018 | 0.01676674 |
| CYP4F35P | -2.2307848 | -2.384802 | 39.9305862 | 2.63E-10 | 2.42E-09 | 0.77052779 | 0.16487589 |
| SLFN12L | -2.2306516 | 1.78234601 | 39.8164035 | 2.79E-10 | 2.56E-09 | 0.41906846 | 0 |
| RIMS4 | -2.228206 | -2.384259 | 39.3370246 | 3.57E-10 | 3.24E-09 | 0.31279343 | 1.34884455 |
| CNTNAP3C | -2.2279788 | -2.105909 | 39.3254259 | 3.59E-10 | 3.26E-09 | 0.22871586 | 1.04327486 |
| STMN2 | -2.2270212 | 8.90440631 | 38.911066 | 4.44E-10 | 3.97E-09 | 1.01679817 | 0.28470914 |
| PGR | -2.2175435 | 6.09326349 | 38.2219895 | 6.31E-10 | 5.52E-09 | 0.70450266 | 0.09915515 |
| ANGPT2 | -2.2155255 | -2.386223 | 38.18998 | 6.42E-10 | 5.61E-09 | 0.97553056 | 0.31070462 |
| SMKR1 | -2.2146264 | -1.5147009 | 38.0443384 | 6.92E-10 | 6.01E-09 | 0.30107903 | 1.09378293 |
| MUC5AC | -2.2143633 | 0.12183153 | 37.9816065 | 7.14E-10 | 6.19E-09 | 0.5550023 | 0.04864225 |
| NPNT | -2.2123604 | 3.0622926 | 37.9215706 | 7.36E-10 | 6.37E-09 | 0.08434058 | 0.6783593 |
| ECSCR | -2.211586 | -1.691712 | 37.7204085 | 8.16E-10 | 7.01E-09 | 0.26063495 | 0.90925254 |
| VCAN-AS1 | -2.2081654 | -1.2127661 | 37.2749317 | 1.03E-09 | 8.71E-09 | 1.16263603 | 0.31214992 |
| SNORD116-21 | -2.2051796 | 0.16180115 | 37.1968535 | 1.07E-09 | 9.04E-09 | 0.66170634 | 0.11717346 |
| LINC00475 | -2.2044442 | -1.8959684 | 36.8271307 | 1.29E-09 | 1.08E-08 | 0.94393557 | 0.28297483 |
| IDO1 | -2.2010953 | 1.94378171 | 34.814975 | 3.63E-09 | 2.86E-08 | 1.06929125 | 0.33956898 |
| CADM2 | -2.1972155 | 7.61134704 | 34.7040655 | 3.84E-09 | 3.01E-08 | 0.48008745 | 0.03417638 |
| WNK4 | -2.1938861 | 1.18795726 | 34.6450907 | 3.96E-09 | 3.10E-08 | 0.8113843 | 0.20708578 |
| DENND2A | -2.1888776 | -2.1365726 | 33.6408772 | 6.63E-09 | 5.01E-08 | 0.2530116 | 0.85746856 |
| MIR4712 | -2.1887481 | -1.3903441 | 33.6323425 | 6.66E-09 | 5.03E-08 | 0.3458312 | 0 |
| RASD2 | -2.183583 | -2.1374829 | 33.4397036 | 7.35E-09 | 5.51E-08 | 0.46242063 | 0.03296855 |
| SPANXA2-OT1 | -2.1822015 | 1.53390715 | 33.2804506 | 7.98E-09 | 5.96E-08 | 0.34401655 | 0 |
| ZG16B | -2.180529 | -1.7157083 | 33.1754422 | 8.42E-09 | 6.27E-08 | 0.0108013 | 0.36656996 |
| CASZ1 | -2.1800557 | 2.07038354 | 32.961285 | 9.40E-09 | 6.95E-08 | 0.19028554 | 0.71652469 |
| GRP | -2.1778347 | -1.3911205 | 32.7885722 | 1.03E-08 | 7.56E-08 | 0.02453484 | 0.41724396 |
| TMEM176B | -2.1743473 | 4.40067553 | 32.6178179 | 1.12E-08 | 8.22E-08 | 0 | 0.29943388 |
| CCL13 | -2.1734686 | 7.66235492 | 32.2096649 | 1.38E-08 | 1.00E-07 | 0.69443074 | 0.14746735 |
| AIF1L | -2.169774 | 6.24587308 | 32.170147 | 1.41E-08 | 1.02E-07 | 0.12626834 | 0.67340171 |
| LGR5 | -2.1679382 | -1.9246139 | 31.7688519 | 1.74E-08 | 1.23E-07 | 0.77785005 | 0.17894236 |
| MUC19 | -2.1631963 | -1.2615235 | 31.6431933 | 1.85E-08 | 1.31E-07 | 0.48726558 | 0.05040241 |
| FRG1JP | -2.1628332 | -2.4238952 | 31.5514501 | 1.94E-08 | 1.37E-07 | 0.91541159 | 0.20866269 |
| BRINP2 | -2.1579685 | 1.76303322 | 30.5565358 | 3.24E-08 | 2.20E-07 | 0.31762696 | 0 |
| KCNMB2 | -2.1527214 | -1.9252965 | 30.4958891 | 3.35E-08 | 2.27E-07 | 0.11730982 | 0.63472081 |
| B3GALT5-AS1 | -2.1480363 | -1.4111566 | 30.4906121 | 3.35E-08 | 2.27E-07 | 0.7964077 | 0.22056302 |
| LINC00514 | -2.1472538 | -1.7399724 | 29.8255116 | 4.73E-08 | 3.14E-07 | 0.03248517 | 0.43209539 |
| VANGL2 | -2.1471033 | -0.9307707 | 29.8016397 | 4.79E-08 | 3.18E-07 | 0.42622895 | 0.03417638 |
| ANO9 | -2.143956 | -1.7402451 | 29.562057 | 5.42E-08 | 3.57E-07 | 0.55852189 | 0.10084747 |
| LINC00852 | -2.1438379 | 3.93660547 | 29.3718843 | 5.97E-08 | 3.91E-07 | 0.17025529 | 0.67192491 |
| PPP2R2B | -2.1378955 | -2.1687417 | 29.2707406 | 6.29E-08 | 4.11E-07 | 0.49153605 | 0.01740964 |
| FGF12 | -2.1360549 | 0.76768579 | 28.9739146 | 7.34E-08 | 4.74E-07 | 0.06828753 | 0.49559719 |
| AMZ1 | -2.1312521 | 1.06700819 | 28.9191402 | 7.55E-08 | 4.86E-07 | 0.75086915 | 0.22334094 |
| PALM2-AKAP2 | -2.1270649 | 2.00240838 | 28.9179793 | 7.55E-08 | 4.87E-07 | 0.59782329 | 0.11666188 |
| MC5R | -2.1250897 | -0.749814 | 28.8288973 | 7.91E-08 | 5.08E-07 | 0.31033443 | 0 |
| RBP1 | -2.124663 | 5.46652686 | 28.7825965 | 8.10E-08 | 5.20E-07 | 0.97704295 | 0.20321315 |
| NOS3 | -2.1225734 | -1.9515902 | 28.7532607 | 8.22E-08 | 5.28E-07 | 0.07740988 | 0.52023584 |
| SIM1 | -2.114856 | 0.40675918 | 28.3281353 | 1.02E-07 | 6.48E-07 | 0.4354073 | 0.03296855 |
| CLCNKA | -2.1137318 | -0.4349761 | 28.2050667 | 1.09E-07 | 6.87E-07 | 0.44103694 | 0.0348435 |
| PTPRD-AS1 | -2.1132191 | 3.97659068 | 27.8093216 | 1.34E-07 | 8.30E-07 | 0.03533614 | 0.39251605 |
| GPR4 | -2.1123765 | 0.58355756 | 27.3496848 | 1.70E-07 | 1.04E-06 | 0.04511723 | 0.41996341 |
| KDR | -2.1093177 | 5.66218877 | 27.1757978 | 1.86E-07 | 1.13E-06 | 0.39101111 | 0.01743386 |
| KIF5A | -2.0996986 | -1.0655313 | 27.161963 | 1.87E-07 | 1.13E-06 | 0.55682815 | 0.10095796 |
| CP | -2.0941232 | 0.08238449 | 26.5853671 | 2.52E-07 | 1.50E-06 | 0.58790221 | 0.13392651 |
| FAM189A2 | -2.0858295 | 0.25548838 | 26.4374103 | 2.72E-07 | 1.61E-06 | 0.1661877 | 0.69416846 |
| ADAM28 | -2.0840048 | -1.9810163 | 26.3095656 | 2.91E-07 | 1.71E-06 | 0.57676819 | 0.0672118 |
| DBET | -2.0808024 | 0.65464383 | 25.9730434 | 3.46E-07 | 2.01E-06 | 0.37821825 | 1.37757111 |
| CARD11 | -2.077896 | 3.66356792 | 25.5521088 | 4.31E-07 | 2.46E-06 | 0.37025723 | 0.01676674 |
| ZNF711 | -2.0737952 | 3.92906718 | 25.269891 | 4.98E-07 | 2.81E-06 | 0.64945009 | 0.18056132 |
| DAPL1 | -2.0721992 | -1.9827498 | 25.0211214 | 5.67E-07 | 3.16E-06 | 0.06710562 | 0.54207414 |
| PTTG2 | -2.0690675 | 5.00987946 | 24.9458834 | 5.90E-07 | 3.28E-06 | 0.16022887 | 0.73227997 |
| FCMR | -2.0672589 | 3.49108412 | 24.8352518 | 6.24E-07 | 3.46E-06 | 0.53449596 | 0.1177247 |
| NRGN | -2.0599725 | 2.38769775 | 24.8248088 | 6.28E-07 | 3.48E-06 | 0.0977642 | 0.55991192 |
| BTLA | -2.0558912 | -0.2261996 | 24.5665722 | 7.18E-07 | 3.94E-06 | 0.45636758 | 0.03425775 |
| PSORS1C2 | -2.0537556 | 1.90294614 | 24.4350587 | 7.69E-07 | 4.20E-06 | 0.11874572 | 0.68653199 |
| ANKRD36BP1 | -2.044507 | 5.6714632 | 24.3725592 | 7.94E-07 | 4.33E-06 | 2.51088708 | 0.94430284 |
| MSH5-SAPCD1 | -2.0430147 | 2.0196489 | 24.0435622 | 9.42E-07 | 5.06E-06 | 0.17123272 | 0.67379627 |
| LINC02587 | -2.0397496 | -2.0102525 | 23.8687413 | 1.03E-06 | 5.52E-06 | 0.0777955 | 0.50604811 |
| SAA2 | -2.038543 | -2.0103658 | 23.8137262 | 1.06E-06 | 5.67E-06 | 0.41964496 | 0.05164482 |
| SNORD116-24 | -2.0331126 | 2.62124783 | 23.4685027 | 1.27E-06 | 6.71E-06 | 0.31510967 | 0.01743386 |
| GYPE | -2.0323709 | 3.83480893 | 23.3151561 | 1.38E-06 | 7.23E-06 | 0.38674782 | 0.01676674 |
| HYDIN2 | -2.0307281 | 4.840868 | 23.3148498 | 1.38E-06 | 7.23E-06 | 0.52433642 | 0.11717346 |
| TREML3P | -2.0187845 | 5.29446374 | 23.1325538 | 1.51E-06 | 7.90E-06 | 0.33459106 | 0.01676674 |
| GYG2P1 | -2.0180787 | -1.1265814 | 23.1322343 | 1.51E-06 | 7.90E-06 | 0.31430443 | 0 |
| LINC01436 | -2.0172643 | 3.56438006 | 23.0144274 | 1.61E-06 | 8.38E-06 | 0 | 0.22334094 |
| LINC01176 | -2.0085887 | -0.8372987 | 22.8764059 | 1.73E-06 | 8.97E-06 | 0.12406778 | 0.56531505 |
| AMTN | -2.0071025 | -0.3828943 | 22.7863967 | 1.81E-06 | 9.36E-06 | 0.26868818 | 0 |
| UNC13A | -2.0056011 | -1.5125124 | 22.7809246 | 1.82E-06 | 9.39E-06 | 0.47788597 | 0.08472387 |
| CSF3 | 2.0005337 | -0.8835349 | 22.7587648 | 1.84E-06 | 9.48E-06 | 0.248481 | 0 |
| LONRF3 | 2.00062274 | 4.59476718 | 22.6696782 | 1.92E-06 | 9.88E-06 | 0.30859552 | 0.01676674 |
| VAT1L | 2.00310363 | 0.85032043 | 22.6456582 | 1.95E-06 | 1.00E-05 | 0.25296189 | 0 |
| LAMB2P1 | 2.00339639 | 2.36399467 | 22.6399723 | 1.95E-06 | 1.00E-05 | 0.13638775 | 0.56318783 |
| CRIP3 | 2.01446743 | 4.05985261 | 22.5880284 | 2.01E-06 | 1.03E-05 | 0.06928798 | 0.4305744 |
| C1QL3 | 2.01600827 | 4.94910557 | 22.3622353 | 2.26E-06 | 1.15E-05 | 0.62507118 | 0.17958527 |
| SSTR2 | 2.01755363 | 1.24648014 | 22.2464327 | 2.40E-06 | 1.21E-05 | 0.05398203 | 0.39557158 |
| IRX6 | 2.02407345 | -2.0277883 | 22.2443785 | 2.40E-06 | 1.21E-05 | 0.11289282 | 0.56836412 |
| TLR2 | 2.0273778 | -2.5840035 | 22.1055879 | 2.58E-06 | 1.29E-05 | 0.48975088 | 0.08146228 |
| PLA2G5 | 2.03012801 | 3.51190042 | 22.030144 | 2.68E-06 | 1.34E-05 | 0.59625178 | 0.1177247 |
| LRFN5 | 2.03145154 | 3.55168183 | 21.7883667 | 3.04E-06 | 1.51E-05 | 0.62040637 | 0.11432479 |
| RPS4Y2 | 2.03383724 | 5.43423914 | 21.7449148 | 3.11E-06 | 1.54E-05 | 0.23498169 | 0 |
| SOWAHD | 2.0416921 | 2.78774874 | 21.7400338 | 3.12E-06 | 1.54E-05 | 0.56840641 | 0.10084747 |
| REM1 | 2.04574482 | 0.55513578 | 21.5550321 | 3.44E-06 | 1.69E-05 | 0.16299078 | 0.65432977 |
| C13orf46 | 2.0535913 | 4.56004955 | 21.3687603 | 3.79E-06 | 1.85E-05 | 0.46918149 | 0.0834136 |
| ELFN2 | 2.05863629 | -2.1215398 | 21.3439143 | 3.84E-06 | 1.87E-05 | 0.24344339 | 0 |
| STAB2 | 2.0588278 | 3.31408511 | 21.1883366 | 4.16E-06 | 2.01E-05 | 0.08015652 | 0.442322 |
| TSPEAR-AS1 | 2.05974047 | -0.351696 | 21.1217761 | 4.31E-06 | 2.08E-05 | 0.26548037 | 0 |
| MALRD1 | 2.0612053 | 3.13315479 | 21.1007057 | 4.36E-06 | 2.10E-05 | 0.71438244 | 0.19395331 |
| FMO3 | 2.06382351 | 3.38885977 | 21.0755696 | 4.42E-06 | 2.12E-05 | 0.42001927 | 0.06717951 |
| TLL1 | 2.0681616 | -2.1217974 | 20.9489436 | 4.72E-06 | 2.26E-05 | 0.53092427 | 0.13174373 |
| ANXA8 | 2.07625691 | 0.06352716 | 20.5605099 | 5.78E-06 | 2.73E-05 | 0.52397967 | 0.11279339 |
| LINC01910 | 2.07701394 | 6.13244857 | 20.4953332 | 5.98E-06 | 2.82E-05 | 0.55536933 | 0.11561399 |
| HOXC13 | 2.07743566 | 0.28550266 | 20.4869588 | 6.00E-06 | 2.83E-05 | 0.46535254 | 0.06717951 |
| KCNS1 | 2.07942691 | 3.26044669 | 20.2276259 | 6.88E-06 | 3.20E-05 | 0.11593141 | 0.47362836 |
| DOC2A | 2.07960532 | 4.00736537 | 20.1635401 | 7.11E-06 | 3.30E-05 | 0.33029081 | 0.03425775 |
| C2CD4B | 2.08492502 | -1.4034322 | 20.0560066 | 7.52E-06 | 3.48E-05 | 0.02453484 | 0.29692093 |
| UBE2QL1 | 2.08936524 | 0.5336791 | 20.0530629 | 7.53E-06 | 3.48E-05 | 0.55948426 | 0.11661472 |
| NR2E3 | 2.09065547 | 4.64992953 | 20.0188289 | 7.67E-06 | 3.54E-05 | 0.03261671 | 0.35193376 |
| GSTT2 | 2.09121572 | 4.31443413 | 19.9840846 | 7.81E-06 | 3.60E-05 | 0.4381571 | 0.08463594 |
| RPRM | 2.09699645 | 1.40741127 | 19.8108023 | 8.55E-06 | 3.91E-05 | 0.0804479 | 0.46098455 |
| HTR1F | 2.10921101 | 1.40932032 | 19.7941361 | 8.62E-06 | 3.94E-05 | 0.01111986 | 0.2525418 |
| PIWIL2 | 2.10990344 | 5.42077407 | 19.7400886 | 8.87E-06 | 4.04E-05 | 0.51908607 | 0.13222508 |
| LSAMP-AS1 | 2.11166637 | 7.02580182 | 19.7110122 | 9.01E-06 | 4.10E-05 | 0.21803711 | 0 |
| CHRM2 | 2.11841633 | -0.79845 | 19.6979589 | 9.07E-06 | 4.13E-05 | 1.08285565 | 0.21756136 |
| PLD5 | 2.11919849 | -2.0888486 | 19.5737064 | 9.68E-06 | 4.39E-05 | 0.42005863 | 0.05044505 |
| FAM151A | 2.12036833 | -2.3739357 | 19.228875 | 1.16E-05 | 5.19E-05 | 0.20976127 | 0 |
| TNFSF14 | 2.12986206 | 4.02330905 | 19.1664938 | 1.20E-05 | 5.35E-05 | 0.26033695 | 0 |
| SPINK1 | 2.13059874 | 4.52496199 | 18.9600879 | 1.33E-05 | 5.92E-05 | 0.2047724 | 0 |
| ZSWIM2 | 2.13247838 | -1.8223876 | 18.7279111 | 1.51E-05 | 6.62E-05 | 0.42856899 | 0.08125505 |
| SEC14L5 | 2.13318217 | 3.97866768 | 18.7124825 | 1.52E-05 | 6.67E-05 | 0.03726718 | 0.42841317 |
| ECRG4 | 2.14841077 | -1.2142962 | 18.6225356 | 1.59E-05 | 6.97E-05 | 0.08813243 | 0.43217635 |
| BPI | 2.16162613 | 0.36713395 | 18.5613628 | 1.65E-05 | 7.18E-05 | 0.47674659 | 0.11652116 |
| MRPL23-AS1 | 2.16217172 | -0.1996558 | 18.4998396 | 1.70E-05 | 7.40E-05 | 0.57549169 | 0.14761125 |
| RNU6-26P | 2.1635005 | 3.81988906 | 18.4953731 | 1.70E-05 | 7.41E-05 | 0.31899574 | 0.03421096 |
| AS3MT | 2.16554567 | -1.8230604 | 18.4187838 | 1.77E-05 | 7.70E-05 | 0.47265196 | 0.09911752 |
| TREH | 2.17031482 | 0.01113957 | 18.1936877 | 2.00E-05 | 8.59E-05 | 0.42189412 | 0.06843413 |
| ERVW-1 | 2.17488392 | 2.63202624 | 17.8905668 | 2.34E-05 | 9.96E-05 | 0.22442215 | 0 |
| SCAT1 | 2.17864067 | -0.0779394 | 17.7608395 | 2.50E-05 | 0.00010599 | 0.21793668 | 0 |
| SLITRK4 | 2.18406316 | -1.34576 | 17.6935207 | 2.60E-05 | 0.00010943 | 0.06981958 | 0.46742063 |
| GTF2IP20 | 2.19039918 | 1.06825041 | 17.6713123 | 2.63E-05 | 0.00011067 | 0.40322608 | 0.06717951 |
| CCL20 | 2.19137262 | -2.1894267 | 17.6418872 | 2.67E-05 | 0.00011215 | 0.43902219 | 0.05037818 |
| PI3 | 2.19367843 | 3.1660068 | 17.6094414 | 2.71E-05 | 0.00011385 | 0.33531207 | 0.05040241 |
| PDZK1IP1 | 2.19473616 | 3.55460167 | 17.4792715 | 2.90E-05 | 0.00012112 | 0.19419599 | 0 |
| NKD2 | 2.20056905 | -0.4516496 | 17.4792715 | 2.90E-05 | 0.00012112 | 0.19419599 | 0 |
| HERC2P10 | 2.20348133 | 0.29920148 | 17.4623908 | 2.93E-05 | 0.00012206 | 0.27240943 | 0.01743386 |
| LINC02076 | 2.20501351 | -2.0584107 | 17.3963669 | 3.03E-05 | 0.00012607 | 0.09027592 | 0.41906653 |
| GAD1 | 2.20932002 | 3.84598433 | 17.2850005 | 3.22E-05 | 0.00013292 | 0.16968337 | 0.62008409 |
| SERPINA6 | 2.20944726 | -1.3245628 | 17.197658 | 3.37E-05 | 0.0001386 | 0.19178445 | 0 |
| PGBD5 | 2.21775054 | 4.10503887 | 17.1167696 | 3.52E-05 | 0.0001442 | 0.35708176 | 0.05040241 |
| TUBBP5 | 2.21942111 | -1.575536 | 17.1128971 | 3.52E-05 | 0.00014442 | 0.07773394 | 0.38204366 |
| LHFPL3-AS2 | 2.22156131 | 0.48557704 | 17.0734118 | 3.60E-05 | 0.00014722 | 0.09298843 | 0.41848713 |
| LINC00640 | 2.22372084 | 5.38854633 | 17.0632095 | 3.62E-05 | 0.00014791 | 0.27601252 | 0.01740964 |
| HOXC13-AS | 2.22892315 | -1.5762165 | 17.049579 | 3.64E-05 | 0.00014881 | 0.19249578 | 0 |
| MIR6835 | 2.22982661 | -1.3055002 | 17.0164876 | 3.71E-05 | 0.00015116 | 0 | 0.16426867 |
| ELF3 | 2.23503624 | 3.21872957 | 16.9103959 | 3.92E-05 | 0.00015893 | 0.26010372 | 0.01676674 |
| PLEKHA6 | 2.23980507 | -0.4731424 | 16.8479468 | 4.05E-05 | 0.00016386 | 0.39231823 | 0.08464566 |
| CD1D | 2.24012999 | 1.88477648 | 16.7346076 | 4.30E-05 | 0.00017321 | 0.18578011 | 0 |
| C14orf39 | 2.24562335 | 0.72890633 | 16.7295828 | 4.31E-05 | 0.00017361 | 0.33239414 | 0.03425775 |
| DGCR5 | 2.2479311 | 0.23762757 | 16.7048964 | 4.37E-05 | 0.00017574 | 0.18692985 | 0 |
| ACTN2 | 2.25453074 | 0.10938583 | 16.6400376 | 4.52E-05 | 0.00018144 | 0.32434474 | 0.05164482 |
| RPTN | 2.26545067 | 4.53880552 | 16.5572848 | 4.72E-05 | 0.0001887 | 0.50993243 | 0.11487878 |
| CD80 | 2.26551274 | -0.411579 | 16.5560574 | 4.72E-05 | 0.00018879 | 0.41929046 | 0.09866468 |
| NMU | 2.26932855 | -2.2979027 | 16.4849776 | 4.90E-05 | 0.00019518 | 0.01111986 | 0.27397735 |
| CFAP221 | 2.27213602 | 3.77614985 | 16.3784849 | 5.19E-05 | 0.0002056 | 0.47569192 | 0.13334505 |
| MIR125B1 | 2.27555556 | 4.46477886 | 16.3485585 | 5.27E-05 | 0.00020852 | 0.30772164 | 0.03421096 |
| LINC01622 | 2.27820408 | -1.9944264 | 16.2847075 | 5.45E-05 | 0.00021495 | 0.61236258 | 0.13326612 |
| CLEC1B | 2.27869869 | 2.07528851 | 16.2247484 | 5.63E-05 | 0.00022139 | 0.01111986 | 0.22229305 |
| SLC8A3 | 2.28084122 | 1.73969129 | 16.1640482 | 5.81E-05 | 0.00022817 | 0.39200227 | 0.0834136 |
| LINC00535 | 2.28165589 | 3.98644465 | 16.0569099 | 6.15E-05 | 0.00024028 | 0.26452102 | 0.01676674 |
| TNFSF18 | 2.2842101 | -1.9949564 | 16.0068506 | 6.31E-05 | 0.00024608 | 0.02421628 | 0.25441478 |
| A1BG-AS1 | 2.29122323 | 1.73376383 | 15.8890307 | 6.72E-05 | 0.00026072 | 0.40924001 | 0.0990873 |
| STK32A | 2.2972251 | 2.94168331 | 15.7868596 | 7.09E-05 | 0.00027419 | 0.29749026 | 0.03425775 |
| PKIB | 2.29800081 | -1.2686899 | 15.6482664 | 7.63E-05 | 0.00029361 | 0.58807336 | 0.1410562 |
| CDHR5 | 2.2999293 | 0.91400198 | 15.6387167 | 7.67E-05 | 0.00029493 | 0.29553167 | 0.01676674 |
| PCDHA4 | 2.30532251 | -2.1214611 | 15.6144424 | 7.77E-05 | 0.00029841 | 0.4255626 | 0.10040672 |
| CXCL11 | 2.30791372 | -1.8506545 | 15.5805031 | 7.91E-05 | 0.00030315 | 0.36816373 | 0.05166739 |
| MS4A4E | 2.32599765 | -2.6295031 | 15.5640085 | 7.98E-05 | 0.00030547 | 0.02453484 | 0.24138079 |
| ARHGAP15 | 2.33547691 | -1.9651419 | 15.519252 | 8.17E-05 | 0.00031256 | 0 | 0.14960021 |
| SLC19A3 | 2.33685375 | 3.46160838 | 15.4390063 | 8.52E-05 | 0.00032494 | 0.37572878 | 0.06846871 |
| MIR4750 | 2.35494773 | -1.5982257 | 15.3930351 | 8.73E-05 | 0.00033221 | 0.04783119 | 0.36025129 |
| LCN1 | 2.35657067 | 2.94613206 | 15.2845054 | 9.25E-05 | 0.00034983 | 0.09315395 | 0.38046693 |
| LINC01592 | 2.35725339 | 2.85945707 | 15.0998676 | 0.00010197 | 0.00038239 | 0.39832917 | 0.08364617 |
| ITK | 2.35920414 | 3.6692752 | 15.0811102 | 0.00010299 | 0.00038579 | 0.39585436 | 0.06727965 |
| DBH | 2.35929415 | 1.82318227 | 15.0686648 | 0.00010367 | 0.00038807 | 0.37025723 | 0.0672118 |
| LIPM | 2.37082296 | 2.79345203 | 14.9880342 | 0.0001082 | 0.00040299 | 0.17040581 | 0 |
| FOXG1 | 2.38053287 | -0.0338271 | 14.9648136 | 0.00010954 | 0.00040748 | 0.23293129 | 0.01676674 |
| ALOX5AP | 2.3853544 | 4.62191265 | 14.8447917 | 0.00011673 | 0.00043218 | 0.22800454 | 0.01676674 |
| SNORD45C | 2.38569022 | 3.25077879 | 14.8274984 | 0.00011781 | 0.00043577 | 0.16383048 | 0 |
| PRRG3 | 2.39132636 | 0.25343136 | 14.8155814 | 0.00011855 | 0.0004383 | 0 | 0.17755961 |
| IRAIN | 2.40945611 | 5.03338666 | 14.6035629 | 0.00013266 | 0.0004856 | 0.40881623 | 0.09915515 |
| MIR27A | 2.41182181 | -0.6415759 | 14.4377159 | 0.00014487 | 0.0005268 | 0.04603169 | 0.28513125 |
| ANKK1 | 2.41902809 | -0.6435904 | 14.385061 | 0.00014898 | 0.00054042 | 0.03533614 | 0.26918419 |
| MIR656 | 2.4223401 | -1.0303791 | 14.3239954 | 0.00015389 | 0.00055603 | 0.1634903 | 0 |
| SMIM32 | 2.42555401 | 2.77855391 | 14.3239954 | 0.00015389 | 0.00055603 | 0.1634903 | 0 |
| CNGA4 | 2.42667146 | 2.27461871 | 14.2870187 | 0.00015694 | 0.00056599 | 0.0347803 | 0.31848043 |
| DSG3 | 2.42698934 | 3.57476321 | 14.2256918 | 0.00016214 | 0.00058363 | 0.04620467 | 0.32288384 |
| GKN1 | 2.42747905 | 5.83038805 | 14.1103928 | 0.00017239 | 0.00061713 | 0.15836689 | 0 |
| PMCH | 2.4313591 | 4.50730614 | 14.0926897 | 0.00017402 | 0.00062233 | 0.15600931 | 0 |
| LINC01483 | 2.45244685 | -0.0993034 | 14.084212 | 0.00017481 | 0.00062472 | 0.24154083 | 0.01676674 |
| LEP | 2.45870172 | 4.95404528 | 14.0458197 | 0.00017841 | 0.00063608 | 0.02192116 | 0.22472054 |
| PSG9 | 2.45920638 | 0.03162283 | 13.9152043 | 0.00019125 | 0.0006777 | 0.36658508 | 0.08291571 |
| PI15 | 2.46756095 | 6.28715139 | 13.8928051 | 0.00019354 | 0.00068513 | 0.03540338 | 0.25522431 |
| CLIP1-AS1 | 2.47388199 | 5.54330895 | 13.8749975 | 0.00019538 | 0.00069119 | 0.15767911 | 0 |
| ERN2 | 2.47403188 | 3.52522541 | 13.8112909 | 0.00020212 | 0.0007125 | 0.15314885 | 0 |
| EREG | 2.48304745 | 5.33995616 | 13.7974647 | 0.00020361 | 0.00071687 | 0.33300605 | 0.06614897 |
| NOVA1 | 2.48804749 | 1.56167077 | 13.7464815 | 0.00020921 | 0.00073455 | 0.33862735 | 0.06623653 |
| PF4V1 | 2.48936682 | 0.34792102 | 13.7452268 | 0.00020935 | 0.00073492 | 0.25941065 | 0.01743386 |
| KRTAP7-1 | 2.49026475 | -2.7774581 | 13.7125032 | 0.00021303 | 0.00074696 | 0.15512932 | 0 |
| LINC00479 | 2.4914486 | -2.7767888 | 13.6412337 | 0.00022127 | 0.00077349 | 0 | 0.1322899 |
| KRT16P3 | 2.49308376 | -1.2115623 | 13.6112485 | 0.00022483 | 0.00078494 | 0.08785335 | 0.47223197 |
| IGSF9B | 2.5026555 | 3.31480065 | 13.5376316 | 0.00023383 | 0.00081269 | 0.25557469 | 0.0348435 |
| C2orf48 | 2.50845243 | 4.4604259 | 13.4230981 | 0.00024855 | 0.00085957 | 0.02421628 | 0.22533092 |
| ADIPOQ | 2.51026831 | -0.9682496 | 13.2867457 | 0.00026729 | 0.0009197 | 0 | 0.1590422 |
| LINC02315 | 2.51549306 | 5.26826251 | 13.1583343 | 0.00028624 | 0.0009801 | 0.1462986 | 0 |
| GGTLC2 | 2.51663745 | 0.37712297 | 13.140392 | 0.000289 | 0.00098792 | 0.27142696 | 0.03417638 |
| NPY4R | 2.51708817 | -1.5983338 | 13.1129595 | 0.00029326 | 0.00100102 | 0.14977655 | 0 |
| MYL4 | 2.52857925 | 3.89752156 | 13.0333452 | 0.00030599 | 0.00103959 | 0.0347803 | 0.24135416 |
| SNORD116-6 | 2.53911327 | 2.2414302 | 12.982258 | 0.00031446 | 0.00106629 | 0.14415461 | 0 |
| MIR3153 | 2.54950729 | 6.83043597 | 12.9390762 | 0.00032179 | 0.00108886 | 0.02136532 | 0.22324489 |
| RDM1 | 2.55954571 | -1.8238989 | 12.8328971 | 0.00034058 | 0.00114633 | 0.09027592 | 0.36668216 |
| GREB1L | 2.56250149 | -1.9640236 | 12.7606032 | 0.000354 | 0.00118787 | 0.38734914 | 0.08123082 |
| CSRP3 | 2.57958195 | -2.4950932 | 12.7423659 | 0.00035747 | 0.00119875 | 0 | 0.14152256 |
| NTN3 | 2.58754676 | -0.0932985 | 12.6800656 | 0.00036958 | 0.00123679 | 0.20416342 | 0.01740964 |
| CYP24A1 | 2.58887132 | -2.7255096 | 12.6769174 | 0.0003702 | 0.00123867 | 0.25111876 | 0.0348435 |
| HAPLN4 | 2.58990919 | 5.32818212 | 12.611203 | 0.00038344 | 0.00127993 | 0.31741569 | 0.06623653 |
| SLC7A11-AS1 | 2.60696513 | 5.17500294 | 12.5934045 | 0.00038711 | 0.00129156 | 0.21274362 | 0.01740964 |
| STAU2-AS1 | 2.61323771 | -0.6167425 | 12.4286854 | 0.00042279 | 0.0013986 | 0 | 0.1323855 |
| FMO2 | 2.6158253 | -3.0008401 | 12.3974621 | 0.00042992 | 0.00142061 | 0.26070419 | 0.03421096 |
| PF4 | 2.63374632 | -1.6457077 | 12.3611521 | 0.00043836 | 0.00144464 | 0.3289333 | 0.0672263 |
| LGALS12 | 2.64918804 | -1.9057267 | 12.3409415 | 0.00044313 | 0.00145853 | 0.0108013 | 0.17963433 |
| MIR6883 | 2.65036136 | -0.219595 | 12.3016009 | 0.00045257 | 0.00148704 | 0.01341498 | 0.32319552 |
| PRRT4 | 2.66653461 | -1.382087 | 12.2928159 | 0.00045471 | 0.00149335 | 0 | 0.12898069 |
| EGFR-AS1 | 2.68186137 | -1.4843553 | 12.2643935 | 0.00046168 | 0.00151438 | 0.14584225 | 0 |
| NKAIN3 | 2.68510916 | 0.91221378 | 12.1974038 | 0.00047856 | 0.00156314 | 0.24034195 | 0.03296855 |
| ENTPD2 | 2.68976074 | -2.6767872 | 12.1704708 | 0.00048552 | 0.0015839 | 0.301871 | 0.01740964 |
| OLAH | 2.70100915 | 3.02187519 | 12.1405146 | 0.00049338 | 0.0016073 | 0.13801922 | 0 |
| LINC00668 | 2.72058834 | -2.024352 | 12.0677698 | 0.00051301 | 0.00166659 | 0.1359001 | 0 |
| SEMA3D | 2.72213444 | 3.56186253 | 12.0574969 | 0.00051585 | 0.0016745 | 0.21959646 | 0.01740964 |
| PDE6A | 2.73688344 | -0.238105 | 12.0073686 | 0.00052991 | 0.00171696 | 0.15126403 | 0 |
| CAMK1G | 2.73785249 | -1.0426438 | 11.994 | 0.00053372 | 0.00172772 | 0.27572748 | 0.05164482 |
| NAT16 | 2.74127844 | 1.6947987 | 11.9509395 | 0.0005462 | 0.00176376 | 0.13139198 | 0 |
| OPN4 | 2.7500256 | -2.9417997 | 11.8658198 | 0.00057174 | 0.00183578 | 0.03758574 | 0.25146879 |
| CPVL | 2.76712213 | -2.6286318 | 11.8631367 | 0.00057256 | 0.00183815 | 0.14066484 | 0 |
| MIR6840 | 2.7729629 | 0.11018584 | 11.7970042 | 0.00059326 | 0.00190025 | 0 | 0.11546601 |
| SNORA1 | 2.77298117 | -1.8216913 | 11.7861324 | 0.00059674 | 0.0019105 | 0.33037565 | 0.08348575 |
| SMIM2-AS1 | 2.77942582 | -2.6288723 | 11.7610328 | 0.00060484 | 0.00193231 | 0.20086043 | 0.01743386 |
| TRPM2 | 2.79069116 | -1.8215796 | 11.6464709 | 0.00064325 | 0.00204228 | 0.27005502 | 0.04864225 |
| MAB21L2 | 2.80729693 | -1.2829677 | 11.5453764 | 0.00067918 | 0.00214631 | 0.03758574 | 0.22612841 |
| ABCB11 | 2.80794417 | -2.3714658 | 11.5326407 | 0.00068385 | 0.00215943 | 0.13920206 | 0 |
| CDH5 | 2.81365192 | -2.1537704 | 11.5197688 | 0.0006886 | 0.00217215 | 0.1004282 | 0.45721272 |
| RBM12B-AS1 | 2.82347337 | -1.7952023 | 11.4942471 | 0.00069812 | 0.0021992 | 0.34859926 | 0.08470281 |
| DDX59-AS1 | 2.83332849 | -1.3603021 | 11.4715201 | 0.00070671 | 0.0022236 | 0.09501526 | 0.38820025 |
| PPP4R1-AS1 | 2.84760981 | 8.20291589 | 11.4425312 | 0.00071782 | 0.00225585 | 0.29816154 | 0.06781205 |
| GRIK1-AS2 | 2.8586643 | -2.5832012 | 11.4300573 | 0.00072266 | 0.00227003 | 0.09315395 | 0.36267972 |
| CLLU1 | 2.86065469 | -2.8840745 | 11.3061981 | 0.00077249 | 0.00241249 | 0.01111986 | 0.17762625 |
| DIO3 | 2.8686141 | -2.5825075 | 11.3004736 | 0.00077487 | 0.00241922 | 0 | 0.11433409 |
| MAGEB17 | 2.88079354 | -0.5227224 | 11.2864063 | 0.00078077 | 0.00243508 | 0 | 0.11487878 |
| ASPA | 2.89539336 | 3.37025023 | 11.24688 | 0.00079757 | 0.00248343 | 0.29276501 | 0.06607611 |
| LRRC10B | 2.8959318 | 6.03445403 | 11.1885651 | 0.00082303 | 0.00255437 | 0 | 0.11563866 |
| MCM8-AS1 | 2.90369554 | -1.9044992 | 11.1653037 | 0.00083341 | 0.0025824 | 0.08028238 | 0.47259417 |
| FMR1-AS1 | 2.94952926 | -1.8764009 | 11.1476206 | 0.0008414 | 0.00260626 | 0.12400879 | 0 |
| SLC14A2 | 2.95550961 | -0.7925713 | 11.071786 | 0.00087651 | 0.00270473 | 0.33896543 | 0.08125505 |
| SNORA31 | 2.97381784 | 3.58838824 | 11.0698314 | 0.00087744 | 0.00270603 | 0.13734007 | 0.53820685 |
| A3GALT2 | 2.98385319 | -2.2578319 | 11.048374 | 0.00088765 | 0.00273311 | 0.12816268 | 0 |
| FAM238C | 2.99787891 | -2.8290972 | 10.9648423 | 0.00092857 | 0.00284908 | 0.0777955 | 0.34840216 |
| SPRY3 | 3.05385379 | 4.94867142 | 10.9452628 | 0.00093843 | 0.00287682 | 0.26275249 | 0.03296855 |
| SMIM22 | 3.06087385 | -3.1939479 | 10.9199341 | 0.00095135 | 0.00291387 | 0.26203383 | 0.05166739 |
| KLB | 3.08656 | -2.7761749 | 10.9006238 | 0.00096132 | 0.0029405 | 0.0347803 | 0.26201057 |
| GZMK | 3.08689692 | -0.8774173 | 10.7860252 | 0.00102269 | 0.00310925 | 0.06739701 | 0.29504598 |
| SMC2-AS1 | 3.08726843 | -2.7768374 | 10.7197072 | 0.00106 | 0.00321338 | 0.29045453 | 0.06605188 |
| C1QTNF4 | 3.08888948 | -2.7769956 | 10.6413837 | 0.00110585 | 0.00334126 | 0.11881002 | 0.4596581 |
| SNORA26 | 3.10210973 | -0.0401701 | 10.6161638 | 0.00112103 | 0.00338227 | 0.13982502 | 0 |
| LINC00271 | 3.1145818 | -2.7763267 | 10.6115756 | 0.00112382 | 0.0033897 | 0.52269057 | 0.12730645 |
| TMEM273 | 3.1441568 | -1.7427107 | 10.5611127 | 0.00115492 | 0.00347501 | 0.24922962 | 0.0348435 |
| ELMOD1 | 3.16979996 | -2.4111192 | 10.527585 | 0.00117606 | 0.00353305 | 0.1178734 | 0 |
| MIR138-1 | 3.18383136 | -2.7249727 | 10.4319543 | 0.00123854 | 0.00370221 | 0.33030412 | 0.08123082 |
| SPINK2 | 3.20871172 | -2.1194429 | 10.4002842 | 0.00125996 | 0.00375822 | 0 | 0.10143726 |
| MIR1281 | 3.21318736 | 1.04618823 | 10.3962268 | 0.00126273 | 0.00376507 | 0.22125277 | 0.03421096 |
| RXFP2 | 3.21598449 | 0.26752229 | 10.297963 | 0.00133177 | 0.00394998 | 0 | 0.10030963 |
| MIR1244-3 | 3.21863229 | -3.1260462 | 10.2876258 | 0.00133925 | 0.00396992 | 0 | 0.10149061 |
| ARHGAP36 | 3.22533583 | 0.54244953 | 10.2850259 | 0.00134114 | 0.0039744 | 0 | 0.10146955 |
| FAM83C | 3.256432 | 3.37073284 | 10.2768383 | 0.00134711 | 0.00398982 | 0.11941599 | 0 |
| C1orf194 | 3.27470647 | -2.6758312 | 10.2340016 | 0.00137875 | 0.00407206 | 0.15307068 | 0 |
| PRC1-AS1 | 3.28870821 | -1.4605728 | 10.2069025 | 0.00139916 | 0.00412595 | 0.21434308 | 0.03296855 |
| ZDHHC8P1 | 3.29633992 | 3.52886522 | 10.1295824 | 0.00145909 | 0.00428641 | 0.22271104 | 0.03425775 |
| CHRM5 | 3.29867022 | -2.3325974 | 10.1272801 | 0.00146091 | 0.00429116 | 0.22958376 | 0.03296855 |
| TAS2R4 | 3.36705928 | -0.9771152 | 10.1235032 | 0.00146391 | 0.00429936 | 0.11388938 | 0 |
| PREX2 | 3.38623135 | -3.0615016 | 10.068836 | 0.00150798 | 0.00441893 | 0.18944376 | 0.01676674 |
| ZYG11A | 3.39011486 | 5.86752697 | 10.0477582 | 0.00152534 | 0.00446541 | 0.06431896 | 0.31724441 |
| LANCL1-AS1 | 3.46452745 | 1.927612 | 10.0449604 | 0.00152765 | 0.00447095 | 0.18720206 | 0.01740964 |
| SLC30A2 | 3.46908581 | -2.2216205 | 10.038456 | 0.00153306 | 0.00448614 | 0 | 0.09866468 |
| RNF144A-AS1 | 3.47018957 | 0.53530243 | 10.0210266 | 0.00154763 | 0.00452186 | 0 | 0.10041291 |
| RIMBP3B | 3.51080086 | -2.9998917 | 10.0210266 | 0.00154763 | 0.00452186 | 0 | 0.10041291 |
| ALDOB | 3.51613694 | -2.9995879 | 9.95575864 | 0.00160347 | 0.00466747 | 0 | 0.0990873 |
| PROZ | 3.5162422 | -2.5370484 | 9.93966008 | 0.00161755 | 0.0047039 | 0.02198839 | 0.20862898 |
| HCAR2 | 3.63215017 | -2.118499 | 9.80622882 | 0.00173922 | 0.00502154 | 0 | 0.11298748 |
| LINC01315 | 3.65757065 | -2.0850433 | 9.79599106 | 0.00174893 | 0.0050468 | 0 | 0.09807893 |
| DUSP26 | 3.67235375 | 0.80773843 | 9.78512261 | 0.0017593 | 0.00507532 | 0.10816269 | 0 |
| THSD7B | 3.74728353 | -0.8024946 | 9.77271599 | 0.00177121 | 0.00510547 | 0.10843024 | 0 |
| IL2RG | 3.80492087 | 0.57303654 | 9.75752242 | 0.0017859 | 0.0051436 | 0 | 0.11353119 |
| MIR4746 | 3.84509324 | -2.8287801 | 9.75397384 | 0.00178936 | 0.00515213 | 0 | 0.10044749 |
| HSPB9 | 3.85049154 | -0.1392131 | 9.71970322 | 0.00182303 | 0.00523974 | 0.01341498 | 0.14700435 |
| CMA1 | 3.85801531 | 3.38395053 | 9.70127814 | 0.0018414 | 0.00529037 | 0.34902674 | 0.08123082 |
| RIMS2 | 3.9463497 | 3.02845379 | 9.62403662 | 0.00192047 | 0.00549874 | 0.18207852 | 0.01740964 |
| BCL2L14 | 4.0329503 | -2.7249253 | 9.57853857 | 0.00196865 | 0.00562518 | 0.04620467 | 0.23960558 |
| CLEC12B | 4.06714486 | -2.1855959 | 9.57242379 | 0.00197522 | 0.00564164 | 0.02453484 | 0.19177053 |
| LINC01936 | 4.11237075 | -3.4997876 | 9.55532898 | 0.0019937 | 0.00568514 | 0.10747491 | 0 |
| TEX14 | 4.11397109 | -3.4997179 | 9.45391412 | 0.00210699 | 0.00597816 | 0.24199456 | 0.05166739 |
| TRBV7-3 | 4.11560308 | -3.4996696 | 9.45201185 | 0.00210918 | 0.00598355 | 0.20416342 | 0.03420061 |
| SLX1B | 4.11669255 | -3.4995999 | 9.36537409 | 0.00221123 | 0.00625111 | 0.03533614 | 0.20960503 |
| BNIPL | 4.11982732 | -3.4994819 | 9.32599566 | 0.00225926 | 0.006374 | 0.08343161 | 0.38383226 |
| LINC01504 | 4.19142883 | -2.6281178 | 9.30601701 | 0.00228403 | 0.0064361 | 0.21825088 | 0.03420061 |
| GPR27 | 4.33549502 | -1.2795688 | 9.24861368 | 0.00235674 | 0.00662411 | 0.24196272 | 0.05040241 |
| LINC01960 | 4.35884027 | -3.4169453 | 9.19337216 | 0.00242893 | 0.0068097 | 0.29505387 | 0.06605188 |
| CNTN2 | 4.36140897 | -3.4168969 | 9.09310338 | 0.00256575 | 0.00715777 | 0.10090424 | 0 |
| SUGT1P4-STRA6LP-CCDC180 | 4.36377211 | -3.4168274 | 9.09310338 | 0.00256575 | 0.00715777 | 0.10090424 | 0 |
| ICOS | 4.36510191 | -3.4167306 | 9.08681679 | 0.00257459 | 0.00717875 | 0.10118333 | 0 |
| CYP3A4 | 4.36550625 | -3.4167094 | 9.07531745 | 0.00259083 | 0.0072202 | 0 | 0.0987901 |
| C1QTNF1-AS1 | 4.36608968 | -3.4166882 | 9.06900179 | 0.00259979 | 0.0072423 | 0.10298197 | 0 |
| SLA | 4.36608968 | -3.4166882 | 9.06714351 | 0.00260243 | 0.0072487 | 0.05925557 | 0.24040597 |
| KRT83 | 4.36662457 | -3.4166399 | 9.0554938 | 0.00261907 | 0.00728826 | 0.10355245 | 0 |
| MYO3B | 4.36740974 | -3.4165915 | 8.99686259 | 0.00270444 | 0.00750295 | 0.32653052 | 0.08468929 |
| LINC02696 | 4.37001883 | -3.4165703 | 8.96601696 | 0.00275048 | 0.0076167 | 0.03261671 | 0.20842719 |
| SNORA11 | 4.37981663 | -3.4164251 | 8.96517952 | 0.00275174 | 0.00761908 | 0.24020736 | 0.04864225 |
| CNTN6 | 4.38278017 | -0.9901794 | 8.9255321 | 0.00281211 | 0.00777088 | 0.09825184 | 0 |
| C11orf42 | 4.48878434 | -2.4103477 | 8.92355655 | 0.00281516 | 0.00777827 | 0.10018677 | 0 |
| FCGR2C | 4.50385335 | 0.5540991 | 8.92201662 | 0.00281753 | 0.0077838 | 0.02136532 | 0.17740756 |
| RNU6ATAC | 4.57175756 | -3.3384942 | 8.85898491 | 0.0029165 | 0.00802553 | 0.19500738 | 0.03417638 |
| ZAR1L | 4.57358231 | -3.3384247 | 8.84708849 | 0.00293557 | 0.00806743 | 0 | 0.13795876 |
| KRT75 | 4.57562401 | -3.3383975 | 8.8167099 | 0.00298484 | 0.00819106 | 0.10697289 | 0 |
| GJB5 | 4.57705689 | -3.3382856 | 8.76738224 | 0.00306665 | 0.00839362 | 0.10202429 | 0 |
| NBPF4 | 4.57888478 | -3.3382373 | 8.73165997 | 0.00312732 | 0.00854963 | 0.10316683 | 0 |
| FAM83B | 4.5971287 | -3.3379259 | 8.73165997 | 0.00312732 | 0.00854963 | 0.10316683 | 0 |
| CCDC144CP | 4.61038402 | -2.3701314 | 8.7202312 | 0.00314698 | 0.00859779 | 0.23082344 | 0.05040241 |
| KLHL33 | 4.75843474 | -3.2639707 | 8.70685113 | 0.00317016 | 0.00865212 | 0.34588339 | 0.08135624 |
| ROPN1 | 4.76138731 | -3.2639495 | 8.67912037 | 0.00321876 | 0.00876539 | 0.10644129 | 0 |
| PNPLA5 | 4.77098254 | -0.6559262 | 8.66226443 | 0.00324867 | 0.00883538 | 0 | 0.08463594 |
| SEMA5A-AS1 | 4.7712345 | -3.2635748 | 8.64925976 | 0.00327194 | 0.00889406 | 0.02192116 | 0.219315 |
| C7orf69 | 4.91254571 | -3.1933704 | 8.62520598 | 0.00331543 | 0.00900229 | 0.26494068 | 0.05037818 |
| NLGN4Y-AS1 | 4.92696176 | -3.192996 | 8.62510224 | 0.00331562 | 0.00900229 | 0.15653357 | 0.01676674 |
| RLN1 | 4.93144968 | -3.1929325 | 8.57264095 | 0.00341252 | 0.00924149 | 0 | 0.08468929 |
| RAD51AP2 | 5.07306704 | -3.1255543 | 8.54022608 | 0.00347383 | 0.00939539 | 0.01111986 | 0.1322899 |
| GGT8P | 5.08344502 | -3.1252435 | 8.52059228 | 0.0035115 | 0.00948874 | 0.20076942 | 0.03296855 |
| NFIA-AS2 | 5.22226935 | -3.0605092 | 8.48429038 | 0.00358226 | 0.00966378 | 0.02192116 | 0.16431549 |
| F13A1 | 5.36727339 | 4.9152034 | 8.4630993 | 0.00362424 | 0.00976322 | 0.01111986 | 0.1590422 |
| HDHD5-AS1 | 5.54516649 | -2.8828873 | 8.44439865 | 0.0036617 | 0.00985148 | 0.23248407 | 0.05051291 |
| SNORD88A | 5.98006226 | -2.6267285 | 8.44055427 | 0.00366944 | 0.0098698 | 0 | 0.0829 |
| SDR9C7 | 6.42437009 | -2.3303695 | 8.43784022 | 0.00367493 | 0.00988327 | 0 | 0.08348146 |
| ALOX5 | 8.34675707 | -0.754886 | 8.42846493 | 0.00369392 | 0.00992419 | 0 | 0.08472387 |

**MSC-ALL3**

|  | **logFC** | **logCPM** | **LR** | **PValue** | **FDR** | **ALL3** | **NBM** |
| --- | --- | --- | --- | --- | --- | --- | --- |
| ADGRG7 | -13.888773 | 5.14197649 | 346.748674 | 2.16E-77 | 2.27E-73 | 6.18703505 | 0 |
| RPS4Y1 | -13.243394 | 7.07103538 | 171.761311 | 3.05E-39 | 2.00E-36 | 7.90332922 | 0.03420061 |
| KDM5D | -12.154081 | 3.41367813 | 143.947418 | 3.65E-33 | 1.47E-30 | 4.3867957 | 0 |
| NLGN4Y | -11.695203 | 2.95854638 | 118.118341 | 1.63E-27 | 3.69E-25 | 3.92348922 | 0 |
| CXCL10 | -11.600476 | 5.43336727 | 255.736405 | 1.46E-57 | 2.78E-54 | 6.4290844 | 0.0348435 |
| BARX1 | -11.069877 | 2.32578051 | 113.577423 | 1.61E-26 | 3.45E-24 | 3.37586812 | 0 |
| CXCL6 | -11.04673 | 5.38491977 | 150.181036 | 1.58E-34 | 7.22E-32 | 6.29381679 | 0.05044505 |
| UTY | -10.390131 | 1.66471349 | 133.689728 | 6.39E-31 | 2.00E-28 | 2.84293455 | 0 |
| TTTY15 | -10.386743 | 1.66489001 | 102.747526 | 3.81E-24 | 6.39E-22 | 2.79026121 | 0 |
| CXCL8 | -10.313907 | 7.84970152 | 248.108469 | 6.71E-56 | 1.17E-52 | 8.79398678 | 0.45664098 |
| CXCL11 | -9.2527166 | 3.59638455 | 136.356844 | 1.67E-31 | 5.38E-29 | 4.5677298 | 0.05166739 |
| TTTY14 | -9.0854166 | 0.36759884 | 63.3309607 | 1.75E-15 | 1.25E-13 | 1.75922402 | 0 |
| PDZK1IP1 | -9.0312394 | 0.30496831 | 54.6671755 | 1.43E-13 | 8.26E-12 | 1.68689487 | 0 |
| CD38 | -8.9801098 | 0.29531521 | 95.9739844 | 1.16E-22 | 1.75E-20 | 1.74370123 | 0 |
| CXCL1 | -8.9096081 | 7.32582691 | 384.589103 | 1.25E-85 | 2.62E-81 | 8.3759057 | 0.74946765 |
| ZFY | -8.9054881 | 1.97987611 | 66.234945 | 4.00E-16 | 3.03E-14 | 2.92843738 | 0.01676674 |
| TXLNGY | -8.8026967 | 1.87336992 | 184.54287 | 4.94E-42 | 3.70E-39 | 3.06910336 | 0.01676674 |
| RSAD2 | -8.6720461 | 7.5011513 | 285.18889 | 5.56E-64 | 1.67E-60 | 8.52389656 | 0.92736046 |
| HOXD11 | -8.5677488 | -0.116961 | 69.3287749 | 8.33E-17 | 6.86E-15 | 1.46206947 | 0 |
| TSPAN8 | -8.4661436 | 1.54204372 | 166.779985 | 3.74E-38 | 2.18E-35 | 2.78266497 | 0.01743386 |
| HLA-DQB1 | -8.408251 | -0.2528067 | 93.0843816 | 5.01E-22 | 6.88E-20 | 1.39545467 | 0 |
| EIF1AY | -8.3953433 | 3.40276141 | 151.097372 | 9.98E-35 | 4.66E-32 | 4.44200036 | 0.08291571 |
| LINC02225 | -8.2684705 | -0.4614131 | 27.2133501 | 1.82E-07 | 4.50E-06 | 1.09841363 | 0 |
| DDX3Y | -7.9259525 | 4.44604168 | 89.5471879 | 2.99E-21 | 3.83E-19 | 5.23910653 | 0.2395204 |
| HLA-DRB1 | -7.7649335 | 4.76197706 | 186.809965 | 1.58E-42 | 1.28E-39 | 5.76523048 | 0.32528087 |
| MIR6891 | -7.6657546 | -0.9533849 | 43.5207496 | 4.19E-11 | 1.78E-09 | 0.95185784 | 0 |
| CPVL | -7.6221479 | -0.9608026 | 67.5446058 | 2.06E-16 | 1.63E-14 | 0.96001774 | 0 |
| ELFN2 | -7.4137158 | -1.1378889 | 66.6850821 | 3.19E-16 | 2.47E-14 | 0.86864425 | 0 |
| USP9Y | -7.3350129 | 2.9868476 | 56.1588224 | 6.68E-14 | 4.06E-12 | 3.71694761 | 0.1322899 |
| OASL | -7.2573999 | 6.10853879 | 236.163224 | 2.70E-53 | 4.36E-50 | 7.10933609 | 0.94755616 |
| CSF3 | -7.2015618 | -1.410769 | 21.6592557 | 3.26E-06 | 6.45E-05 | 0.68716603 | 0 |
| CCL8 | -7.1523549 | 2.6232139 | 227.954116 | 1.67E-51 | 2.06E-48 | 3.76180082 | 0.11652116 |
| CHL1 | -7.1392334 | 0.28065772 | 40.2648333 | 2.22E-10 | 8.64E-09 | 1.59889329 | 0.01676674 |
| DGCR5 | -7.0697059 | -1.433525 | 47.5552683 | 5.35E-12 | 2.57E-10 | 0.71981626 | 0 |
| IDO1 | -7.0178709 | 4.08617643 | 258.217921 | 4.20E-58 | 8.81E-55 | 5.15685671 | 0.33956898 |
| CXCL5 | -7.0165131 | 2.49544731 | 102.765606 | 3.77E-24 | 6.39E-22 | 3.53286069 | 0.11661472 |
| TAC3 | -6.9910477 | 0.861221 | 99.7462033 | 1.73E-23 | 2.78E-21 | 2.19117817 | 0.03425775 |
| PI3 | -6.9819662 | 1.31161105 | 37.0920814 | 1.13E-09 | 3.94E-08 | 2.26167667 | 0.05040241 |
| RPS4Y2 | -6.8602316 | -1.668045 | 23.6395571 | 1.16E-06 | 2.47E-05 | 0.60175793 | 0 |
| IFNB1 | -6.8575018 | -1.5952296 | 44.5997905 | 2.42E-11 | 1.07E-09 | 0.61745956 | 0 |
| MMP10 | -6.7772716 | 1.80671831 | 179.676696 | 5.70E-41 | 3.86E-38 | 3.01336 | 0.08291571 |
| KRTAP7-1 | -6.7534242 | -1.6791006 | 47.8545327 | 4.59E-12 | 2.22E-10 | 0.60124135 | 0 |
| SFRP2 | -6.7121395 | 6.17960021 | 312.382023 | 6.61E-70 | 3.47E-66 | 7.22753349 | 1.2693566 |
| SOST | -6.695066 | -1.6787251 | 24.0458003 | 9.41E-07 | 2.02E-05 | 0.54438573 | 0 |
| IFIT2 | -6.6616459 | 8.43044866 | 215.25386 | 9.81E-49 | 1.03E-45 | 9.39976911 | 3.0134581 |
| KLHDC7B | -6.6120565 | 3.31601703 | 151.251348 | 9.24E-35 | 4.41E-32 | 4.36420389 | 0.26770288 |
| HOXD10 | -6.6087292 | -0.2439988 | 76.221079 | 2.54E-18 | 2.45E-16 | 1.39080727 | 0.01743386 |
| BICDL2 | -6.5486172 | -1.9883655 | 11.5384001 | 0.00068173 | 0.00711237 | 0.44846798 | 0 |
| MX1 | -6.5356234 | 9.18597402 | 322.633639 | 3.87E-72 | 2.71E-68 | 10.2132767 | 3.80574554 |
| CCL5 | -6.5300804 | 5.45409911 | 138.612567 | 5.35E-32 | 1.78E-29 | 6.40243116 | 0.97361115 |
| SLC12A5 | -6.5280596 | -1.8512362 | 35.3966072 | 2.69E-09 | 8.80E-08 | 0.5193318 | 0 |
| HLA-DQA1 | -6.5019884 | -1.8696316 | 41.4705305 | 1.20E-10 | 4.84E-09 | 0.52328971 | 0 |
| ARAP2 | -6.4769261 | -1.8815395 | 23.1640727 | 1.49E-06 | 3.11E-05 | 0.48378281 | 0 |
| TMEM52B | -6.473052 | -1.8752896 | 29.713674 | 5.01E-08 | 1.37E-06 | 0.48663222 | 0 |
| IL36B | -6.4376529 | -1.8554686 | 12.9776007 | 0.00031524 | 0.00367098 | 0.45940849 | 0 |
| CP | -6.4261762 | 2.09835276 | 94.4614674 | 2.50E-22 | 3.59E-20 | 3.16550311 | 0.13392651 |
| IL1RN | -6.4164784 | -0.433805 | 54.0888688 | 1.92E-13 | 1.09E-11 | 1.25517127 | 0.01740964 |
| IL32 | -6.3978474 | 4.03541756 | 73.9907135 | 7.85E-18 | 7.26E-16 | 4.8441757 | 0.48059631 |
| NBAT1 | -6.3866862 | -1.9824149 | 27.3122485 | 1.73E-07 | 4.29E-06 | 0.48499515 | 0 |
| FDCSP | -6.3828136 | -1.9850249 | 26.8630989 | 2.18E-07 | 5.28E-06 | 0.48253148 | 0 |
| ZIC1 | -6.373605 | 1.86783048 | 119.927897 | 6.56E-28 | 1.57E-25 | 3.03319557 | 0.11561399 |
| GDF10 | -6.359124 | -1.9729752 | 38.5076447 | 5.45E-10 | 1.98E-08 | 0.48328356 | 0 |
| SLCO1A2 | -6.3314515 | -1.9997091 | 35.9379298 | 2.04E-09 | 6.85E-08 | 0.45612189 | 0 |
| ACTN2 | -6.2720331 | 0.66473567 | 140.272494 | 2.32E-32 | 8.55E-30 | 2.05422643 | 0.05164482 |
| DCLK3 | -6.255207 | 0.16490841 | 112.872903 | 2.30E-26 | 4.88E-24 | 1.67645314 | 0.03420061 |
| NOD2 | -6.224637 | -0.5646644 | 39.871933 | 2.71E-10 | 1.05E-08 | 1.10969099 | 0.01676674 |
| MT1F | -6.1913102 | 4.44811107 | 50.1393491 | 1.43E-12 | 7.30E-11 | 5.14123551 | 0.66976108 |
| CLEC2L | -6.1716539 | -2.201433 | 15.0845749 | 0.0001028 | 0.00137652 | 0.39773725 | 0 |
| WFDC2 | -6.1513482 | -2.2398136 | 11.9287055 | 0.00055276 | 0.00592298 | 0.37721717 | 0 |
| PRKY | -6.1473591 | 1.82001945 | 144.354512 | 2.97E-33 | 1.22E-30 | 3.01925076 | 0.12918928 |
| BEX1 | -6.1294294 | 1.40354215 | 75.0157799 | 4.67E-18 | 4.44E-16 | 2.58557916 | 0.09915515 |
| OLR1 | -6.1213974 | 2.83894258 | 125.803528 | 3.39E-29 | 8.91E-27 | 3.89958363 | 0.26683758 |
| DAPP1 | -6.1105862 | -2.1517286 | 29.3734115 | 5.97E-08 | 1.60E-06 | 0.4005673 | 0 |
| ANOS1 | -6.0891285 | 5.57149742 | 107.948182 | 2.76E-25 | 5.22E-23 | 6.45922991 | 1.27487596 |
| GBP6 | -6.0433448 | -0.7248675 | 30.7896935 | 2.88E-08 | 8.20E-07 | 0.99522181 | 0.01676674 |
| MT3 | -5.9913386 | -2.3390846 | 12.72903 | 0.00036002 | 0.00412612 | 0.35238099 | 0 |
| MMP3 | -5.9521679 | 5.62432742 | 305.321229 | 2.28E-68 | 9.59E-65 | 6.66598655 | 1.39757679 |
| LGALS9 | -5.9508531 | 5.01721607 | 96.7600109 | 7.83E-23 | 1.20E-20 | 5.9139085 | 1.03895158 |
| CCL13 | -5.9506937 | 1.77979973 | 90.8843567 | 1.52E-21 | 2.04E-19 | 2.92036642 | 0.14746735 |
| PCDH17 | -5.9473542 | -0.8300466 | 57.962035 | 2.67E-14 | 1.73E-12 | 1.00788442 | 0.01743386 |
| HLA-DRB5 | -5.9373971 | 3.2510562 | 149.102365 | 2.72E-34 | 1.22E-31 | 4.29460222 | 0.39438672 |
| BTC | -5.8803203 | 0.67850901 | 37.8725645 | 7.55E-10 | 2.71E-08 | 1.87595997 | 0.06846871 |
| CNTN2 | -5.8577745 | -2.3193701 | 24.2767584 | 8.34E-07 | 1.81E-05 | 0.34040181 | 0 |
| HERC5 | -5.8390059 | 3.93725579 | 228.716175 | 1.14E-51 | 1.49E-48 | 4.99330353 | 0.62770091 |
| C15orf48 | -5.8178295 | 2.83351995 | 167.07461 | 3.22E-38 | 1.93E-35 | 3.92245805 | 0.32679333 |
| GBP5 | -5.8109004 | 2.53576381 | 96.7258608 | 7.96E-23 | 1.21E-20 | 3.55737261 | 0.27039017 |
| GYG2P1 | -5.7586231 | -2.3870425 | 23.1429163 | 1.50E-06 | 3.15E-05 | 0.33843133 | 0 |
| TNF | -5.7439292 | -2.377054 | 16.5858781 | 4.65E-05 | 0.00068316 | 0.31256784 | 0 |
| PCSK1N | -5.736968 | 4.69888734 | 43.9517676 | 3.37E-11 | 1.45E-09 | 5.30354816 | 0.97615219 |
| CD1D | -5.7220679 | -2.4303593 | 18.4584139 | 1.74E-05 | 0.00028686 | 0.3245395 | 0 |
| CMPK2 | -5.7090275 | 5.39496369 | 104.398088 | 1.65E-24 | 2.92E-22 | 6.27317706 | 1.40406551 |
| TBX1 | -5.7077459 | 1.81462779 | 78.7546786 | 7.03E-19 | 7.17E-17 | 2.92629768 | 0.17753707 |
| CCL3 | -5.6806321 | -0.3743111 | 57.1429598 | 4.05E-14 | 2.55E-12 | 1.28452572 | 0.03420061 |
| CCL20 | -5.6654852 | 0.10237494 | 59.8886657 | 1.00E-14 | 6.73E-13 | 1.56302381 | 0.05037818 |
| C5orf46 | -5.6518523 | 0.40296845 | 43.1685899 | 5.02E-11 | 2.11E-09 | 1.75076534 | 0.06717951 |
| IFI27 | -5.6149804 | 6.96510597 | 76.3010675 | 2.44E-18 | 2.37E-16 | 7.72503041 | 2.64135418 |
| ISG15 | -5.5983262 | 9.30316628 | 44.5906866 | 2.43E-11 | 1.07E-09 | 9.80717059 | 4.7802541 |
| MEFV | -5.5976005 | -1.0991957 | 27.5121163 | 1.56E-07 | 3.92E-06 | 0.80417922 | 0.01676674 |
| ZIC2 | -5.5420144 | -2.4871245 | 15.9303694 | 6.57E-05 | 0.00092726 | 0.26773291 | 0 |
| CXCL9 | -5.5020559 | -0.5158434 | 56.6486066 | 5.21E-14 | 3.24E-12 | 1.17605831 | 0.03421096 |
| TMEM233 | -5.4966244 | 1.18648701 | 93.1767851 | 4.78E-22 | 6.61E-20 | 2.43273279 | 0.13392651 |
| HLA-DPB2 | -5.4793785 | -2.532729 | 15.9421255 | 6.53E-05 | 0.00092338 | 0.25987398 | 0 |
| SLC22A3 | -5.466595 | 5.85854039 | 142.872428 | 6.27E-33 | 2.48E-30 | 6.80744401 | 1.87655354 |
| MGAT4C | -5.4588497 | -2.5649848 | 19.2521911 | 1.15E-05 | 0.00019941 | 0.26934428 | 0 |
| SNAP25 | -5.4335415 | 0.97666758 | 43.6011986 | 4.03E-11 | 1.71E-09 | 2.16638604 | 0.11567325 |
| NPY4R | -5.3677173 | -2.6171699 | 18.6768524 | 1.55E-05 | 0.00025886 | 0.25623882 | 0 |
| TDRD1 | -5.3654943 | -2.6496826 | 12.6926421 | 0.0003671 | 0.00418606 | 0.25487591 | 0 |
| TF | -5.3288297 | 3.89887256 | 189.613748 | 3.86E-43 | 3.38E-40 | 4.95408235 | 0.79995858 |
| MX2 | -5.3105877 | 7.01869761 | 289.783655 | 5.54E-65 | 1.94E-61 | 8.04007818 | 2.93936724 |
| SPINK1 | -5.2772624 | -2.6652508 | 18.1039015 | 2.09E-05 | 0.00033545 | 0.24770162 | 0 |
| ICOSLG | -5.2567863 | -0.7642827 | 37.7150077 | 8.19E-10 | 2.90E-08 | 1.04380601 | 0.03421096 |
| EHF | -5.2503199 | -2.6838884 | 17.8832066 | 2.35E-05 | 0.00036914 | 0.22906396 | 0 |
| BST2 | -5.2490666 | 7.22721177 | 54.5943256 | 1.48E-13 | 8.55E-12 | 7.90926538 | 3.15450007 |
| C1QTNF1-AS1 | -5.235721 | -2.7090132 | 14.4899648 | 0.00014091 | 0.0018117 | 0.2425465 | 0 |
| APOBEC3B | -5.2187034 | -0.3173698 | 74.8315667 | 5.13E-18 | 4.83E-16 | 1.33302997 | 0.05051291 |
| AMTN | -5.1871997 | -2.7216378 | 15.1615428 | 9.87E-05 | 0.00132972 | 0.23326039 | 0 |
| CYP26C1 | -5.1868856 | -2.7184964 | 15.9537772 | 6.49E-05 | 0.00091958 | 0.23641618 | 0 |
| CX3CL1 | -5.1534566 | 2.11120619 | 93.3074215 | 4.48E-22 | 6.23E-20 | 3.25069404 | 0.29801337 |
| C3 | -5.1466323 | 6.79173104 | 273.713023 | 1.76E-61 | 4.11E-58 | 7.8102216 | 2.87939506 |
| LIPM | -5.1426758 | -2.7405288 | 16.67311 | 4.44E-05 | 0.00065705 | 0.21520296 | 0 |
| LIPG | -5.1188084 | 3.8661814 | 108.611927 | 1.97E-25 | 3.87E-23 | 4.82022479 | 0.88658474 |
| GBP1P1 | -5.1008805 | 1.84603682 | 144.984889 | 2.16E-33 | 9.09E-31 | 3.01341333 | 0.27043158 |
| ADGRF4 | -5.0549575 | -2.78373 | 15.1169125 | 0.00010105 | 0.00135574 | 0.21674241 | 0 |
| PDE6A | -5.045253 | -2.7900903 | 14.6427487 | 0.00012993 | 0.00168504 | 0.21147698 | 0 |
| COL22A1 | -5.0439136 | 0.14858412 | 85.7045926 | 2.09E-20 | 2.45E-18 | 1.64467711 | 0.08523546 |
| SLFN12L | -5.033619 | -2.8323914 | 11.2764898 | 0.00078495 | 0.00798101 | 0.2099452 | 0 |
| LGALS9C | -4.9989096 | -1.699568 | 13.7397551 | 0.00020996 | 0.00256004 | 0.56148681 | 0.01743386 |
| KLK10 | -4.9923941 | -1.7012158 | 16.6119684 | 4.59E-05 | 0.00067571 | 0.5757695 | 0.01676674 |
| IGF1 | -4.9906982 | 0.71119394 | 105.569408 | 9.16E-25 | 1.66E-22 | 2.05640573 | 0.13334505 |
| LBP | -4.9899386 | 5.32162535 | 219.74965 | 1.03E-49 | 1.13E-46 | 6.34252561 | 1.82122587 |
| PLA2G4E-AS1 | -4.9786879 | -2.8388588 | 12.4879974 | 0.00040958 | 0.00460109 | 0.2065341 | 0 |
| CLDN1 | -4.9563946 | 4.85785029 | 148.649389 | 3.42E-34 | 1.50E-31 | 5.83190223 | 1.53180731 |
| MAOB | -4.9462388 | 1.09647815 | 128.177432 | 1.03E-29 | 2.91E-27 | 2.37150448 | 0.17894236 |
| IFIT1 | -4.9289164 | 8.54806045 | 229.751411 | 6.75E-52 | 9.45E-49 | 9.54551926 | 4.69778708 |
| IGFBP1 | -4.9244649 | 1.30540861 | 139.464551 | 3.49E-32 | 1.22E-29 | 2.55271821 | 0.21092114 |
| DSC2 | -4.9117111 | 1.39676244 | 107.950398 | 2.76E-25 | 5.22E-23 | 2.61760677 | 0.22247677 |
| P2RX1 | -4.9083433 | -2.8549657 | 14.0669994 | 0.00017641 | 0.00220211 | 0.19392527 | 0 |
| MGAM | -4.8950176 | 2.54783184 | 67.513166 | 2.09E-16 | 1.65E-14 | 3.55472462 | 0.46661208 |
| CES1P1 | -4.8897852 | -2.8646162 | 11.9856442 | 0.00053612 | 0.00577546 | 0.18443719 | 0 |
| MCF2L | -4.8826004 | -1.7162055 | 30.5006954 | 3.34E-08 | 9.37E-07 | 0.58125546 | 0.01676674 |
| KRTAP4-12 | -4.8708792 | -2.8741789 | 13.3803449 | 0.00025428 | 0.00303684 | 0.17374282 | 0 |
| GYPE | -4.8680737 | -1.7396973 | 26.2090897 | 3.06E-07 | 7.18E-06 | 0.5707134 | 0.01676674 |
| PIK3AP1 | -4.8546962 | -0.2756511 | 39.1568167 | 3.91E-10 | 1.48E-08 | 1.28586853 | 0.06787892 |
| NKD1 | -4.8503084 | -2.8742105 | 11.0982223 | 0.00086411 | 0.00863657 | 0.17395593 | 0 |
| CSF2 | -4.8451348 | -0.3063655 | 73.4661689 | 1.02E-17 | 9.19E-16 | 1.31743768 | 0.06792254 |
| LINC01882 | -4.8267947 | -1.8543894 | 11.2827235 | 0.00078232 | 0.00795811 | 0.49974809 | 0.01740964 |
| KCNMB1 | -4.8023634 | 2.15449918 | 138.657689 | 5.23E-32 | 1.77E-29 | 3.29222255 | 0.39002282 |
| WFDC1 | -4.7884653 | 2.5040636 | 68.6380402 | 1.18E-16 | 9.66E-15 | 3.55644213 | 0.47720346 |
| SLC7A2 | -4.764637 | 1.52261146 | 56.211663 | 6.51E-14 | 3.97E-12 | 2.63403059 | 0.26268989 |
| ART4 | -4.7614761 | -2.9260549 | 11.8810141 | 0.00056709 | 0.00605319 | 0.17104848 | 0 |
| CALCB | -4.7540534 | -2.9293735 | 11.4429956 | 0.00071764 | 0.00743343 | 0.1705762 | 0 |
| DUSP26 | -4.7540534 | -2.9293735 | 11.4429956 | 0.00071764 | 0.00743343 | 0.1705762 | 0 |
| CHRNA1 | -4.750867 | -1.1765639 | 39.0806134 | 4.07E-10 | 1.52E-08 | 0.82341659 | 0.03421096 |
| LUZP2 | -4.7462519 | -2.9325739 | 12.7920471 | 0.0003481 | 0.00400692 | 0.16786172 | 0 |
| GCH1 | -4.7349538 | 3.32697544 | 83.6677076 | 5.85E-20 | 6.43E-18 | 4.31282788 | 0.79397221 |
| OLAH | -4.7189852 | -2.939081 | 10.7940929 | 0.00101825 | 0.00992068 | 0.16048328 | 0 |
| RFX4 | -4.7189852 | -2.939081 | 10.7940929 | 0.00101825 | 0.00992068 | 0.16048328 | 0 |
| CD80 | -4.7160296 | 0.07874398 | 81.5297489 | 1.73E-19 | 1.87E-17 | 1.57634741 | 0.09866468 |
| CABP1 | -4.709886 | -1.2973352 | 12.4546691 | 0.00041695 | 0.00467392 | 0.70539175 | 0.03421096 |
| RASD2 | -4.7098242 | -1.2417411 | 27.0134808 | 2.02E-07 | 4.93E-06 | 0.78573093 | 0.03296855 |
| NLRP2 | -4.6744351 | 1.65419748 | 79.1177183 | 5.85E-19 | 5.99E-17 | 2.82173618 | 0.30321064 |
| CST2 | -4.6719369 | 0.5512114 | 32.7993548 | 1.02E-08 | 3.10E-07 | 1.83129313 | 0.14712182 |
| CXCL3 | -4.668716 | 3.36647053 | 128.147438 | 1.04E-29 | 2.92E-27 | 4.38341922 | 0.85296015 |
| DCDC2 | -4.6614233 | -1.8614742 | 25.8375277 | 3.71E-07 | 8.59E-06 | 0.48949272 | 0.01743386 |
| TNFRSF18 | -4.6458616 | -1.3174049 | 17.8181167 | 2.43E-05 | 0.00038056 | 0.72291945 | 0.03417638 |
| IFIT1B | -4.6446201 | -1.2586486 | 46.730873 | 8.14E-12 | 3.83E-10 | 0.76954991 | 0.03421096 |
| UBE2QL1 | -4.6219387 | 0.19443621 | 73.6970115 | 9.11E-18 | 8.21E-16 | 1.65714062 | 0.11661472 |
| DSC3 | -4.616585 | -1.2756041 | 39.5395935 | 3.21E-10 | 1.23E-08 | 0.74888763 | 0.03421096 |
| BRINP2 | -4.6022928 | -2.9940306 | 11.2730646 | 0.0007864 | 0.00799187 | 0.15967846 | 0 |
| NRTN | -4.5972137 | -2.9972535 | 11.0652989 | 0.00087958 | 0.00878459 | 0.15479825 | 0 |
| KRT75 | -4.5885463 | -3.0005612 | 11.4356015 | 0.0007205 | 0.00745204 | 0.15315275 | 0 |
| SERPINA6 | -4.5885463 | -3.0005612 | 11.4356015 | 0.0007205 | 0.00745204 | 0.15315275 | 0 |
| STMN2 | -4.5783359 | 1.42747218 | 139.570968 | 3.30E-32 | 1.18E-29 | 2.64516787 | 0.28470914 |
| CDH8 | -4.5746692 | -3.0070735 | 10.9773638 | 0.00092232 | 0.00911291 | 0.14476145 | 0 |
| OPRM1 | -4.5746692 | -3.0070735 | 10.9773638 | 0.00092232 | 0.00911291 | 0.14476145 | 0 |
| ZDHHC8P1 | -4.5685666 | -1.3195638 | 46.8377651 | 7.71E-12 | 3.64E-10 | 0.74564151 | 0.03425775 |
| LONRF3 | -4.5662252 | -1.9351357 | 26.3327296 | 2.87E-07 | 6.77E-06 | 0.46357216 | 0.01676674 |
| GRIK4 | -4.552475 | 0.84503969 | 119.278766 | 9.10E-28 | 2.12E-25 | 2.16439594 | 0.19377116 |
| NRIR | -4.536485 | -1.9629878 | 30.1345617 | 4.03E-08 | 1.12E-06 | 0.45875958 | 0.01676674 |
| RTP4 | -4.534569 | 3.62407252 | 99.3085534 | 2.16E-23 | 3.44E-21 | 4.60270685 | 1.03697113 |
| HES4 | -4.5280404 | 4.1194218 | 46.9305112 | 7.35E-12 | 3.49E-10 | 4.92644405 | 1.29733385 |
| TREM1 | -4.4786672 | 0.0443199 | 41.4666867 | 1.20E-10 | 4.84E-09 | 1.52047271 | 0.11661472 |
| IFIT3 | -4.4675204 | 8.39768172 | 154.49823 | 1.80E-35 | 9.23E-33 | 9.35137556 | 4.98168755 |
| SPATA22 | -4.4609313 | -0.9799062 | 54.0045281 | 2.00E-13 | 1.13E-11 | 0.92207147 | 0.04864225 |
| SLC19A3 | -4.4523004 | -0.6521384 | 64.5022803 | 9.64E-16 | 7.05E-14 | 1.10403275 | 0.06846871 |
| TRPA1 | -4.4461913 | 1.15066172 | 74.7657829 | 5.30E-18 | 4.97E-16 | 2.37191394 | 0.25347873 |
| PKIB | -4.4132035 | 0.35890785 | 25.9882002 | 3.44E-07 | 8.00E-06 | 1.72043807 | 0.1410562 |
| TNFSF13B | -4.4039473 | 3.55752123 | 147.059352 | 7.62E-34 | 3.26E-31 | 4.57955394 | 1.07116354 |
| CEACAM1 | -4.3882551 | -2.0599866 | 25.2524023 | 5.03E-07 | 1.14E-05 | 0.42984642 | 0.01743386 |
| C2orf72 | -4.3786474 | -0.7196102 | 48.0008872 | 4.26E-12 | 2.07E-10 | 1.0599182 | 0.06841157 |
| SLPI | -4.3657737 | 3.79011741 | 37.5307413 | 9.00E-10 | 3.17E-08 | 4.49562102 | 1.2115611 |
| IFI6 | -4.3654805 | 10.467789 | 57.9698854 | 2.66E-14 | 1.72E-12 | 11.2270364 | 7.10864286 |
| GRIP2 | -4.3635707 | 0.9881521 | 60.6480201 | 6.82E-15 | 4.62E-13 | 2.21443655 | 0.23958923 |
| TEKT4P2 | -4.3367172 | 2.96455184 | 95.5668096 | 1.43E-22 | 2.10E-20 | 3.97263144 | 0.81703692 |
| HOXD13 | -4.3305347 | -2.1024499 | 19.2231658 | 1.16E-05 | 0.00020163 | 0.40941096 | 0.01676674 |
| TSPAN2 | -4.3127412 | 4.62054284 | 136.552486 | 1.51E-31 | 4.96E-29 | 5.59970443 | 1.78531133 |
| LINC00842 | -4.3071249 | -0.3109558 | 35.6542069 | 2.36E-09 | 7.84E-08 | 1.28156374 | 0.10028707 |
| USP30-AS1 | -4.3007439 | -0.5385991 | 37.6848162 | 8.31E-10 | 2.94E-08 | 1.15609814 | 0.08463594 |
| IL4I1 | -4.2936412 | 2.41285162 | 61.5736451 | 4.26E-15 | 2.96E-13 | 3.40383317 | 0.61517672 |
| DIO2 | -4.278908 | 2.032604 | 109.513934 | 1.25E-25 | 2.50E-23 | 3.16065746 | 0.48679315 |
| TREML3P | -4.2758297 | -2.1398938 | 19.1948291 | 1.18E-05 | 0.00020397 | 0.39787718 | 0.01676674 |
| CES1 | -4.2748678 | 4.16966956 | 60.0928584 | 9.05E-15 | 6.11E-13 | 5.04095629 | 1.4885862 |
| ALOX5AP | -4.2722675 | -2.18654 | 13.2519747 | 0.00027229 | 0.00323546 | 0.39134176 | 0.01676674 |
| TMOD1 | -4.2668084 | -1.5591162 | 36.8850432 | 1.25E-09 | 4.35E-08 | 0.61902248 | 0.03417638 |
| IFIH1 | -4.2615305 | 5.95809615 | 117.044333 | 2.81E-27 | 6.27E-25 | 6.88202629 | 2.8979975 |
| ETV7 | -4.2372077 | 3.06001289 | 68.4425071 | 1.31E-16 | 1.05E-14 | 4.00077845 | 0.90442187 |
| TPD52 | -4.2349657 | 3.10074894 | 63.3439998 | 1.74E-15 | 1.25E-13 | 4.0059924 | 0.92423979 |
| MMP1 | -4.2324929 | 6.64119777 | 197.366816 | 7.84E-45 | 7.54E-42 | 7.62467019 | 3.52700266 |
| RARRES2 | -4.2316402 | 6.08446997 | 34.7936261 | 3.67E-09 | 1.17E-07 | 6.65878647 | 3.02668524 |
| STC1 | -4.222367 | 4.73782703 | 101.247917 | 8.12E-24 | 1.32E-21 | 5.67650314 | 1.92955246 |
| GPRC5C | -4.20973 | 1.83483331 | 89.4098368 | 3.21E-21 | 4.06E-19 | 2.95740292 | 0.45617019 |
| CHRM5 | -4.2034418 | -1.5993555 | 27.4971928 | 1.57E-07 | 3.95E-06 | 0.58850829 | 0.03296855 |
| FGF10 | -4.184776 | -0.5598586 | 11.5575173 | 0.00067476 | 0.0070554 | 1.03992886 | 0.08135624 |
| GREM1 | -4.1843541 | 8.96460167 | 65.9741314 | 4.57E-16 | 3.41E-14 | 9.77063399 | 5.79532935 |
| EREG | -4.176893 | -0.8479844 | 13.8254242 | 0.0002006 | 0.00246596 | 0.89592587 | 0.06614897 |
| TLR2 | -4.1687589 | -0.6115789 | 29.2492663 | 6.36E-08 | 1.70E-06 | 1.09494923 | 0.08146228 |
| SLC24A3 | -4.1603257 | 2.31972342 | 152.305778 | 5.43E-35 | 2.72E-32 | 3.42125462 | 0.62661484 |
| SLC22A2 | -4.1486732 | -0.6549136 | 44.2419998 | 2.90E-11 | 1.27E-09 | 1.09352594 | 0.08285321 |
| ADAP1 | -4.1441109 | 2.13179568 | 77.7249995 | 1.18E-18 | 1.17E-16 | 3.19450974 | 0.5670261 |
| DDX58 | -4.134922 | 7.03894125 | 170.258084 | 6.50E-39 | 4.13E-36 | 7.99925141 | 3.9875168 |
| GPR37L1 | -4.1275959 | -1.6965923 | 13.351745 | 0.00025818 | 0.00307476 | 0.55665366 | 0.03296855 |
| MMP12 | -4.1240687 | -1.6337524 | 18.111248 | 2.08E-05 | 0.00033473 | 0.55646094 | 0.03421096 |
| PAX9 | -4.1135111 | 0.56143372 | 35.483316 | 2.57E-09 | 8.45E-08 | 1.80017575 | 0.20949478 |
| PLEKHS1 | -4.1022279 | 0.31815239 | 48.6646385 | 3.04E-12 | 1.49E-10 | 1.69699118 | 0.17639603 |
| RBP1 | -4.0745743 | 0.47827613 | 19.6439917 | 9.33E-06 | 0.00016671 | 1.73782089 | 0.20321315 |
| PF4 | -4.0674501 | -0.9979545 | 30.3842411 | 3.54E-08 | 9.89E-07 | 0.89725104 | 0.0672263 |
| PTGER3 | -4.064355 | -1.6972791 | 20.5574707 | 5.79E-06 | 0.00010858 | 0.54518858 | 0.03421096 |
| SYNPO2 | -4.0557015 | 6.19901748 | 116.297416 | 4.09E-27 | 8.86E-25 | 7.13372982 | 3.27054672 |
| CLDN14 | -4.0497062 | 1.21318762 | 98.8047454 | 2.79E-23 | 4.37E-21 | 2.43450862 | 0.34152364 |
| SLC15A3 | -4.043529 | 5.92708831 | 73.7639686 | 8.80E-18 | 7.97E-16 | 6.79260572 | 3.03561951 |
| IL34 | -4.0285281 | 1.61261175 | 50.3962413 | 1.26E-12 | 6.43E-11 | 2.68505531 | 0.44463304 |
| NPPB | -4.0220107 | -1.3305877 | 38.4528035 | 5.61E-10 | 2.03E-08 | 0.73333516 | 0.05166739 |
| RBM11 | -3.9983148 | -1.7598542 | 27.0267552 | 2.01E-07 | 4.90E-06 | 0.54872812 | 0.03296855 |
| HSD11B1 | -3.9746369 | 4.11690603 | 127.746417 | 1.28E-29 | 3.45E-27 | 5.09249124 | 1.66032861 |
| SLC8A2 | -3.9690284 | -0.2159372 | 72.5884589 | 1.60E-17 | 1.40E-15 | 1.36636993 | 0.13334505 |
| LRP2 | -3.9564378 | -1.7922486 | 25.1194718 | 5.39E-07 | 1.22E-05 | 0.53370963 | 0.03296855 |
| BATF2 | -3.9497856 | 1.88334106 | 86.3260631 | 1.53E-20 | 1.80E-18 | 2.99778441 | 0.54093854 |
| TYMP | -3.9384646 | 6.83305959 | 38.7055873 | 4.93E-10 | 1.82E-08 | 7.50108925 | 3.94816128 |
| HLA-DRB6 | -3.9257355 | -0.6502713 | 26.9023483 | 2.14E-07 | 5.19E-06 | 1.06602979 | 0.10028707 |
| ICAM1 | -3.9053591 | 6.44132008 | 180.862166 | 3.14E-41 | 2.27E-38 | 7.40367355 | 3.63294795 |
| TNFAIP6 | -3.8891533 | 5.52760062 | 72.4202681 | 1.74E-17 | 1.52E-15 | 6.3808636 | 2.82741823 |
| KMO | -3.8855289 | -0.4594657 | 59.049787 | 1.54E-14 | 1.02E-12 | 1.20024208 | 0.11652116 |
| WIPF3 | -3.8812342 | -1.8377379 | 24.4705202 | 7.55E-07 | 1.66E-05 | 0.48589787 | 0.03420061 |
| AGT | -3.8555877 | 3.18547843 | 116.933583 | 2.97E-27 | 6.56E-25 | 4.20574216 | 1.14187657 |
| PLG | -3.8498644 | -0.6367941 | 17.0291312 | 3.68E-05 | 0.00055245 | 0.99295384 | 0.10034957 |
| PPP1R14A | -3.849244 | 2.85518729 | 28.5180453 | 9.28E-08 | 2.43E-06 | 3.6350199 | 0.95920321 |
| GBP4 | -3.8453116 | 3.66924068 | 81.3607531 | 1.88E-19 | 2.02E-17 | 4.62608279 | 1.42872438 |
| TMEM132E | -3.8450556 | -2.4071721 | 16.7031405 | 4.37E-05 | 0.00064718 | 0.3079463 | 0.01676674 |
| LINC01592 | -3.8447378 | -0.9247204 | 20.1540222 | 7.14E-06 | 0.00013206 | 0.92147321 | 0.08364617 |
| HERC6 | -3.835339 | 6.32286949 | 185.935635 | 2.45E-42 | 1.91E-39 | 7.28564032 | 3.58448071 |
| JPH3 | -3.8233268 | 0.38871811 | 48.248981 | 3.75E-12 | 1.82E-10 | 1.75632955 | 0.22064701 |
| C19orf81 | -3.8217551 | -1.0109102 | 12.3795573 | 0.00043406 | 0.00483733 | 0.80106647 | 0.08461337 |
| PLA1A | -3.8201878 | 1.18014816 | 94.1223347 | 2.97E-22 | 4.21E-20 | 2.39449141 | 0.38015208 |
| ENTPD2 | -3.8176249 | -2.4180322 | 13.9260808 | 0.00019014 | 0.00235113 | 0.2945301 | 0.01740964 |
| RNF175 | -3.8046109 | -2.4270553 | 15.1978778 | 9.68E-05 | 0.00130718 | 0.28411885 | 0.01743386 |
| ELOVL7 | -3.797461 | -1.1793466 | 16.6589508 | 4.47E-05 | 0.00066104 | 0.74144633 | 0.0672118 |
| CD7 | -3.7952696 | -0.994963 | 17.4856748 | 2.89E-05 | 0.00044527 | 0.86909926 | 0.08348146 |
| ACTG2 | -3.7900294 | 2.80548135 | 55.2552474 | 1.06E-13 | 6.26E-12 | 3.73193908 | 0.98420425 |
| TINAGL1 | -3.7876805 | 2.3285445 | 104.81798 | 1.34E-24 | 2.40E-22 | 3.40017974 | 0.75644497 |
| TGM2 | -3.7725863 | 7.45870472 | 123.147251 | 1.29E-28 | 3.28E-26 | 8.3790618 | 4.70921371 |
| A1BG | -3.7694066 | 1.69973575 | 20.3642644 | 6.40E-06 | 0.00011926 | 2.4792478 | 0.54355071 |
| C1QTNF1 | -3.7681372 | 5.6391994 | 104.221899 | 1.81E-24 | 3.14E-22 | 6.55175488 | 3.02375167 |
| NLGN4Y-AS1 | -3.7653777 | -2.450901 | 16.319869 | 5.35E-05 | 0.00077739 | 0.29761842 | 0.01676674 |
| SOD2 | -3.7622493 | 8.20203546 | 154.827574 | 1.53E-35 | 8.02E-33 | 9.14625225 | 5.43943093 |
| DCHS2 | -3.7538886 | -0.9455092 | 22.4087852 | 2.20E-06 | 4.49E-05 | 0.86526613 | 0.08472387 |
| OAS1 | -3.7439598 | 6.05112829 | 165.198746 | 8.28E-38 | 4.57E-35 | 7.00391246 | 3.41372345 |
| TGFB2 | -3.7439374 | 5.25102687 | 41.9661483 | 9.29E-11 | 3.80E-09 | 5.98523703 | 2.69417366 |
| MIP | -3.7393787 | -2.483113 | 12.2807543 | 0.00045765 | 0.00505997 | 0.2940477 | 0.01743386 |
| MT1G | -3.7376937 | 3.1586499 | 29.4812345 | 5.65E-08 | 1.52E-06 | 3.89570089 | 1.18315553 |
| AMZ1 | -3.737672 | 0.31057888 | 73.8214842 | 8.55E-18 | 7.77E-16 | 1.71667186 | 0.22334094 |
| PF4V1 | -3.7329187 | -2.4709939 | 14.8443695 | 0.00011676 | 0.00154177 | 0.27347705 | 0.01743386 |
| SMCO3 | -3.7320128 | -0.5898527 | 49.8364344 | 1.67E-12 | 8.41E-11 | 1.10682851 | 0.11656479 |
| ADAMDEC1 | -3.7256642 | -0.4063282 | 24.1250745 | 9.03E-07 | 1.94E-05 | 1.16936023 | 0.13010453 |
| SIK1 | -3.7228023 | 0.20690213 | 46.9388455 | 7.32E-12 | 3.49E-10 | 1.59919791 | 0.21048697 |
| ISG20 | -3.722576 | 3.99280516 | 52.2444147 | 4.90E-13 | 2.64E-11 | 4.83909643 | 1.73033261 |
| SOWAHD | -3.7215649 | -0.8268648 | 21.2941444 | 3.94E-06 | 7.69E-05 | 0.95552438 | 0.10084747 |
| PGBD5 | -3.7211655 | -1.5787891 | 18.3852146 | 1.80E-05 | 0.00029577 | 0.60175802 | 0.05040241 |
| GPR27 | -3.7037847 | -1.5595797 | 20.5837055 | 5.71E-06 | 0.00010729 | 0.5818542 | 0.05040241 |
| PCDHB2 | -3.6929622 | 1.95551588 | 65.6015036 | 5.52E-16 | 4.08E-14 | 3.03073521 | 0.63961908 |
| CSF2RB | -3.688186 | 1.68766217 | 32.919126 | 9.61E-09 | 2.92E-07 | 2.6526377 | 0.55261145 |
| KCNC3 | -3.6777808 | -0.46359 | 43.0722049 | 5.28E-11 | 2.20E-09 | 1.19382477 | 0.13035507 |
| SAA1 | -3.6691202 | 0.63048388 | 19.8575781 | 8.34E-06 | 0.00015166 | 1.75315582 | 0.29692093 |
| SCG2 | -3.6642783 | 4.5887456 | 127.734137 | 1.28E-29 | 3.45E-27 | 5.54050475 | 2.22585227 |
| FMO3 | -3.6631673 | -1.3014863 | 29.7358511 | 4.95E-08 | 1.35E-06 | 0.72175631 | 0.06717951 |
| SUN3 | -3.6461072 | -2.5162291 | 11.2911225 | 0.00077879 | 0.00793835 | 0.26467386 | 0.01740964 |
| OAS2 | -3.6457027 | 7.42772646 | 141.208328 | 1.45E-32 | 5.43E-30 | 8.36026202 | 4.7917874 |
| CYTL1 | -3.6430459 | 3.03001101 | 131.394891 | 2.03E-30 | 6.09E-28 | 4.05222899 | 1.17059749 |
| CCDC81 | -3.6351616 | 1.63325057 | 102.157486 | 5.13E-24 | 8.41E-22 | 2.77831439 | 0.5543454 |
| BEX2 | -3.6329425 | -0.3610442 | 34.8930096 | 3.48E-09 | 1.12E-07 | 1.24830278 | 0.14719844 |
| GALNT17 | -3.6279852 | -0.3736465 | 38.7116093 | 4.91E-10 | 1.81E-08 | 1.24554151 | 0.14445802 |
| ADAM28 | -3.622605 | -1.2765768 | 11.5761538 | 0.00066803 | 0.006992 | 0.66423271 | 0.0672118 |
| HOXB9 | -3.6173083 | 0.4583759 | 51.4398085 | 7.38E-13 | 3.89E-11 | 1.80766748 | 0.26517346 |
| CDH3 | -3.6099426 | -0.5255384 | 56.3968274 | 5.92E-14 | 3.66E-12 | 1.14758722 | 0.13341604 |
| SERPINA9 | -3.6051641 | 1.23460338 | 72.8120672 | 1.43E-17 | 1.26E-15 | 2.42231347 | 0.44239516 |
| BTLA | -3.5940438 | -2.0396385 | 18.4206119 | 1.77E-05 | 0.00029169 | 0.43356841 | 0.03425775 |
| DNAH3 | -3.5766534 | -0.1367069 | 29.2008287 | 6.53E-08 | 1.74E-06 | 1.35782491 | 0.1776796 |
| KANK4 | -3.5750436 | -0.0369808 | 49.7107288 | 1.78E-12 | 8.93E-11 | 1.46325795 | 0.18903393 |
| OAS3 | -3.5705484 | 5.53629991 | 24.4231221 | 7.73E-07 | 1.69E-05 | 6.06534218 | 3.06307742 |
| PLVAP | -3.5659527 | -2.563503 | 12.9356535 | 0.00032238 | 0.00374585 | 0.25623882 | 0.01740964 |
| TSPAN13 | -3.5428376 | 3.08791684 | 71.78973 | 2.39E-17 | 2.07E-15 | 4.03767711 | 1.25507622 |
| PDE10A | -3.5357869 | 1.78346209 | 37.8500103 | 7.64E-10 | 2.74E-08 | 2.75609524 | 0.63811202 |
| CST1 | -3.5348779 | -1.4231449 | 20.8819451 | 4.88E-06 | 9.34E-05 | 0.67479451 | 0.06781205 |
| STK32A | -3.5131837 | -2.0870856 | 19.0771347 | 1.26E-05 | 0.000215 | 0.41191396 | 0.03425775 |
| SLCO2A1 | -3.5127927 | -1.705447 | 20.3294507 | 6.52E-06 | 0.00012113 | 0.53407354 | 0.05044505 |
| ANKRD1 | -3.512073 | 9.25564151 | 142.106582 | 9.22E-33 | 3.52E-30 | 10.1829126 | 6.70614764 |
| RIMKLA | -3.4971079 | -1.4469324 | 20.8399229 | 4.99E-06 | 9.52E-05 | 0.66576722 | 0.06843413 |
| IGF2-AS | -3.4940428 | -1.1873964 | 26.6662149 | 2.42E-07 | 5.80E-06 | 0.7866542 | 0.08300327 |
| BIRC3 | -3.4920067 | 2.0622974 | 89.7279885 | 2.73E-21 | 3.56E-19 | 3.12115393 | 0.76763533 |
| IL6 | -3.4890181 | 7.87942204 | 130.985316 | 2.49E-30 | 7.38E-28 | 8.79742251 | 5.37263194 |
| LINC01504 | -3.4889133 | -2.1458962 | 10.9894975 | 0.0009163 | 0.00907052 | 0.39134176 | 0.03420061 |
| PDZD2 | -3.4831772 | 0.58097743 | 24.5497171 | 7.24E-07 | 1.60E-05 | 1.75410639 | 0.3107499 |
| CCR1 | -3.4812494 | 0.88422798 | 37.7589792 | 8.00E-10 | 2.85E-08 | 2.04138922 | 0.38162483 |
| ELF3 | -3.4713372 | -2.6135487 | 12.6151017 | 0.00038264 | 0.00432865 | 0.24572379 | 0.01676674 |
| SCN11A | -3.469966 | -2.6126181 | 12.5997372 | 0.0003858 | 0.00435968 | 0.24572379 | 0.01740964 |
| TMEM273 | -3.4677544 | -2.1148612 | 17.3645141 | 3.09E-05 | 0.00047284 | 0.37977578 | 0.0348435 |
| TMEM178B | -3.4569622 | 2.68773593 | 51.6857501 | 6.51E-13 | 3.45E-11 | 3.63113414 | 1.0672931 |
| RSPO3 | -3.4543442 | 3.84372944 | 116.565602 | 3.57E-27 | 7.82E-25 | 4.80160503 | 1.80884485 |
| IL1B | -3.4520163 | -1.4372468 | 14.7261785 | 0.00012431 | 0.00162514 | 0.61691245 | 0.06605188 |
| FGF11 | -3.4465185 | 1.09201657 | 54.4101 | 1.63E-13 | 9.28E-12 | 2.29933003 | 0.43186913 |
| BEST3 | -3.4413599 | -1.0158225 | 33.6311706 | 6.66E-09 | 2.07E-07 | 0.86712171 | 0.10030963 |
| CXCL2 | -3.4196684 | 3.97084334 | 142.414649 | 7.89E-33 | 3.07E-30 | 4.9377285 | 1.92642276 |
| SAMD9 | -3.4135397 | 6.12778064 | 29.8972628 | 4.56E-08 | 1.25E-06 | 6.77168522 | 3.72780851 |
| CA9 | -3.4124436 | -0.6983609 | 22.6262572 | 1.97E-06 | 4.05E-05 | 1.02255648 | 0.13238073 |
| GAL | -3.4024977 | 1.08751345 | 23.4746003 | 1.27E-06 | 2.68E-05 | 2.16172594 | 0.45050334 |
| PTGDS | -3.4002212 | 8.64315312 | 29.3492646 | 6.04E-08 | 1.62E-06 | 9.23586674 | 6.19803781 |
| CYP7B1 | -3.3902133 | 1.09437709 | 61.6621228 | 4.08E-15 | 2.83E-13 | 2.27852242 | 0.46020761 |
| ENPEP | -3.3896316 | 1.74087556 | 29.9112627 | 4.52E-08 | 1.25E-06 | 2.66398501 | 0.67213802 |
| GREB1L | -3.3848564 | -1.2705977 | 30.6319354 | 3.12E-08 | 8.83E-07 | 0.72891321 | 0.08123082 |
| ACSM5 | -3.3741292 | 2.00742831 | 86.5726172 | 1.35E-20 | 1.60E-18 | 3.07930265 | 0.78275841 |
| CAMK1G | -3.3698874 | -1.8127525 | 24.9331968 | 5.94E-07 | 1.33E-05 | 0.50287372 | 0.05164482 |
| ANXA8 | -3.3627396 | -0.9228262 | 20.2876603 | 6.66E-06 | 0.00012369 | 0.91133938 | 0.11279339 |
| LINC01483 | -3.3561095 | -2.6740022 | 11.2900377 | 0.00077924 | 0.00793835 | 0.22488447 | 0.01676674 |
| IL36RN | -3.3532614 | -2.1950397 | 17.7697678 | 2.49E-05 | 0.00038919 | 0.35853938 | 0.03296855 |
| ACTA2 | -3.3433753 | 9.8042883 | 108.407707 | 2.19E-25 | 4.21E-23 | 10.7026985 | 7.40102642 |
| IL17C | -3.341063 | -2.6823947 | 11.1131458 | 0.00085718 | 0.00858243 | 0.21520296 | 0.01743386 |
| LINC01583 | -3.3369302 | -1.5449474 | 30.5106434 | 3.32E-08 | 9.35E-07 | 0.61250497 | 0.06843413 |
| CD200 | -3.3205431 | 0.68968635 | 18.0773244 | 2.12E-05 | 0.00033929 | 1.82888549 | 0.35641961 |
| HCP5 | -3.3094038 | 2.0146025 | 102.413578 | 4.51E-24 | 7.45E-22 | 3.08544428 | 0.81649301 |
| TCIM | -3.3009058 | -1.858661 | 19.7639935 | 8.76E-06 | 0.00015777 | 0.47387804 | 0.05044505 |
| MMP8 | -3.2982224 | 2.52157623 | 39.7921112 | 2.82E-10 | 1.09E-08 | 3.42303152 | 1.05715528 |
| IFI44L | -3.2959479 | 6.91592018 | 90.9212152 | 1.50E-21 | 2.01E-19 | 7.79949965 | 4.59938982 |
| BLNK | -3.2903044 | -1.1273379 | 30.503675 | 3.33E-08 | 9.37E-07 | 0.8011357 | 0.10095796 |
| GBP1 | -3.2891394 | 6.57765385 | 66.6869798 | 3.18E-16 | 2.47E-14 | 7.40578235 | 4.29274613 |
| VMO1 | -3.2854822 | 4.86176451 | 21.420021 | 3.69E-06 | 7.23E-05 | 5.3644999 | 2.72964167 |
| AMIGO2 | -3.2763159 | 6.98860657 | 89.7081581 | 2.76E-21 | 3.58E-19 | 7.86034219 | 4.69525424 |
| ACTR3C | -3.2726176 | -1.8873399 | 17.2004449 | 3.36E-05 | 0.0005099 | 0.48119416 | 0.05164482 |
| PARP12 | -3.2706015 | 5.66213458 | 107.784124 | 3.00E-25 | 5.62E-23 | 6.56829678 | 3.45028637 |
| COL6A6 | -3.2670137 | 1.31463367 | 34.6426161 | 3.96E-09 | 1.25E-07 | 2.38368106 | 0.55246911 |
| A1BG-AS1 | -3.2653962 | -1.1377362 | 19.7419002 | 8.86E-06 | 0.00015947 | 0.76250885 | 0.0990873 |
| CCL3L3 | -3.2622494 | 0.06430847 | 33.1362434 | 8.59E-09 | 2.62E-07 | 1.48588387 | 0.2551829 |
| RASGEF1B | -3.255788 | 1.02510341 | 52.4558842 | 4.40E-13 | 2.38E-11 | 2.20877061 | 0.46932546 |
| PAX1 | -3.2431377 | -1.9034569 | 20.6205753 | 5.60E-06 | 0.00010543 | 0.4482398 | 0.05161024 |
| TCAF2 | -3.2430168 | 1.65693149 | 54.7495274 | 1.37E-13 | 7.96E-12 | 2.74986206 | 0.67763096 |
| APOL1 | -3.2412085 | 7.31508588 | 73.1779788 | 1.18E-17 | 1.05E-15 | 8.15704408 | 5.04204544 |
| DSG3 | -3.2395822 | 0.43726536 | 36.422875 | 1.59E-09 | 5.42E-08 | 1.74151948 | 0.32288384 |
| GPR84 | -3.2363524 | -0.8381667 | 29.4372177 | 5.78E-08 | 1.56E-06 | 0.95398547 | 0.12898069 |
| CFB | -3.2216597 | 6.17127807 | 109.577607 | 1.21E-25 | 2.45E-23 | 7.06829207 | 3.96467725 |
| HCG4 | -3.2215126 | -1.003537 | 34.1780347 | 5.03E-09 | 1.58E-07 | 0.87119362 | 0.11652116 |
| PGF | -3.2160296 | 4.33776786 | 37.2062582 | 1.06E-09 | 3.73E-08 | 5.10737505 | 2.33518949 |
| CCL2 | -3.2090415 | 7.52707631 | 122.895383 | 1.47E-28 | 3.67E-26 | 8.42885587 | 5.27540465 |
| IL15RA | -3.1964291 | 2.82014806 | 80.6943861 | 2.63E-19 | 2.81E-17 | 3.81828957 | 1.2681604 |
| SERPINB2 | -3.1935121 | 4.57524094 | 32.0038666 | 1.54E-08 | 4.57E-07 | 5.26827647 | 2.55924356 |
| SLCO2B1 | -3.1832817 | -1.4150174 | 29.9091111 | 4.53E-08 | 1.25E-06 | 0.66537513 | 0.08472387 |
| HLA-DQA2 | -3.1805639 | -1.9682442 | 12.0619251 | 0.00051462 | 0.00555812 | 0.44557414 | 0.04864225 |
| ANGPTL1 | -3.1771639 | 2.63892759 | 66.2850079 | 3.90E-16 | 2.98E-14 | 3.5983904 | 1.1903146 |
| LINC00623 | -3.1687242 | 1.86724902 | 45.3697014 | 1.63E-11 | 7.38E-10 | 2.90158065 | 0.79769465 |
| GPRC5B | -3.1618066 | 1.24681173 | 66.0285952 | 4.44E-16 | 3.34E-14 | 2.38910587 | 0.56990388 |
| KIAA0040 | -3.1583612 | 1.19880951 | 51.403865 | 7.52E-13 | 3.96E-11 | 2.35893068 | 0.54313124 |
| CHI3L2 | -3.1565722 | 3.87886241 | 67.6381882 | 1.96E-16 | 1.56E-14 | 4.76849185 | 2.03252991 |
| OXTR | -3.1492439 | 4.69391024 | 122.722529 | 1.60E-28 | 3.96E-26 | 5.61830793 | 2.69632671 |
| ASPA | -3.1442979 | -1.6836857 | 24.4021372 | 7.82E-07 | 1.71E-05 | 0.55230947 | 0.06607611 |
| CCN6 | -3.1439405 | 2.65914747 | 89.4295064 | 3.18E-21 | 4.04E-19 | 3.64892733 | 1.22174442 |
| HELZ2 | -3.1438528 | 6.47014373 | 55.9958122 | 7.26E-14 | 4.39E-12 | 7.28884787 | 4.29532426 |
| EGLN3 | -3.1424559 | -0.5087156 | 36.7964354 | 1.31E-09 | 4.54E-08 | 1.14216813 | 0.17762625 |
| HLA-DPA1 | -3.1350385 | 3.81757007 | 112.603273 | 2.64E-26 | 5.48E-24 | 4.76230624 | 1.99925739 |
| HSD17B6 | -3.1349666 | 2.57907664 | 68.822005 | 1.08E-16 | 8.84E-15 | 3.57265006 | 1.16232653 |
| P2RX5 | -3.127151 | -0.7793037 | 29.4049271 | 5.87E-08 | 1.58E-06 | 0.97556762 | 0.14854505 |
| DDX60L | -3.126945 | 6.00943789 | 44.199339 | 2.97E-11 | 1.29E-09 | 6.80081259 | 3.858989 |
| CCDC3 | -3.1224716 | -0.2130436 | 55.7916409 | 8.06E-14 | 4.85E-12 | 1.32448679 | 0.22663971 |
| INA | -3.1132119 | 1.90464188 | 71.8309782 | 2.34E-17 | 2.03E-15 | 2.95124972 | 0.84387298 |
| WFDC21P | -3.0969154 | 3.99491328 | 17.2337452 | 3.31E-05 | 0.0005025 | 4.46900672 | 2.15276836 |
| LINC00323 | -3.0909938 | -0.5677054 | 12.3549152 | 0.00043983 | 0.0048938 | 1.04433971 | 0.1776796 |
| XAF1 | -3.0879328 | 5.6597585 | 112.669681 | 2.55E-26 | 5.35E-24 | 6.55507348 | 3.60320452 |
| LINC02015 | -3.0857827 | 0.1591336 | 49.4562553 | 2.03E-12 | 1.00E-10 | 1.56833177 | 0.29778695 |
| SECTM1 | -3.0835337 | 4.29028876 | 39.5534396 | 3.19E-10 | 1.22E-08 | 5.07259479 | 2.40156381 |
| IER3 | -3.0823773 | 7.94027158 | 79.2990262 | 5.34E-19 | 5.51E-17 | 8.78915103 | 5.79264016 |
| GATD3A | -3.0793628 | 2.3525037 | 22.6004282 | 1.99E-06 | 4.10E-05 | 3.1136658 | 1.08812212 |
| PKNOX2 | -3.0789721 | 0.72920141 | 74.0746757 | 7.52E-18 | 7.02E-16 | 1.99225389 | 0.4339354 |
| NR2F2-AS1 | -3.0764931 | -0.9556738 | 31.840508 | 1.67E-08 | 4.94E-07 | 0.88396245 | 0.13392651 |
| STC2 | -3.0666967 | 10.2260744 | 106.236441 | 6.54E-25 | 1.19E-22 | 11.1075715 | 8.07222457 |
| MRVI1 | -3.0660145 | 2.91087269 | 74.8738145 | 5.02E-18 | 4.75E-16 | 3.88975832 | 1.39585429 |
| CLIC3 | -3.0643655 | 4.41955218 | 17.2941834 | 3.20E-05 | 0.00048924 | 4.86115476 | 2.52854775 |
| CPA4 | -3.063767 | 5.33844504 | 121.332394 | 3.23E-28 | 7.89E-26 | 6.2423799 | 3.33047679 |
| ITGB6 | -3.0629499 | -0.3576724 | 38.2571488 | 6.20E-10 | 2.24E-08 | 1.20602974 | 0.2109761 |
| MT1M | -3.0513407 | 3.33810883 | 26.3235926 | 2.89E-07 | 6.80E-06 | 4.04172084 | 1.71248677 |
| MT1L | -3.0424842 | 3.93086603 | 17.7630147 | 2.50E-05 | 0.00039028 | 4.45231224 | 2.13220414 |
| SLC6A13 | -3.0397575 | -2.3783781 | 12.638217 | 0.00037794 | 0.00429164 | 0.28658251 | 0.03420061 |
| NRXN3 | -3.0292347 | 3.80875431 | 35.4798232 | 2.58E-09 | 8.45E-08 | 4.59460603 | 2.04820517 |
| ZIC4 | -3.0225768 | 0.58468967 | 33.1483125 | 8.54E-09 | 2.61E-07 | 1.84448938 | 0.39683247 |
| SCHLAP1 | -3.0160839 | -1.1491612 | 16.0195961 | 6.27E-05 | 0.00089177 | 0.74642979 | 0.1127466 |
| MSC | -3.0150696 | 5.60723839 | 77.8531721 | 1.11E-18 | 1.10E-16 | 6.46383315 | 3.61336927 |
| XIRP1 | -3.0067835 | -1.3422887 | 24.5728227 | 7.16E-07 | 1.58E-05 | 0.6752453 | 0.10146955 |
| TLR3 | -3.0021856 | 3.8032238 | 12.8243697 | 0.00034213 | 0.00394912 | 4.21070219 | 2.01167479 |
| ROR1-AS1 | -2.9935061 | 1.50615551 | 22.533719 | 2.06E-06 | 4.23E-05 | 2.47244036 | 0.69885786 |
| AK4 | -2.9846964 | 6.36900216 | 94.7734195 | 2.13E-22 | 3.11E-20 | 7.23892546 | 4.3511467 |
| CSRP2 | -2.977346 | 3.4863475 | 67.0659694 | 2.63E-16 | 2.06E-14 | 4.39141377 | 1.85524587 |
| PTPRO | -2.9761087 | -0.3166397 | 15.7992364 | 7.04E-05 | 0.0009865 | 1.21995415 | 0.21487616 |
| IRF7 | -2.9759822 | 5.73556165 | 38.605799 | 5.19E-10 | 1.90E-08 | 6.4701815 | 3.76041486 |
| AKAP3 | -2.9668434 | -0.4404187 | 44.4782977 | 2.57E-11 | 1.13E-09 | 1.1605646 | 0.21048697 |
| IBSP | -2.9617514 | 1.20696828 | 47.230158 | 6.31E-12 | 3.02E-10 | 2.31025346 | 0.61724379 |
| KLHL30 | -2.9513262 | -1.2115033 | 22.2843796 | 2.35E-06 | 4.76E-05 | 0.75298968 | 0.11563866 |
| NSUN7 | -2.9472708 | -1.5911672 | 23.5915755 | 1.19E-06 | 2.52E-05 | 0.56591866 | 0.08461337 |
| TNFRSF9 | -2.9457025 | -1.3494533 | 11.8433058 | 0.00057869 | 0.0061551 | 0.63514784 | 0.10040672 |
| ANO3 | -2.9426123 | -0.2668289 | 14.7928117 | 0.00011999 | 0.00157939 | 1.17177693 | 0.23422408 |
| TPTEP1 | -2.9415518 | -0.7851671 | 27.8599379 | 1.30E-07 | 3.32E-06 | 0.95818596 | 0.16091789 |
| NKX6-1 | -2.9412713 | 1.11762209 | 69.7462914 | 6.74E-17 | 5.58E-15 | 2.28712189 | 0.58785441 |
| LINC02432 | -2.9376751 | -1.0515582 | 22.2143139 | 2.44E-06 | 4.92E-05 | 0.79098755 | 0.13341604 |
| SLITRK6 | -2.9365943 | 0.62224212 | 19.0631346 | 1.26E-05 | 0.00021623 | 1.74872483 | 0.43209539 |
| ADRA2A | -2.9314362 | 0.09966728 | 44.1580749 | 3.03E-11 | 1.32E-09 | 1.4976578 | 0.31259172 |
| GLDN | -2.9298217 | -1.6329147 | 11.4268669 | 0.0007239 | 0.00748347 | 0.56188837 | 0.08123082 |
| HCG26 | -2.9271922 | -1.0745113 | 20.8196808 | 5.05E-06 | 9.61E-05 | 0.81874664 | 0.1323855 |
| ATG9B | -2.9270043 | -0.6788607 | 35.7726946 | 2.22E-09 | 7.43E-08 | 1.03124895 | 0.18056132 |
| ARFGEF3 | -2.9265307 | 0.57150617 | 18.5394734 | 1.66E-05 | 0.00027644 | 1.73763252 | 0.4160092 |
| OLFM2 | -2.915269 | 2.99568052 | 70.7714253 | 4.01E-17 | 3.37E-15 | 3.94840303 | 1.53723032 |
| EPSTI1 | -2.9069883 | 5.77005339 | 106.950097 | 4.57E-25 | 8.48E-23 | 6.6481817 | 3.8539735 |
| NEFL | -2.9026143 | -0.8338345 | 16.8722181 | 4.00E-05 | 0.00059495 | 0.93952667 | 0.15918195 |
| ODF3B | -2.8994114 | 3.4268202 | 19.2460889 | 1.15E-05 | 0.00019972 | 4.10004781 | 1.8184374 |
| CXCL16 | -2.8961819 | 4.60991639 | 96.5776557 | 8.58E-23 | 1.30E-20 | 5.50258327 | 2.81447627 |
| SYNGR2 | -2.8943077 | 4.73961868 | 46.7116589 | 8.22E-12 | 3.86E-10 | 5.54593069 | 2.92092262 |
| PDZD4 | -2.8924913 | 0.59220001 | 20.133914 | 7.22E-06 | 0.0001331 | 1.75838398 | 0.44662549 |
| MROCKI | -2.8802696 | 0.92918647 | 66.4369301 | 3.61E-16 | 2.77E-14 | 2.1263455 | 0.54612455 |
| CH25H | -2.8769077 | 3.98825959 | 84.8066155 | 3.29E-20 | 3.66E-18 | 4.87956375 | 2.31556382 |
| C7orf69 | -2.8744431 | -2.1386654 | 13.7053136 | 0.00021385 | 0.00259986 | 0.37152972 | 0.05037818 |
| ANKRD20A5P | -2.8736704 | -1.4438121 | 22.8923325 | 1.71E-06 | 3.55E-05 | 0.61943036 | 0.10028707 |
| CCND2 | -2.8619484 | 3.24408022 | 63.0497511 | 2.02E-15 | 1.44E-13 | 4.14351928 | 1.75968833 |
| ART3 | -2.8607162 | -0.9795794 | 27.1246823 | 1.91E-07 | 4.70E-06 | 0.86258328 | 0.14649973 |
| PARP10 | -2.8600889 | 6.6778057 | 38.2932986 | 6.09E-10 | 2.20E-08 | 7.39891975 | 4.7549786 |
| HLA-DPB1 | -2.8560282 | 3.29435864 | 28.7162684 | 8.38E-08 | 2.21E-06 | 4.11804041 | 1.75202329 |
| SALL1 | -2.8525116 | -0.0905398 | 20.8520117 | 4.96E-06 | 9.47E-05 | 1.33074022 | 0.27803598 |
| C3orf80 | -2.8485716 | 0.28563504 | 24.3026106 | 8.23E-07 | 1.79E-05 | 1.56406409 | 0.36532048 |
| CTSS | -2.8467451 | 4.58048444 | 63.6556211 | 1.48E-15 | 1.07E-13 | 5.42528906 | 2.8270583 |
| TACSTD2 | -2.8275437 | 0.40883964 | 45.3318528 | 1.66E-11 | 7.51E-10 | 1.7275868 | 0.39752143 |
| EGFL7 | -2.8185627 | 4.60356503 | 18.516519 | 1.68E-05 | 0.00027935 | 5.13582998 | 2.85947123 |
| UBL4B | -2.8156684 | -0.6667639 | 25.0218549 | 5.67E-07 | 1.28E-05 | 1.02240886 | 0.19530803 |
| ZDHHC15 | -2.8012941 | 0.38942724 | 22.0195124 | 2.70E-06 | 5.40E-05 | 1.66523435 | 0.38930864 |
| IFITM1 | -2.8002867 | 8.23739318 | 27.4578546 | 1.61E-07 | 4.01E-06 | 8.88389477 | 6.31198388 |
| ARHGEF16 | -2.7972491 | 1.64955386 | 57.0440449 | 4.26E-14 | 2.66E-12 | 2.70257985 | 0.85379576 |
| ZSWIM5 | -2.7892297 | 0.28675425 | 31.420378 | 2.08E-08 | 6.05E-07 | 1.60207617 | 0.37732349 |
| MCF2L2 | -2.788099 | -2.1907428 | 13.5822555 | 0.00022833 | 0.00275573 | 0.34938235 | 0.05040241 |
| MIR210HG | -2.7693575 | 3.44970106 | 51.0096846 | 9.19E-13 | 4.76E-11 | 4.30565986 | 1.96880522 |
| CPA3 | -2.7677835 | -0.3299621 | 46.9216247 | 7.39E-12 | 3.50E-10 | 1.22425738 | 0.25517212 |
| CDH1 | -2.7517755 | -0.8144943 | 18.2662683 | 1.92E-05 | 0.00031214 | 0.91684491 | 0.17416944 |
| CYTOR | -2.7487188 | 6.61840884 | 18.2238663 | 1.96E-05 | 0.00031843 | 7.12144036 | 4.76494492 |
| IFI35 | -2.7476262 | 6.69403173 | 23.2430152 | 1.43E-06 | 3.00E-05 | 7.28480242 | 4.85339554 |
| RRAD | -2.7353082 | 1.85459704 | 23.4908253 | 1.26E-06 | 2.66E-05 | 2.74572506 | 0.97974182 |
| SEPTIN3 | -2.7293391 | -1.2157826 | 17.2751733 | 3.23E-05 | 0.00049309 | 0.71417102 | 0.12898069 |
| KRT19 | -2.7211256 | 5.72721816 | 19.4988702 | 1.01E-05 | 0.0001779 | 6.26559317 | 3.95238792 |
| FILIP1 | -2.7205592 | -0.8231199 | 13.0508315 | 0.00030315 | 0.00355002 | 0.85980333 | 0.17943517 |
| SIK1B | -2.7188542 | 3.93009139 | 87.8432769 | 7.09E-21 | 8.65E-19 | 4.81906091 | 2.37773896 |
| RENBP | -2.716796 | 3.97319691 | 17.7166019 | 2.56E-05 | 0.00039903 | 4.52972431 | 2.40834754 |
| FAM241B | -2.7132961 | 0.40896733 | 27.8273404 | 1.33E-07 | 3.36E-06 | 1.70260338 | 0.42496835 |
| GSTA1 | -2.6997606 | -1.9731321 | 15.1579305 | 9.89E-05 | 0.00133085 | 0.42988128 | 0.06846871 |
| AP3B2 | -2.6986618 | -0.2319325 | 26.7474382 | 2.32E-07 | 5.56E-06 | 1.28024522 | 0.27858067 |
| PALMD | -2.6969238 | 2.51583247 | 35.6159079 | 2.40E-09 | 7.98E-08 | 3.41782995 | 1.34593017 |
| PDE1C | -2.6960467 | 5.47621397 | 46.0999247 | 1.12E-11 | 5.19E-10 | 6.24882844 | 3.7492211 |
| NLGN4X | -2.6956415 | 1.84192238 | 24.65655 | 6.85E-07 | 1.52E-05 | 2.76094635 | 0.96979329 |
| ACPP | -2.6904662 | -0.7554074 | 24.2971768 | 8.26E-07 | 1.79E-05 | 0.9563836 | 0.19329995 |
| DNER | -2.6871605 | 2.36015414 | 36.8362189 | 1.28E-09 | 4.45E-08 | 3.2547809 | 1.27321026 |
| RETREG1 | -2.6850036 | 2.97117605 | 55.557675 | 9.08E-14 | 5.40E-12 | 3.85715499 | 1.67679888 |
| TRIL | -2.6788411 | 0.48494118 | 39.8251701 | 2.78E-10 | 1.07E-08 | 1.7466577 | 0.45919371 |
| PAX8 | -2.6786843 | 2.50964744 | 39.9659861 | 2.58E-10 | 1.00E-08 | 3.41866191 | 1.36625342 |
| NXPH3 | -2.6781243 | 3.9424959 | 34.7589005 | 3.73E-09 | 1.19E-07 | 4.70776841 | 2.4157528 |
| LINC01114 | -2.6776538 | -0.3225324 | 30.9269017 | 2.68E-08 | 7.67E-07 | 1.22386239 | 0.26525407 |
| PADI1 | -2.6723709 | 1.85759533 | 31.5156075 | 1.98E-08 | 5.79E-07 | 2.81978774 | 1.00039261 |
| MTTP | -2.6703482 | -1.5703035 | 11.8405034 | 0.00057956 | 0.00616125 | 0.55948153 | 0.09812298 |
| QPRT | -2.6692863 | 5.96130793 | 56.2865317 | 6.26E-14 | 3.86E-12 | 6.75430725 | 4.22980943 |
| SYT1 | -2.668645 | 1.16361299 | 43.1089002 | 5.18E-11 | 2.17E-09 | 2.25783765 | 0.69475762 |
| FIBCD1 | -2.6633366 | 3.10504198 | 43.8898469 | 3.47E-11 | 1.50E-09 | 3.97884891 | 1.76868348 |
| MYOZ2 | -2.661903 | -0.4983863 | 33.1719035 | 8.44E-09 | 2.58E-07 | 1.10435547 | 0.23708284 |
| DEUP1 | -2.6610728 | -2.0035719 | 14.4468442 | 0.00014417 | 0.001848 | 0.39972386 | 0.06727965 |
| IL1RL1 | -2.6566558 | -0.4219602 | 22.9666283 | 1.65E-06 | 3.43E-05 | 1.15493664 | 0.2535105 |
| DPP4 | -2.6566198 | 6.61912665 | 42.8810985 | 5.82E-11 | 2.41E-09 | 7.36634804 | 4.8668418 |
| SPAG4 | -2.6545387 | 1.77300729 | 19.4172517 | 1.05E-05 | 0.0001852 | 2.65568847 | 0.96415193 |
| FUOM | -2.6528833 | 3.23557421 | 14.4505389 | 0.00014389 | 0.0018455 | 3.80007725 | 1.8570248 |
| WNT16 | -2.6524402 | 2.63919786 | 16.0007142 | 6.33E-05 | 0.00089888 | 3.27927155 | 1.46744195 |
| SLC22A1 | -2.6453656 | -0.6949763 | 25.6093413 | 4.18E-07 | 9.61E-06 | 0.99489992 | 0.20896733 |
| LGR5 | -2.6425492 | -0.8938937 | 19.3578062 | 1.08E-05 | 0.00018994 | 0.85105767 | 0.17894236 |
| GATA6 | -2.6423079 | 4.14932357 | 57.3002641 | 3.74E-14 | 2.38E-12 | 4.98181436 | 2.61259918 |
| HTATSF1P2 | -2.6388612 | 2.7701228 | 13.7458906 | 0.00020928 | 0.00255317 | 3.33690541 | 1.55757019 |
| CACNA2D3 | -2.6381529 | 0.72233797 | 45.7784918 | 1.32E-11 | 6.03E-10 | 1.94305883 | 0.53977452 |
| TRIM29 | -2.6310218 | -0.1528789 | 20.0398581 | 7.58E-06 | 0.00013898 | 1.32388937 | 0.30125688 |
| PRXL2A | -2.6243217 | 3.20103987 | 65.0047122 | 7.47E-16 | 5.50E-14 | 4.10899826 | 1.86072222 |
| TRIM14 | -2.6238691 | 2.97603858 | 41.4418627 | 1.21E-10 | 4.89E-09 | 3.82921021 | 1.70934046 |
| ADGRL3 | -2.6215917 | -0.7990619 | 14.1836841 | 0.0001658 | 0.00208954 | 0.88477084 | 0.19138187 |
| HIST1H1C | -2.6197047 | 5.49658809 | 20.5251989 | 5.89E-06 | 0.00011023 | 6.05970446 | 3.83214358 |
| SHC2 | -2.615408 | -1.1744894 | 18.1208501 | 2.07E-05 | 0.0003333 | 0.75044295 | 0.14852399 |
| GALNT18 | -2.6148829 | 3.46057553 | 71.7255442 | 2.47E-17 | 2.13E-15 | 4.35724822 | 2.06528386 |
| LINC01436 | -2.6124789 | -0.6285461 | 27.3404185 | 1.71E-07 | 4.24E-06 | 1.03300422 | 0.22334094 |
| SLC16A6 | -2.6113341 | 2.55082253 | 40.8063232 | 1.68E-10 | 6.63E-09 | 3.43282524 | 1.43548326 |
| BHLHE41 | -2.6082475 | 4.64256561 | 55.6652672 | 8.59E-14 | 5.14E-12 | 5.45400702 | 3.06469251 |
| EPHX4 | -2.6050164 | -0.2294214 | 16.2647677 | 5.51E-05 | 0.00079644 | 1.27190278 | 0.28302749 |
| ALMS1P1 | -2.6010708 | -1.6305856 | 19.8370165 | 8.43E-06 | 0.00015304 | 0.55070175 | 0.09866468 |
| INHBE | -2.6007105 | 0.41922721 | 52.6935872 | 3.90E-13 | 2.13E-11 | 1.71621117 | 0.45958656 |
| TIGD3 | -2.5807613 | -2.3032775 | 10.8121307 | 0.00100837 | 0.00983362 | 0.31845771 | 0.05164482 |
| USP2-AS1 | -2.5778504 | -2.3041379 | 11.3383948 | 0.00075921 | 0.00778338 | 0.31845771 | 0.05161024 |
| CNN1 | -2.5749266 | 3.45319908 | 41.8568361 | 9.82E-11 | 4.00E-09 | 4.27259885 | 2.09910547 |
| RORB | -2.5742543 | -0.3325933 | 34.4087228 | 4.47E-09 | 1.41E-07 | 1.19694616 | 0.28148142 |
| FBXO16 | -2.5724211 | 0.39060703 | 14.3201125 | 0.00015421 | 0.00195517 | 1.69194614 | 0.43145475 |
| PNLIPRP3 | -2.5723181 | 0.49409895 | 23.7907768 | 1.07E-06 | 2.29E-05 | 1.73278329 | 0.4758616 |
| COL4A4 | -2.5708006 | 2.4213934 | 19.8214798 | 8.50E-06 | 0.00015389 | 3.18728702 | 1.36154856 |
| LCP1 | -2.5620978 | 2.90654647 | 68.4821154 | 1.28E-16 | 1.04E-14 | 3.82152479 | 1.69695225 |
| CCDC190 | -2.5574508 | 0.54933365 | 49.1802653 | 2.33E-12 | 1.15E-10 | 1.80604827 | 0.50887158 |
| SYNGR3 | -2.5566685 | -0.3538957 | 22.501411 | 2.10E-06 | 4.30E-05 | 1.17360178 | 0.28297483 |
| CD300C | -2.5481908 | 0.82446318 | 38.6407505 | 5.09E-10 | 1.87E-08 | 1.99895217 | 0.60439069 |
| LIMS3 | -2.546369 | -1.8577747 | 16.1261273 | 5.93E-05 | 0.00084758 | 0.44994588 | 0.08461337 |
| TEKT2 | -2.5455201 | -2.077834 | 13.3161026 | 0.00026314 | 0.00313021 | 0.37329377 | 0.06605188 |
| LINC00862 | -2.5412135 | -1.1075429 | 15.6947956 | 7.44E-05 | 0.00103556 | 0.7782202 | 0.1626931 |
| MGP | -2.5394855 | 7.76023961 | 32.1315857 | 1.44E-08 | 4.31E-07 | 8.45244488 | 6.07486792 |
| CNTN1 | -2.5303661 | -0.7702699 | 18.1027803 | 2.09E-05 | 0.00033545 | 0.90711825 | 0.21048697 |
| CYP26B1 | -2.5303025 | -1.3417964 | 12.2008053 | 0.00047769 | 0.00524558 | 0.61908058 | 0.13334505 |
| PLAAT4 | -2.5296599 | 3.76913995 | 15.417204 | 8.62E-05 | 0.00118679 | 4.31087989 | 2.37063104 |
| LRRC3 | -2.5281331 | 1.3436253 | 24.4382067 | 7.67E-07 | 1.68E-05 | 2.34467644 | 0.80697914 |
| APOBEC3G | -2.526696 | 3.55903104 | 85.1774567 | 2.73E-20 | 3.11E-18 | 4.44037497 | 2.21451384 |
| PRL | -2.518132 | -0.8893594 | 31.0964507 | 2.46E-08 | 7.07E-07 | 0.88301895 | 0.19414963 |
| BEGAIN | -2.5142698 | -0.6241893 | 21.3075299 | 3.91E-06 | 7.65E-05 | 1.02679718 | 0.23681818 |
| CELF5 | -2.5130695 | -2.0879481 | 12.5473324 | 0.00039677 | 0.00447162 | 0.38412616 | 0.06843413 |
| FBXO39 | -2.5057751 | -1.8804179 | 14.6521765 | 0.00012929 | 0.00167975 | 0.44902344 | 0.08463594 |
| MMP13 | -2.4997196 | 4.89618212 | 44.9165756 | 2.06E-11 | 9.19E-10 | 5.67284367 | 3.36857562 |
| VLDLR-AS1 | -2.4943174 | -0.0903576 | 17.6210713 | 2.70E-05 | 0.00041799 | 1.30873223 | 0.35541314 |
| LCNL1 | -2.4898589 | 3.10859002 | 19.9371819 | 8.00E-06 | 0.00014599 | 3.7894815 | 1.89589537 |
| REEP1 | -2.4896201 | 0.07916216 | 18.1487534 | 2.04E-05 | 0.00032871 | 1.45112008 | 0.38235022 |
| IL12RB1 | -2.4883242 | -1.7068401 | 13.2227297 | 0.00027658 | 0.00327707 | 0.51388683 | 0.10146955 |
| RNF224 | -2.4880301 | -1.1363428 | 20.5035567 | 5.95E-06 | 0.00011138 | 0.75748802 | 0.15834645 |
| RHCE | -2.4794686 | 0.6620658 | 43.2424979 | 4.84E-11 | 2.04E-09 | 1.86873769 | 0.56380101 |
| FAM89A | -2.4750225 | 3.18607504 | 62.2099315 | 3.09E-15 | 2.17E-13 | 4.0739769 | 1.94707097 |
| KRT7 | -2.4740234 | 6.60019808 | 19.0551309 | 1.27E-05 | 0.00021678 | 7.15528359 | 4.98406739 |
| GMPR | -2.4610656 | 4.6908282 | 44.9445863 | 2.03E-11 | 9.07E-10 | 5.47431247 | 3.21243203 |
| VCAM1 | -2.4469058 | 7.75105318 | 60.7315126 | 6.54E-15 | 4.44E-13 | 8.53587595 | 6.14994438 |
| HLA-L | -2.4451533 | 4.66072674 | 42.9477214 | 5.62E-11 | 2.34E-09 | 5.4294996 | 3.20493372 |
| LIF | -2.4445547 | 6.17059936 | 75.0479496 | 4.59E-18 | 4.38E-16 | 6.97871749 | 4.61146842 |
| HLA-B | -2.4379838 | 11.0206386 | 38.8728757 | 4.52E-10 | 1.68E-08 | 11.7452395 | 9.40859855 |
| CCL26 | -2.4366482 | 2.02868425 | 11.7572417 | 0.00060607 | 0.0064203 | 2.71059725 | 1.20046355 |
| MEST | -2.4279928 | 7.22175895 | 85.0694988 | 2.88E-20 | 3.25E-18 | 8.03357611 | 5.64691839 |
| SIGLEC9 | -2.4261126 | -1.9331361 | 13.9170278 | 0.00019106 | 0.0023597 | 0.41362517 | 0.08464566 |
| PCSK9 | -2.4239672 | 3.07218938 | 43.1561839 | 5.05E-11 | 2.12E-09 | 3.90615892 | 1.90704153 |
| TAP1 | -2.4231432 | 6.90429435 | 76.4013656 | 2.31E-18 | 2.26E-16 | 7.70763573 | 5.34030201 |
| PWP2 | -2.4167856 | 1.18179561 | 44.3088065 | 2.80E-11 | 1.23E-09 | 2.27410671 | 0.78753956 |
| HSPB7 | -2.4107557 | 6.24247908 | 39.1428988 | 3.94E-10 | 1.48E-08 | 6.9705589 | 4.70630499 |
| CARD9 | -2.4022195 | 2.35665053 | 19.7818158 | 8.68E-06 | 0.00015658 | 3.18086877 | 1.39898692 |
| TLDC2 | -2.3885526 | 4.70996671 | 35.78749 | 2.20E-09 | 7.38E-08 | 5.45585052 | 3.28359736 |
| CFAP43 | -2.3876358 | -0.0958868 | 29.3090778 | 6.17E-08 | 1.65E-06 | 1.32895377 | 0.36518678 |
| GSTT2B | -2.3865921 | -0.0584683 | 14.8655327 | 0.00011545 | 0.00152745 | 1.31361627 | 0.37905264 |
| JPH2 | -2.3822283 | 1.9897732 | 43.7592689 | 3.71E-11 | 1.59E-09 | 2.94734721 | 1.20733486 |
| AQP3 | -2.3821462 | -0.2303553 | 19.0119044 | 1.30E-05 | 0.00022139 | 1.22903796 | 0.33901786 |
| MAST4-AS1 | -2.3817447 | -1.7776571 | 14.3468324 | 0.00015204 | 0.00193228 | 0.46175054 | 0.10028707 |
| TRIM67 | -2.3786646 | -1.6015642 | 12.8434549 | 0.00033866 | 0.00391334 | 0.52896497 | 0.11666188 |
| GBP2 | -2.3785099 | 5.65802307 | 55.7788786 | 8.11E-14 | 4.86E-12 | 6.44381463 | 4.1698187 |
| PNMA6A | -2.3766879 | 1.72810727 | 38.5506317 | 5.34E-10 | 1.94E-08 | 2.7039438 | 1.06999215 |
| STXBP2 | -2.3693186 | 1.19727398 | 17.2505278 | 3.28E-05 | 0.00049844 | 2.16839164 | 0.8161516 |
| GMNC | -2.3657081 | -0.9804205 | 11.4445842 | 0.00071703 | 0.00743343 | 0.76478189 | 0.19620668 |
| ZBTB32 | -2.3652774 | -2.175507 | 10.8399149 | 0.00099335 | 0.0097097 | 0.34939815 | 0.06717951 |
| THEMIS2 | -2.3607537 | 4.27471525 | 59.0197871 | 1.56E-14 | 1.03E-12 | 5.08851598 | 2.92468849 |
| PHLDA2 | -2.360156 | 6.47369285 | 20.087331 | 7.40E-06 | 0.00013591 | 7.05686546 | 4.95675737 |
| KRT80 | -2.3535986 | 1.26191145 | 35.1186495 | 3.10E-09 | 1.01E-07 | 2.30314892 | 0.84399213 |
| IL12A | -2.3534834 | 1.23950894 | 44.6022738 | 2.41E-11 | 1.07E-09 | 2.29980429 | 0.83794687 |
| CHCHD10 | -2.3488659 | 5.05024562 | 16.8104516 | 4.13E-05 | 0.00061354 | 5.61433014 | 3.60332442 |
| ADAMTS16 | -2.3488633 | -0.168413 | 15.9822397 | 6.39E-05 | 0.00090708 | 1.20985926 | 0.35541314 |
| COL4A1 | -2.3471112 | 10.2959756 | 17.4068562 | 3.02E-05 | 0.00046276 | 10.8248489 | 8.74811738 |
| TBX18 | -2.346582 | 2.91006697 | 19.5799098 | 9.65E-06 | 0.00017123 | 3.60914618 | 1.83118846 |
| EDN1 | -2.3452702 | 2.79418624 | 30.440987 | 3.44E-08 | 9.64E-07 | 3.60189058 | 1.74758359 |
| CDH6 | -2.3438447 | 2.81967024 | 38.7102151 | 4.92E-10 | 1.81E-08 | 3.65726747 | 1.76997152 |
| RRAGD | -2.341801 | 4.82117763 | 71.1132513 | 3.37E-17 | 2.86E-15 | 5.63378904 | 3.42741866 |
| CTXN1 | -2.340952 | 3.90281404 | 13.1099589 | 0.00029373 | 0.00345112 | 4.40525202 | 2.60536022 |
| SDK1 | -2.331668 | 0.47656437 | 21.9706101 | 2.77E-06 | 5.53E-05 | 1.67577942 | 0.53594962 |
| SLC2A8 | -2.3307325 | 2.64926393 | 21.4589163 | 3.61E-06 | 7.10E-05 | 3.4119447 | 1.65837026 |
| HLA-J | -2.3239418 | 4.85720417 | 22.4211956 | 2.19E-06 | 4.47E-05 | 5.51109999 | 3.45400802 |
| MICA | -2.3175537 | 6.70528243 | 71.6129263 | 2.62E-17 | 2.23E-15 | 7.49773293 | 5.22839087 |
| SLC39A8 | -2.3159382 | 4.31774795 | 35.6100672 | 2.41E-09 | 8.00E-08 | 5.08374938 | 2.9755075 |
| NGF | -2.3139129 | 4.35729887 | 66.5245155 | 3.46E-16 | 2.66E-14 | 5.17324514 | 3.03185998 |
| ANKRD37 | -2.3128807 | 2.78736959 | 39.1473773 | 3.93E-10 | 1.48E-08 | 3.64406473 | 1.75850648 |
| COL4A5 | -2.2994156 | -0.5346225 | 11.2356044 | 0.00080243 | 0.00813118 | 0.99537576 | 0.28111514 |
| LY6E | -2.2987254 | 9.91621458 | 14.1436097 | 0.00016937 | 0.00212687 | 10.3657456 | 8.410267 |
| TMEM132A | -2.2896253 | 5.37199662 | 53.2592468 | 2.92E-13 | 1.62E-11 | 6.14299556 | 3.97690412 |
| CD274 | -2.2871831 | 2.53014678 | 43.7538655 | 3.72E-11 | 1.60E-09 | 3.40159781 | 1.60308955 |
| C11orf96 | -2.2783758 | 5.91198441 | 28.9917421 | 7.27E-08 | 1.93E-06 | 6.59963237 | 4.48250569 |
| PDPN | -2.2779549 | 2.56153417 | 46.0098412 | 1.18E-11 | 5.41E-10 | 3.44060963 | 1.62988999 |
| PARP14 | -2.2778703 | 6.75772381 | 50.0834168 | 1.47E-12 | 7.47E-11 | 7.50846655 | 5.31446815 |
| PCDHB16 | -2.2766525 | 1.13995146 | 23.2339377 | 1.43E-06 | 3.01E-05 | 2.14601652 | 0.8193222 |
| SLC12A7 | -2.2748575 | 4.19604404 | 61.278768 | 4.95E-15 | 3.41E-13 | 5.00957222 | 2.91647435 |
| SP110 | -2.272603 | 5.05958022 | 73.9346738 | 8.07E-18 | 7.44E-16 | 5.86379599 | 3.69775923 |
| APOL2 | -2.2680566 | 7.24930404 | 27.3870118 | 1.67E-07 | 4.14E-06 | 7.90653758 | 5.79908802 |
| KRT86 | -2.2654549 | 0.17693702 | 15.859968 | 6.82E-05 | 0.00095662 | 1.46351606 | 0.46835155 |
| FRZB | -2.2653945 | 2.48169521 | 53.1206148 | 3.14E-13 | 1.73E-11 | 3.38798711 | 1.57199418 |
| RAP1GAP | -2.257025 | -1.0959407 | 12.3332758 | 0.00044496 | 0.00493517 | 0.76093556 | 0.19140293 |
| FSTL3 | -2.2566758 | 7.00726743 | 23.8689379 | 1.03E-06 | 2.20E-05 | 7.63915361 | 5.56480208 |
| FRMPD3 | -2.2545764 | 0.93989662 | 14.4387849 | 0.00014479 | 0.00185362 | 1.92727284 | 0.73478618 |
| KRT34 | -2.2518042 | 4.36602147 | 57.3357333 | 3.67E-14 | 2.34E-12 | 5.16491488 | 3.08371725 |
| NPAS1 | -2.2476691 | 4.80793192 | 13.6419895 | 0.00022118 | 0.00267509 | 5.29733233 | 3.47108382 |
| PLAAT1 | -2.2474085 | -1.8589961 | 10.8862097 | 0.00096883 | 0.00950095 | 0.43692482 | 0.10030963 |
| FAM83H-AS1 | -2.2460605 | -0.2613501 | 27.3113008 | 1.73E-07 | 4.29E-06 | 1.2019285 | 0.35392858 |
| MAOA | -2.2427065 | 1.37184008 | 51.0126438 | 9.18E-13 | 4.76E-11 | 2.41526106 | 0.94205335 |
| IFITM10 | -2.2381743 | 7.45264567 | 14.5370449 | 0.00013743 | 0.00177024 | 7.9228193 | 6.01817601 |
| IFI44 | -2.2344763 | 6.06234662 | 72.2519396 | 1.89E-17 | 1.65E-15 | 6.8461535 | 4.67652621 |
| KCP | -2.2336719 | -1.8688555 | 12.5820826 | 0.00038946 | 0.00439396 | 0.42705236 | 0.10143726 |
| HLA-F | -2.2327233 | 5.75569088 | 47.6935904 | 4.98E-12 | 2.40E-10 | 6.50479876 | 4.38365583 |
| CASP1 | -2.2311395 | 3.63392217 | 47.8779867 | 4.54E-12 | 2.19E-10 | 4.43983506 | 2.47208978 |
| SPON1 | -2.2306104 | 2.83810128 | 18.3449067 | 1.84E-05 | 0.00030092 | 3.55858485 | 1.83056691 |
| PLSCR1 | -2.2208451 | 5.57449353 | 35.7193922 | 2.28E-09 | 7.62E-08 | 6.29034767 | 4.21552938 |
| RNF144B | -2.2174515 | 3.59832175 | 52.9373698 | 3.44E-13 | 1.89E-11 | 4.41198827 | 2.45183477 |
| MIR155HG | -2.2111635 | 1.17021815 | 38.8845116 | 4.50E-10 | 1.67E-08 | 2.23814786 | 0.85673844 |
| HLA-DRA | -2.2101577 | 3.41644062 | 62.3874848 | 2.82E-15 | 1.99E-13 | 4.25278261 | 2.30798248 |
| GRK3 | -2.2029572 | 1.93110893 | 48.516651 | 3.27E-12 | 1.60E-10 | 2.88045326 | 1.26152684 |
| CRISPLD2 | -2.2000658 | 5.11351003 | 68.6111919 | 1.20E-16 | 9.76E-15 | 5.90203111 | 3.80408343 |
| MPPED2 | -2.1970875 | 1.46483856 | 23.3604627 | 1.34E-06 | 2.83E-05 | 2.42978631 | 0.99264202 |
| ADAMTS5 | -2.1907807 | 6.78390074 | 17.6208137 | 2.70E-05 | 0.00041799 | 7.33860705 | 5.39304398 |
| MAPK8IP2 | -2.1871911 | 1.29828783 | 30.5126233 | 3.32E-08 | 9.35E-07 | 2.3380339 | 0.91425842 |
| HIF1A-AS1 | -2.1867888 | -0.6243288 | 30.0151168 | 4.29E-08 | 1.19E-06 | 0.99981964 | 0.28513125 |
| FBXL16 | -2.1780422 | -0.7018307 | 24.7465414 | 6.54E-07 | 1.46E-05 | 0.95579433 | 0.27039017 |
| MYPN | -2.1729838 | 2.17637247 | 42.5978406 | 6.72E-11 | 2.78E-09 | 3.07703389 | 1.42885583 |
| ENKUR | -2.1709112 | -0.638482 | 14.1109983 | 0.00017233 | 0.00215632 | 0.98711073 | 0.2750329 |
| MCOLN3 | -2.167949 | 2.07232401 | 36.0715238 | 1.90E-09 | 6.42E-08 | 2.98519062 | 1.35477409 |
| SDSL | -2.1632655 | 4.43060176 | 11.7183773 | 0.00061886 | 0.00653929 | 4.87082307 | 3.20448361 |
| PLOD2 | -2.1628068 | 8.48969275 | 11.9189397 | 0.00055566 | 0.00594934 | 8.8871686 | 7.10168049 |
| JAM2 | -2.1595634 | 3.77877295 | 50.8898513 | 9.77E-13 | 5.04E-11 | 4.57757891 | 2.63914816 |
| HOXA11 | -2.1577726 | 2.86547756 | 55.6038271 | 8.87E-14 | 5.29E-12 | 3.72439141 | 1.91356812 |
| TMEM140 | -2.1559077 | 5.05819204 | 62.8823351 | 2.19E-15 | 1.56E-13 | 5.84130874 | 3.78116075 |
| GRB14 | -2.1547786 | 0.44127033 | 43.5512985 | 4.13E-11 | 1.75E-09 | 1.67612616 | 0.58288628 |
| MT1X | -2.1532013 | 4.95939632 | 11.1662223 | 0.000833 | 0.00837629 | 5.36661544 | 3.68430366 |
| CNTNAP2 | -2.1461141 | 2.81723296 | 18.9125219 | 1.37E-05 | 0.00023172 | 3.52814259 | 1.88100562 |
| GPR150 | -2.1449345 | -1.917263 | 12.3266662 | 0.00044653 | 0.00494745 | 0.4211357 | 0.10028707 |
| FBXL22 | -2.1432865 | 1.04895116 | 32.6828163 | 1.08E-08 | 3.27E-07 | 2.12487769 | 0.82533839 |
| CPZ | -2.1409716 | 4.54102812 | 42.7908501 | 6.09E-11 | 2.52E-09 | 5.29979111 | 3.31736263 |
| KRTAP2-3 | -2.1408811 | 4.25202968 | 54.4897144 | 1.56E-13 | 8.96E-12 | 5.03698201 | 3.06463986 |
| IGFBP4 | -2.134964 | 12.6082747 | 19.4027189 | 1.06E-05 | 0.00018599 | 13.1904404 | 11.2368768 |
| PLPP4 | -2.1340059 | 3.36210422 | 23.2106858 | 1.45E-06 | 3.05E-05 | 4.09064996 | 2.3032512 |
| IL26 | -2.1283956 | -0.473634 | 26.3974833 | 2.78E-07 | 6.56E-06 | 1.06215315 | 0.32761872 |
| SHC3 | -2.1242314 | 1.5542094 | 41.15246 | 1.41E-10 | 5.62E-09 | 2.53207443 | 1.08543544 |
| C3orf52 | -2.1228664 | 1.73584323 | 31.2166678 | 2.31E-08 | 6.67E-07 | 2.69318205 | 1.17250682 |
| HIST1H2BC | -2.1164745 | 1.0533404 | 22.4068769 | 2.21E-06 | 4.49E-05 | 2.06673091 | 0.83865196 |
| ZMYND15 | -2.1124142 | 0.50289724 | 15.2985232 | 9.18E-05 | 0.00125061 | 1.67966369 | 0.60534035 |
| USP32P1 | -2.1115697 | 1.26813033 | 15.5467462 | 8.05E-05 | 0.00111329 | 2.19424335 | 0.93249498 |
| RASSF2 | -2.1076338 | -0.3133821 | 20.4156745 | 6.23E-06 | 0.00011641 | 1.16499681 | 0.36889038 |
| NUAK2 | -2.1072717 | 2.80873082 | 54.9411801 | 1.24E-13 | 7.26E-12 | 3.66706408 | 1.89925327 |
| PDLIM4 | -2.1064459 | 7.16517155 | 15.3752019 | 8.81E-05 | 0.00120477 | 7.68090398 | 5.83559419 |
| SYT8 | -2.1044299 | 0.34494944 | 12.1557373 | 0.00048937 | 0.00534591 | 1.5270563 | 0.56380101 |
| PIEZO2 | -2.0924722 | 5.00923276 | 14.0702314 | 0.00017611 | 0.00220095 | 5.52855569 | 3.7687762 |
| CD40 | -2.089063 | 1.61214451 | 31.3275482 | 2.18E-08 | 6.31E-07 | 2.58787747 | 1.11677561 |
| PFKFB4 | -2.0855861 | 4.57473151 | 62.0544486 | 3.34E-15 | 2.33E-13 | 5.35412271 | 3.39360929 |
| DYSF | -2.0847402 | 5.0144646 | 57.6666854 | 3.11E-14 | 1.99E-12 | 5.77858993 | 3.79903919 |
| CD34 | -2.082337 | 0.44953566 | 33.5829489 | 6.83E-09 | 2.11E-07 | 1.66653167 | 0.60307418 |
| TMEM229B | -2.0794353 | 3.33426294 | 42.9254176 | 5.69E-11 | 2.36E-09 | 4.13930329 | 2.32019519 |
| PARP9 | -2.0793692 | 6.20198467 | 31.8574632 | 1.66E-08 | 4.91E-07 | 6.88347245 | 4.92985926 |
| SNHG17 | -2.0787828 | 3.93788539 | 18.0098524 | 2.20E-05 | 0.00034929 | 4.56793089 | 2.82698636 |
| SHFL | -2.0762025 | 5.99619998 | 41.3389605 | 1.28E-10 | 5.14E-09 | 6.71347244 | 4.73607885 |
| SEMA4D | -2.0717684 | -0.1793279 | 24.1393567 | 8.96E-07 | 1.93E-05 | 1.23867065 | 0.40581742 |
| ZC3H12A | -2.0679518 | 5.15267456 | 53.9549671 | 2.05E-13 | 1.16E-11 | 5.90672072 | 3.94028113 |
| BEND5 | -2.0613901 | 0.49113016 | 17.873057 | 2.36E-05 | 0.00037055 | 1.68654243 | 0.61338883 |
| HLA-DOB | -2.061017 | 0.11687239 | 30.302685 | 3.70E-08 | 1.03E-06 | 1.4362788 | 0.49817897 |
| PSMB9 | -2.0593648 | 4.88453476 | 17.7566502 | 2.51E-05 | 0.0003913 | 5.47286683 | 3.69119906 |
| RSPO1 | -2.0584846 | -1.1250558 | 12.0918509 | 0.00050643 | 0.0054979 | 0.7275105 | 0.20118866 |
| CHODL | -2.0571655 | -0.6666495 | 11.3398352 | 0.00075862 | 0.00778114 | 0.9588911 | 0.29220308 |
| FST | -2.0516766 | 8.80819733 | 40.9168084 | 1.59E-10 | 6.30E-09 | 9.51605706 | 7.52167704 |
| TMEM51 | -2.0429305 | 1.38839844 | 26.5655029 | 2.55E-07 | 6.06E-06 | 2.39462231 | 1.01036653 |
| IL18BP | -2.0401373 | 2.54576438 | 46.7084738 | 8.24E-12 | 3.86E-10 | 3.40856578 | 1.74700218 |
| TNFRSF10A | -2.0368326 | 2.43082218 | 24.9918753 | 5.76E-07 | 1.29E-05 | 3.24567983 | 1.65862331 |
| LMNTD2 | -2.0314075 | 1.30262074 | 24.9867254 | 5.77E-07 | 1.29E-05 | 2.29037737 | 0.992686 |
| HEPH | -2.0291642 | 4.80648068 | 46.9142883 | 7.42E-12 | 3.51E-10 | 5.54903749 | 3.6487588 |
| EYA4 | -2.0272539 | 3.19797648 | 15.0283926 | 0.00010591 | 0.0014136 | 3.85918368 | 2.21650557 |
| SSC5D | -2.0230638 | 5.70816295 | 59.4467042 | 1.26E-14 | 8.40E-13 | 6.4575141 | 4.49974226 |
| FHIT | -2.0180171 | 0.81806864 | 13.8311638 | 0.00019999 | 0.00245988 | 1.87419748 | 0.76277927 |
| GNA14 | -2.0163952 | 1.61012057 | 28.020996 | 1.20E-07 | 3.08E-06 | 2.57587515 | 1.14008109 |
| UBE2L6 | -2.0161637 | 6.779431 | 28.2356518 | 1.07E-07 | 2.78E-06 | 7.43582409 | 5.54182241 |
| FILIP1L | -2.0154842 | 3.09122247 | 11.3370352 | 0.00075977 | 0.00778528 | 3.63628776 | 2.17186675 |
| NMB | -2.0075508 | 5.97069302 | 13.2460805 | 0.00027315 | 0.00324198 | 6.44869955 | 4.75694614 |
| LINC01139 | -2.003536 | 0.92467578 | 16.0759207 | 6.09E-05 | 0.00086857 | 1.92247985 | 0.81866261 |
| RTKN2 | -2.0026651 | 3.15370801 | 39.5917956 | 3.13E-10 | 1.20E-08 | 3.94730181 | 2.22868649 |
| TRIM21 | -2.0025265 | 5.11343024 | 56.4829168 | 5.67E-14 | 3.51E-12 | 5.86618699 | 3.95075677 |
| CD74 | -2.0021446 | 6.25220463 | 30.4157914 | 3.49E-08 | 9.75E-07 | 6.92387288 | 5.03778451 |
| GBP3 | -2.0009281 | 5.77139925 | 24.5049519 | 7.41E-07 | 1.63E-05 | 6.40792037 | 4.57764265 |
| RTN4RL2 | 2.00047881 | 0.12511873 | 27.0495596 | 1.98E-07 | 4.86E-06 | 0.4931096 | 1.42061963 |
| KIF18A | 2.00415604 | 1.831312 | 15.7000945 | 7.42E-05 | 0.00103406 | 1.21213104 | 2.74359223 |
| SLC7A8 | 2.00513709 | 4.80118743 | 50.903372 | 9.70E-13 | 5.02E-11 | 3.63305365 | 5.57367983 |
| AKR1C3 | 2.00574983 | 6.05173933 | 41.9786198 | 9.23E-11 | 3.79E-09 | 4.80334312 | 6.80451668 |
| LINC01004 | 2.00619535 | 2.19539931 | 42.5556333 | 6.87E-11 | 2.83E-09 | 1.49062683 | 3.0960455 |
| CKS2 | 2.00846049 | 4.33571568 | 57.2146285 | 3.91E-14 | 2.47E-12 | 3.21676553 | 5.11965081 |
| UHRF1 | 2.0099636 | 4.23585389 | 40.1239769 | 2.38E-10 | 9.27E-09 | 3.09202343 | 5.02416216 |
| ADRB2 | 2.01302513 | 0.49970542 | 23.4518532 | 1.28E-06 | 2.71E-05 | 0.58510489 | 1.69774487 |
| NCAPG2 | 2.01411471 | 4.46295366 | 29.998395 | 4.32E-08 | 1.20E-06 | 3.26309027 | 5.24175638 |
| CDCA2 | 2.01561676 | 2.98461501 | 56.2227932 | 6.47E-14 | 3.96E-12 | 2.07425326 | 3.82446269 |
| SNORD17 | 2.01625874 | -1.2615956 | 13.9229567 | 0.00019046 | 0.00235365 | 0.16099888 | 0.65039857 |
| MSH5-SAPCD1 | 2.01634447 | -1.1772129 | 11.2332951 | 0.00080343 | 0.00813458 | 0.2065341 | 0.67379627 |
| PLIN4 | 2.01663046 | -0.464788 | 19.2037934 | 1.17E-05 | 0.00020328 | 0.32346365 | 1.05165879 |
| PLPP2 | 2.01702417 | -1.2979866 | 11.015786 | 0.00090339 | 0.00897238 | 0.18910591 | 0.6229305 |
| ARHGAP11A | 2.02424039 | 4.10203402 | 15.4256146 | 8.58E-05 | 0.00118307 | 2.84153859 | 4.86924728 |
| SHROOM3 | 2.03180871 | 3.61760294 | 35.5508416 | 2.48E-09 | 8.19E-08 | 2.53794187 | 4.42219258 |
| PCOLCE2 | 2.03911074 | 4.87601769 | 44.9044627 | 2.07E-11 | 9.22E-10 | 3.65944794 | 5.65672802 |
| SLC8A1 | 2.04001311 | 3.67423845 | 18.8789076 | 1.39E-05 | 0.00023565 | 2.50179005 | 4.46780581 |
| FOXQ1 | 2.04257821 | -0.1361257 | 17.0406803 | 3.66E-05 | 0.00054989 | 0.38425811 | 1.2525117 |
| JCHAIN | 2.04320743 | -0.4578974 | 20.9132363 | 4.81E-06 | 9.24E-05 | 0.31176833 | 1.06157812 |
| FRMD4B | 2.04885997 | 0.8765227 | 15.2648627 | 9.34E-05 | 0.00126898 | 0.71885762 | 1.96625557 |
| ARTN | 2.05789502 | -1.3051272 | 15.3277732 | 9.04E-05 | 0.0012346 | 0.17875216 | 0.62721082 |
| LINC01001 | 2.06178438 | -0.2756786 | 14.6447544 | 0.0001298 | 0.00168504 | 0.37350416 | 1.16967111 |
| LYG1 | 2.06309159 | -0.3985775 | 25.0660455 | 5.54E-07 | 1.25E-05 | 0.34764937 | 1.09342138 |
| OIP5 | 2.06465922 | 1.42246671 | 33.9439255 | 5.67E-09 | 1.77E-07 | 1.01515587 | 2.42646071 |
| CENPM | 2.06534039 | 2.84099879 | 11.1860131 | 0.00082416 | 0.00830332 | 1.75686018 | 3.67657107 |
| SBSN | 2.07371183 | 3.27009288 | 39.800093 | 2.81E-10 | 1.08E-08 | 2.2401399 | 4.10195899 |
| LSAMP | 2.07592243 | 3.59931858 | 40.8861096 | 1.61E-10 | 6.38E-09 | 2.49687472 | 4.41772205 |
| HES1 | 2.07697565 | 4.08405004 | 47.4643876 | 5.60E-12 | 2.69E-10 | 2.92910216 | 4.88604022 |
| RUBCNL | 2.077194 | 0.82765654 | 32.3588233 | 1.28E-08 | 3.84E-07 | 0.72150293 | 1.94180542 |
| TYMSOS | 2.07848603 | -0.105968 | 21.6391954 | 3.29E-06 | 6.51E-05 | 0.4098728 | 1.26921268 |
| ARHGAP30 | 2.07872335 | -1.4616874 | 15.0935948 | 0.00010231 | 0.00137083 | 0.13031927 | 0.57073637 |
| PICART1 | 2.08681452 | -1.4154357 | 13.5713109 | 0.00022967 | 0.00276816 | 0.16193355 | 0.58091505 |
| XKR5 | 2.0881071 | -1.1343986 | 14.9141208 | 0.00011252 | 0.00149426 | 0.20768524 | 0.70449069 |
| SNCA | 2.09003486 | 0.06058948 | 28.4605326 | 9.56E-08 | 2.50E-06 | 0.45624787 | 1.38616209 |
| KSR2 | 2.09159521 | -1.4244362 | 14.7623289 | 0.00012195 | 0.00160026 | 0.15479825 | 0.57918696 |
| TMEM71 | 2.09170174 | 0.18544254 | 33.6035535 | 6.76E-09 | 2.09E-07 | 0.48353394 | 1.47966027 |
| AEBP1 | 2.09678883 | 7.72017363 | 50.2916075 | 1.33E-12 | 6.77E-11 | 6.37285341 | 8.48765333 |
| EPYC | 2.10040639 | -0.7909145 | 15.3180527 | 9.08E-05 | 0.00124016 | 0.23568788 | 0.87571892 |
| LRRC71 | 2.10600355 | -0.5806143 | 14.4272629 | 0.00014568 | 0.0018605 | 0.29339645 | 0.97709352 |
| ZFHX4-AS1 | 2.10851092 | 0.04509611 | 24.3281915 | 8.12E-07 | 1.77E-05 | 0.42927748 | 1.38080738 |
| MXD3 | 2.11732053 | 3.16772934 | 18.4932503 | 1.71E-05 | 0.00028256 | 2.04125946 | 4.00648422 |
| RACGAP1 | 2.11951285 | 5.26221679 | 45.9736769 | 1.20E-11 | 5.49E-10 | 3.95771041 | 6.04836651 |
| ASF1B | 2.12410654 | 3.61601541 | 59.2134078 | 1.41E-14 | 9.40E-13 | 2.50829929 | 4.44498675 |
| ZFP36L2 | 2.12806671 | 7.24806076 | 52.0263691 | 5.48E-13 | 2.93E-11 | 5.8782234 | 8.02531165 |
| GAL3ST4 | 2.12852731 | 2.02515937 | 37.0035583 | 1.18E-09 | 4.11E-08 | 1.3300806 | 2.95367777 |
| MYLIP | 2.13965123 | 2.38768771 | 49.5970643 | 1.89E-12 | 9.39E-11 | 1.55389538 | 3.2939252 |
| USP32P2 | 2.1411867 | 1.88186683 | 14.6774946 | 0.00012756 | 0.00166042 | 1.14653349 | 2.8388161 |
| LRATD1 | 2.14271766 | 4.43429736 | 35.5890459 | 2.44E-09 | 8.07E-08 | 3.14757323 | 5.24203494 |
| TMEM155 | 2.14851531 | 1.98097837 | 39.0277336 | 4.18E-10 | 1.56E-08 | 1.28190269 | 2.91830357 |
| ERCC6L | 2.14955691 | 1.79822252 | 49.8026151 | 1.70E-12 | 8.54E-11 | 1.18761277 | 2.76501481 |
| CHGB | 2.15108002 | -1.7170495 | 11.1711175 | 0.00083081 | 0.00836222 | 0.10713998 | 0.46817813 |
| MUC20 | 2.15298589 | 1.3646416 | 28.6750233 | 8.56E-08 | 2.25E-06 | 0.94714408 | 2.38840401 |
| SLC14A1 | 2.15534572 | 7.35765835 | 61.2030468 | 5.15E-15 | 3.53E-13 | 5.9771307 | 8.13946103 |
| PCDH18 | 2.16245727 | 6.4643562 | 15.3972176 | 8.71E-05 | 0.00119622 | 4.85560208 | 7.23802415 |
| NOTCH2NLR | 2.16526857 | 1.03216437 | 38.7462666 | 4.83E-10 | 1.79E-08 | 0.79921263 | 2.11583611 |
| WNT9A | 2.16532863 | 1.35727311 | 35.067095 | 3.19E-09 | 1.03E-07 | 0.91049362 | 2.39474693 |
| PEG10 | 2.16696184 | 3.70086099 | 40.4322857 | 2.04E-10 | 7.97E-09 | 2.51125254 | 4.53178219 |
| SNORD14D | 2.17010721 | -0.3872027 | 12.290828 | 0.00045519 | 0.0050354 | 0.30434346 | 1.08976147 |
| LRP5L | 2.17389741 | 1.67974026 | 51.6583457 | 6.60E-13 | 3.49E-11 | 1.12034104 | 2.66282273 |
| ADAMTSL5 | 2.17447335 | 1.2429988 | 44.8944735 | 2.08E-11 | 9.25E-10 | 0.88968876 | 2.29242636 |
| DCHS1 | 2.17774246 | 1.11944991 | 29.2742767 | 6.28E-08 | 1.68E-06 | 0.78948441 | 2.19627332 |
| FAM20A | 2.18194525 | 5.53943151 | 31.3434714 | 2.16E-08 | 6.28E-07 | 4.12450879 | 6.32558941 |
| NSD2 | 2.18543935 | 5.95658972 | 54.5848685 | 1.49E-13 | 8.56E-12 | 4.57516629 | 6.74901249 |
| PAMR1 | 2.18929235 | 5.81137225 | 66.6396554 | 3.26E-16 | 2.52E-14 | 4.45185445 | 6.60501836 |
| ZNF853 | 2.20174262 | -1.3313347 | 12.1357369 | 0.00049465 | 0.00539232 | 0.16193355 | 0.61632034 |
| EVI2A | 2.20605113 | 3.48034118 | 44.0325815 | 3.23E-11 | 1.40E-09 | 2.31336681 | 4.32347167 |
| PHEX | 2.21201993 | 2.19793509 | 36.542035 | 1.49E-09 | 5.13E-08 | 1.37542086 | 3.11837525 |
| PRRT2 | 2.21783482 | 0.98727134 | 25.5815264 | 4.24E-07 | 9.74E-06 | 0.72225022 | 2.08939088 |
| ESPNL | 2.21888191 | 0.40155638 | 12.9230875 | 0.00032455 | 0.00376899 | 0.52030355 | 1.6212731 |
| SHCBP1 | 2.22049598 | 4.34101308 | 35.1015789 | 3.13E-09 | 1.02E-07 | 3.00071088 | 5.16247584 |
| PKDCC | 2.22783403 | 0.45714434 | 37.4310069 | 9.47E-10 | 3.33E-08 | 0.55825261 | 1.68100519 |
| PTPRC | 2.22977939 | -1.363124 | 12.6278102 | 0.00038005 | 0.00431326 | 0.13440673 | 0.61400821 |
| THRB | 2.22995193 | 0.8446349 | 28.8397501 | 7.86E-08 | 2.08E-06 | 0.67226852 | 1.97601306 |
| CARD10 | 2.23056922 | 1.74386244 | 41.8221974 | 1.00E-10 | 4.06E-09 | 1.106072 | 2.72981028 |
| BCL2L11 | 2.23070832 | 1.72727014 | 14.2091848 | 0.00016357 | 0.00206514 | 1.0210368 | 2.71489864 |
| MASP1 | 2.24508289 | 1.73647304 | 57.1596842 | 4.02E-14 | 2.53E-12 | 1.11583039 | 2.72437109 |
| TCEA3 | 2.24623018 | 4.39039799 | 25.5196001 | 4.38E-07 | 1.00E-05 | 2.98689596 | 5.2076388 |
| PTTG1 | 2.24651002 | 4.78710365 | 24.8731206 | 6.12E-07 | 1.37E-05 | 3.32659812 | 5.60276896 |
| OLFML2B | 2.24863142 | 6.50886989 | 62.5899954 | 2.55E-15 | 1.80E-13 | 5.06457174 | 7.31063361 |
| HTR2B | 2.25766823 | -1.2228842 | 15.6028676 | 7.81E-05 | 0.00108502 | 0.14418026 | 0.68127962 |
| FES | 2.2587529 | 3.25961774 | 24.3118079 | 8.19E-07 | 1.78E-05 | 2.04419808 | 4.12355227 |
| PBX1 | 2.26302635 | 3.0836734 | 35.3057484 | 2.82E-09 | 9.17E-08 | 1.93841344 | 3.95957833 |
| CRYBG1 | 2.26831693 | 5.27671253 | 33.9516434 | 5.65E-09 | 1.76E-07 | 3.81450824 | 6.07692526 |
| KCNS3 | 2.2730994 | 3.06551507 | 55.1362591 | 1.12E-13 | 6.61E-12 | 1.95309676 | 3.94691665 |
| MBOAT1 | 2.27758733 | 0.52232957 | 27.0176768 | 2.02E-07 | 4.92E-06 | 0.56666255 | 1.7200824 |
| WDR76 | 2.28041435 | 2.65246568 | 21.2284767 | 4.08E-06 | 7.94E-05 | 1.57312307 | 3.55280647 |
| RAB3D | 2.28530855 | 0.98685299 | 33.7723941 | 6.20E-09 | 1.92E-07 | 0.73711348 | 2.09119624 |
| CCNA2 | 2.28789628 | 4.28583559 | 37.7509448 | 8.04E-10 | 2.86E-08 | 2.90522313 | 5.1209113 |
| RASD1 | 2.29524757 | 4.76768619 | 65.9739782 | 4.57E-16 | 3.41E-14 | 3.38676785 | 5.59488194 |
| CYP19A1 | 2.29586093 | -1.6327201 | 11.9812598 | 0.00053738 | 0.00578609 | 0.10133789 | 0.5070744 |
| SPIN2A | 2.3028398 | -1.8062944 | 12.7766271 | 0.00035098 | 0.00403787 | 0.08616479 | 0.44463304 |
| COL10A1 | 2.30406451 | 4.43287693 | 11.4069386 | 0.0007317 | 0.00755673 | 2.75809633 | 5.26892311 |
| MIR616 | 2.30468856 | -0.8515482 | 17.1148143 | 3.52E-05 | 0.00053073 | 0.19296812 | 0.8636081 |
| CHDH | 2.30610851 | -0.735331 | 19.9628783 | 7.90E-06 | 0.00014429 | 0.22257323 | 0.92217516 |
| KIF20B | 2.30678022 | 3.26778508 | 35.6674246 | 2.34E-09 | 7.80E-08 | 2.05188887 | 4.13608566 |
| KCNB1 | 2.3173355 | 0.05762223 | 32.2550626 | 1.35E-08 | 4.05E-07 | 0.39400532 | 1.41320946 |
| SGO1 | 2.33252499 | 0.92621048 | 49.9824719 | 1.55E-12 | 7.83E-11 | 0.68238137 | 2.0623679 |
| NHSL2 | 2.33630884 | 2.50603182 | 52.7778976 | 3.73E-13 | 2.05E-11 | 1.52181213 | 3.42842859 |
| NLGN1 | 2.34152329 | 0.3797683 | 11.1521136 | 0.00083936 | 0.00842409 | 0.44840265 | 1.63458138 |
| ZNF90 | 2.34246617 | -1.0304661 | 16.7259789 | 4.32E-05 | 0.0006408 | 0.16483472 | 0.77593944 |
| ROBO2 | 2.34346802 | -1.2795997 | 12.9143483 | 0.00032607 | 0.00378245 | 0.13495264 | 0.65678874 |
| KIFC1 | 2.34653859 | 4.41341495 | 53.6173808 | 2.44E-13 | 1.36E-11 | 3.01771806 | 5.25173845 |
| TPX2 | 2.35354678 | 5.61039668 | 68.1520836 | 1.51E-16 | 1.22E-14 | 4.11820122 | 6.43717974 |
| NAPA-AS1 | 2.35622302 | -1.2232307 | 13.1996457 | 0.00028 | 0.00331207 | 0.16193355 | 0.67041958 |
| ARHGAP11B | 2.3588213 | -0.2727368 | 11.7824126 | 0.00059793 | 0.00634047 | 0.29049628 | 1.17775206 |
| CENPE | 2.36374017 | 3.74529249 | 46.6908793 | 8.31E-12 | 3.89E-10 | 2.42720085 | 4.59356 |
| AGAP11 | 2.36416859 | 1.16364448 | 30.6568195 | 3.08E-08 | 8.73E-07 | 0.73939078 | 2.25555838 |
| RGPD1 | 2.36779996 | 0.25400849 | 27.4416005 | 1.62E-07 | 4.04E-06 | 0.45178678 | 1.54393022 |
| NCAPG | 2.36793472 | 3.81915079 | 54.8593135 | 1.29E-13 | 7.55E-12 | 2.49154855 | 4.67322074 |
| ESCO2 | 2.36870602 | 1.29730511 | 19.2814554 | 1.13E-05 | 0.00019654 | 0.79569134 | 2.32443534 |
| CCNB1 | 2.37494383 | 5.56603716 | 76.1404434 | 2.64E-18 | 2.54E-16 | 4.06949209 | 6.39657129 |
| HPSE | 2.37562916 | 1.24486565 | 37.7648325 | 7.98E-10 | 2.84E-08 | 0.78498237 | 2.32178258 |
| MIR503HG | 2.37922347 | 1.01826653 | 19.2028266 | 1.18E-05 | 0.00020328 | 0.69320111 | 2.12200866 |
| LURAP1 | 2.38068533 | 0.37177814 | 38.6587659 | 5.05E-10 | 1.85E-08 | 0.49364233 | 1.63754983 |
| TFAP2E | 2.38460342 | -0.6798634 | 26.7868679 | 2.27E-07 | 5.47E-06 | 0.21418193 | 0.9684379 |
| ADAMTS7P1 | 2.38610509 | 2.73011225 | 53.2876384 | 2.88E-13 | 1.60E-11 | 1.63409493 | 3.6487945 |
| SKA1 | 2.38969828 | 2.85178393 | 67.6701043 | 1.93E-16 | 1.54E-14 | 1.75150444 | 3.75584415 |
| DAPK2 | 2.39264207 | 2.39427957 | 33.1420388 | 8.57E-09 | 2.61E-07 | 1.39992312 | 3.33391855 |
| IDI2-AS1 | 2.39814073 | 0.71309669 | 15.4112638 | 8.65E-05 | 0.00118975 | 0.53880092 | 1.8976055 |
| NCAM1 | 2.40204727 | 2.38402019 | 56.9846858 | 4.39E-14 | 2.74E-12 | 1.41939655 | 3.32569905 |
| KIF15 | 2.40291168 | 1.93172347 | 44.4366798 | 2.63E-11 | 1.15E-09 | 1.12454367 | 2.91273365 |
| KIF11 | 2.41439849 | 4.08926663 | 45.4991572 | 1.53E-11 | 6.92E-10 | 2.67008357 | 4.93540868 |
| FANCD2 | 2.42129212 | 2.77000827 | 18.160743 | 2.03E-05 | 0.00032726 | 1.53778384 | 3.68999407 |
| CDKN2C | 2.42547887 | 4.30373386 | 59.9763943 | 9.60E-15 | 6.46E-13 | 2.86810274 | 5.15215623 |
| HAAO | 2.43064935 | 0.1528557 | 12.2367749 | 0.00046857 | 0.00515891 | 0.39908168 | 1.45837419 |
| NEIL3 | 2.4308427 | 1.11844251 | 37.5919259 | 8.72E-10 | 3.08E-08 | 0.71298607 | 2.21932934 |
| AURKB | 2.4350258 | 3.4844522 | 27.536689 | 1.54E-07 | 3.88E-06 | 2.10561211 | 4.35831169 |
| LINC01776 | 2.43960217 | -1.9252296 | 12.7001557 | 0.00036563 | 0.00417664 | 0.07967406 | 0.40560068 |
| ARC | 2.44035054 | -0.4497605 | 31.5094711 | 1.98E-08 | 5.80E-07 | 0.24954888 | 1.10613951 |
| CSDC2 | 2.44684807 | 1.73855729 | 23.6014329 | 1.18E-06 | 2.51E-05 | 0.98399836 | 2.74719863 |
| MYBPH | 2.44867655 | -1.4983494 | 16.543267 | 4.76E-05 | 0.00069722 | 0.12055144 | 0.56512323 |
| DMC1 | 2.4645211 | -0.7236528 | 27.4893575 | 1.58E-07 | 3.96E-06 | 0.19072175 | 0.9520258 |
| AURKA | 2.47353769 | 4.18475412 | 78.1412709 | 9.59E-19 | 9.64E-17 | 2.74124521 | 5.05324865 |
| PURPL | 2.47814488 | -0.2239516 | 16.0417876 | 6.20E-05 | 0.00088197 | 0.27781264 | 1.24195522 |
| GAS2L3 | 2.49042784 | 2.32965235 | 20.2479083 | 6.80E-06 | 0.00012607 | 1.24232453 | 3.28287979 |
| MIR221 | 2.4955487 | 1.42734053 | 38.8603472 | 4.55E-10 | 1.69E-08 | 0.81725274 | 2.4884995 |
| LIN7A | 2.49655052 | 2.4625645 | 64.95926 | 7.65E-16 | 5.61E-14 | 1.41339643 | 3.41140527 |
| ZNF385D | 2.4970722 | 3.85697554 | 70.2588963 | 5.20E-17 | 4.33E-15 | 2.45335005 | 4.73033938 |
| STARD8 | 2.49791714 | 2.8472014 | 21.1952291 | 4.15E-06 | 8.07E-05 | 1.5614084 | 3.7690039 |
| ESM1 | 2.50176855 | 4.36810687 | 54.0467684 | 1.96E-13 | 1.11E-11 | 2.84183385 | 5.23606576 |
| SFN | 2.50203271 | -1.1617527 | 19.218015 | 1.17E-05 | 0.00020201 | 0.14265181 | 0.71736494 |
| E2F2 | 2.52467675 | 0.94996987 | 52.3437528 | 4.66E-13 | 2.51E-11 | 0.63016445 | 2.10421404 |
| SPC25 | 2.53528355 | 2.06255229 | 53.6974689 | 2.34E-13 | 1.31E-11 | 1.16467789 | 3.04287186 |
| WNT2 | 2.54098383 | 0.26751155 | 35.535948 | 2.50E-09 | 8.24E-08 | 0.41097387 | 1.57889655 |
| PTTG3P | 2.5519591 | 0.76038134 | 39.0530876 | 4.12E-10 | 1.54E-08 | 0.57207912 | 1.93419283 |
| DDX12P | 2.55217561 | 1.09636333 | 44.8552595 | 2.12E-11 | 9.42E-10 | 0.68001839 | 2.20854248 |
| PLK4 | 2.55513939 | 2.27178998 | 36.1264379 | 1.85E-09 | 6.25E-08 | 1.22533378 | 3.23569475 |
| ABCG1 | 2.55643328 | -1.8713087 | 14.7508716 | 0.00012269 | 0.001607 | 0.06582606 | 0.43282684 |
| MND1 | 2.56397278 | 0.94680378 | 31.4535827 | 2.04E-08 | 5.96E-07 | 0.58275363 | 2.10710831 |
| FGF5 | 2.56814378 | 5.02988458 | 35.0086669 | 3.28E-09 | 1.06E-07 | 3.3174059 | 5.88951875 |
| PRR11 | 2.5690295 | 4.82401803 | 80.2896056 | 3.23E-19 | 3.41E-17 | 3.23246087 | 5.69191439 |
| TDRD9 | 2.56999749 | 1.83929596 | 36.7008134 | 1.38E-09 | 4.75E-08 | 0.97390947 | 2.8599712 |
| TACR1 | 2.5927478 | -0.8148077 | 29.8486075 | 4.67E-08 | 1.28E-06 | 0.18865983 | 0.90249209 |
| CCNB2 | 2.60295758 | 4.35553826 | 75.4590081 | 3.73E-18 | 3.58E-16 | 2.7913168 | 5.23512186 |
| SPC24 | 2.60712734 | 3.49221965 | 16.5622031 | 4.71E-05 | 0.00069125 | 1.87411972 | 4.3884147 |
| STARD9 | 2.61670776 | 2.24193776 | 36.3213539 | 1.67E-09 | 5.68E-08 | 1.17563092 | 3.22585785 |
| LINC00242 | 2.62499463 | -1.60906 | 13.7705727 | 0.00020655 | 0.00252571 | 0.0839097 | 0.5276686 |
| HCG27 | 2.63366777 | -0.4657761 | 27.476662 | 1.59E-07 | 3.98E-06 | 0.23676764 | 1.0947567 |
| AGMO | 2.6384812 | -0.6720013 | 32.0986628 | 1.47E-08 | 4.37E-07 | 0.19895273 | 0.98543023 |
| FOXM1 | 2.65683834 | 5.53637233 | 82.8800094 | 8.72E-20 | 9.53E-18 | 3.81373618 | 6.40378955 |
| SEMA4A | 2.6612988 | -0.9976508 | 21.9088347 | 2.86E-06 | 5.68E-05 | 0.16313975 | 0.80943202 |
| SPAG5 | 2.66500796 | 4.46696633 | 87.5551238 | 8.20E-21 | 9.93E-19 | 2.8463393 | 5.35449784 |
| FBLN1 | 2.66738124 | 9.09582318 | 67.9831531 | 1.65E-16 | 1.32E-14 | 7.24880412 | 9.95522454 |
| F2RL2 | 2.68212385 | 4.32229131 | 41.6198625 | 1.11E-10 | 4.49E-09 | 2.63455883 | 5.20540339 |
| MTSS1 | 2.68844888 | 3.06454565 | 84.9739195 | 3.02E-20 | 3.39E-18 | 1.71376516 | 4.00191211 |
| DACT3 | 2.69830821 | 2.55524064 | 58.0594287 | 2.54E-14 | 1.66E-12 | 1.37216537 | 3.51012559 |
| TRPC3 | 2.69848124 | -0.7607558 | 27.050796 | 1.98E-07 | 4.86E-06 | 0.17078521 | 0.93883453 |
| EBF2 | 2.70175844 | 2.60732444 | 17.6350073 | 2.68E-05 | 0.0004156 | 1.27198323 | 3.57328091 |
| MIR24-2 | 2.70870556 | -1.1594534 | 28.7183276 | 8.37E-08 | 2.21E-06 | 0.12522362 | 0.73784197 |
| CRYBB2 | 2.71250674 | -0.4197164 | 19.1893832 | 1.18E-05 | 0.00020438 | 0.22909894 | 1.11050168 |
| CDC25C | 2.71399828 | 1.63628815 | 52.5895362 | 4.11E-13 | 2.23E-11 | 0.86189437 | 2.68372957 |
| EDA | 2.72125952 | -0.2379923 | 28.5916136 | 8.94E-08 | 2.34E-06 | 0.25428475 | 1.24917824 |
| DEPDC1 | 2.72500847 | 2.66702511 | 30.5437514 | 3.26E-08 | 9.21E-07 | 1.35718558 | 3.60954296 |
| RAB37 | 2.72508019 | -1.4707854 | 14.5469887 | 0.00013671 | 0.00176308 | 0.11075424 | 0.58191747 |
| ADORA1 | 2.73606452 | 2.65719336 | 19.3579704 | 1.08E-05 | 0.00018994 | 1.29825661 | 3.61878923 |
| TMPO-AS1 | 2.7529747 | 0.99190179 | 64.2076623 | 1.12E-15 | 8.11E-14 | 0.59439553 | 2.15524002 |
| UBE2C | 2.75952117 | 4.35156123 | 57.8972208 | 2.76E-14 | 1.78E-12 | 2.64393219 | 5.24709371 |
| MIR27A | 2.76181442 | -2.35097 | 11.508597 | 0.00069275 | 0.0072112 | 0.03498547 | 0.28513125 |
| DDX59-AS1 | 2.76440856 | -1.9984899 | 11.6356453 | 0.000647 | 0.00680922 | 0.05846758 | 0.38820025 |
| PLCL2 | 2.76724405 | 2.00658248 | 79.8188689 | 4.10E-19 | 4.29E-17 | 1.0154241 | 3.03387717 |
| NCAPH | 2.77034847 | 3.01210267 | 86.8915342 | 1.15E-20 | 1.37E-18 | 1.62786845 | 3.96099077 |
| SKA3 | 2.77137623 | 2.6040172 | 70.6230637 | 4.32E-17 | 3.62E-15 | 1.33989985 | 3.58154872 |
| RSPO2 | 2.77319954 | 1.63579371 | 46.6459638 | 8.50E-12 | 3.97E-10 | 0.81987828 | 2.70064556 |
| LGI2 | 2.77440874 | -1.9968269 | 14.4924338 | 0.00014072 | 0.00181044 | 0.05306911 | 0.39486697 |
| GTSE1 | 2.78019595 | 4.27746341 | 95.6229933 | 1.39E-22 | 2.05E-20 | 2.59943005 | 5.18252211 |
| KCND1 | 2.78024744 | 1.00099849 | 61.4047368 | 4.65E-15 | 3.21E-13 | 0.58373079 | 2.16360284 |
| FSD1 | 2.78354073 | -1.6837607 | 11.2860842 | 0.0007809 | 0.00795142 | 0.08367749 | 0.49684131 |
| ADH1C | 2.78554691 | -0.8955884 | 25.7702544 | 3.85E-07 | 8.88E-06 | 0.17102227 | 0.8659831 |
| EFHB | 2.78628442 | -1.4820975 | 20.7579805 | 5.21E-06 | 9.89E-05 | 0.08834966 | 0.59326179 |
| CCDC102B | 2.78727632 | 2.1711575 | 65.7319751 | 5.17E-16 | 3.83E-14 | 1.08995969 | 3.18282563 |
| CDCA3 | 2.79086966 | 3.81092923 | 92.0854969 | 8.30E-22 | 1.12E-19 | 2.21426156 | 4.72658072 |
| HERC2P4 | 2.79253398 | -0.2610886 | 28.5704573 | 9.04E-08 | 2.37E-06 | 0.23573064 | 1.24106882 |
| KLRC2 | 2.79645957 | -1.0793726 | 27.6010002 | 1.49E-07 | 3.76E-06 | 0.13797964 | 0.77462946 |
| ERAP2 | 2.81369368 | 4.21617234 | 89.9373283 | 2.46E-21 | 3.23E-19 | 2.52558136 | 5.12483901 |
| NACAD | 2.82014029 | 1.9030523 | 51.3577297 | 7.70E-13 | 4.03E-11 | 0.94383536 | 2.93745879 |
| PSCA | 2.82231944 | 0.3556572 | 37.8175817 | 7.77E-10 | 2.77E-08 | 0.38944218 | 1.65591657 |
| CPLX1 | 2.82376883 | -2.2993913 | 11.0246358 | 0.00089909 | 0.00893733 | 0.04539526 | 0.29727265 |
| BIRC5 | 2.83741715 | 5.64729946 | 35.6761681 | 2.33E-09 | 7.78E-08 | 3.6361791 | 6.53663081 |
| SAPCD2 | 2.84910195 | 2.98522913 | 85.0731688 | 2.88E-20 | 3.25E-18 | 1.55085203 | 3.94864424 |
| CEP55 | 2.84964182 | 4.04035782 | 33.997484 | 5.52E-09 | 1.72E-07 | 2.23718485 | 4.95556781 |
| C15orf54 | 2.86339674 | -1.6583247 | 18.0750616 | 2.12E-05 | 0.00033933 | 0.07049731 | 0.52191509 |
| GPSM2 | 2.87399801 | 3.4078523 | 60.8395967 | 6.19E-15 | 4.22E-13 | 1.80838581 | 4.3465196 |
| BDKRB1 | 2.88097558 | 3.65840805 | 79.2952352 | 5.35E-19 | 5.51E-17 | 2.02571199 | 4.58989228 |
| TEK | 2.882497 | 2.51171144 | 33.6110596 | 6.73E-09 | 2.08E-07 | 1.17782205 | 3.4946536 |
| REM1 | 2.88540163 | -1.32689 | 14.324728 | 0.00015383 | 0.00195156 | 0.11478107 | 0.65432977 |
| KIF2C | 2.89748684 | 4.34044774 | 84.1907127 | 4.49E-20 | 4.96E-18 | 2.55671852 | 5.25719689 |
| KCNA1 | 2.89814466 | -0.1793772 | 18.4031769 | 1.79E-05 | 0.00029368 | 0.23492298 | 1.27605284 |
| DMBT1 | 2.90542996 | -2.2641601 | 11.7338959 | 0.00061372 | 0.00649152 | 0.03687395 | 0.31084309 |
| STMN1 | 2.9116744 | 6.77192279 | 71.6341362 | 2.59E-17 | 2.22E-15 | 4.74080418 | 7.66522333 |
| RBM44 | 2.91659634 | -1.6690075 | 11.332559 | 0.0007616 | 0.00779266 | 0.06174472 | 0.51310511 |
| LINC00840 | 2.92605325 | -1.900489 | 16.1000041 | 6.01E-05 | 0.00085818 | 0.05306911 | 0.43072812 |
| CDK1 | 2.9290155 | 3.66329045 | 79.7817429 | 4.18E-19 | 4.35E-17 | 1.99057187 | 4.59560752 |
| BABAM2-AS1 | 2.93356883 | -0.438245 | 36.0095252 | 1.96E-09 | 6.62E-08 | 0.20252083 | 1.13704351 |
| SFTA1P | 2.93709354 | 3.39410702 | 49.111815 | 2.42E-12 | 1.19E-10 | 1.75227153 | 4.34119809 |
| NDC80 | 2.94054503 | 3.42535653 | 99.0919962 | 2.41E-23 | 3.81E-21 | 1.81903305 | 4.37315675 |
| ZNF300P1 | 2.94365248 | -1.1591636 | 30.6249902 | 3.13E-08 | 8.85E-07 | 0.11051464 | 0.74678065 |
| KIF14 | 2.94985625 | 2.27384287 | 46.2786334 | 1.03E-11 | 4.74E-10 | 1.05521152 | 3.25616633 |
| B3GALT2 | 2.95985904 | 2.6282997 | 73.8452758 | 8.45E-18 | 7.71E-16 | 1.26032234 | 3.61488311 |
| NEAT1 | 2.96232209 | 9.32666011 | 85.2543281 | 2.62E-20 | 3.02E-18 | 7.22511815 | 10.2238563 |
| LMNB1 | 2.96758718 | 4.07303329 | 53.3311477 | 2.82E-13 | 1.57E-11 | 2.23018996 | 5.00295472 |
| MYRIP | 2.9756207 | -1.8662285 | 18.1600615 | 2.03E-05 | 0.00032726 | 0.05306911 | 0.44662549 |
| CIT | 2.98201663 | 4.17377477 | 93.8683062 | 3.37E-22 | 4.75E-20 | 2.36151336 | 5.10208901 |
| CDCA8 | 2.98637861 | 3.60018725 | 56.2478045 | 6.39E-14 | 3.92E-12 | 1.86099683 | 4.54571487 |
| HJURP | 2.98695993 | 3.75070394 | 111.453837 | 4.71E-26 | 9.69E-24 | 2.03155971 | 4.69669685 |
| PITX2 | 2.98972409 | 3.61675702 | 88.5297336 | 5.01E-21 | 6.18E-19 | 1.90785719 | 4.56671641 |
| TYMS | 2.98990994 | 4.00349511 | 74.0097442 | 7.77E-18 | 7.22E-16 | 2.19980765 | 4.92974579 |
| CLDN7 | 2.99213152 | -0.5199406 | 36.4297457 | 1.58E-09 | 5.41E-08 | 0.17856692 | 1.09437586 |
| KIF4A | 2.99293598 | 3.68374093 | 96.8748383 | 7.39E-23 | 1.14E-20 | 1.96233375 | 4.63242661 |
| PCLAF | 3.00502431 | 3.41279971 | 110.658319 | 7.03E-26 | 1.43E-23 | 1.7751608 | 4.37058541 |
| GAS8-AS1 | 3.00929217 | -1.8671164 | 11.3062602 | 0.00077246 | 0.00788843 | 0.05065295 | 0.43560584 |
| CDC20 | 3.01192868 | 5.08478567 | 53.0800186 | 3.20E-13 | 1.76E-11 | 3.05147723 | 5.99673833 |
| ANLN | 3.01390162 | 5.67535736 | 22.9463844 | 1.67E-06 | 3.46E-05 | 3.38047898 | 6.57383919 |
| NCALD | 3.01872396 | 3.59698176 | 108.551715 | 2.03E-25 | 3.96E-23 | 1.8968853 | 4.5476445 |
| INPP5D | 3.01936546 | -1.8321047 | 19.4061333 | 1.06E-05 | 0.00018581 | 0.05846758 | 0.46013963 |
| EN1 | 3.02207358 | 3.21505498 | 100.136298 | 1.42E-23 | 2.30E-21 | 1.62656852 | 4.18227833 |
| A2M | 3.03016356 | 3.17023598 | 87.1777847 | 9.92E-21 | 1.19E-18 | 1.56634354 | 4.14127313 |
| ABLIM2 | 3.04536966 | -2.1836367 | 15.3838631 | 8.77E-05 | 0.00120052 | 0.03498547 | 0.34150822 |
| AIF1L | 3.04538107 | -1.3080791 | 20.856526 | 4.95E-06 | 9.46E-05 | 0.0839097 | 0.67340171 |
| PBK | 3.05607703 | 3.53292557 | 94.5922474 | 2.34E-22 | 3.39E-20 | 1.81135077 | 4.48822183 |
| C16orf74 | 3.0722907 | -0.5416218 | 27.9729228 | 1.23E-07 | 3.15E-06 | 0.17933491 | 1.07340962 |
| LINC00173 | 3.07329042 | -1.8043442 | 16.1940853 | 5.72E-05 | 0.00082219 | 0.05306911 | 0.46835155 |
| E2F8 | 3.08194532 | 0.81578797 | 31.6143275 | 1.88E-08 | 5.53E-07 | 0.39807155 | 2.05042015 |
| RANBP3L | 3.08573715 | 1.77993344 | 31.6343431 | 1.86E-08 | 5.48E-07 | 0.73416527 | 2.83450349 |
| CENPF | 3.09252337 | 5.43033467 | 102.44315 | 4.44E-24 | 7.40E-22 | 3.3594493 | 6.34899208 |
| EPB41L3 | 3.09982616 | 2.45314582 | 55.971518 | 7.35E-14 | 4.44E-12 | 1.06314787 | 3.46718316 |
| TCF21 | 3.10553008 | -0.9147334 | 16.9782625 | 3.78E-05 | 0.00056583 | 0.11289107 | 0.85678946 |
| KIF18B | 3.10695449 | 3.14864328 | 106.68652 | 5.21E-25 | 9.60E-23 | 1.52383692 | 4.12699177 |
| CROCC2 | 3.11083285 | -1.7742354 | 21.5632385 | 3.42E-06 | 6.75E-05 | 0.05306911 | 0.48607932 |
| TENT5C | 3.11382211 | -0.185372 | 55.2447096 | 1.06E-13 | 6.28E-12 | 0.21520296 | 1.31701256 |
| LINC02568 | 3.11427379 | -1.7797894 | 12.7117292 | 0.00036337 | 0.00415992 | 0.05306911 | 0.46988396 |
| LRRTM2 | 3.12732868 | 0.38091933 | 40.9119543 | 1.59E-10 | 6.31E-09 | 0.29124925 | 1.71812833 |
| BUB1 | 3.14088598 | 3.79254739 | 45.2549168 | 1.73E-11 | 7.79E-10 | 1.86677213 | 4.75074258 |
| GPAT2 | 3.14227382 | 1.47387508 | 34.300028 | 4.72E-09 | 1.48E-07 | 0.62218833 | 2.59414833 |
| SPTB | 3.14377663 | -1.4549798 | 21.5313945 | 3.48E-06 | 6.84E-05 | 0.0772579 | 0.610659 |
| PRC1 | 3.14408311 | 5.50147042 | 45.8244774 | 1.29E-11 | 5.90E-10 | 3.26690991 | 6.42950816 |
| CKAP2L | 3.15599887 | 3.36154304 | 43.9735644 | 3.33E-11 | 1.44E-09 | 1.55643028 | 4.33156609 |
| STAB1 | 3.16398899 | 0.02428996 | 37.1637099 | 1.09E-09 | 3.81E-08 | 0.22339809 | 1.45952785 |
| ADAM33 | 3.16474467 | 2.91435147 | 104.380086 | 1.67E-24 | 2.92E-22 | 1.35039044 | 3.90995666 |
| C10orf142 | 3.16851986 | -2.1088115 | 16.4503475 | 4.99E-05 | 0.00073071 | 0.03498547 | 0.3665382 |
| EGR1 | 3.17415368 | 7.99612091 | 64.2969881 | 1.07E-15 | 7.77E-14 | 5.66850562 | 8.91476079 |
| ERG | 3.17953929 | 0.68377948 | 43.6417655 | 3.94E-11 | 1.68E-09 | 0.38504045 | 1.92241987 |
| HS3ST1 | 3.18244605 | 0.65911936 | 55.107308 | 1.14E-13 | 6.69E-12 | 0.36701801 | 1.92220538 |
| CENPA | 3.18777228 | 2.43156432 | 89.9879301 | 2.40E-21 | 3.16E-19 | 1.04640081 | 3.45895464 |
| SCARA5 | 3.18997211 | 0.44738905 | 52.6602828 | 3.97E-13 | 2.16E-11 | 0.30319991 | 1.7679366 |
| BUB1B | 3.19481735 | 3.56801899 | 70.0471451 | 5.79E-17 | 4.81E-15 | 1.70927707 | 4.54076905 |
| IL20RA | 3.19992787 | -1.1862052 | 32.7603763 | 1.04E-08 | 3.15E-07 | 0.08053503 | 0.7474427 |
| GRIA1 | 3.2011288 | 0.8032595 | 80.4356279 | 3.00E-19 | 3.18E-17 | 0.40939233 | 2.04678338 |
| TOP2A | 3.20896307 | 5.6264946 | 24.7426073 | 6.55E-07 | 1.46E-05 | 3.17297343 | 6.54760523 |
| ESPL1 | 3.22447876 | 2.91273904 | 51.9544698 | 5.68E-13 | 3.03E-11 | 1.25257857 | 3.91725476 |
| PSTPIP1 | 3.22536284 | -2.0668528 | 17.1441981 | 3.46E-05 | 0.00052409 | 0.03687395 | 0.38242685 |
| NUF2 | 3.22580274 | 2.53879533 | 52.1678828 | 5.10E-13 | 2.74E-11 | 1.04743278 | 3.55637586 |
| DEPDC1B | 3.22963984 | 0.69340665 | 73.1842984 | 1.18E-17 | 1.05E-15 | 0.36259156 | 1.96344814 |
| KNL1 | 3.23882713 | 2.70032721 | 17.2065796 | 3.35E-05 | 0.00050863 | 1.05267277 | 3.67879028 |
| GNGT2 | 3.25639516 | -1.3960202 | 25.6416734 | 4.11E-07 | 9.46E-06 | 0.06174472 | 0.64947232 |
| WNT2B | 3.26134297 | 1.02752207 | 73.8591597 | 8.39E-18 | 7.69E-16 | 0.46410655 | 2.22302444 |
| GABRB3 | 3.26593825 | -1.3656553 | 21.5805644 | 3.39E-06 | 6.70E-05 | 0.0772579 | 0.65130482 |
| FBLN2 | 3.27491235 | 5.75986138 | 129.475697 | 5.34E-30 | 1.56E-27 | 3.5167368 | 6.69821143 |
| SLC1A2 | 3.30002554 | -0.3877447 | 28.011968 | 1.21E-07 | 3.09E-06 | 0.16111154 | 1.1857851 |
| CD70 | 3.30469971 | 3.01840857 | 118.938057 | 1.08E-27 | 2.49E-25 | 1.33102156 | 4.02365144 |
| NEURL1B | 3.30492223 | 2.12462202 | 104.02679 | 2.00E-24 | 3.41E-22 | 0.85510423 | 3.18808411 |
| SLC1A3 | 3.30522049 | 5.23235231 | 54.4307273 | 1.61E-13 | 9.21E-12 | 2.91641521 | 6.17903118 |
| TROAP | 3.30648395 | 3.25519216 | 45.8361182 | 1.29E-11 | 5.88E-10 | 1.41325838 | 4.24778462 |
| SCRG1 | 3.30697322 | 1.68630683 | 93.5588138 | 3.94E-22 | 5.52E-20 | 0.66012251 | 2.79652663 |
| HMMR | 3.31289872 | 2.77825594 | 26.5584256 | 2.56E-07 | 6.08E-06 | 1.08441369 | 3.77257579 |
| PPL | 3.33828755 | 4.90572399 | 132.402172 | 1.22E-30 | 3.72E-28 | 2.71689508 | 5.85521762 |
| DLGAP5 | 3.34378027 | 3.86818646 | 104.612646 | 1.49E-24 | 2.64E-22 | 1.86798255 | 4.83497488 |
| SMCO2 | 3.34483986 | -1.3220928 | 24.1712694 | 8.81E-07 | 1.90E-05 | 0.06873659 | 0.67923806 |
| NUSAP1 | 3.34824148 | 4.13810766 | 85.5263744 | 2.29E-20 | 2.65E-18 | 2.04164945 | 5.10602047 |
| MADCAM1 | 3.34866375 | -2.0013137 | 17.0744725 | 3.59E-05 | 0.00054096 | 0.03186264 | 0.40684886 |
| DAPL1 | 3.3567698 | -1.6117395 | 14.754134 | 0.00012248 | 0.00160622 | 0.05306911 | 0.54207414 |
| ADGRG6 | 3.36155579 | 0.50318744 | 40.3023857 | 2.18E-10 | 8.51E-09 | 0.28655842 | 1.79785185 |
| TNFRSF11A | 3.36164106 | -1.0544777 | 36.37895 | 1.62E-09 | 5.53E-08 | 0.08616479 | 0.81603938 |
| TLE2 | 3.36559593 | 0.91682351 | 71.2065039 | 3.22E-17 | 2.73E-15 | 0.41234262 | 2.14113919 |
| ASPM | 3.38020118 | 4.0725992 | 49.5250106 | 1.96E-12 | 9.72E-11 | 1.94174129 | 4.99837324 |
| SCARNA22 | 3.38942284 | -1.9697037 | 14.9755563 | 0.00010891 | 0.00144914 | 0.03498547 | 0.4118404 |
| MKI67 | 3.39557232 | 5.82584006 | 139.994599 | 2.67E-32 | 9.66E-30 | 3.4804446 | 6.7746971 |
| CDKN3 | 3.3964183 | 3.59738448 | 95.63828 | 1.38E-22 | 2.05E-20 | 1.6419995 | 4.58545554 |
| PART1 | 3.40603247 | -0.0966303 | 34.722206 | 3.80E-09 | 1.21E-07 | 0.17993512 | 1.3761484 |
| LYPD6B | 3.41574359 | 2.73839179 | 67.5290061 | 2.08E-16 | 1.64E-14 | 1.0963128 | 3.7573723 |
| ECRG4 | 3.44230291 | -1.9317099 | 18.8484106 | 1.42E-05 | 0.00023887 | 0.03498547 | 0.43217635 |
| ZFP36 | 3.45986674 | 7.34703628 | 89.0758728 | 3.80E-21 | 4.72E-19 | 4.80835491 | 8.29275758 |
| PLK1 | 3.46195347 | 4.72167719 | 120.659365 | 4.54E-28 | 1.09E-25 | 2.45994756 | 5.68276825 |
| PIMREG | 3.4712947 | 3.72249938 | 129.102911 | 6.44E-30 | 1.85E-27 | 1.69864844 | 4.70653663 |
| NTNG1 | 3.47813015 | 2.59458921 | 109.30767 | 1.39E-25 | 2.75E-23 | 1.01159517 | 3.63615366 |
| TCP11 | 3.48825694 | -2.4437952 | 12.4038999 | 0.00042844 | 0.00478483 | 0.01619516 | 0.26912923 |
| CDHR3 | 3.51309694 | -0.0734143 | 58.9811952 | 1.59E-14 | 1.04E-12 | 0.19181563 | 1.40683182 |
| PI16 | 3.53405305 | 0.28338915 | 32.8970678 | 9.72E-09 | 2.95E-07 | 0.24909583 | 1.64857128 |
| ASPN | 3.54361136 | 2.08854029 | 58.7664726 | 1.78E-14 | 1.16E-12 | 0.70107862 | 3.16342119 |
| CRIP1 | 3.54800294 | 5.74108777 | 20.6563302 | 5.50E-06 | 0.00010376 | 2.84722094 | 6.67544087 |
| IQGAP3 | 3.555581 | 4.37282006 | 136.315457 | 1.70E-31 | 5.42E-29 | 2.11215915 | 5.35035346 |
| KRT14 | 3.56089496 | 4.78930336 | 46.6268843 | 8.59E-12 | 3.99E-10 | 2.32376732 | 5.74820899 |
| LINC01423 | 3.56687666 | 2.86574101 | 25.2322162 | 5.08E-07 | 1.15E-05 | 0.98475665 | 3.88471109 |
| INSRR | 3.57254916 | 0.63123811 | 50.5456249 | 1.16E-12 | 5.99E-11 | 0.27733668 | 1.91608509 |
| LINC01535 | 3.57981777 | 0.35317136 | 58.0265872 | 2.59E-14 | 1.68E-12 | 0.25532196 | 1.71129271 |
| SNTG2 | 3.60931587 | -0.6525083 | 41.1946445 | 1.38E-10 | 5.51E-09 | 0.10133789 | 1.0439907 |
| CDH5 | 3.62414122 | -1.8386966 | 12.2100266 | 0.00047533 | 0.00522519 | 0.03186264 | 0.45721272 |
| MFSD4A | 3.62593982 | -1.8048184 | 22.995806 | 1.62E-06 | 3.39E-05 | 0.03498547 | 0.48454551 |
| KLF17 | 3.62910131 | -0.2881647 | 40.660706 | 1.81E-10 | 7.12E-09 | 0.13440673 | 1.26462974 |
| TFAP2C | 3.6365416 | -2.349594 | 15.740429 | 7.27E-05 | 0.00101562 | 0.01879031 | 0.29943388 |
| PTPRZ1 | 3.63913465 | -1.1065104 | 28.2063914 | 1.09E-07 | 2.82E-06 | 0.06582606 | 0.79133624 |
| SOAT2 | 3.63920325 | -2.3540718 | 15.3102207 | 9.12E-05 | 0.00124369 | 0.01619516 | 0.29771591 |
| HCLS1 | 3.6709307 | 0.97493888 | 76.5816384 | 2.11E-18 | 2.07E-16 | 0.35513698 | 2.20040755 |
| WNT7B | 3.67803254 | -0.5701646 | 56.2090149 | 6.52E-14 | 3.97E-12 | 0.12055144 | 1.09708853 |
| FLT1 | 3.69298163 | -0.4023489 | 45.528579 | 1.50E-11 | 6.84E-10 | 0.11604687 | 1.19807027 |
| FBXO43 | 3.70921201 | -0.1982087 | 59.3606846 | 1.31E-14 | 8.75E-13 | 0.15043352 | 1.33289841 |
| DNM1 | 3.70958028 | 2.51743937 | 127.779115 | 1.25E-29 | 3.45E-27 | 0.85920035 | 3.58011573 |
| LINC02587 | 3.70996584 | -1.7416943 | 18.5961402 | 1.62E-05 | 0.00026941 | 0.03687395 | 0.50604811 |
| TNXB | 3.71342251 | 1.17951726 | 70.773708 | 4.01E-17 | 3.37E-15 | 0.37977578 | 2.37107502 |
| CRTAM | 3.71991371 | 0.53729785 | 66.2745628 | 3.92E-16 | 2.98E-14 | 0.25487588 | 1.85519434 |
| MEOX2 | 3.7533523 | 4.21973991 | 89.6232701 | 2.88E-21 | 3.71E-19 | 1.80920832 | 5.21918105 |
| S1PR1 | 3.76298037 | 2.47099117 | 53.8637433 | 2.15E-13 | 1.21E-11 | 0.7601891 | 3.54399938 |
| HRH2 | 3.7699183 | 0.85144324 | 87.5464157 | 8.23E-21 | 9.93E-19 | 0.31333743 | 2.11304714 |
| HCG22 | 3.78066249 | -1.6901 | 26.6019287 | 2.50E-07 | 5.97E-06 | 0.03687395 | 0.53245971 |
| FOS | 3.79597602 | 7.99342436 | 80.0855123 | 3.59E-19 | 3.76E-17 | 5.09378555 | 8.9652837 |
| LYPD6 | 3.80166485 | 2.24088491 | 94.2919574 | 2.72E-22 | 3.89E-20 | 0.71626854 | 3.32691433 |
| KIF20A | 3.80703249 | 4.59990148 | 50.4568255 | 1.22E-12 | 6.25E-11 | 1.98350192 | 5.59460026 |
| RYR2 | 3.82995712 | -2.2282099 | 11.0421146 | 0.00089065 | 0.00887103 | 0.01879031 | 0.32632203 |
| RFX8 | 3.83238389 | 4.32842641 | 118.600904 | 1.28E-27 | 2.92E-25 | 1.85811142 | 5.32944297 |
| FAM83D | 3.83671357 | 3.01347805 | 119.423381 | 8.46E-28 | 2.00E-25 | 1.02367844 | 4.05918409 |
| NEK2 | 3.83716202 | 2.39027487 | 62.98827 | 2.08E-15 | 1.48E-13 | 0.7209282 | 3.46817392 |
| COL26A1 | 3.83736886 | -1.2400204 | 31.3626575 | 2.14E-08 | 6.22E-07 | 0.05430215 | 0.73384268 |
| RGMA | 3.85143018 | 1.28473122 | 87.8879694 | 6.93E-21 | 8.51E-19 | 0.39201874 | 2.47849853 |
| LINC01940 | 3.85783312 | -0.9171898 | 29.6366776 | 5.21E-08 | 1.42E-06 | 0.07049731 | 0.89106921 |
| KIF4B | 3.85820741 | -0.9226201 | 33.233568 | 8.17E-09 | 2.51E-07 | 0.06873659 | 0.89086555 |
| FOSB | 3.85906577 | 5.82107749 | 166.40742 | 4.51E-38 | 2.56E-35 | 3.08996987 | 6.80490852 |
| MIR3189 | 3.89607168 | -1.6134455 | 25.3417628 | 4.80E-07 | 1.09E-05 | 0.03498547 | 0.56262154 |
| VSNL1 | 3.90242773 | 1.02327515 | 85.5616192 | 2.25E-20 | 2.62E-18 | 0.31134871 | 2.26071304 |
| WIF1 | 3.90745221 | -2.1823973 | 18.4099849 | 1.78E-05 | 0.00029286 | 0.01879031 | 0.35387987 |
| P2RY2 | 3.91999073 | 1.63857129 | 104.148587 | 1.88E-24 | 3.23E-22 | 0.47694453 | 2.79029531 |
| C12orf56 | 3.93417466 | -0.589268 | 51.9587989 | 5.67E-13 | 3.03E-11 | 0.08731592 | 1.09591838 |
| SLAMF9 | 3.93790089 | -1.128818 | 37.9533855 | 7.25E-10 | 2.60E-08 | 0.07576876 | 0.78899526 |
| LSP1 | 3.95856005 | 4.92730199 | 62.2034945 | 3.10E-15 | 2.17E-13 | 2.15627794 | 5.9214316 |
| SYNE2 | 3.96365271 | 2.65318602 | 45.2195149 | 1.76E-11 | 7.92E-10 | 0.75209865 | 3.7190683 |
| FBN2 | 3.99187503 | 6.18789634 | 57.2146981 | 3.91E-14 | 2.47E-12 | 3.15925296 | 7.16111579 |
| ADGRG2 | 4.00263352 | 1.85169345 | 66.1883868 | 4.10E-16 | 3.10E-14 | 0.46371736 | 2.9874872 |
| GABBR2 | 4.00357709 | 1.34766005 | 92.6458011 | 6.25E-22 | 8.53E-20 | 0.37152972 | 2.53547184 |
| TSPOAP1 | 4.03476134 | 1.4900583 | 81.5986711 | 1.67E-19 | 1.81E-17 | 0.36504188 | 2.67120738 |
| TRH | 4.05103275 | 0.65719108 | 84.9192015 | 3.11E-20 | 3.47E-18 | 0.23960295 | 1.9719831 |
| ITPRIPL1 | 4.08352045 | -2.0746159 | 20.5969013 | 5.67E-06 | 0.00010665 | 0.01619516 | 0.39151828 |
| TMEM176A | 4.08390287 | -2.0738778 | 21.1050372 | 4.35E-06 | 8.41E-05 | 0.01619516 | 0.39251605 |
| GJB2 | 4.0916803 | -0.0526223 | 66.8040101 | 3.00E-16 | 2.34E-14 | 0.12141238 | 1.4498354 |
| FAM43B | 4.17929764 | 0.27386534 | 46.6419924 | 8.52E-12 | 3.97E-10 | 0.17102227 | 1.65935495 |
| LHFPL3-AS2 | 4.19268221 | -2.00172 | 21.4530764 | 3.63E-06 | 7.11E-05 | 0.01619516 | 0.41848713 |
| NGFR | 4.27544147 | 3.48257977 | 164.297536 | 1.30E-37 | 7.01E-35 | 1.09056946 | 4.52559127 |
| CDCA7 | 4.36344811 | 0.183618 | 85.2455078 | 2.64E-20 | 3.02E-18 | 0.11604687 | 1.62973999 |
| CTNND2 | 4.41057768 | 0.76581016 | 61.1506554 | 5.29E-15 | 3.62E-13 | 0.16608795 | 2.06903671 |
| OMD | 4.41344919 | 1.34557353 | 96.8799211 | 7.37E-23 | 1.14E-20 | 0.28489445 | 2.55200891 |
| NR4A1 | 4.41670321 | 5.98363121 | 191.634264 | 1.40E-43 | 1.28E-40 | 2.76918751 | 6.99583543 |
| FABP4 | 4.43215675 | -0.7652455 | 57.0817692 | 4.18E-14 | 2.62E-12 | 0.05306911 | 1.00444643 |
| LPAR3 | 4.52485456 | -1.1314058 | 32.0091919 | 1.53E-08 | 4.56E-07 | 0.03498547 | 0.79055768 |
| TOX2 | 4.53622566 | -0.0732461 | 78.02014 | 1.02E-18 | 1.02E-16 | 0.092431 | 1.44697227 |
| PODXL | 4.63540806 | 5.04745409 | 133.023891 | 8.93E-31 | 2.76E-28 | 1.80725726 | 6.07884295 |
| CFAP73 | 4.68890562 | -2.8856641 | 11.0507116 | 0.00088653 | 0.00883838 | 0 | 0.16325951 |
| MIR3681HG | 4.68890562 | -2.8856641 | 11.0507116 | 0.00088653 | 0.00883838 | 0 | 0.16325951 |
| SOX8 | 4.69095206 | -2.8847159 | 11.2908917 | 0.00077888 | 0.00793835 | 0 | 0.16426867 |
| MIR6835 | 4.69095206 | -2.8847159 | 11.2908917 | 0.00077888 | 0.00793835 | 0 | 0.16426867 |
| LINC01844 | 4.6911874 | -2.8846883 | 11.558035 | 0.00067457 | 0.0070554 | 0 | 0.16477914 |
| CYP2A6 | 4.69497026 | -2.882736 | 10.9416367 | 0.00094027 | 0.00926416 | 0 | 0.1648377 |
| CHRM3 | 4.74845016 | 1.84457288 | 90.6413554 | 1.72E-21 | 2.29E-19 | 0.30319991 | 2.98553772 |
| SNTG2-AS1 | 4.81064131 | 0.41673181 | 78.6089493 | 7.57E-19 | 7.68E-17 | 0.12055144 | 1.79679511 |
| FCGR2C | 4.82064537 | -2.8221211 | 10.7817323 | 0.00102507 | 0.00996957 | 0 | 0.17740756 |
| LINC00618 | 4.82223365 | -2.8220393 | 12.1290868 | 0.00049641 | 0.00540597 | 0 | 0.17894236 |
| SYT9 | 4.82405764 | -2.821132 | 12.6944929 | 0.00036673 | 0.00418606 | 0 | 0.18050277 |
| OIT3 | 4.82775472 | -2.819235 | 11.995951 | 0.00053316 | 0.00574655 | 0 | 0.1805798 |
| NGEF | 4.92986556 | 3.19997605 | 186.830521 | 1.56E-42 | 1.28E-39 | 0.6805033 | 4.28245329 |
| PHF24 | 4.94319797 | -2.7611374 | 11.1329238 | 0.00084809 | 0.00849545 | 0 | 0.19206682 |
| STMN3 | 4.9442673 | 2.78992635 | 168.723537 | 1.41E-38 | 8.68E-36 | 0.51870022 | 3.88745577 |
| LRRC25 | 4.94714711 | -2.7593214 | 13.5993657 | 0.00022626 | 0.00273337 | 0 | 0.19623502 |
| STEAP4 | 4.954085 | 4.62177241 | 89.3935217 | 3.24E-21 | 4.07E-19 | 1.28374713 | 5.66757313 |
| TRIM58 | 4.97875228 | -0.2727142 | 73.2657397 | 1.13E-17 | 1.01E-15 | 0.07060517 | 1.31652549 |
| LINC00619 | 4.98645132 | -1.4171875 | 43.7087322 | 3.81E-11 | 1.63E-09 | 0.01619516 | 0.6714077 |
| LINC01126 | 5.05267143 | -2.7038295 | 11.5912906 | 0.00066261 | 0.0069457 | 0 | 0.20644357 |
| LINC01446 | 5.05534731 | -2.70375 | 14.4574349 | 0.00014336 | 0.00183988 | 0 | 0.20947507 |
| MEIS1-AS3 | 5.05826933 | -2.70188 | 14.3404797 | 0.00015255 | 0.00193764 | 0 | 0.21043141 |
| PROZ | 5.06878325 | -2.6991267 | 11.1671211 | 0.0008326 | 0.00837624 | 0 | 0.20862898 |
| INSC | 5.07960411 | -1.350772 | 30.4784447 | 3.38E-08 | 9.46E-07 | 0.01619516 | 0.69133242 |
| C2orf48 | 5.16458947 | -2.6450017 | 14.6661901 | 0.00012833 | 0.00166937 | 0 | 0.22533092 |
| MYT1L | 5.16871726 | -2.6441052 | 12.8562963 | 0.00033635 | 0.00389301 | 0 | 0.22356214 |
| ACAN | 5.18630806 | 6.80304891 | 275.957853 | 5.71E-62 | 1.50E-58 | 2.84436671 | 7.83548219 |
| EGR3 | 5.23301259 | 3.75629043 | 125.862942 | 3.29E-29 | 8.76E-27 | 0.7290122 | 4.82301886 |
| KCNN2 | 5.25576428 | -2.5946416 | 16.5464752 | 4.75E-05 | 0.00069652 | 0 | 0.23906456 |
| OR2W3 | 5.26176616 | -2.5910802 | 15.789465 | 7.08E-05 | 0.00099095 | 0 | 0.24053819 |
| EMX2 | 5.30354218 | 2.88679071 | 197.353173 | 7.90E-45 | 7.54E-42 | 0.43697618 | 3.9955924 |
| SYT17 | 5.34033443 | -2.5443885 | 14.1510905 | 0.0001687 | 0.0021197 | 0 | 0.24888011 |
| HTR1F | 5.34503937 | -2.5435424 | 16.87626 | 3.99E-05 | 0.00059411 | 0 | 0.2525418 |
| SGK2 | 5.35600385 | -2.5390292 | 13.3651776 | 0.00025634 | 0.00305802 | 0 | 0.25007513 |
| DENND2A | 5.42726077 | -1.0499872 | 59.1782221 | 1.44E-14 | 9.54E-13 | 0.02660494 | 0.85746856 |
| PITX1 | 5.47442198 | 3.185101 | 125.571256 | 3.82E-29 | 9.89E-27 | 0.49109394 | 4.26868243 |
| RUNX1-IT1 | 5.51395062 | -2.4441066 | 19.810524 | 8.55E-06 | 0.00015438 | 0 | 0.28336773 |
| ABCB4 | 5.5877773 | -2.3989529 | 19.7674816 | 8.75E-06 | 0.00015762 | 0 | 0.29563582 |
| TMEM176B | 5.5916118 | -2.3963643 | 21.9498435 | 2.80E-06 | 5.58E-05 | 0 | 0.29943388 |
| LERFS | 5.65282525 | -0.8761716 | 57.3478271 | 3.65E-14 | 2.34E-12 | 0.01619516 | 0.9535879 |
| MIR8085 | 5.73210595 | -2.308731 | 23.2796184 | 1.40E-06 | 2.95E-05 | 0 | 0.32590804 |
| MMP9 | 5.92219455 | -2.1845675 | 26.1680123 | 3.13E-07 | 7.31E-06 | 0 | 0.36735711 |
| ACRV1 | 5.98221128 | -2.1445512 | 24.2754865 | 8.35E-07 | 1.81E-05 | 0 | 0.37905264 |
| ISL1 | 6.03783159 | -2.1066225 | 26.1674895 | 3.13E-07 | 7.31E-06 | 0 | 0.39344081 |
| PNLDC1 | 6.0414396 | -0.5407894 | 77.9662287 | 1.05E-18 | 1.04E-16 | 0.01619516 | 1.16112676 |
| GRP | 6.13748351 | -2.0379781 | 28.3519281 | 1.01E-07 | 2.63E-06 | 0 | 0.41724396 |
| GPR4 | 6.13998663 | -2.0364744 | 30.7344458 | 2.96E-08 | 8.43E-07 | 0 | 0.41996341 |
| CRIP3 | 6.18865393 | -2.0019954 | 28.6723868 | 8.57E-08 | 2.25E-06 | 0 | 0.4305744 |
| ALX1 | 6.26917867 | 1.95338677 | 179.702155 | 5.63E-41 | 3.86E-38 | 0.11289107 | 3.14205837 |
| FOXE1 | 6.35630368 | -0.2681697 | 64.4291568 | 1.00E-15 | 7.30E-14 | 0.01619516 | 1.32467988 |
| EMX2OS | 6.44371973 | 3.58255741 | 226.433816 | 3.57E-51 | 4.17E-48 | 0.34024566 | 4.68159231 |
| PSG4 | 6.50128242 | 2.93922161 | 233.525168 | 1.02E-52 | 1.52E-49 | 0.22290664 | 4.06139121 |
| KNDC1 | 6.61809462 | -1.6883432 | 33.8493723 | 5.95E-09 | 1.85E-07 | 0 | 0.55439192 |
| ABI3 | 6.64334186 | 0.73651724 | 123.634799 | 1.01E-28 | 2.59E-26 | 0.03186264 | 2.08737405 |
| ADH1B | 6.65242076 | 0.00858845 | 78.5589308 | 7.76E-19 | 7.84E-17 | 0.01879031 | 1.51983884 |
| GPR21 | 6.69440905 | -1.6324171 | 22.7728808 | 1.82E-06 | 3.76E-05 | 0 | 0.554553 |
| IGSF1 | 6.74143062 | 0.0899046 | 89.2220515 | 3.53E-21 | 4.41E-19 | 0.01879031 | 1.58460456 |
| LINC00707 | 6.91151554 | -1.4599325 | 49.4191496 | 2.07E-12 | 1.02E-10 | 0 | 0.66193572 |
| SLC30A8 | 7.03082618 | -1.3660635 | 41.2813683 | 1.32E-10 | 5.28E-09 | 0 | 0.70008489 |
| ECEL1 | 7.7454975 | -0.7683909 | 80.9899532 | 2.27E-19 | 2.43E-17 | 0 | 1.03002459 |
| MKRN3 | 7.80978373 | -0.7124587 | 65.9274767 | 4.68E-16 | 3.48E-14 | 0 | 1.05507571 |
| LHX9 | 7.83471183 | -0.6922028 | 50.0483003 | 1.50E-12 | 7.59E-11 | 0 | 1.04923555 |
| SHOX2 | 8.21941035 | 1.49611948 | 138.966075 | 4.48E-32 | 1.54E-29 | 0.02660494 | 2.71864207 |
| XIST | 10.5808632 | 3.82296847 | 151.755655 | 7.17E-35 | 3.50E-32 | 0.02660494 | 4.81299358 |
