# Supplementary material for: Patient-Derived Bone Marrow Spheroids Reveal Leukemia-Initiating Cells Supported by Mesenchymal Hypoxic Niches in Pediatric B-ALL
Source: Front Immunol. 2021 Oct 19;12:746492. doi: 10.3389/fimmu.2021.746492 (PMC8561951; doi:10.3389/fimmu.2021.746492)
Supplement: Supplementary file 2 [file DataSheet_2.docx]

Supplementary Material

# Supplementary Figures

**Supplementary Figure 1. Purity of sorted primary B-ALL cells. (A)** Sort gating strategy is shown for ProB-ALL and **(B)** preB ALL (left panel). Blasts population was highly purified using a FACSAria II flow cytometer (BD Biosciences) and purity was confirmed after cell sorting (right panel). **(C)** FACS gating strategy after anti-human CD45-PE staining for PDLS analysis in empty spheroids and occupied PDLS are shown. **(D)** CD45 (left) and CD19 (right) expression after TrypLE Express enzymatic digestion on PDLS-in and PDLS-out cells. B-ALL, B-cell acute lymphoblastic leukemia; PDLS, patient-derived leukemic spheroids.

**Supplementary Figure 2. ALL-MSC form 3D low proliferative spheroids.** **(A)** 50x10^3^ MSC from B-ALL BM were cultured in non-adherent conditions and 24 hours time-lapse recorded. **(B)** HBM and ALL spheroids were formed with different MSC numbers and their sizes were determined (*n*=3). **(C)** representative plots for proliferation analyses in 2D and 3D cultures of HBM- or ALL-MSC by CRFR dilution or Ki-67 staining (*n*=3). **(D)** representative 3D MSC immunophenotype is shown (*n*=5). **(E)** Nestin, PDFGRα and LepR were evaluated by indirect fluorescence microscopy and FACS (n=6). MSC, mesenchymal stromal cells; HBM, healthy bone marrow; B-ALL, B-cell acute lymphoblastic leukemia; BM, bone marrow; CTFR, Cell Trace Far Red; MPB, mobilized peripheral blood.


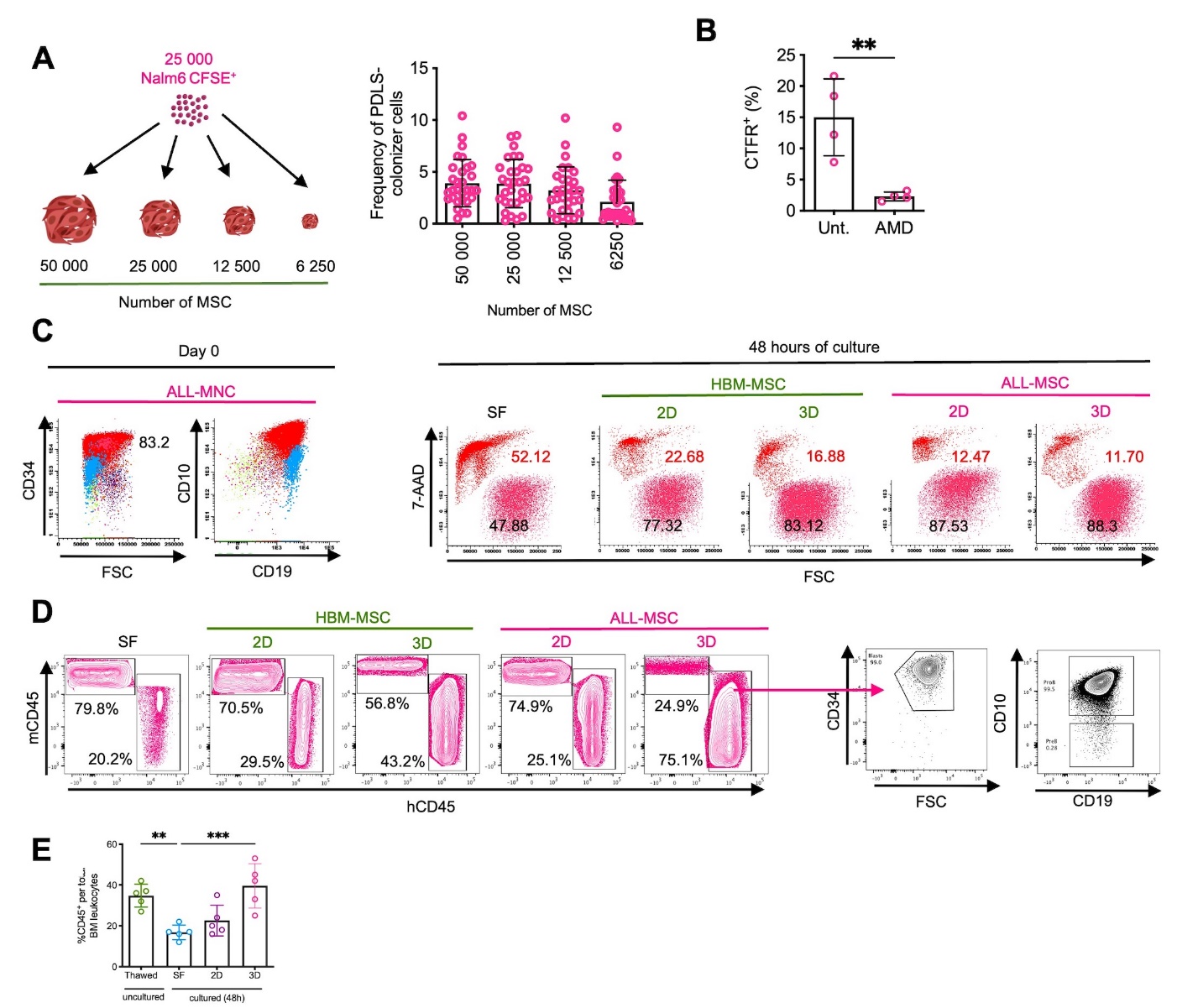


**Supplementary Figure 3. MSC-ALL 3D cultures favor viability and maintenance of primitive B-ALL cells.** **(A)** 25 000 Nalm6 cells CFSE-labeled were co-cultured with stromal spheroids with different MSC cell number composition, upon 24 hours PDLS were washed and enzymatically/mechanically disrupted for FACS analysis, frequency of CFSE^+^ cells was recorded (*n*=30). **(B)** CTFR-labeled primary B-ALL cells were treated with a CXCR4 inhibitor (AMD3100) 5mM for 3h and then co-cultured within HBM-MSC. Upon 24 hours, CTFR^+^ content was determined by FACS (n=4). **(C)** 25 000 MNC from B-ALL were co-cultured in SF, monolayer (2D) and spheroid (3D) conditions with MSC from HBM or ALL for 48 hours. B-ALL immunophenotype and blasts frequency are shown (left panel). Viability analyses by FACS was recorded (right panel). **(D)** After 48 hours, only leukemic cells were harvested and transplanted to NSG mice. 6 weeks after, human engraftment was determined by FACS in mouse BM (upper right panel) and immunophenotype of engrafted B-ALL is also shown (right panel). **(E)** 25 000 primary MNC from B-ALL patients were cultured in SF (blue), 2D (violet) and 3D (pink) conditions while some MNC were cryopreserved. Upon 48 hours culture, MNC cells were harvested and transplanted into NSG mice. Cryopreserved MNC were thawed, and 25 000 cells were xeno-transplanted. After 6 weeks, engraftment was analyzed (n=5). MNC, mononuclear cells; B-ALL, B-cell acute lymphoblastic leukemia; SF, stromal-free; MSC, mesenchymal stromal cell; HBM, healthy bone marrow; FACS, Fluorescence-activated cell sorting.

**Supplementary Figure 4 | Patient-derived leukemic spheroids create inner quiescent hypoxic niches. (A)** Primary B-ALL blasts were cultured in stromal-free (SF) conditions and co-cultured with ALL-MSC in monolayer (2D) and PDLS settings for 48 h. Representative plots of cell cycle status on CD45^+^ cells are shown. **(B)** HIF-1𝛼 expression was investigated in MSC in 2D and 3D conditions by FACS and fluorescence microscopy (left and middle panel) (*n*=7), FACS analysis on inner B-ALL cells in PDLS is also shown (right). **(C)** Pimonidazole incorporation was evaluated by FACS in different culture derived cells, including SF, 2D, PDLS-out and PDLS-in after co-culture with B-ALL CTFR-labeled cells (*n*=5). B-ALL, B-cell acute lymphoblastic leukemia; SF, stromal-free; MSC, mesenchymal stromal cell; PDLS, patient-derived leukemic spheroid; FACS, Fluorescence-activated cell sorting; CTFR, Cell Trace Far Red.

**
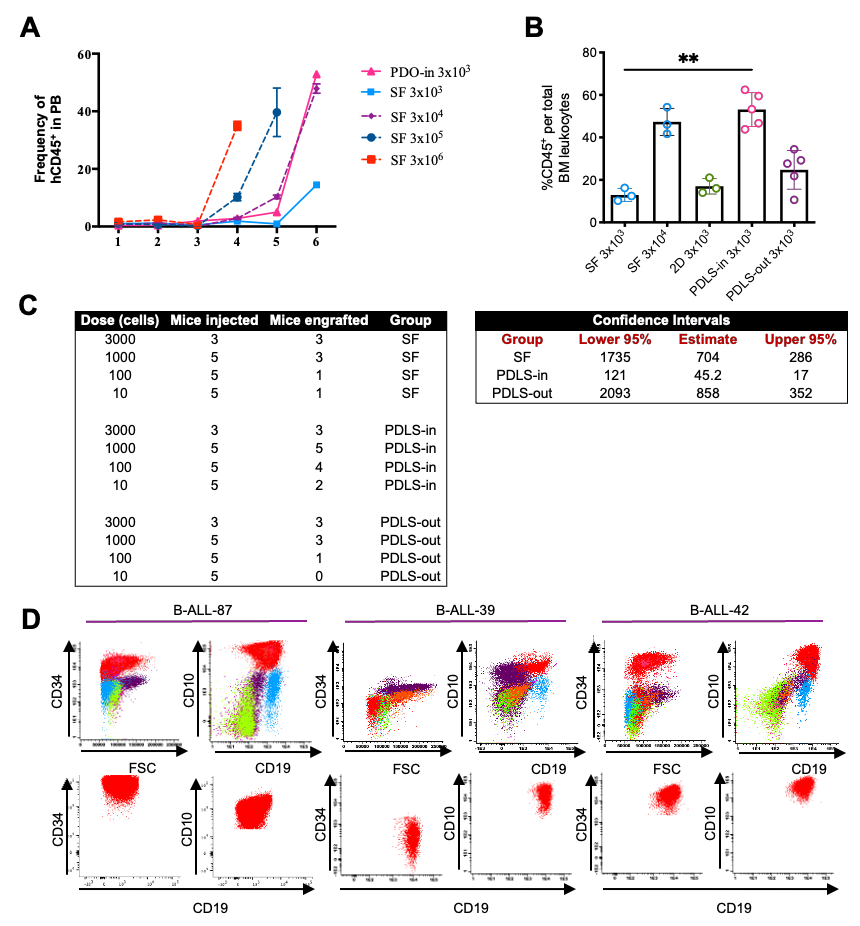
**

**Supplementary Figure 5. Limiting-dilution assay confirmed leukemia-initiating cell (LIC) enrichment within PDLS. (A)** Crescent numbers (3x10^3^, 3x10^4^, 3x10^5^ and 3x10^6^) of RS4;11 leukemic cells were transplanted into NSG mice in parallel with 3x10^3^ RS4;11 cells harvested from PDLS-in cultures. Engraftment was weekly monitored in peripheral blood by FACS (*n*=5). **(B)** BM engraftment was determined after BM aspiration after 6 weeks of transplantation. **(C)**The total number of injected cells per dilution and the number of injected and engrafted mice is shown (left), calculation of the LIC content in each group were obtained from the web-based ELDA (extreme limiting dilution analysis) program (right). **(D)** Immunophenotype of input (upper panel) and engrafted B-ALL cells (lower panel) are shown. NSG, NOD-SCID gamma chain: PDLS, patient-derived leukemic spheroid; FACS, fluorescence-activated cell sorting; BM, bone marrow.

**
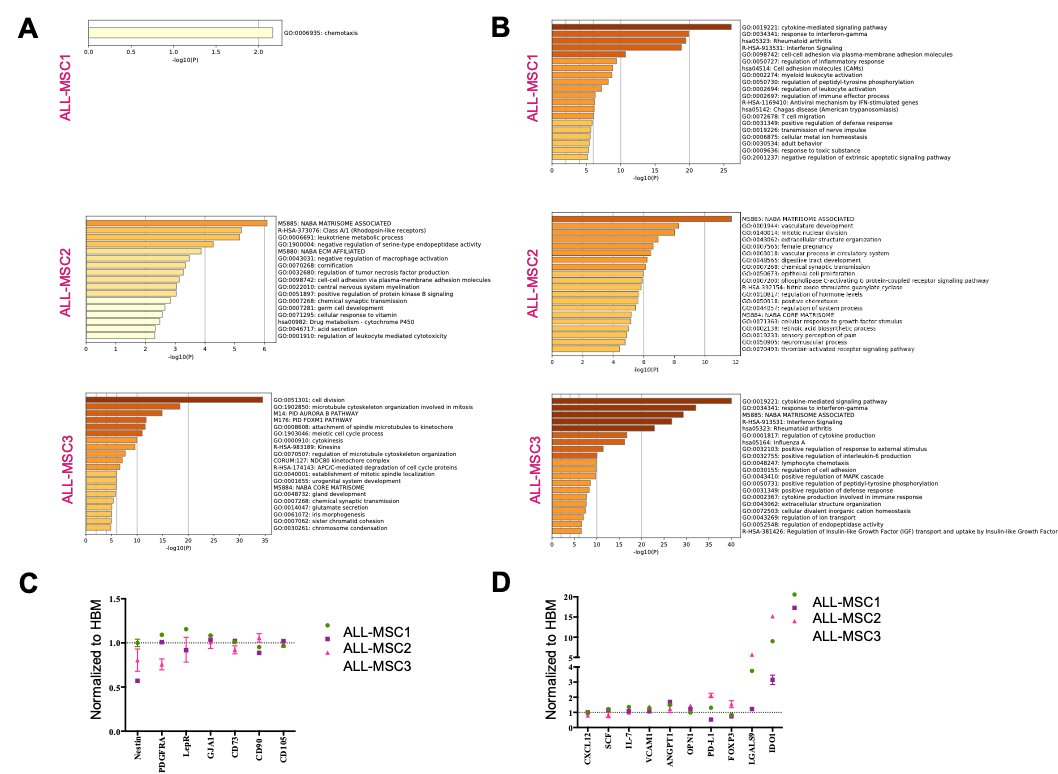
**

**Supplementary Figure 6. RNA-sequencing analysis on ALL-MSC.** **(A)** gene ontology and functional enrichment analysis for downregulated genes and **(B)** upregulated are shown. **(C)**, mRNA normalized expression of CAR-niche phenotype associated molecules and **(D)** niche- and suppressor associated molecules (*n*=3). B-ALL, B-cell acute lymphoblastic leukemia; MSC, mesenchymal stromal cell; HBM, healthy bone marrow; CAR, CXCL12-derived abundant reticular.


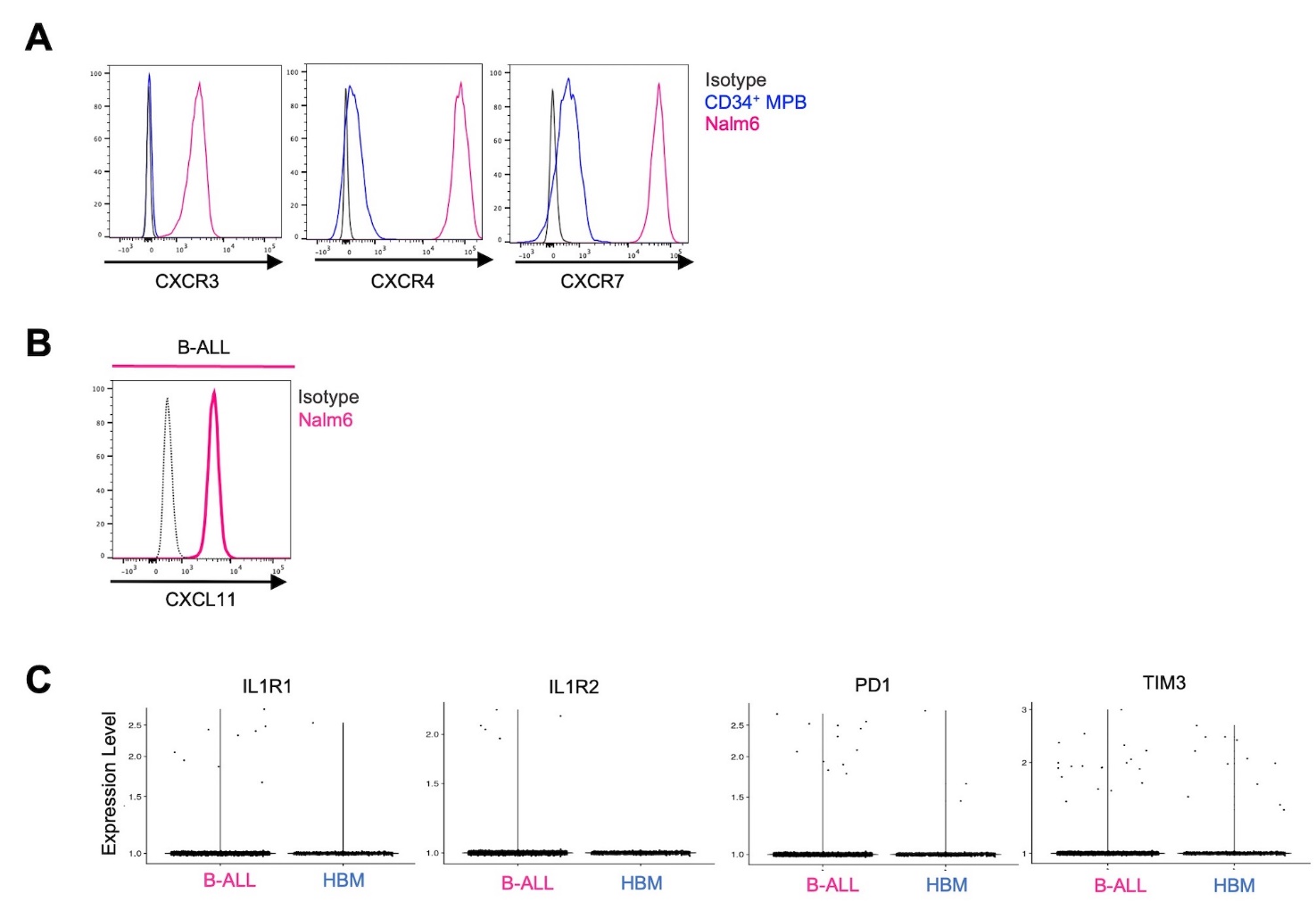


**Supplementary Figure 7. B-ALL cells express CXCL11/CXCL12 receptors. (A)** CD34^+^ cells from MPB were analyzed for CXCR3, CXCR4 and CXCR7 expression by FACS. Nalm-6 receptor expression is also shown for comparison. **(B)** CXCL11 was determined by FACS in Nalm6 cell line. **(C)** Cytokine/chemokine receptor expression and ligands of relevance for ALL BM niches, were analyzed in B-ALL and Healthy BM CD19^+^CD79^+^ populations obtained from database GSE132509. MPB, mobilized peripheral blood.
